# Synthesis of β-Hydroxy α-Amino Acids Through Brønsted Base-Catalyzed *syn*-Selective Direct Aldol Reaction of Schiff Bases of Glycine *o*-Nitroanilide

**DOI:** 10.1021/acs.joc.1c00406

**Published:** 2021-05-16

**Authors:** Silvia Vera, Ana Vázquez, Ricardo Rodriguez, Sandra del Pozo, Iñaki Urruzuno, Abel de Cózar, Antonia Mielgo, Claudio Palomo

**Affiliations:** † Departamento de Química Orgánica I, Universidad del País Vasco UPV/EHU , Manuel Lardizábal 3, 20018 San Sebastián, Spain; ‡ IKERBASQUE, Basque Foundation for Science , 48009 Bilbao, Spain

## Abstract

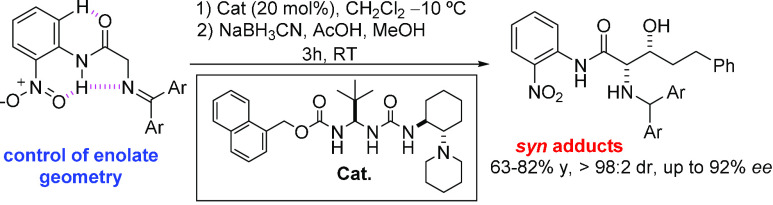

Here we report the highly enantio- and *syn*-selective synthesis of β-hydroxy α-amino acids from glycine imine derivatives under Brønsted base (BB) catalysis. The key of this approach is the use of benzophenone-derived imine of glycine *o*-nitroanilide as a pronucleophile, where the *o*-nitroanilide framework provides an efficient hydrogen-bonding platform that accounts for nucleophile reactivity and diastereoselectivity.

Due to their prevalence in natural products, including antibiotics and enzyme inhibitors and their presence as structural components of many biologically active products, β-hydroxy α-amino acids are compounds of high interest in medicinal chemistry.^1^ Thus, different approaches for their enantioselective synthesis have been reported, most of them relying on the use of glycine derivatives.^2^ Among these and since their introduction by O’Donnel and Eckrich in 1978,^3^ glycine Schiff bases stand up as the most appealing substrates due to their bench stability. In this context, the asymmetric aldol reaction of glycine Schiff bases is very effective for the production of β-hydroxy α-amino acids because, concomitant to the assembly of the 1,2-aminoalcohol functionality during the carbon–carbon bond forming step, up to two vicinal stereogenic centers are created in a single synthetic operation. In 1991, Miller and Gasparski reported the first catalytic direct aldol reaction of benzophenone imines of glycine esters using phase-transfer catalysis. The method leads to *anti*-adducts but in low diastereoselectivities and negligible enantiomeric excesses.^4^ Following this report, other protocols involving phase-transfer catalytic conditions, metal catalysis, or the use of lithium/(−)-sparteine have also been described.^5−7^ Among these, the only report concerning the enantioselective direct synthesis of *syn*-isomers from glycine Schiff bases has been documented, as far as we know, by Trost using a zinc-ProPhenol catalyst.^8^ The reaction works well for α-substituted aldehydes but provides less satisfactory enantioselectivities for linear alkyl aldehydes.

Herein we report the first aldol reaction of Schiff bases of glycine derivatives by Brønsted base (BB) catalysis, which provides *syn* β-hydroxy α-amino acids in high diastereo- and enantioselectivity. The key for this development is the use of a glycine *o*-nitro anilide derivative in combination with an ureidopeptide-based BB catalyst.

Benzophenone imines of glycine esters have shown to be very efficient substrates with applications in many transformations.^3,9^ However, their use in enantioselective synthesis has been mainly limited to metal^10^ and phase transfer^11^ catalysis, while their development in organocatalysis^12^ remains essentially underexplored.^13^ The main reason that can account for this deficiency is the relatively low acidity of the methylenic carbon, which precludes enolate generation through deprotonation by the weak BB catalysts^14^ usually employed. Only recently, three examples have been documented (Figure 1a) in which this problem has been solved by using more acidic structural analogues as fluorenone imine^15^ (**A**), 2-hydroxybenzophenone imine^16^ (**B**), and (*R*)-3-hydroxy-[1,1′-binaphthalene]-2-carbaldehyde^17^ imine (**C**) of glycine derivatives. The increased acidity of these iminoesters is the result of structural modifications on the imine function by the incorporation of motifs that promote either stabilization of the corresponding conjugate base by extensive charge delocalization (**A**) or intramolecular hydrogen bonding as in **B** and **C**. It was our consideration that the installation of an *o*-nitroaniline motif in the carboxy terminus might be another possibility to increase α-carbon acidity of glycine Schiff bases (Figure 1b). It has been reported that *o*-nitroanilides of simple carboxylic acids exhibit intramolecular hydrogen bonding between the oxygen of the nitro group and the hydrogen of the amide moiety,^18^ largely facilitating hydrolysis by enzymes.^19^ We expected that Schiff base **1**, besides this H-bond pattern, should exhibit an additional H-bonding with the imine nitrogen increasing the acidity of the methylenic carbon. If this were the case, enolization of **1** by a weak tertiary amine base should be feasible while generating an *E*-enolate ion pair, mainly.

**Figure 1:**
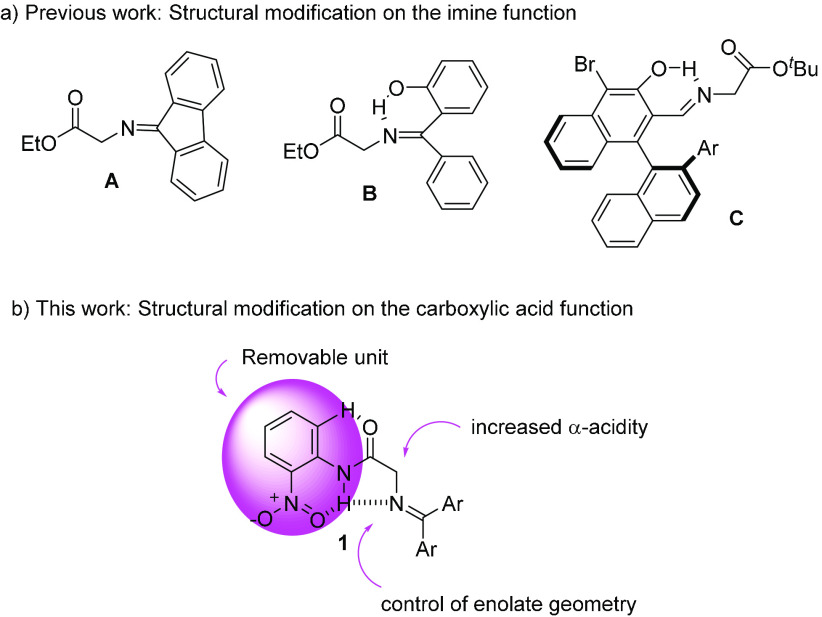
(a) Previously developed Schiff bases of glycine for Brønsted base (BB) catalysis. (b) Schiff base of glycine *o*-nitroanilide proposed as a pronucleophile for BB catalysis.

The first promising piece of evidence supporting this assumption was provided by a single-crystal X-ray analysis of compound **1**, which revealed hydrogen bond lengths of 1.987 Å and 2.149 Å that fit well with the proposed bifurcated hydrogen bond motif^20^ (Figure 2). Interestingly, the X-ray analysis also showed an additional hydrogen bonding (2.234 Å) between the *o*-aromatic hydrogen and the carbonyl oxygen. Therefore, while amides are known to be reluctant to enolization,^21^ we expected these structural features should render substrate **1** quite promising for Brønsted base-promoted stereoselective transformations.

**Figure 2:**
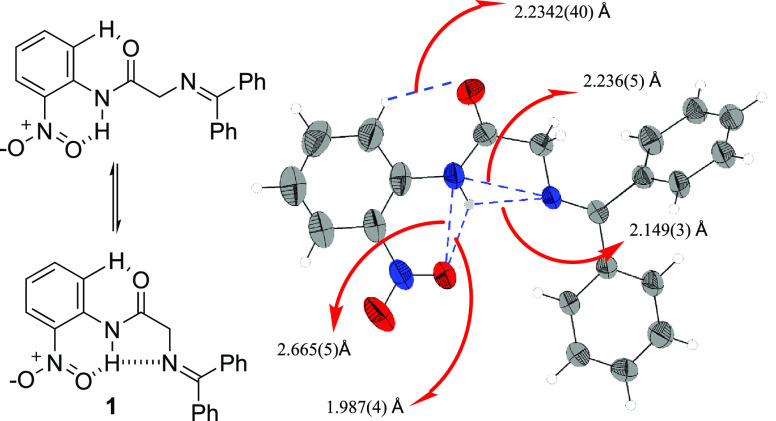
Representative H-bonding interactions of **1** in the solid state. View of the molecular structure of **1** with 50% probability displacement ellipsoids.

Initially, our approach was evaluated from the reaction of benzophenone imine **1**(22) with hydrocinnamaldehyde **4a** (Scheme 1) mediated by squaramides **C1** and **C2**.^23^ Using these bases, the reaction indeed proceeded to give the aldol product **5a** after one-pot reductive workup, but with very poor diastereoselectivity and negligible enantioselectivity for the major *syn* isomer, albeit good for the minor isomer (Table 1, entries 1 and 2). Using the parent ureas, **C3** and **C4**, much better diastereocontrol was achieved (entries 3 and 4), but the enantioselectivity of product **5a** was still poor. To improve stereocontrol through the incorporation of additional H-bond donors^24^ during the reaction, we focused on ureidopeptide-derived Brønsted bases previously developed by us.^25^ It was gratifying to observe that the new variants **C5**, **C6**, and **C7** provided **5a** with diastereomeric ratios greater than 98:2 and in each case with good enantioselectivity (entries 5, 6, and 8). Further improvement was achieved using catalyst **C6** and carrying out the reaction at 0 °C, and **5a** was obtained in 77% isolated yield and 94% *ee* (entry 7). Although in these reactions, the formation of small amounts (10–15%) of the cycled product **8a** was also observed,^5c^ its reductive workup also furnished the amino alcohol derivative **5a**.^22^ Likewise, iminoamides **2** and **3** upon treatment with **4a** in CH_2_Cl_2_ at 0 °C led to products **6a** and **7a** in less than 24 h with good isolated yields and stereoselectivities, Table 1. The best result was attained for the latter, which was obtained essentially as a single *syn*-diastereomer and with excellent enantioselectivity.^26,27^

**Scheme 1:**
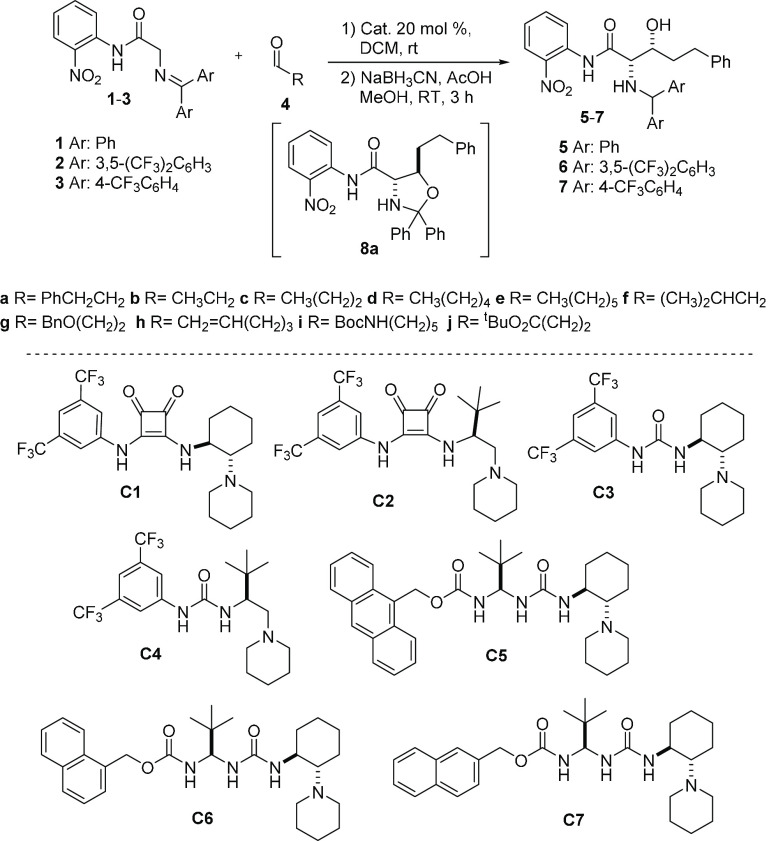
Aldol Reaction between Nitroanilides **1**–**3** and Hydrocinnamaldehyde **4a** Promoted by Brønsted Base Catalysts

**Table 1:** Catalyst Screening for the Reaction of **1**–**3** with Hydrocinnamaldehyde **4a** to Afford **5a**–**7a**^a^

entry	cat.	*t* (h)	*T* (°C)	conv (%)^b^	yield (%)^c^	dr^d^	ee^e^
1	**C1**	48	rt	92	73	75:25	34(83)
2	**C2**	48	rt	90	70	75:25	38(93)
3	**C3**	48	rt	69	57	98:2	40
4	**C4**	16	rt	82	73	95:5	36
5	**C5**	48	rt	87	75	>98:2	85
6	**C6**	16	rt	95	70	>98:2	88
7	**C6**	64	0	99	77	>98:2	94
8	**C7**	48	rt	78	58	>98:2	78

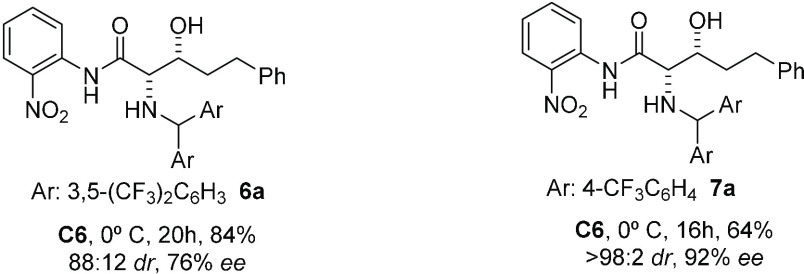

a Reactions conducted on a 0.2 mmol scale in 0.4 mL of CH_2_Cl_2_ (mol ratio *N*-(diarylmethylene)glycine *o*-nitroanilide/aldehyde/catalyst 1:3:0.2).

b Determined by the disappearance of the starting **1**.

c Isolated yield of **5a** and the corresponding minor isomer.

d Determined by ^1^H NMR (300 MHz) analysis on the crude product before isolation by column chromatography.

e Determined by chiral HPLC.

The scope of the new glycine amide reagent **3** to the synthesis of *syn*-β-hydroxy α-amino acids was next examined from a selection of representative enolizable aldehydes (Table 2). With the exception of α-branched aldehydes such as isobutyraldehyde and cyclohexanecarboxaldehyde, which were inert to this system, results were consistently good. As the data in Table 2 show, *syn*-β-hydroxy α-amino acids bearing short and long linear chains, which cannot be accessed by the above known methods, *vide supra*, may be prepared with very good yields and *ee* values. Isovaleraldehyde and aldehydes bearing side chains with functional groups (e.g., ester, carbamate, and ether) are equally tolerated to give the respective *syn*-isomer with good enantiomeric excess. Importantly, in every case under these reaction conditions, self-aldol products from the corresponding enolizable aldehyde **4** were not detected.^28^ Likewise, product **7a** was chemically and stereochemically unchanged when exposed to treatment with aldehyde **4b** at room temperature in the presence of both Et_3_N (20 mol %) and Schreiber achiral diarylthiourea^23^ (20 mol %) for 24 h, thus indicating product stability.

**Table 2:**
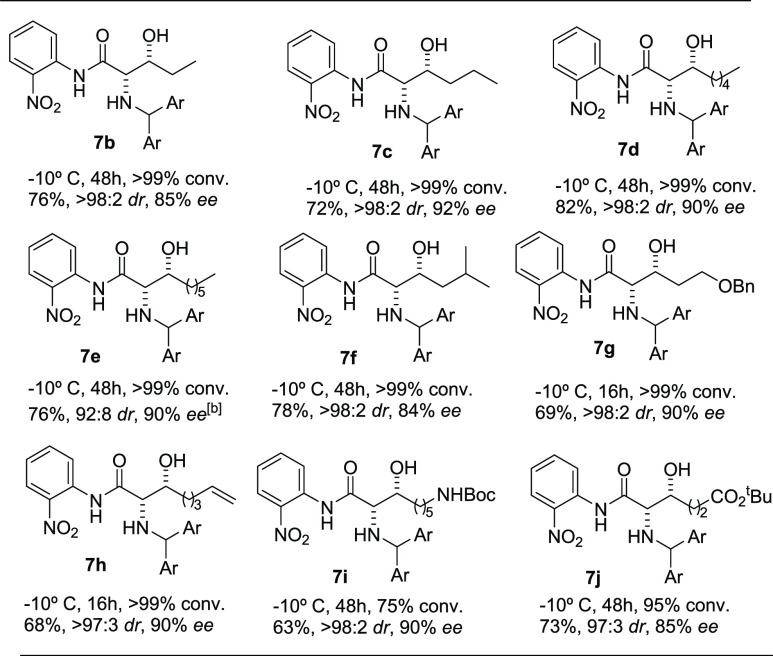
Scope of the Aldol Reaction of **3** with Aldehydes **4** Assisted by **C6**^a^

a Reactions conducted on a 0.2 mmol scale in 0.4 mL of CH_2_Cl_2_ (mol ratio *N*-(diarylmethylene)glycine *o*-nitroanilide **3**/aldehyde/catalyst 1:3:0.2). Conversion determined by the disappearance of the starting *N*-(diarylmethylene)glycine *o*-nitroanilide. Yield of the isolated major isomer. Diastereomeric ratio determined by ^1^H NMR (300 MHz) analysis on the crude product. Enantiomeric excess determined by chiral HPLC. Ar: 4-CF_3_C_6_H_4_.

b Yield of the two isomers.

The absolute configuration of the adducts was established by X-ray analysis of compound **10** derived from the reaction of **3** with hydrocinnamaldehyde, Scheme 2, and by assuming a uniform reaction mechanism.^29^ On the other hand, treatment of aldol product **7a** with 2 equiv of (Boc)_2_O led to **11**, which upon Evans hydrolytic conditions provided the carboxylic acid **12** along with *N*-Boc nitroaniline.^30^

**Scheme 2:**
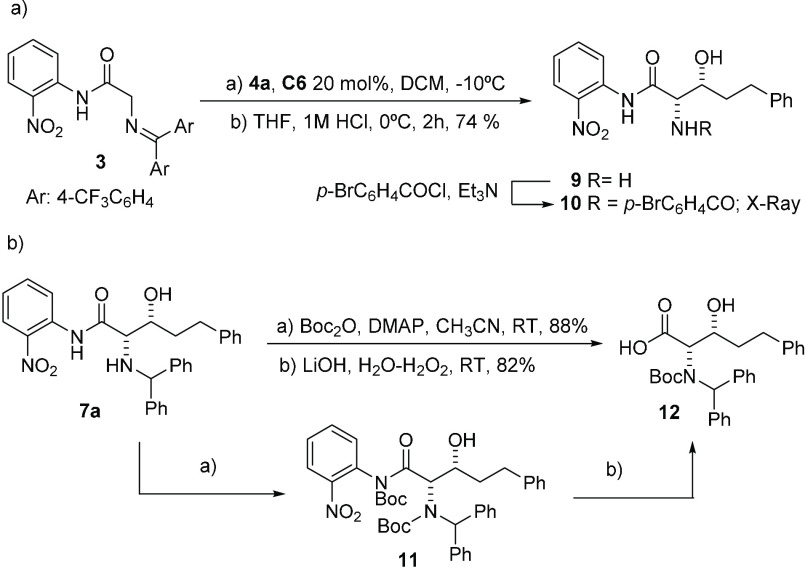
(a) One-Pot Reaction/Hydrolytic Work-up and Acylation Reaction. (b) Anilide Cleavage from the Adducts

The above experimental results clearly show that benzophenone imines of glycine *o*-nitroanilides are acidic enough to react in BB-catalyzed reactions under soft enolization conditions. To further prove the significance of the intramolecular hydrogen bonding in *o*-nitroanilides **1**–**3**,^31^ benzophenone imines **13**, **14**, and **15** were prepared and subjected to treatment with **4a** under the above conditions (Scheme 3), and in no case was a reaction observed. Likewise, in an attempt to strengthen the hydrogen bonding, compound **16** with an additional nitro group in the *para* position of the aromatic ring was also prepared. While, in this case, the reaction proceeded, product **17** was obtained in a modest yield and in an almost racemic form.

**Scheme 3:**
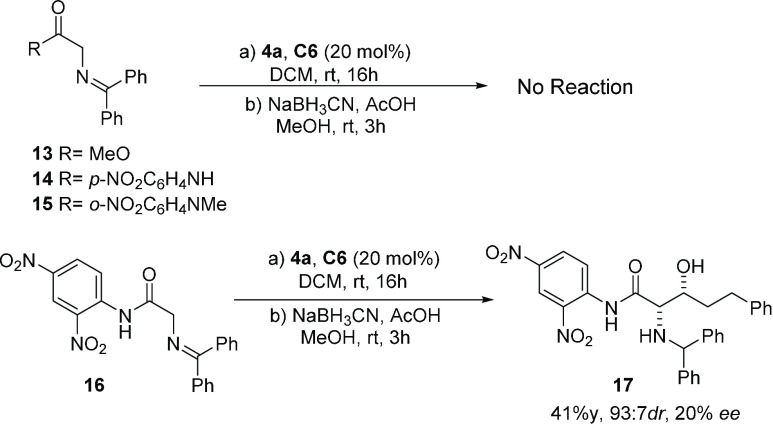
Reactivity of Benzophenone Imines **13**–**16** in the Aldol Addition

In order to understand the origin of the *syn* selectivity in the reaction, we performed an initial evaluation of the relative stabilities of the naked enolates derived from ketimine **3**,^32^ and, as expected, it was found that the naked *E*-enolate was more stable than the respective *Z*-enolate, Δ*G*_*E/Z*_*=* +3.4,^33^ (Figure 3a). Our interpretation of the higher stability of the *E* enolate relies on three main pieces of evidence. First, the Pauli repulsion of the negative charge of the amide oxygen atom and the lone pair of the imine nitrogen present in the *Z*-enolate. Second, the existence of an additional H-bonding interaction between the lone pair of the iminic nitrogen and the NH moiety only present in the *E*-enolate. Lastly, the planarity observed in the *E*-enolate (dihedral θ_1234_ close to 180°) that points to the existence of extended π-conjugation that is not present in the *Z* counterpart (θ_1234_ far from 180° or 0°). Remarkably, the internal NH···O_2_N and CH···O^–^ hydrogen–bonding interactions shown in the X-ray analysis are also detected in this study. Assuming that the ketimine *E*-enolate (E**-Ar**) participates during the reaction, a plausible explanation of the observed *syn*-selectivity can be provided by the model shown in Figure 3b, wherein the *si* face of the *E*-enolate approaches the *si* face of the aldehyde.

**Figure 3:**
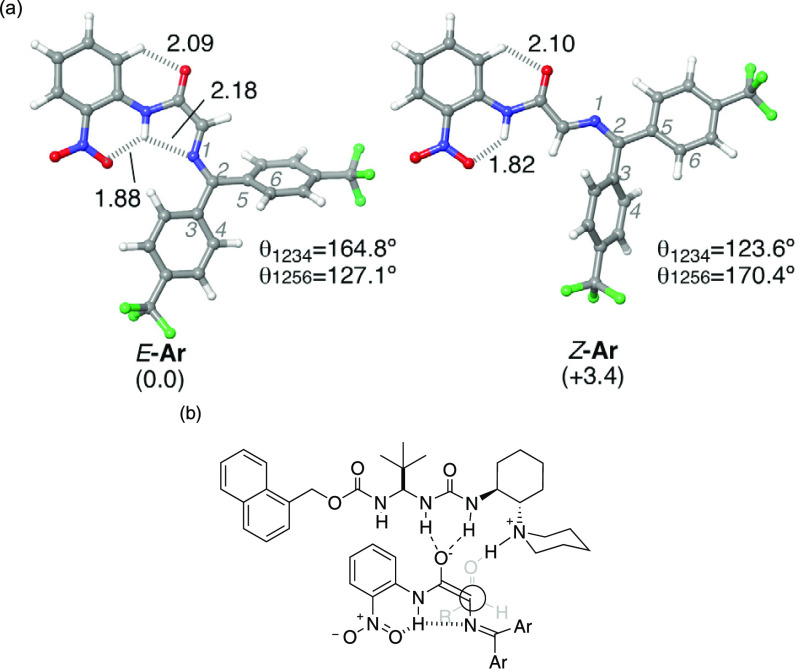
(a) Computed naked *E*- and Z-enolates of ketimine **3**. Relative Gibbs free energy values in kcal mol^–1^ calculated at B3LYP-D3(PCM)/6-311+G(d,p)//B3LYP-D3(PCM)/6-31G(d) at 298 K. Distances are in Å. (b) Plausible model accounting for the *syn*-selectivity observed from the reaction with ketimine-glycine amides as pronucleophiles.

While these results confirm the initial assumption concerning the role of the 2-nitroanilide tether, further evidence is provided from the reactions in Scheme 4. For instance, treatment of the pyridylacetic acid-derived 2-nitroanilide **18** with 20 mol % of TEA and hydrocinnamaldehyde for 48 h at rt, followed by *in situ* reaction with Ac_2_O and pyridine, provided the corresponding aldols **20** with >99% conversion (67% combined isolated yield) and as a 50:50 diastereomeric mixture. However, when the same reaction conditions were used with the 4-nitroanilide **19** and the anilide derivative **20**, compounds **23** and **24** were produced in lower conversions (50% and 33% respectively). Similarly, the essential absence of reactivity of **21,** which bears the 2-nitroanilide motif but lacks the heteroatom at the aromatic ring, for producing compound **25**, provides further proof of the significance of the whole H-bonding network in the starting pronucleophile for reactivity.

**Scheme 4:**
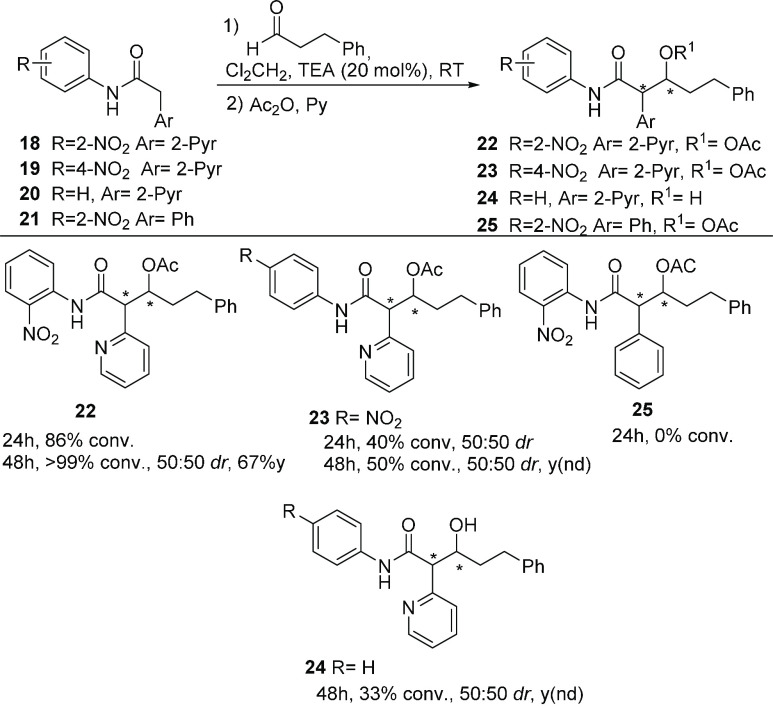
Aldol Reaction of α-Pyridyl and Phenyl Acetanilides

In summary, an effective organocatalytic direct access to *syn*-β-hydroxy α-amino acids is reported. The strategy is based on Schiff bases of glycine *o*-nitroanilide, wherein the *o*-nitroanilide motif is key for enolate generation by a soft Brønsted base allowing direct reaction with aldehydes under efficient diastereo- and enantiocontrol. Further applications of both the nitroanilide tether and the catalyst and/or variants may be easily anticipated.

## Experimental Section

### General Information

All nonaqueous reactions were performed under an inert atmosphere using oven-dried glassware, and the mixtures were magnetically stirred. Yields refer to chromatographically purified and spectroscopically pure compounds, unless otherwise stated. Heat requiring reactions were performed using a hot plate with a sand or an oil bath and a condenser. Reactions requiring low temperatures were performed using cooling bath circulators, Huber T100E, and acetone or isopropanol baths. Organic layers washed with aqueous phases were dried over MgSO_4_ or Na_2_SO_4_ and filtered through cotton. Organic solvents were evaporated under reduced pressure using rotavapors Büchi R-100, R-200, and R-210, the latter equipped with a Büchi V-700 vacuum pump and a Büchi V-850 vacuum controller, appropriate for the evaporation of solvents when products were volatile compounds. For the complete removal of solvents, a vacuum pump, Telstar Top-3 (≈ 0.5 mmHg), was employed. Reagents were purchased from different commercial suppliers (Aldrich, Across, Alfa Aesar, Fluka, TCI, Merck, Fluorochem, etc.), stored as specified by the manufacturer, and used without previous purification unless otherwise stated. Triethylamine was purified by distillation. When anhydrous solvents were required, they were dried following established procedures.^34^ Dichloromethane was dried over CaH_2_, and tetrahydrofuran was dried by filtration through activated alumina (powder 150 mesh, pore size 58 Å, basic Sigma-Aldrich) columns. Reactions and flash chromatographic columns were monitored by thin-layer chromatography (TLC) using Merck silica gel 60 F254 plates and visualized by fluorescence quenching under UV light, Fisher Biolock lamp VL-4LC, λ = 254 and 365 nm. In addition, TLC plates were stained with a dipping solution of potassium permanganate (1g) in 100 mL of water (limited lifetime), followed by heating and charring with 1% w/w ninhydrin in ethanol followed by heating. Chromatographic purification was performed on Merck ROCC 60 silica gel 40–63 μm as stationary phase and a suitable mixture of solvents (typically hexane: ethyl acetate or dichloromethane/methanol) as eluent. Optical rotations were recorded using a Jasco P-2000 polarimeter; specific rotations (SR) ([α]^D^) are reported in 10^–1^ deg cm^2^ g^–1^; concentrations (c) are quoted in g/100 mL; D refers to the D line of sodium (589 nm); temperatures (T) are given in degree Celsius (°C). Melting points were determined in open capillaries in a Stuart SHP3 melting point apparatus and were uncorrected. NMR spectra were recorded using a Bruker Avance 300 (300 MHz for ^1^H, 75 MHz for ^13^C) spectrometer, Bruker 400 spectrometer (400 MHz for ^1^H, 100 MHz for ^13^C), or Bruker AV-500 spectrometer (500 MHz for ^1^H, 125 MHz for ^13^C). Chemical shifts (δ) are quoted in parts per million referenced to the residual solvent peak, usually CDCl_3_, ^1^H (δ = 7.26) and ^13^C (δ = 77.0). The multiplicity of each signal is designated using the following abbreviations: s, singlet; d, doublet; t, triplet; q, quartet; m, multiplet; brs, broad singlet. Coupling constants (*J*) are reported in hertz (Hz). The MestrReNova Mnova 8.1 program was used to process and edit the registered spectra. Mass spectra were recorded on an ESI-ion trap mass spectrometer (Agilent 1100 series LC/MSD, SL model) on a UPLC-DAD-QTOF, ultra high-performance liquid chromatography–mass spectrometer, Waters UPLC ACQUITY, Waters PDA detector, Waters Sunapt G2, or on an Agilent Thermoquest LCT spectrometer. Mass spectrometry analyses were performed in the General Research Service (SGIker) of the University of the Basque Country (UPV/EHU). Enantiomeric excesses were determined using analytical high-performance liquid chromatography (HPLC) performed on Waters 600-E (equipped with 2996 and 2998 photodiode array UV detector) employing Daicel columns (IA, IF) and Phenomenex Lux (cellulose 3 μm, amylose 3 μm). The X-ray diffraction analysis experiments were conducted in the General Research Service (SGIker) of the University of the Basque Country (UPV/EHU) using difractometers for monocrystals. Aliphatic aldehydes **4a**, **4b**, **4c**, **4d**, **4e**, and **4f** are commercially available and were purchased from commercial suppliers. Aldehydes **4g**, **4h**, **4i**, and **4j** were prepared following literature procedures.^35^ All aldehydes were dissolved in Cl_2_CH, treated with an aqueous saturated solution of NaHCO_3_, and subsequently distilled before their use in the aldol reaction.

### Synthesis of Catalysts

Bifunctional organocatalysts **C1**,^36^**C2**,^37^**C3**,^38^ and **C4**(39) were prepared following reported procedures. Catalysts **C5**, **C6**, and **C7** were synthesized following a modification of the procedures previously reported.^25a^

### Synthesis of Catalysts **C5**, **C6**, and **C7**

#### *a) Preparation* of *N*-Protected *l*-*tert*-*Leucine*.^40^

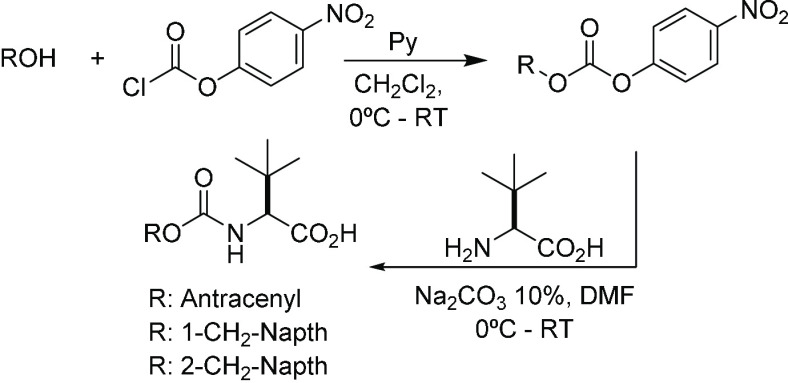



In the first step, pyridine (0.9 mL, 11 mmol, 1.1 equiv) was added to a stirred solution of *p*-nitrophenyl chloroformate (2.2 g, 11 mmol, 1.1 equiv) in dichloromethane (13.6 mL). The white slurry was cooled to 0 °C, and the corresponding alcohol (10 mmol, 1 equiv) was slowly added in at the same temperature. After addition, the mixture was allowed to warm to room temperature and stirred for 16 h. The reaction mixture was diluted with CH_2_Cl_2_ (40 mL) and washed with 1 M HCl (20 mL), water (20 mL), and brine (20 mL). The organic layer was dried over MgSO_4_ and concentrated under reduced pressure. The residue was used in the next step without further purification.

In the second step, to a stirred solution of l-*tert*-leucine (1.31 g, 10 mmol, 1 equiv) in 10% Na_2_CO_3_ (26 mL) and dimethylformamide (10 mL) was added a solution of the corresponding carbonate (10 mmol, 1 equiv) in dimethylformamide (30 mL) slowly at 0 °C. The mixture was stirred at the same temperature for 1 h and at room temperature for 16 h. The reaction mixture was poured into H_2_O (100 mL) and washed with Et_2_O (3 × 50 mL). The aqueous layer was cooled in an ice bath and acidified with concentrated HCl, followed by extraction with EtOAc (3 × 50 mL). The combined organic phases were washed with brine (5 × 50 mL), dried over MgSO_4_, and concentrated under reduced pressure.

##### (*S*)-2-(((Anthracen-9-ylmethoxy)carbonyl)amino)-3,3-dimethylbutanoic Acid.^25a^

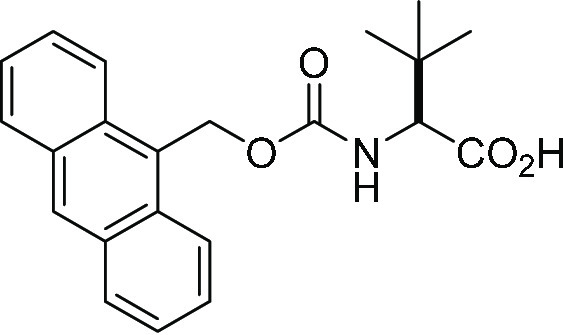



The title compound was prepared from 9-anthracenemethanol (2.08 g, 10 mmol) following the general procedure. Purification by flash column chromatography on silica gel (hexane/EtOAc, 70:30) afforded the title compound as a white solid. Yield: 88% (3.2 g, 8.8 mmol). All of the spectroscopic data were coincident with those previously reported. ^1^H NMR (300 MHz, CDCl_3_) δ: 8.52 (s, 1H), 8.38 (d, *J* = 8.9 Hz, 2H), 8.04 (d, *J* = 8.7 Hz, 2H), 7.65–7.54 (m, 2H), 7.53–7.46 (m, 2H), 6.18 (q, *J* = 12.1 Hz, 2H), 5.24 (d, *J* = 10.4 Hz, 1H), 4.28 (d, *J* = 10.2 Hz, 1H), 1.01 (s, 9H).

##### (*S*)-3,3-Dimethyl-2-(((naphthalen-1-ylmethoxy)carbonyl)amino)butanoic Acid

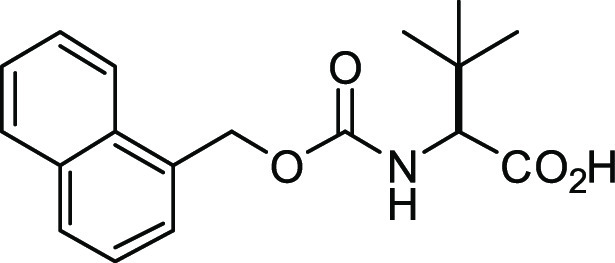



The title compound was prepared from 1-naphthalenemethanol (1.58 g, 10 mmol) following the General Procedure. Purification by flash column chromatography on silica gel (hexane/EtOAc, 80:20) afforded the title compound as a white solid. Yield: 88% (2.8 g, 8.8 mmol). Mp: 131–135 °C. ^1^H NMR (300 MHz, CDCl_3_): δ 10.10 (s,1H), 8.04 (d, *J* = 8.0 Hz, 1H), 7.87 (t, *J* = 8.8 Hz, 2H), 7.49 (dt, *J* = 27.2, 7.3 Hz, 4H), 5.60 (q, *J* = 12.3 Hz, 2H), 5.40 (d, *J* = 9.5 Hz, 1H), 4.26 (d, *J* = 9.6 Hz, 1H), 1.02 (s, 9H). ^13^C{^1^H} NMR (75 MHz, CDCl_3_): δ 176.6, 156.4, 133.8, 131.7, 129.5, 128.8, 127.6, 126.7, 126.07, 125.4, 123.7, 65.6, 62.3, 34.7, 26.6. UPLC-DAD-QTOF, HRMS (ESI) *m*/*z*: [M + Na]^+^ calcd for C_18_H_21_NO_4_Na, 338.1368; found, 338.1369.

##### (*S*)-3,3-Dimethyl-2-(((naphthalen-2-ylmethoxy)carbonyl)amino)butanoic Acid.^25a^

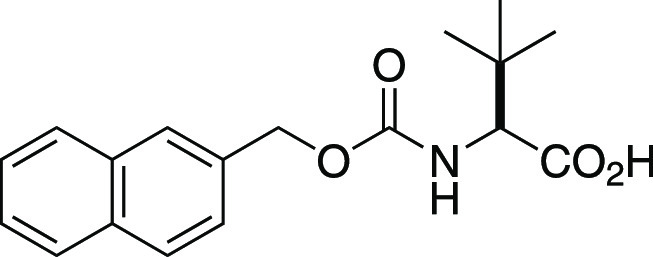



The title compound was prepared from 2-naphthalenemethanol (1.58 g, 10 mmol) following the general procedure. Removal of the remaining phenol was not possible by column chromatography. Therefore, after the work-up described in the general procedure, the crude was dissolved in Et_2_O (30 mL) and basified with NaHCO_3_ saturated aq. solution. The aqueous phase was washed with Et_2_O (3 × 20 mL), acidified with concentrated HCl, and extracted with EtOAc (3 × 25 mL). The combined organic phases were dried over MgSO_4_ and evaporated under reduced pressure to afford the title compound as a white solid. Yield 48% (1.5 g, 4.8 mmol). All of the spectroscopic data were coincident with those previously reported. ^1^H NMR (300 MHz, CDCl_3_): δ 7.92–7.74 (m, 4H), 7.58–7.36 (m, 3H), 5.47 (d, *J* = 9.4 Hz, 1H), 5.30 (s, 2H), 4.26 (d, *J* = 9.6 Hz, 1H), 1.04 (s, 9H).

#### *b) General Procedure for Isocyanate Synthesis and Coupling with the Amine*

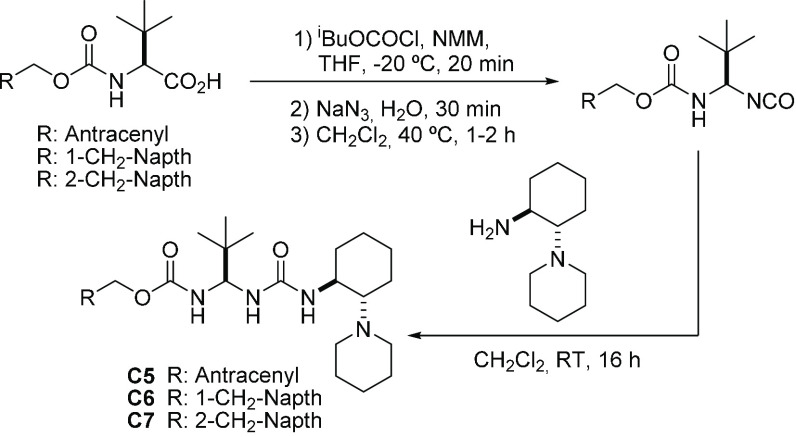



To a cooled solution of the corresponding *N*-protected α-amino acid (5 mmol, 1 equiv) in dry THF (20 mL) were added isobutyl chloroformate (0.65 mL, 5 mmol, 1 equiv) and *N*-methylmorpholine (0.6 mL, 5 mmol, 1 equiv) at −20 °C. The mixture was stirred at the same temperature for 20 min. Then, a suspension of NaN_3_ (0.48 g, 7.5 mmol, 1.5 equiv) in 5 mL of H_2_O was added, and the reaction mixture was stirred at the same temperature for 30 min. The organic layer was separated and evaporated, and the residue was dissolved in CH_2_Cl_2_ (30 mL) and washed with water (15 mL). The organic phase was dried over MgSO_4_, filtered, and concentrated *in vacuo* to give a yellow oil, which was dissolved in dry CH_2_Cl_2_ (10 mL). The resulting solution was stirred at 40 °C under nitrogen for 1–2 h. The reaction was monitored by IR analysis until the disappearance of the azide band (from azide ≈2136 cm^–1^ to isocyanate ≈2239 cm^–1^). After isocyanate generation, (1*S*,2*S*)-2-(piperidin-1-yl)cyclohexan-1-amine^41^ was added (638 mg, 3.5 mmol, 0.7 equiv), and the reaction mixture was stirred for 16 h at room temperature. The solvent was evaporated under reduced pressure, and the residue was purified by flash column chromatography on silica gel to afford the desired catalysts.

##### Anthracen-9-yl-methyl((*S*)-2,2-dimethyl-1-(3-((1*S*,2*S*)-2-(piperidin-1-yl)cyclohexyl)ureido) propyl)carbamate **C5**

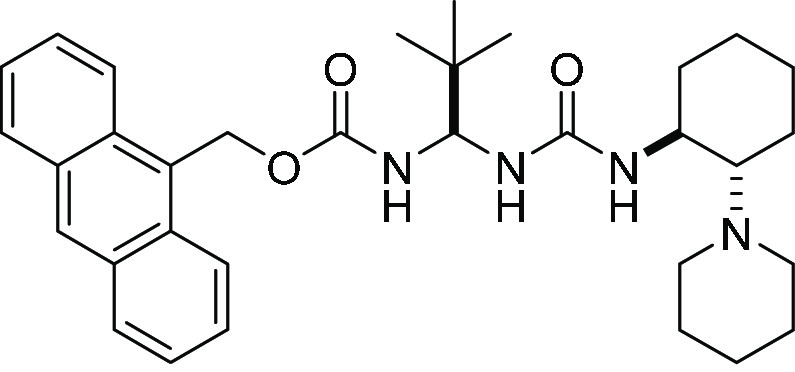



The title compound was prepared from (*S*)-2-(((anthracen-9-ylmethoxy) carbonyl)amino)-3,3-dimethyl butanoic acid (1.8 g, 5.0 mmol, 1.0 equiv) and (1*S*,2*S*)-2-(piperidin-1-yl)cyclohexan-1-amine (316 mg, 1.4 mmol, 0.7 equiv) according to the general procedure. The catalyst was isolated by flash column chromatography on silica gel (Hex/EtOAc 80:20) as a white solid. Yield: 63% (1.7 g, 3.1 mmol). Mp:146–148 °C. [α]_D_^25^ +65.5 (*c* 1, CH_2_Cl_2_). ^1^H NMR (500 MHz, DMSO-*d*_6_, 70 °C): δ 8.67 (s, 1H), 8.37 (d, *J* = 8.7 Hz, 2H), 8.12 (d, *J* = 8.0 Hz, 2H), 7.62–7.57 (m, 2H), 7.58–7.49 (m, 2H), 7.21 (d, *J* = 9.1 Hz, 1H), 6.11–6.00 (m, 2H), 5.79 (s, 1H), 5.17 (s, 1H), 2.55–2.50 (m, 1H), 2.33–2.22 (m, 1H), 2.02–1.94 (m, 1H), 1.75 (d, *J* = 8.2 Hz, 1H), 1.70–1.60 (m, 2H), 1.58–1.47 (m, 1H), 1.38 (s, 4H), 1.25 (s, 2H), 1.16–1.08 (m, 3H), 0.82 (s, 9H). ^13^C{^1^H} NMR (126 MHz, DMSO-*d*_6_): δ 157.3, 155.7, 131.0, 130.5, 128.9, 128.5, 127.5, 126.5, 125.2, 124.1, 65.0, 59.5, 57.7, 53.9, 53.4, 36.1, 34.5, 26.3, 25.4, 25.1. UPLC-DAD-QTOF, HRMS (ESI) *m*/*z*: [M + H]^+^ calcd for C_33_H_45_N_4_O_3_, 545.3492; found, 545.3506.

##### Naphthalen-1-yl-methyl((1*S*)-2,2-dimethyl-1-(3-((2*S*)-2-(piperidin-1-yl)cyclohexyl) ureido)propyl)carbamate **C6**


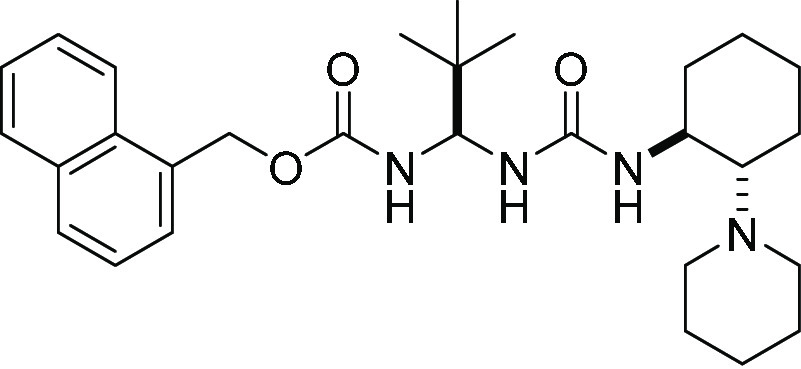


The compound was prepared according to the general procedure starting from (*S*)-3,3-dimethyl-2-(((naphthalen-1-ylmethoxy) carbonyl)amino)butanoic acid (1.57 g, 5 mmol) and was isolated by flash column chromatography on silica gel (hexane/EtOAc, 90:10) as a white solid. Yield: 60% (1.42 g, 3 mmol). Mp: 174–179 °C. [α]_D_^23^ −19.1 (*c* 0.5, CH_2_Cl_2_). ^1^H NMR (500 MHz, DMSO-*d*_6_, 70 °C): δ 8.32–8.27 (m, 1H), 8.21–8.18 (m, 1H), 8.14 (d, *J* = 8.2 Hz, 1H), 7.85–7.77 (m, 3H), 7.76–7.69 (m, 1H), 7.19 (br s, 1H), 6.16 (d, *J* = 9.1 Hz, 1H), 6.00–5.95 (m, 1H), 5.75 (q, 2H), 5.39 (t, *J* = 9.2 Hz, 1H), 3.63 (br s, *J* = 6.7, 5.6 Hz, 1H), 2.85 (br s, 2H), 2.60 (br s, 2H), 2.43 (s, 1H), 2.28 (d, *J* = 12.5 Hz, 1H), 2.04 (d, *J* = 11.0 Hz, 1H), 1.94 (d, *J* = 11.4 Hz, 1H), 1.82 (d, *J* = 10.1 Hz, 1H), 1.74–1.66 (m, 5H), 1.64–1.53 (m, 3H), 1.47–1.36 (m, 3H), 1.12 (s, 9H). ^13^C{^1^H} NMR (75 MHz, DMSO-*d*_6_): δ 157.3, 155.5, 133.2, 132.7, 131.0, 128.5, 126.6, 126.4, 125.9, 125.3, 123.6, 65.2, 63.4, 49.5, 49.3, 35.8, 33.6, 25.5, 24.5, 23.8. UPLC-DAD-QTOF, HRMS (ESI) *m*/*z*: [M + H]^+^ calcd for C_29_H_43_N_4_O_3_, 495.3335; found, 495.3349.

##### Naphthalen-2-yl-methyl((1*S*)-2,2-dimethyl-1-(3-((2*S*)-2-(piperidin-1-yl)cyclohexyl) ureido)propyl)carbamate **C7**

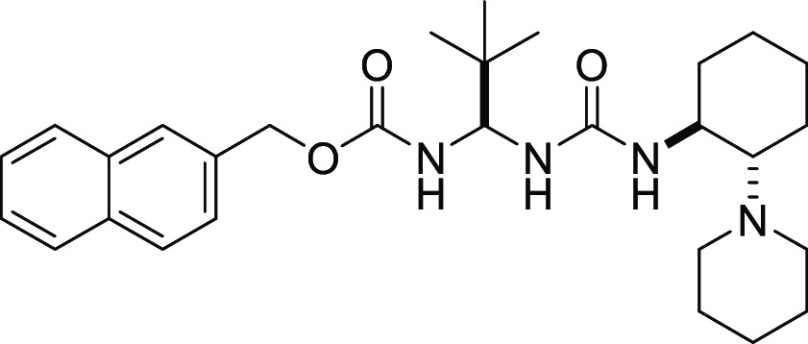



The compound was prepared according to the general procedure starting from ((*S*)-3,3 dimethyl-2-(((naphthalen-2-ylmethoxy)carbonyl)amino)butanoic acid (1.57 g, 5 mmol) and was isolated by flash column chromatography on silica gel (hexane/EtOAc, 70:30) as a white solid. Yield: 61% (1.06 g, 2.14 mmol). Mp: 170–172 °C. [α]_D_^23^ −15.6 (*c* 1, CH_2_Cl_2_). ^1^H NMR (500 MHz, DMSO-*d*_6_, 70 °C): δ 7.99–7.80 (m, 4H), 7.65–7.41 (m, 3H), 6.88 (s, 1H), 5.91 (d, *J* = 9.2 Hz, 1H), 5.69 (d, *J* = 6.3 Hz, 1H), 5.21 (s, 2H), 5.15 (t, *J* = 9.2 Hz, 1H), 3.48–3.31 (m, 1H), 2.60–2.55 (m, 2H), 2.39–2.27 (m, 2H), 2.24–2.11 (m, 1H), 2.09–2.03 (m, 1H), 1.82–1.74 (m, 1H), 1.72–1.64 (m, 1H), 1.64–1.52 (m, 1H), 1.52–1.39 (m, 4H), 1.39–1.25 (m, 3H), 1.24–1.12 (m, 3H), 0.91 (s, 9H). ^13^C{^1^H} NMR (126 MHz, DMSO-*d*_6_): δ 157.2, 155.5, 134.9, 132.7, 132.5, 127.9, 127.7, 127.6, 126.3, 126.2, 126.1, 125.7, 67.5, 65.2, 49.7, 49.0, 35.9, 33.8, 25.9, 25.5, 25.0, 24.6, 23.6. UPLC-DAD-QTOF, HRMS (ESI) *m*/*z*: [M + H]^+^ calcd for C_29_H_43_N_4_O_3_, 495.3335; found, 495.3348.

### Preparation of Ketimine Hydrochlorides

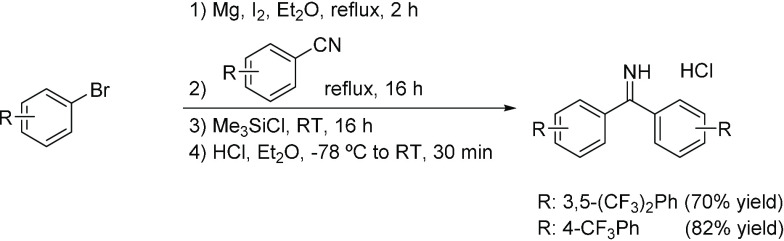



The imine hydrochlorides employed for the study were prepared by adapting literature procedures.^42^ Dry diethyl ether (50 mL) was added to a three-necked round-bottom flask equipped with a reflux condenser containing magnesium powder (434 mg, 20 mmol, 1 equiv) and iodine (20 mg). The resulting suspension was heated to mild reflux, and the corresponding bromobenzene was added dropwise (20 mmol, 1 equiv). The resulting mixture was stirred at the same temperature for 2 h, resulting in the dissolution of the magnesium and the darkening of the solution. Then, the corresponding benzonitrile (20 mmol, 1 equiv) was added dropwise to the solution, and the mixture was allowed to stir at the same temperature for 16 h, resulting in the formation of a white salt. Thus, Me_3_SiCl (2.5 mL, 20 mmol, 1 equiv) was added dropwise with vigorous stirring after removing the heating, and the resulting mixture was stirred at room temperature for 16 h. A brown solid formed as a result, and the mixture was concentrated under reduced pressure and dissolved in benzene in order to filter off the salts. Benzene was then removed under reduced pressure, the resulting crude was dissolved in dry diethyl ether (10 mL), and the mixture was cooled to −78 °C. Then, HCl (2 M in Et_2_O, 10 mL, 20 mmol, 1 equiv) was added, the resulting suspension was allowed to warm to room temperature over 30 min, and the solid was filtered, washed with diethyl ether, and dried under an IR lamp in order to afford the desired product.

### Preparation of Ketimines of Glycine Nitroanilides

#### 2-((Diphenylmethylene)amino)-*N*-(2-nitrophenyl)acetamide **1**

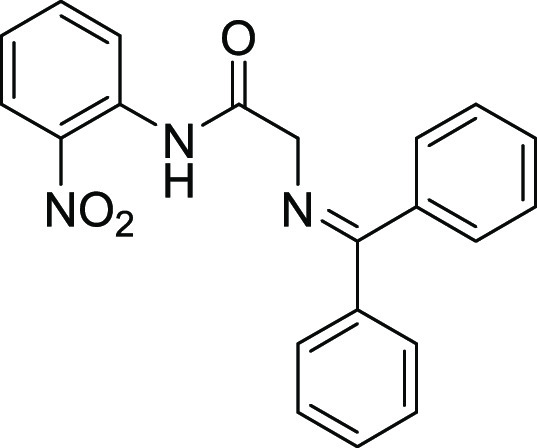



**N*-Boc glycine *o*-nitroanilide*:^43^ Boc-Gly-OH (1.38 g, 10 mmol, 1 equiv) and *o*-nitroaniline (1.38 g, 10 mmol, 1 equiv) were dissolved in dry pyridine (30 mL). The solution was cooled to −15 °C, and phosphorus oxychloride (1 mL, 11 mmol, 1.1 equiv) was added dropwise with vigorous stirring. During addition, the reaction mixture was colored deeply red and slowly changed to brown. The reaction was complete after 30 min (monitored by TLC); afterward, it was quenched with ice–water (100 mL), and the mixture was extracted with EtOAc (4 × 60 mL). The combined organic phases were dried over MgSO_4_, and the solvent was evaporated *in vacuo*. The residue was coevaporated successively with hexane and diethyl ether, and the resulting solid was crushed with diethyl ether and hexane. This afforded a yellow solid. Yield: 68% (2.01 g, 6.8 mmol). Mp: 128–130 °C. ^1^H NMR (300 MHz, CDCl_3_): δ 11.02 (brs, 1H), 8.84 (dd, *J* = 8.6, 1.3 Hz, 1H), 8.25 (dd, *J* = 8.5, 1.6 Hz, 1H), 7.69 (ddd, *J* = 8.7, 7.2, 1.6 Hz, 1H), 7.26–7.10 (m, 1H), 5.19 (brs, 1H), 4.05 (d, *J* = 6.1 Hz, 2H), 1.57 (s, 3H), 1.53 (s, 6H). ^13^C{^1^H} NMR (75 MHz, CDCl_3_): δ 169.6, 136.6, 134.9, 127.0, 126.5, 124.2, 122.7, 81.8, 46.4, 28.9. UPLC-DAD-QTOF, HRMS (ESI) *m*/*z*: [M + H]^+^ calcd for C_13_H_17_N_3_O_5_, 296.1168; found, 296.1274.

**N*-Deprotection*: To a solution of the previous *N*-Boc aminoamide (3 mmol) in CH_2_Cl_2_ (12 mL) was added trifluoroacetic acid (4.5 mL) at 0 °C. The mixture was stirred at room temperature for 30 min until full conversion (monitored by TLC). The solvents were evaporated, and the residue was coevaporated successively with a mixture of diethyl ether and pentane. Then it was dried *in vacuo*, and the resulting solid, obtained in a quantitative yield, was used in the next step without further purification. Mp: 148–152 °C. ^1^H NMR (300 MHz, D_2_O): δ 8.15–7.93 (m, 1H), 7.84–7.59 (m, 2H), 7.41 (ddd, *J* = 8.6, 6.6, 2.3 Hz, 1H), 4.03 (s, 3H). ^13^C NMR (75 MHz, D_2_O): δ 166.4, 135.1, 129.6, 127.1, 126.4, 125.6, 41.2. Measured after neutralization: UPLC-DAD-QTOF, HRMS (ESI) *m*/*z*: [M + H]+ calcd for C_8_H_10_N_3_O_3_, 196.0722; found, 196.0723.

*Iminoamide formation*: To a suspension of the aminoamide trifluororacetate salt obtained in the previous step (927 mg, 3 mmol, 1 equiv) in CH_2_Cl_2_ (11 mL) were added benzophenone imine (3 mmol, 1 equiv) and anhydrous MgSO_4_ (903 mg, 7.5 mmol, 2.5 equiv). The reaction mixture was stirred at room temperature until consumption of the starting material. The mixture was then filtered to remove the salts and evaporated *in vacuo*. The crude was crushed in diethyl ether/hexane to afford a pure yellow solid. Yield: 78% (840 mg, 2.34 mmol). Mp: 111–114 °C. ^1^H NMR (300 MHz, CDCl_3_): δ 12.15 (s, 1H), 8.93 (dd, *J* = 8.6, 1.3 Hz, 1H), 8.29 (dd, *J* = 8.4, 1.6 Hz, 1H), 7.97–7.79 (m, 2H), 7.69 (ddd, *J* = 8.7, 7.4, 1.6 Hz, 1H), 7.64–7.39 (m, 5H), 7.31–7.16 (m, 3H), 4.17 (s, 2H). ^13^C{^1^H} NMR (75 MHz, CDCl_3_): δ 172.3, 171.6, 139.4, 137.4, 136.7, 135.5, 132.2, 130.2, 130.1, 129.9, 129.5, 128.2, 126.9, 124.5, 123.5, 58.6. UPLC-DAD-QTOF, HRMS (ESI) *m*/*z*: [M + H]^+^ calcd for C_21_H_17_N_3_O_3_,360.1270; found, 360.1274.

#### 2-((Bis(3,5-bis(trifluoromethyl)phenyl)methylene)amino)-*N*-(2-nitrophenyl) acetamide **2**

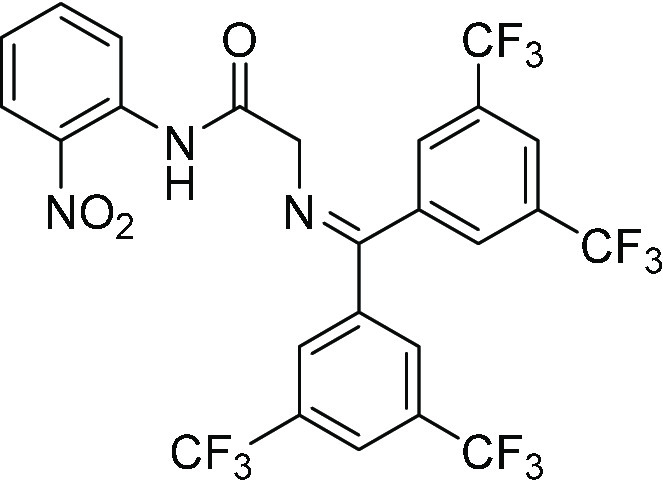



**N*-Boc glycine *o*-nitroanilide*: It was prepared following the same protocol as in the case of iminoamide **1**.

**N*-Deprotection and iminoamide formation*: To a solution of the previous *N*-Boc aminoamide (3 mmol) in CH_2_Cl_2_ (12 mL) was added trifluoroacetic acid (4.5 mL) at 0 °C. The mixture was stirred at room temperature for 30 min until full conversion (monitored by TLC). The solvents were evaporated, and the residue was coevaporated successively with a mixture of diethyl ether and pentane. Then it was dried *in vacuo*, and the resulting solid, obtained in a quantitative yield, was used in the next step without further purification. The resulting aminoamide trifluoroacetate salt was neutralized by the addition of a saturated aqueous solution of NaHCO_3_, followed by successive extractions with dichloromethane (≈70% yield in the extraction). To a suspension of the resulting free 2-amino-*N*-(2-nitrophenyl)acetamide (585 mg, 3 mmol) in CH_2_Cl_2_ (11 mL) were added bis(3,5-bis(trifluoromethyl)phenyl)methanimine hydrochloride (1.47 g, 3 mmol, 1 equiv) and anhydrous MgSO_4_ (903 mg, 7.5 mmol, 2.5 equiv). The reaction mixture was stirred at room temperature until consumption of the starting material (monitored by ^1^H NMR), then filtered to remove the salts, and evaporated *in vacuo*. The crude was crushed in diethyl ether/hexane to afford a pure yellow solid. Yield: 66% (1.25 g, 1.98 mmol). Mp: 178–180 °C. ^1^H NMR (300 MHz, CDCl_3_): δ 12.07 (s, 1H), 8.88 (d, *J* = 9.6 Hz, 0H), 8.31–8.22 (m, 2H), 8.13 (s, 1H), 8.06 (s, 1H), 7.76–7.59 (m, 2H), 7.24 (t, *J* = 9.1 Hz, 0H), 4.16 (s, 1H). ^13^C{^1^H} NMR (75 MHz, CDCl_3_): δ 168.2, 165.3, 139.0, 137.2, 136.4, 136.0, 133.2 (q), 128.7, 127.5, 126.0, 125.4, 124.4, 124.0, 122.3, 57.9. UPLC-DAD-QTOF, HRMS (ESI) *m*/*z*: [M + H]^+^ calcd C_25_H_14_F_12_N_3_O_3_, 632.0842; found, 632.0844.

#### 2-((Bis(4-(trifluoromethyl)phenyl)methylene)amino)-*N*-(2-nitrophenyl)acetamide **3**

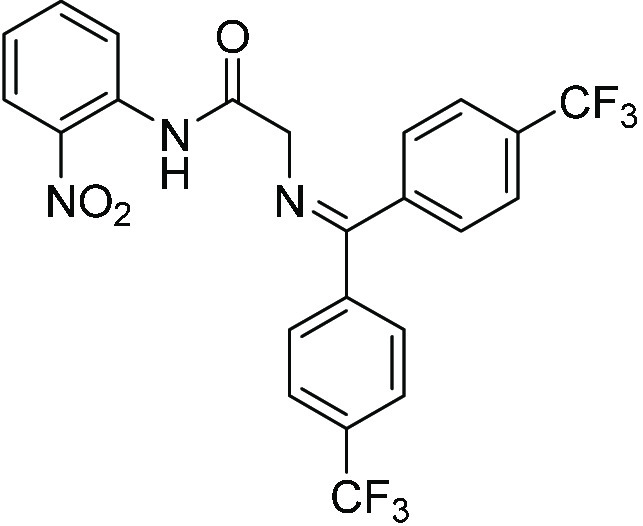



To a suspension of the free 2-amino-*N*-(2-nitrophenyl)acetamide, prepared as in the case of iminoamide **2**, (585 mg, 3 mmol) in CH_2_Cl_2_ (11 mL) were added bis(4-(trifluoro methyl)phenyl) methane iminium hydrochloride (1.06 g, 3 mmol, 1 equiv) and anhydrous MgSO_4_ (903 mg, 7.5 mmol, 2.5 equiv). The reaction mixture was stirred at room temperature until consumption of the starting material, then filtered to remove the salts, and evaporated *in vacuo*. The crude was crushed in diethyl ether/hexane to afford a pure yellow solid. Yield: 75% (1.1 g, 2.25 mmol). Mp: 165–168 °C. ^1^H NMR (300 MHz, CDCl_3_): δ 11.94 (s, 1H), 8.85 (dd, *J* = 8.5, 1.2 Hz, 1H), 8.26–8.16 (m, 1H), 7.91 (d, *J* = 8.1 Hz, 4H), 7.78 (d, *J* = 8.2 Hz, 4H), 7.70–7.60 (m, 1H), 7.23–7.16 (m, 1H), 3.57 (s, 2H). ^13^C{^1^H} NMR (75 MHz, CDCl_3_): δ 169.3, 168.5, 140.5, 139.0, 136.9, 135.8, 134.2, 129.0, 127.5, 126.5, 126.4, 125.9, 125.6, 125.5, 123.6, 122.3, 57.6. UPLC-DAD-QTOF, HRMS (ESI) *m*/*z*: [M + H]^+^ calcd for C_23_H_16_N_3_O_3_F_6_, 496.1096; found, 496.1102.

#### 2-((Diphenylmethylene)amino)-*N*-(4-nitrophenyl)acetamide **14**

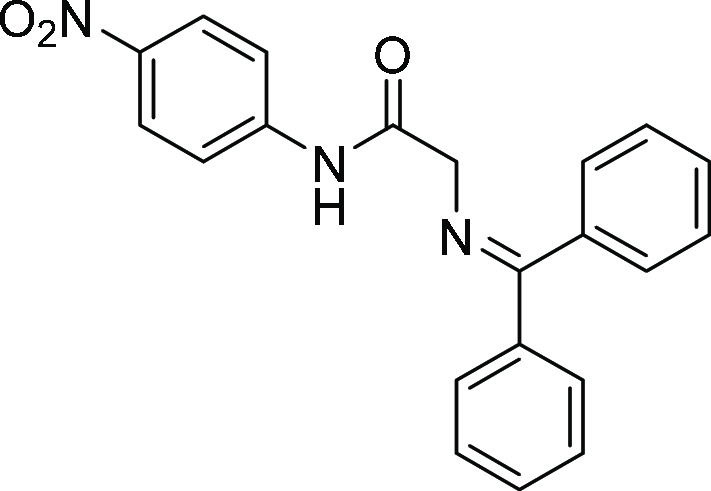



**N*-Boc glycine *p*-nitroanilide*: It was prepared following the same procedure as in the case of iminoamide **1**, but starting from p-nitroaniline (1.38 g, 10 mmol) and was obtained as a white solid. Yield: 69% (1.27 g, 3.9 mmol). ^1^H NMR (300 MHz, CDCl_3_): δ 8.21 (d, *J* = 9.2 Hz, 2H), 7.70 (d, *J* = 9.2 Hz, 2H), 3.95 (d, *J* = 6.1 Hz, 2H), 1.49 (s, 9H). All data were consistent with those previously reported.^44^

**N*-Deprotection*: The same protocol as that described for iminoamide **1** was followed starting from *N*-Boc glycine *p*-nitroanilide (885 mg, 3 mmol). Yield: quantitative. Mp: 153–155 °C. ^1^H NMR (300 MHz, D_2_O): δ 8.15 (d, *J* = 9.2 Hz, 2H), 7.61 (d, *J* = 9.2 Hz, 2H), 3.97 (s, 2H). ^13^C{^1^H} NMR (75 MHz, D_2_O): δ 165.8, 143.7, 142.9, 127.4, 125.1, 120.1, 114.4, 41.2. UPLC-DAD-QTOF, HRMS (ESI, measured after neutralization): *m*/*z* [M + H]^+^ calcd for C_8_H_10_N_3_O_3_, 196.0722; found, 196.0727.

*Iminoamide formation*: To a suspension of the aminoamide trifluororacetate salt obtained in the previous step (927 mg, 3 mmol, 1 equiv) in CH_2_Cl_2_ (11 mL) were added benzophenone imine (0.5 mL, 3 mmol, 1 equiv) and anhydrous MgSO_4_ (903 mg, 7.5 mmol, 2.5 equiv). The reaction mixture was stirred at room temperature until consumption of the starting material. The mixture was then filtered to remove the salts and evaporated *in vacuo*. The crude was crushed in diethyl ether/hexane to afford a pure white solid. Yield: 82% (883 mg, 2.5 mmol). Mp: 183–188 °C. ^1^H NMR (300 MHz, CDCl_3_): δ 9.78 (brs, 1H), 8.43–8.15 (m, 2H), 7.96–7.81 (m, 2H), 7.71 (dd, *J* = 8.2, 1.5 Hz, 2H), 7.61–7.39 (m, 5H), 7.39–7.08 (m, 3H), 4.13 (s, 2H). ^13^C{^1^H} NMR (75 MHz, CDCl_3_): δ 171.9, 170.0, 144.3, 144.0, 139.0, 136.5, 131.9, 130.1, 129.8, 129.2, 127.8, 125.9, 119.8, 57.4. UPLC-DAD-QTOF, HRMS (ESI) *m*/*z*: [M + H]^+^ calcd for C_21_H_17_N_3_O_3,_ 360.1270; found, 360.1372.

#### *N*-(2,4-Dinitrophenyl)-2-((diphenylmethylene)amino)acetamide **16**

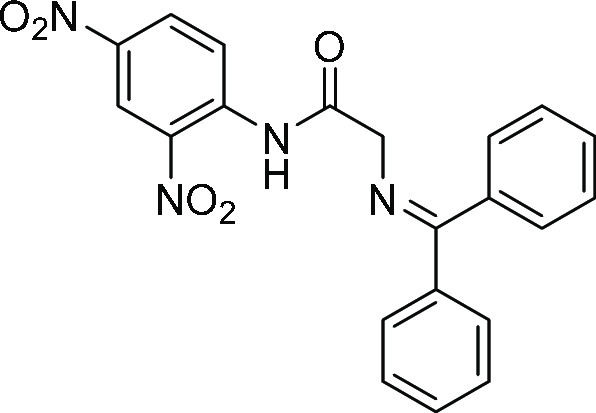



**N*-Boc glycine 2,4-dinitroanilide*:^43^ Boc-Gly-OH (1.05 g, 6 mmol, 1.2 equiv) was dissolved in dry DMF (14 mL), and DIPEA (5.2 mL, 30 mmol, 6 equiv) was added at room temperature. Then, 2,4-ninitroaniline (920 mg, 5 mmol, 1 equiv) was added, followed by HATU (2.09 g, 5.5 mmol, 1.1 equiv). The mixture was stirred at room temperature for 16 h. Then, a solution of EtOAc/H_2_O (1:1) was added to the reaction mixture, and it was extracted with EtOAc (3 × 50 mL), washed with brine (5 × 30 mL), dried over MgSO_4_, evaporated under reduced pressure, and purified by flash column chromatography on silica gel (hexane/EtOAc, 90:10) to afford the pure product as a yellow solid. Yield: 77% (1.31 g, 3.85 mmol). Mp: 186–188 °C. ^1^H NMR (300 MHz, CDCl_3_): δ 11.29 (s, 1H), 9.12–9.02 (m, 2H), 8.43 (dd, *J* = 9.4, 2.6 Hz, 1H), 5.53 (t, *J* = 5.7 Hz, 1H), 4.01 (d, *J* = 6.1 Hz, 2H), 1.45 (s, 9H). ^13^C{^1^H} NMR (75 MHz, CDCl_3_): δ 169.8, 156.2, 142.0, 139.2, 135.3, 130.2, 122.3, 122.1, 81.5, 46.0, 28.3. UPLC-DAD-QTOF, HRMS (ESI) *m*/*z*: [M + Na]^+^ calcd for C_13_H_16_N_4_O_7_Na, 363.0917; found, 363.0911.

**N*-Deprotection*: To a solution of the previously obtained *N*-Boc aminoamide (1.02 g, 3 mmol) in CH_2_Cl_2_ (12 mL) was added trifluoroacetic acid (4.5 mL) at 0 °C. The mixture was stirred at room temperature for 30 min until full conversion. The solvents were evaporated, and the residue was coevaporated successively with a mixture of diethyl ether and pentane. Then it was dried *in vacuo*, and the resulting solid, which was obtained in a quantitative yield, was used in the next step without further purification. Yield: quantitative. Mp: 146–150 °C. ^1^H NMR (300 MHz, D_2_O): δ 8.97 (d, *J* = 2.6 Hz, 1H), 8.57–8.42 (m, 1H), 8.25 (d, *J* = 9.2 Hz, 1H), 4.11 (s, 2H). ^13^C{^1^H} NMR (75 MHz, D_2_O): δ 166.4, 143.5, 139.3, 136.3, 129.4, 125.4, 121.9, 41.7. UPLC-DAD-QTOF, HRMS (ESI, measured after neutralization) *m*/*z*: [M + Na]^+^ calcd for C_8_H_8_N_4_O_5_Na, 263.0392; found, 263.0403.

*Iminoamide formation*: To a suspension of the aminoamide trifluororacetate salt obtained in the previous step (1.06 mg, 3 mmol, 1 equiv) in CH_2_Cl_2_ (11 mL) were added benzophenone imine (0.5 mL, 3 mmol, 1 equiv) and anhydrous MgSO_4_ (903 mg, 7.5 mmol, 2.5 equiv). The reaction mixture was stirred at room temperature until consumption of the starting material (monitored by ^1^H NMR). The mixture was then filtered to remove the salts and evaporated *in vacuo*. The crude was crushed in diethyl ether/hexane to afford a pure yellow solid. Yield: 66% (800 mg, 1.98 mmol). Mp: 175–180 °C. ^1^H NMR (300 MHz, CDCl_3_): δ 12.53 (s, 1H), 9.24 (d, *J* = 9.4 Hz, 1H), 9.18 (d, *J* = 2.7 Hz, 1H), 8.48 (dd, *J* = 9.4, 2.7 Hz, 1H), 7.87 (dd, *J* = 8.1, 1.6 Hz, 2H), 7.60–7.38 (m, 6H), 7.23–7.12 (m, 2H), 4.18 (s, 2H). ^13^C{^1^H} NMR (75 MHz, CDCl_3_): δ 172.0, 171.1, 139.5, 138.1, 136.2, 132.5, 131.4, 130.2, 130.0, 129.4, 129.3, 128.97, 128.6, 128.4, 127.1, 122.7, 122.2, 57.6. UPLC-DAD-QTOF, HRMS (ESI) *m*/*z*: [M + H]^+^ calcd for C_21_H_17_N_4_O_5_, 405.1199; found, 405.1192.

### Preparation of *N*-Methyl Iminoamide **15**

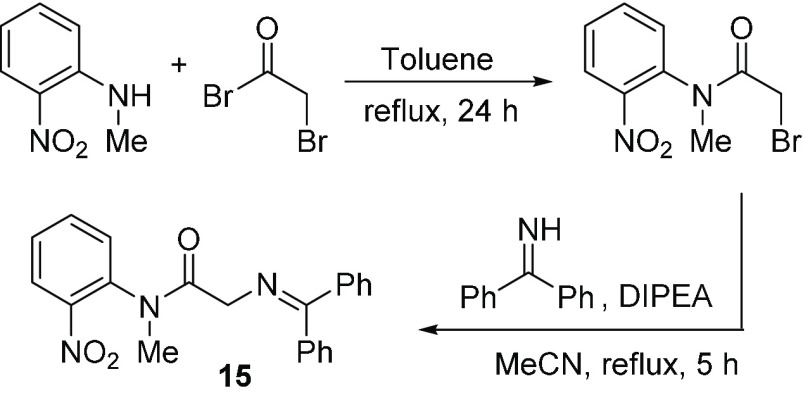



In step 1,^44^*N*-methyl-2-nitroaniline (1.5 g, 10 mmol, 1 equiv) was dissolved in toluene (25 mL), and bromoacetyl bromide (1.0 mL, 12 mmol, 1 equiv) was added. The resulting solution was refluxed overnight (caution: HBr evolution), cooled, and concentrated *in vacuo*. The residue was purified through silica gel chromatography (eluting with hexane/EtOAc 70:30), affording the desired product as a yellow oil in 94% yield (2.27 g, 9.7 mmol). The spectroscopic data were coincident with those described in the literature.^45^^1^H NMR (300 MHz, CHCl_3_), major rotamer: δ 8.05 (dd, *J* = 8.1, 1.5 Hz, 1H), 7.74 (dd, *J* = 7.7, 1.6 Hz, 1H), 7.62 (td, *J* = 7.9, 1.5 Hz, 1H), 7.53 (dd, *J* = 7.8, 1.4 Hz, 1H), 3.65 (d, *J* = 11.2 Hz, 1H), 3.52 (d, *J* = 10.8 Hz, 1H), 3.23 (s, 3H); minor rotamer δ 7.99 (dd, *J* = 8.2, 1.4 Hz, 1H), 7.71–7.64 (m, 1H), 7.48 (td, *J* = 8.1, 1.4 Hz, 1H), 7.34 (dd, *J* = 7.9, 1.3 Hz, 1H), 3.98 (s, 2H), 3.50 (s, 3H).

In step 2,^46^ to a solution of the bromide obtained in the previous step (546 mg, 2 mmol, 1 equiv) and DIPEA (0.35 mL, 2 mmol, 1 equiv) in dry acetonitrile (4 mL) under argon was added benzophenone imine (0.34 mL, 2 mmol, 1 equiv), and the resulting solution was refluxed for 5 h. The mixture was then cooled, dissolved in CH_2_Cl_2_ (20 mL), and washed with aqueous 5% NaHCO_3_ (20 mL). The water phase was extracted with CH_2_Cl_2_ (2 × 15 mL), and the organic phases were combined and dried over MgSO_4_. The solvent was eliminated under reduced pressure, and the residue was purified through flash chromatography on silica gel (eluting with hexane/EtOAc 60:40) to afford the desired product as a viscous orange oil in 70% yield (520 mg, 1.39 mmol). ^1^H NMR (300 MHz, CDCl_3_), major rotamer: δ 8.00–7.87 (m, 1H), 7.74–7.57 (m, 2H), 7.54–7.42 (m, 3H), 7.43–7.16 (m, 7H), 7.05–6.95 (m, 1H), 3.92 (s, 2H), 3.25 (s, 3H); Minor rotamer: δ 8.00–7.87 (m, 1H), 7.74–7.57 (m, 2H), 7.54–7.42 (m, 3H), 7.43–7.16 (m, 7H), 7.05–6.95 (m, 1H), 3.55 (s, 3H), 3.49 (s, 3H). ^13^C{^1^H} NMR (75 MHz, CDCl_3_), mixture of rotamers: δ 171.2, 169.2, 146.4, 139.0, 136.8, 135.5, 134.2, 134.0, 131.5, 130.8, 130.3, 130.2, 129.4, 129.2, 128.8, 128.7, 128.6, 128.4, 128.1, 128.0, 127.8, 127.71, 127.5, 125.5, 125.0, 124.0, 114.1, 56.9, 56.4, 53.4, 38.2, 37.1, 29.5. UPLC-DAD-QTOF, HRMS (ESI) *m*/*z*: [M + H]^+^ calcd for C_22_H_20_N_3_O_3_, 373.1426; found, 373.1425.

### Preparation of Benzophenone Imine of Glycine Methyl Ester **13**.^47^

To a suspension of glycine ester hydrochloride (377 mg, 3 mmol, 1 equiv) in DCM (6 mL) was added benzophenone imine (0.5 mL, 3 mmol, 1 equiv). Triethylamine (0.42 mL, 3 mmol, 1 equiv) was then added dropwise, and the reaction mixture was stirred at room temperature until consumption of the starting material (monitored by ^1^H NMR). The mixture was then diluted with Et_2_O (6 mL), filtered, and washed with H_2_O (3 × 10 mL) and brine (3 × 10 mL). The combined organic layers were dried over MgSO_4_ and evaporated to dryness. The crude was obtained with a quantitative yield (759 mg, 3 mmol) and was used without further purification. All of the spectroscopic data were coincident with those previously reported. ^1^H NMR (300 MHz, CDCl_3_): δ 7.74–7.61 (m, 2H), 7.56–7.42 (m, 4H), 7.43–7.29 (m, 3H), 7.25–7.14 (m, 1H), 4.25 (s, 2H), 3.77 (s, 3H).

### Preparation of α-Pyridyl and Phenyl Acetanilides **18**–**21**

#### Synthesis of 2-(Pyridin-2-yl)acetanilides.^48^

##### General Procedure

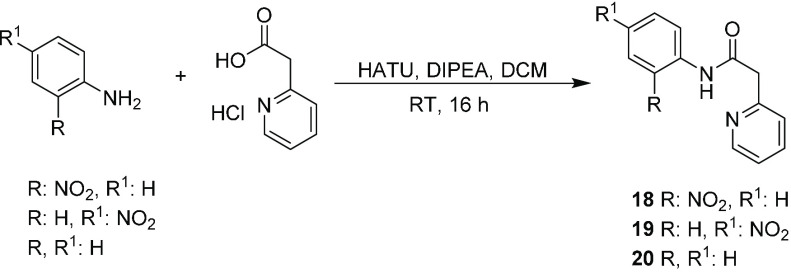



To a suspension of 2-pyridylacetic acid hydrochloride (1.42 g, 6 mmol, 1.2 equiv) in dry Cl_2_CH_2_ (12 mL) were added DIPEA (5.2 mL, 30 mmol, 6 equiv) and the corresponding aniline (5 mmol, 1 equiv), followed by HATU (2.09 g, 5.5 mmol, 1.1 equiv). The mixture was stirred at room temperature for 16 h. Then, a solution of Cl_2_CH_2_/H_2_O (1:1, 30 mL) was added to the reaction mixture, and it was extracted with Cl_2_CH_2_ (3 × 50 mL), washed with brine (2 × 30 mL), dried over MgSO_4_, and evaporated under reduced pressure. The residue was purified by flash column chromatography on silica gel (eluting with hexane/EtOAc 80:20) to afford the desired product.

##### *N*-(2-Nitrophenyl)-2-(pyridin-2-yl)acetamide **18**

The compound was prepared according to the general procedure starting from *o*-nitroaniline (852, 6 mmol) and 2-pyridylacetic acid hydrochloride (1.42 g, 6 mmol, 1.2 equiv) and was obtained as an orange solid in 54% yield (832g, 3.3 mmol). Mp: 69–71 °C. ^1^H-RMN (300 MHz, CDCl_3_): δ (ppm) 11.43 (s, 1H), 8.76 (dd, *J* = 8.5, 1.2 Hz, 2H), 8.17 (dd, *J* = 8.4, 1.6 Hz, 1H), 7.74 (td, *J* = 7.7, 1.8 Hz, 1H), 7.69–7.59 (m, 1H), 7.33 (t, *J* = 6.3 Hz, 1H), 7.18 (ddd, *J* = 8.5, 7.3, 1.3 Hz, 2H), 4.02 (s, 2H). ^13^C{^1^H} NMR (75 MHz, CDCl_3_): δ (ppm) 150.4, 138.0, 136.0, 126.2, 124.5, 124.0, 123.5, 123.2, 47.8. UPLC-DAD-QTOF, HRMS (ESI) *m*/*z*: [M + H]^+^ calcd for C_13_H_11_N_2_O_3_, 257.0800; found, 257.0821.

##### *N*-(4-Nitrophenyl)-2-(pyridin-2-yl)acetamide **19**

The compound was prepared according to the general procedure starting from *p*-nitroaniline (852, 6 mmol) and 2-pyridylacetic acid hydrochloride (1.42 g, 6 mmol, 1.2 equiv) and was obtained as a yellow solid in 57% yield (852g, 3.42 mmol). Mp: 155–158 °C. ^1^H NMR (300 MHz, CDCl_3_): δ 10.79 (s, 1H), 8.67 (dt, *J* = 4.8, 1.4 Hz, 1H), 8.48–8.09 (m, 2H), 7.94–7.64 (m, 2H), 7.50–6.87 (m, 2H), 3.94 (s, 2H). ^13^C{^1^H} NMR (75 MHz, CDCl_3_): δ 168.2, 155.6, 149.3, 144.1, 138.7, 125.9, 125.4, 123.1, 119.8, 119.6, 46.0. UPLC-DAD-QTOF, HRMS (ESI) *m*/*z*: [M + H]^+^ calcd for C_13_H_11_N_2_O_3_, 257.0800; found, 257.0802.

##### *N*-Phenyl-2-(pyridin-2-yl)acetamide **20**

The compound was prepared according to the general procedure starting from aniline (0.55 mL, 6 mmol) and 2-pyridineacetic acid hydrochloride (1.42 g, 6 mmol, 1.2 equiv) and was obtained as a yellow solid. Yield: 34% (0.432 mg, 2.04 mmol). Mp: 135–138 °C. ^1^H NMR (300 MHz, CDCl_3_): δ 9.83 (s, 1H), 8.64 (ddd, *J* = 5.0, 1.9, 1.0 Hz, 1H), 7.72 (td, *J* = 7.7, 1.8 Hz, 1H), 7.65–7.46 (m, 2H), 7.39–7.21 (m, 5H), 7.21–7.00 (m, 1H), 3.90 (s, 2H). ^13^C{^1^H} NMR (75 MHz, CDCl_3_): δ 170.9, 136.4, 135.2, 134.7, 130.1, 129.9, 128.8, 126.2, 123.8, 122.5, 46.8. UPLC-DAD-QTOF, HRMS (ESI) *m*/*z*: [M + H]^+^ calcd for C_13_H_12_N_2_O, 212.0950; found, 212.0958.

#### Synthesis of *N*-(2-Nitrophenyl)-2-phenylacetamide **21**

A solution of benzeneacetic acid (1.36 g, 10 mmol, 1.25 equiv) in Cl_2_CH_2_ (10 mL) was cooled to −15 °C, and SOCl_2_ (101 mL, 15 mmol, 1.5 equiv) and DMF (2 drops) were added dropwise with vigorous stirring. After the mixture was stirred for 2 h at room temperature, a mixture of *o*-nitroaniline (8 mmol, 1 equiv) and K_2_CO_3_ (1.5 g) in dry Cl_2_CH_2_ (5 mL) was added, and the resulting suspension was stirred at room temperature for 16 h. The reaction mixture was then quenched with ice–water (100 mL) and extracted with EtOAc (4 × 60 mL). The organic phase was dried over MgSO_4_, and the solvent was evaporated *in vacuo*. The residue was crushed with diethyl ether and hexane to afford a white solid. Mp: 79–81 °C. Yield: 81% (1.8 g, 7 mmol). ^1^H NMR (300 MHz, CDCl_3_): δ 10.28 (s, 1H), 8.81 (dd, *J* = 8.6, 1.3 Hz, 1H), 8.15 (dd, *J* = 8.4, 1.5 Hz, 1H), 7.63 (ddd, *J* = 8.6, 7.3, 1.6 Hz, 1H), 7.56–7.32 (m, 5H), 7.22–6.82 (m, 1H), 3.84 (s, 3H). ^13^C{^1^H} NMR (75 MHz, CDCl_3_): δ 170.9, 136.4, 135.2, 134.7, 130.1, 129.1, 128.8, 126.2, 123.8, 122.5, 46.9. UPLC-DAD-QTOF, HRMS (ESI) *m*/*z*: [M + H]^+^ calcd for C_14_H_12_N_2_O_3_, 256.0848; found, 256.0850.

### General Procedure for the Asymmetric Aldol Reaction of Schiff Bases of Glycine Nitroanilide

The corresponding nitroanilide (0.2 mmol, 1 equiv) and the corresponding catalyst (0.02 mmol, 20 mol %) were dissolved in dry dichloromethane (0.5 mL) at the indicated temperature. To the mixture was added NaHCO_3_ (0.02 mmol, 20 mol %) in one portion, followed by the corresponding freshly distilled aldehyde (previously washed with a saturated NaHCO_3_ solution) (0.6 mmol, 3 equiv). The reaction mixture was stirred at the indicated temperature until consumption of the starting material (monitored by ^1^H NMR). Then, MeOH (0.4 mL) was added, followed by NaBH_3_CN (32 mg, 0.5 mmol, 2.5 equiv) and AcOH (24 μL, 0.4 mmol, 2 equiv), and the mixture was stirred for 2 h (the reduction of the imine can be monitored by ^1^H NMR). The solvents were evaporated under reduced pressure, and the residue was redissolved in dichloromethane and washed with a saturated NaHCO_3_ solution (1 × 4 mL). The organic phase was dried over MgSO_4_ and evaporated *in vacuo*. The crude was purified by flash column chromatography.

#### (2*S*,3*R*)-2-(Benzhydrylamino)-3-hydroxy-*N*-(2-nitrophenyl)-5-phenylpentanamide **5a**

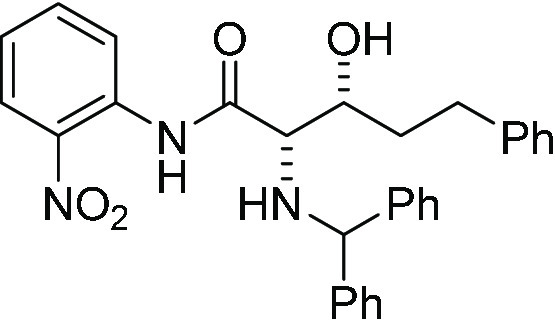



The compound was prepared according to the general procedure starting from 2-((diphenyl methylene)amino)-*N*-(2-nitrophenyl) acetamide **1** (72 mg, 0.2 mmol) and hydrocinnamaldehyde **4a** (80 μL, 0.6 mmol) and was purified by flash column chromatography on silica gel (hexane/EtOAc, 90:10) to afford **5a** as a yellow oil. Yield: 71% (70 mg, 0.14 mmol). [α]_D_^23^ −1.3 (*c* 1.35, 94% ee, CH_2_Cl_2_). ^1^H NMR (300 MHz, CDCl_3_): δ 11.77 (s, 1H), 8.72 (d, *J* = 8.5 Hz, 1H), 8.20 (d, *J* = 10.0 Hz, 1H), 7.60 (t, *J* = 8.6 Hz, 1H), 7.52–7.45 (m, 2H), 7.46–7.31 (m, 4H), 7.31–7.05 (m, 9H), 4.91 (s, 12H), 4.07 (m, 1H), 3.32 (d, *J* = 3.9 Hz, 1H), 2.86 (m, 1H), 2.67 (m, 1H), 1.88 (m, 2H). ^13^C{^1^H} NMR (75 MHz, CDCl_3_): δ 173.6, 143.0, 142.4, 141.4, 137.1, 135.7, 134.0, 129.0, 128.8, 128.7, 128.6, 127.8, 127.6, 127.4, 126.2, 125.9, 123.7, 122.2, 72.1, 66.8, 65.0, 35.4, 32.4. UPLC-DAD-QTOF, HRMS (ESI) *m*/*z*: [M + H]^+^ calcd C_30_H_30_N_3_O_4_, 496.2233; found, 496.2236. The enantiomeric purity was determined by HPLC analysis (Daicel Chiralcel IA hexane/ethanol 90:10, flow rate = 1 mL/min, retention times: 18 min (major) and 40 min (minor)).

#### (2*S*,3*R*)-2-((Bis(3,5-bis(trifluoromethyl)phenyl)methyl)amino)-3-hydroxy-*N*-(2-nitrophenyl)-5-phenylpentanamide **6a**

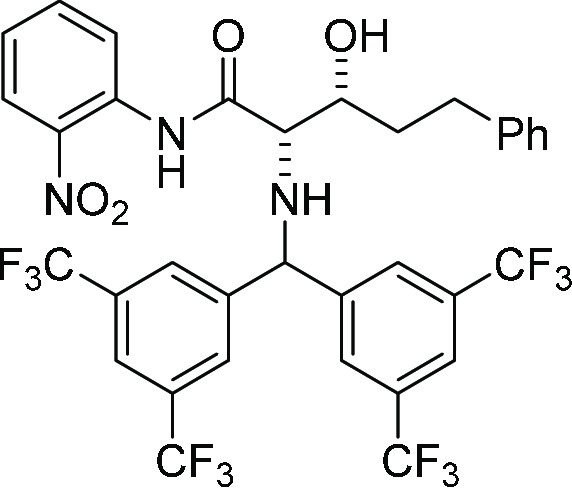



The compound was prepared according to the general procedure starting from 2-((bis(3,5-bis(trifluoromethyl)phenyl)methylene)amino)-*N*-(2-nitrophenyl) acetamide **2** (126 mg, 0.2 mmol) and hydrocinnamaldehyde **4a** (80 μL, 0.6 mmol) and was isolated by flash column chromatography on silica gel (hexane/EtOAc, 90:10) as a yellow oil. Yield (diastereomeric ratio 88:12): 84% (129 mg, 0.17 mmol). ^1^H NMR (500 MHz, CDCl_3_): δ 11.75 (s, 1H), 8.75 (d, *J* = 9.5 Hz, 1H), 8.26 (d, *J* = 9.9 Hz, 1H), 8.00 (s, 2H), 7.88 (s, 3H), 7.71–7.63 (m, 1H), 7.33–7.27 (m, 2H), 7.23–7.14 (m, 4H), 5.13 (s, 1H), 4.21–4.11 (m, 1H), 3.24–3.14 (m, 1H), 2.93–2.85 (m, 1H), 2.80–2.67 (m, 1H), 2.01–1.89 (m, 2H). ^13^C{^1^H} NMR (126 MHz, CDCl_3_): δ 171.8, 143.8, 143.6, 140.8, 136.1, 133.8, 133.5–132.2 (m), 128.9, 128.6, 127.8, 127.4, 126.6, 126.1, 121.8, 72.1, 65.8, 65.4, 35.5, 32.3, 29.9. UPLC-DAD-QTOF, HRMS (ESI) *m*/*z*: [M + H]^+^ calcd for C_34_H_25_F_12_N_3_O_4_, 768.1746; found, 768.1732. The enantiomeric purity was determined by HPLC analysis (Daicel Chiralpak IAIA) hexane/isopropanol 98:2, flow rate = 0.5 mL/min. Retention times: minor diastereoisomer, 30 min (major) and 36 min (minor); major diastereoisomer, 49 min (major) and 56 min (minor).

#### (2*S*,3*R*)-2-((Bis(4-(trifluoromethyl)phenyl)methyl)amino)-3-hydroxy-*N*-(2-nitrophenyl)-5-phenylpentanamide **7a**

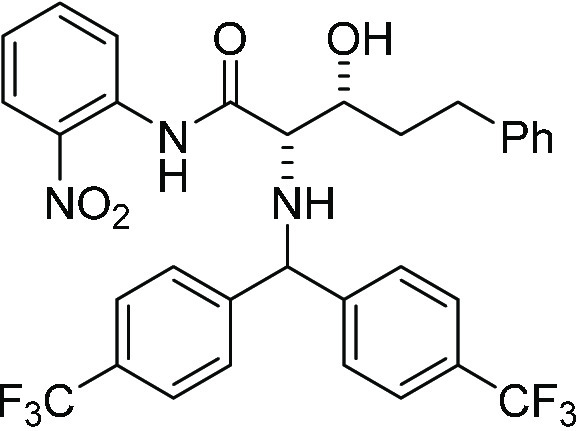



The compound was prepared according to the general procedure starting from 2-((bis(4-(trifluoromethyl) phenyl)methylene)amino)-*N*-(2-nitrophenyl) acetamide **3** (99 mg, 0.2 mmol) and hydrocinnamaldehyde **4a** (80 μL, 0.6 mmol) and was isolated by flash column chromatography on silica gel (hexane/EtOAc, 90:10) to afford **7a** as a yellow oil. Yield: 64% (81 mg, 0.13 mmol). [α]_D_^24^ −20.8 (*c* 1, 92% ee, CH_2_Cl_2_). ^1^H NMR (300 MHz, CDCl_3_): δ 11.76 (s, 1H), 8.83–8.60 (m, 1H), 8.24 (d, *J* = 8.4 Hz, 1H), 7.71–7.58 (m, 5H), 7.52 (d, *J* = 1.8 Hz, 4H), 7.36–7.23 (m, 3H), 7.24–7.12 (m, 4H), 5.04 (s, 1H), 4.19–4.06 (m, 1H), 3.28 (d, 1H), 4.12 (m, 1H), 3.38–3.18 (m, 1H), 3.00–2.83 (m, 1H), 2.67 (m, 1H), 1.93 (m, 2H). ^13^C{^1^H} NMR (75 MHz, CDCl_3_): δ 172.8, 146.0, 145.6, 141.2, 136.9, 136.0, 133.9, 130.6 (q), 128.8, 128.6, 128.1, 127.7, 124.0, 122.1, 72.0, 66.1, 65.2, 35.5, 32.4. UPLC-DAD-QTOF, HRMS (ESI) *m*/*z*: [M + H]^+^ calcd for C_32_H_28_F_6_N_3_O_4_, 632.1992; found, 632.1984. The enantiomeric purity was determined by HPLC analysis (Daicel Chiralpak IF) hexane/isopropanol 98:2, flow rate = 0.5 mL/min. Retention times: minor diastereoisomer, 32 min (minor) and 37 min (major); major diastereoisomer, 40 min (minor) and 45 min (major).

#### (2*S*,3*R*)-2-((Bis(4-(trifluoromethyl)phenyl)methyl)amino)-3-hydroxy-*N*-(2-nitrophenyl)pentanamide **7b**

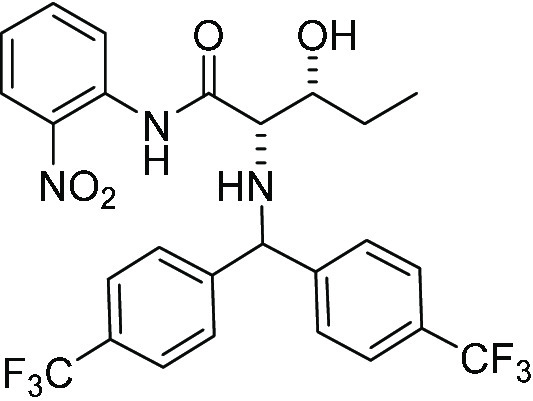



The compound was prepared according to the general procedure starting from 2-((bis(4-(trifluoromethyl) phenyl) methylene)amino)-*N*-(2-nitrophenyl) acetamide **3** (99 mg, 0.2 mmol) and propionaldehyde **4b** (72 μL, 1 mmol, 5 equiv) and was isolated by column chromatography (hexane/EtOAc, 85:15) as a yellow foam. Yield (diastereomeric ratio 98:2): 76% (84 mg, 0.15 mmol). ^1^H NMR (300 MHz, CDCl_3_), major diastereomer: δ 11.80 (s, 1H), 8.75 (d, *J* = 8.5 Hz, 1H), 8.23 (d, *J* = 9.5 Hz, 1H), 7.65 (d, *J* = 9.3 Hz, 5H), 7.53 (q, *J* = 8.4 Hz, 4H), 7.21 (t, *J* = 7.8 Hz, 1H), 5.11 (s, 1H), 4.05 (dd, *J* = 9.9, 5.8 Hz, 1H), 3.26 (d, *J* = 3.1 Hz, 1H), 2.93 (s, 0H), 2.43 (s, 1H), 1.65 (dq, *J* = 15.0, 7.0 Hz, 2H), 1.02 (t, *J* = 7.3 Hz, 3H). ^13^C{^1^H} NMR (75 MHz, CDCl_3_), major diastereomer: δ 172.7, 146.0, 145.6, 136.8, 135.8, 133.9, 130.5 (q), 128.0, 127.5, 126.1, 126.0, 125.8, 123.7, 121.9, 74.2, 66.2, 64.9, 26.9, 10.4. UPLC-DAD-QTOF, HRMS (ESI) *m*/*z*: [M + H]^+^ calcd for C_26_H_24_F_6_N_3_O_4_, 556.1671; found, 556.1670. The enantiomeric purity of the major isomer was determined by chiral HPLC analysis of the crude reaction mixture (Phenomenex Amylose-1, hexane/isopropanol, 98:2; flux = 1 mL/min. Retention times for major diastereomer: 62.2 min (minor), 72.8 min (major); minor diastereomer, 48.5 min (minor), 53.6 min (major))

#### (2*S*,3*R*)-2-((Bis(4-(trifluoromethyl)phenyl)methyl)amino)-3-hydroxy-*N*-(2-nitrophenyl)hexanamide **7c**

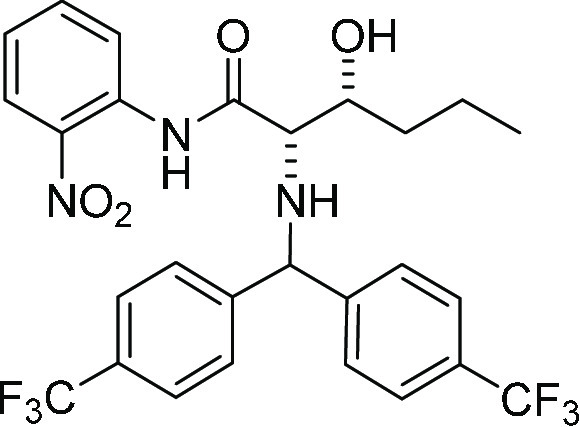



The compound was prepared according to the general procedure starting from 2-((bis(4-(trifluoromethyl)phenyl)methylene)amino)-*N*-(2-nitrophenyl)acetamide **3** (99 mg, 0.2 mmol) and butyraldehyde **4c** (54 μL, 0.6 mmol) and was purified by flash column chromatography on silica gel (hexane/EtOAc, 95:5) to afford **7c** as a yellow oil. Yield: 72% (80 mg, 0.14 mmol). [α]_D_^24^ −13.9 (*c* 0.5, 92% ee, CH_2_Cl_2_). ^1^H NMR (300 MHz, CDCl_3_): δ 11.80 (s, 1H), 8.76 (d, *J* = 8.4 Hz, 1H), 8.24 (d, *J* = 8.4 Hz, 1H), 7.71–7.66 (m, 4H), 7.66–7.62 (m, 1H), 7.54 (q, *J* = 8.3 Hz, 4H), 7.22 (t, *J* = 7.6 Hz, 1H), 5.10 (s, 1H), 4.31–3.97 (m, 1H), 3.44–2.99 (m, 1H), 1.72–1.51 (m, 3H), 1.46–1.31 (m, 1H), 1.10–0.65 (m, 3H). ^13^C{^1^H} NMR (75 MHz, CDCl_3_): δ 172.9, 146.1, 145.7, 136.0, 134.0, 130.56 (q), 128.1, 127.7, 126.3, 126.2, 126.0, 123.9, 122.0, 72.6, 66.3, 65.3, 36.1, 19.3, 14.0. UPLC-DAD-QTOF, HRMS (ESI) *m*/*z*: [M + H]^+^ calcd for C_27_H_26_F_6_N_3_O_4_, 570.1836; found, 570.1828. The enantiomeric purity was determined by HPLC analysis (Phenomenex-Lux 3 μm i-Amilose-1 (00G-4729-E0)) hexane/isopropanol 95:5, flow rate = 0.5 mL/min. Retention times: minor diastereoisomer, 31 min (major) and 41 min (minor); major diastereoisomer, 45 min (minor) and 49 min (major).

#### (2*S*,3*R*)-2-((Bis(4-(trifluoromethyl)phenyl)methyl)amino)-3-hydroxy-*N*-(2-nitrophenyl)octanamide **7d**

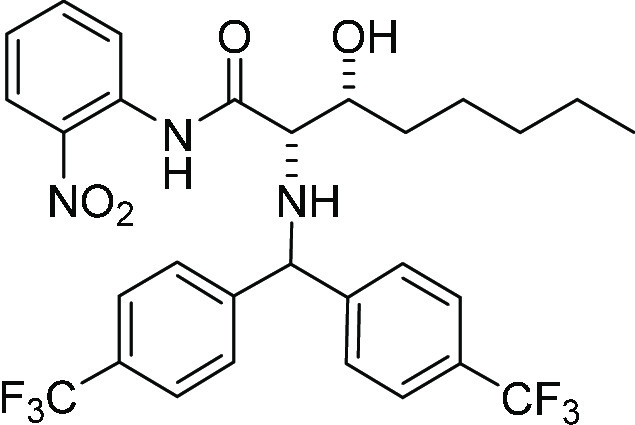



The compound was prepared according to the general procedure starting from 2-((bis(4-(trifluoromethyl)phenyl)methylene)amino)-*N*-(2-nitrophenyl)acetamide **3** (99 mg, 0.2 mmol) and hexanal **4d** (74 μL, 0.6 mmol) and was purified by flash column chromatography on silica gel (hexane/EtOAc, 95:5) to afford **7d** as a yellow oil. Yield: 82% (98 mg, 0.16 mmol). [α]_D_^24^ −2.4 (*c* 1, 90% ee, CH_2_Cl_2_). ^1^H NMR (300 MHz, CDCl_3_): δ 11.80 (s, 1H), 8.75 (dd, *J* = 8.5, 1.2 Hz, 1H), 8.23 (dd, *J* = 8.5, 1.5 Hz, 1H), 7.66 (s, 4H), 7.65–7.60 (m, 1H), 7.54 (q, *J* = 8.5 Hz, 4H), 7.21 (t, 1H), 5.10 (s, 1H), 4.23–4.04 (m, 1H), 3.25 (d, *J* = 3.7 Hz, 1H), 1.69–1.51 (m, 2H), 1.40–1.20 (m, 4H), 0.99–0.81 (m, 3H). ^13^C{^1^H} NMR (75 MHz, CDCl_3_): δ 172.9, 146.2, 145.8, 136.9, 136.0, 134.0, 130.3 (q, *J* = 32.6, 10.5 Hz), 128.1, 127.7, 126.2, 126.2, 126.0, 123.8, 122.0, 72.8, 66.3, 65.3, 34.0, 31.8, 25.8, 22.7, 14.1. UPLC-DAD-QTOF, HRMS (ESI) *m*/*z*: [M + H]^+^ calcd for C_29_H_30_F_6_N_3_O_4_, 598.2138; found, 598.2141. The enantiomeric purity was determined by HPLC analysis (Phenomenex-Lux 3 μm i-Amilose-1 (00G-4729-E0)) hexane/isopropanol 95:5, flow rate = 0.5 mL/min. Retention times: minor diastereoisomer, 31 min (major) and 39 min (minor); major diastereoisomer, 43 min (minor) and 46 min (major).

#### (2*S*,3*R*)-2-((Bis(4-(trifluoromethyl)phenyl)methyl)amino)-3-hydroxy-*N*-(2-nitrophenyl)nonanamide **7e**

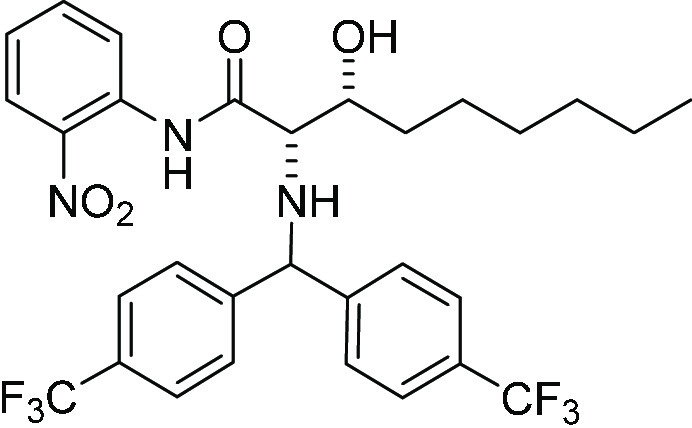



The compound was prepared according to the general procedure starting from 2-((bis(4-(trifluoromethyl)phenyl)methylene) amino)-*N*-(2-nitrophenyl)acetamide **3** (99 mg, 0.2 mmol) and heptanal **4e** (84 μL, 0.6 mmol) and was isolated by flash column chromatography on silica gel (hexane/EtOAc, 94:6) as a yellow oil. Yield (diastereomeric ratio 92:8): 76% (93 mg, 0.15 mmol). ^1^H NMR (300 MHz, CDCl_3_): δ 11.80 (s, 1H), 8.75 (d, *J* = 8.5 Hz, 1H), 8.23 (d, *J* = 8.4 Hz, 1H), 7.66 (s, 4H), 7.62 (t, *J* = 8.6 Hz, 1H), 7.51 (d, *J* = 8.4 Hz, 4H), 7.22 (t, *J* = 7.8 Hz, 1H), 5.10 (s, 1H), 4.13 (s, 1H), 3.37–3.19 (m, 1H), 1.71–1.47 (m, 2H), 1.41–1.20 (m, 8H), 1.04–0.76 (m, 3H). ^13^C{^1^H} NMR (75 MHz, CDCl_3_): δ 173.5, 146.7, 146.3, 137.5, 136.5, 134.6, 131.0 (q, *J* = 32.4 Hz), 128.6, 128.2, 126.8, 126.7, 126.5, 126.5, 124.4, 122.6, 73.4, 66.9, 65.8, 34.6, 32.4, 29.8, 26.6, 23.2, 14.7. UPLC-DAD-QTOF, HRMS (ESI) *m*/*z*: [M + H]^+^ calcd for C_30_H_32_F_6_N_3_O_4_, 612.2298; found, 612.2297. The enantiomeric purity was determined by HPLC analysis (Phenomenex-Lux 3 μm i-Cellulose) hexane/isopropanol 95:5, flow rate = 0.5 mL/min. Retention times: minor diastereoisomer, 19 min (major) and 21 min (minor); major diastereoisomer, 23 min (major) and 29 min (minor).

#### (2*S*,3*R*)-2-((Bis(4-(trifluoromethyl)phenyl)methyl)amino)-3-hydroxy-5-methyl-*N*-(2-nitrophenyl)hexanamide **7f**

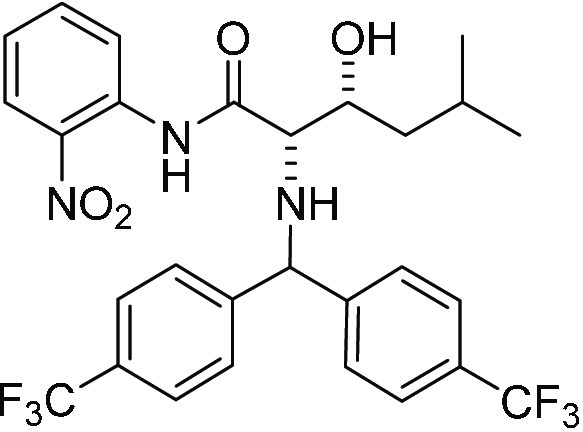



The compound was prepared according to the general procedure starting from 2-((bis(4-(trifluoromethyl)phenyl)methylene)amino)-*N*-(2-nitrophenyl)acetamide **3** (99 mg, 0.2 mmol) and isovaleraldehyde **4f** (64 μL, 0.6 mmol) and was purified by flash column chromatography on silica gel (hexane/EtOAc, 95:5) to afford **7f** as a yellow oil. Yield: 78% (91 mg, 0.16 mmol). [α]_D_^24^ −6.9 (*c* 0.5, 84% ee, CH_2_Cl_2_). ^1^H NMR (300 MHz, CDCl_3_): δ 11.80 (s, 1H), 8.75 (d, *J* = 8.5 Hz, 1H), 8.24 (d, *J* = 1.4 Hz, 1H), 7.66 (s, 4H), 7.65–7.60 (m, 1H), 7.54 (q, *J* = 8.5 Hz, 3H), 7.21 (td, *J* = 7.9, 7.4, 1.3 Hz, 1H), 5.10 (s, 6H), 4.31–4.18 (m, 1H), 3.23 (d, *J* = 3.6 Hz, 1H), 1.79 (s, 0H), 1.56 (d, *J* = 4.1 Hz, 1H), 1.37 (d, *J* = 8.9 Hz, 1H), 1.01–0.88 (m, 8H). ^13^C{^1^H} NMR (75 MHz, CDCl_3_): δ 172.9, 146.2, 145.8, 137.0, 136.0, 134.0, 130.42 (q, *J* = 32.6 Hz), 129.6, 128.1, 127.7, 126.2, 126.0, 123.9, 122.1, 70.9, 66.3, 65.5, 42.9, 24.9, 23.6, 21.8. UPLC-DAD-QTOF, HRMS (ESI) *m*/*z*: [M + H]^+^ calcd for C_28_H_28_F_6_N_3_O_4_, 584.1980; found, 584.1987. The enantiomeric purity was determined by HPLC analysis (Phenomenex-Lux 3 μm i-Amilose-1 (00G-4729-E0)) hexane/isopropanol 95:5, flow rate = 0.5 mL/min. Retention times: minor diastereoisomer, 18 min (major) and 26 min (minor); major diastereoisomer, 30 min (major) and 36 min (minor).

#### (2*S*,3*R*)-5-(Benzyloxy)-2-((bis(4-(trifluoromethyl)phenyl)methyl) amino)-3-hydroxy-*N*-(2-nitrophenyl)pentanamide **7g**

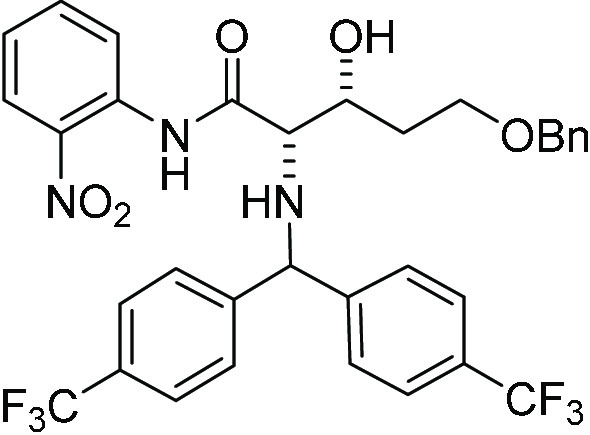



The compound was prepared according to the general procedure starting from 2-((bis(4-(trifluoromethyl)phenyl)methylene)amino)-*N*-(2-nitrophenyl)acetamide **3** (99 mg, 0.2 mmol) and (benzyloxy)acetaldehyde **4i** (84 μL, 0.6 mmol) and was purified by flash column chromatography on silica gel (hexane/EtOAc, 85:15) to afford **7g** as a yellow oil. Yield: 70% (91 mg, 0.14 mmol). [α]_D_^24^ −13.2 (*c* 0.5, 90% ee, CH_2_Cl_2_). ^1^H NMR (300 MHz, CDCl_3_): δ 11.74 (s, 1H), 8.71 (d, *J* = 9.7 Hz, 1H), 8.22 (d, *J* = 9.9 Hz, 1H), 7.64 (s, 5H), 7.50 (d, *J* = 1.6 Hz, 4H), 7.38–7.25 (m, 5H), 7.26–7.13 (m, 1H), 5.08 (s, 1H), 4.47 (s, 2H), 4.37–4.24 (m, 1H), 3.80–3.61 (m, 2H), 3.30 (d, *J* = 4.9 Hz, 1H), 2.06–1.84 (m, 2H). ^13^C{^1^H} NMR (75 MHz, CDCl_3_): δ 172.5, 146.0 (q), 135.8, 134.1, 129.6, 128.7, 128.2, 127.9, 127.8, 126.1, 126.1, 125.9, 125.9, 123.7, 122.1, 73.8, 72.9, 69.2, 66.2, 65.6, 33.2. UPLC-DAD-QTOF, HRMS (ESI) *m*/*z*: [M + H]^+^ calcd for C_33_H_30_F_6_N_3_O_4_, 662.2083; found, 662.2090. The enantiomeric purity was determined by HPLC analysis (Phenomenex-Lux 3 μm i-Amilose-1 (00G-4729-E0)) hexane/isopropanol 95:5, flow rate = 0.5 mL/min. Retention times: minor diastereoisomer, 31 min (major) and 37 min (minor); major diastereoisomer, 37 min (minor) and 45 min (major).

#### (2*S*,3*R*)-2-((Bis(4-(trifluoromethyl)phenyl)methyl)amino)-3-hydroxy-*N*-(2-nitrophenyl)oct-7-enamide **7h**

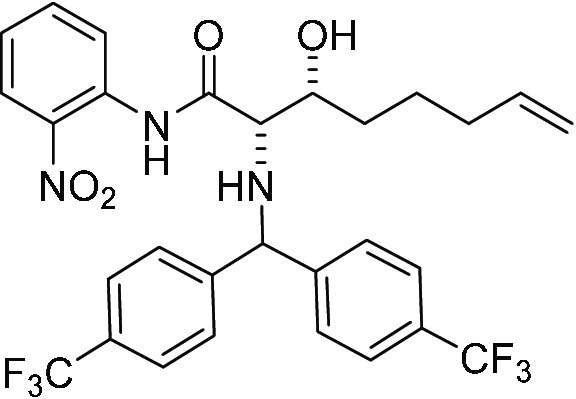



The compound was prepared according to the general procedure starting from 2-((bis(4-(trifluoromethyl)phenyl)methylene)amino)-*N*-(2-nitrophenyl)acetamide **3** (99 mg, 0.2 mmol) and 5-hexenal **4h** (59 mg, 0.6 mmol, 3 equiv) and was isolated by flash column chromatography on silica gel (hexane/EtOAc, 90:10) as a yellow foam. Yield (diastereomeric ratio 97:3): 63% (75 mg, 0.13 mmol). ^1^H NMR (300 MHz, CDCl_3_), major diastereomer: δ 11.77 (s, 1H), 8.75 (d, *J* = 8.5 Hz, 1H), 8.25 (d, *J* = 8.5 Hz, 1H), 7.65 (s, 5H), 7.59–7.45 (m, 4H), 7.32–7.18 (m, 2H), 5.90–5.66 (m, 1H), 5.12–4.92 (m, 3H), 4.11 (s, 1H), 3.24 (d, *J* = 3.6 Hz, 1H), 2.87 (s, 1H), 2.31 (s, 1H), 2.17–2.00 (m, 2H), 1.73–1.50 (m, 1H), 1.51–1.34 (m, 2H), 0.92–0.74 (m, 1H). ^13^C{^1^H} NMR (75 MHz, CDCl_3_), major diastereomer: δ 145.9, 145.5, 138.0, 135.9, 133.9, 130.1 (q), 127.9, 127.5, 126.1, 126.1, 125.9, 123.8, 121.9, 115.2, 72.5, 66.1, 65.1, 33.3, 33.1, 29.7, 25.1. UPLC-DAD-QTOF, HRMS (ESI) *m*/*z*: [M + H]^+^ calcd for C_29_H_28_F_6_N_3_O_4_, 596.1984; found, 596.1989. The enantiomeric purity of the major isomer was determined by chiral HPLC analysis of the crude reaction mixture (Phenomenex i-Cellulose-5, hexane/isopropanol, 98:2; flux = 1 mL/min; retention times for major diastereomer: 12.5 min (major), 37.3 min (minor)).

#### *tert*-Butyl ((2*R*,3*S*)-(3-((Bis(4-(trifluoromethyl)phenyl)methyl) amino)-2-hydroxy-4-((2-nitrophenyl)amino)-4-oxobutyl)carbamate **7i**

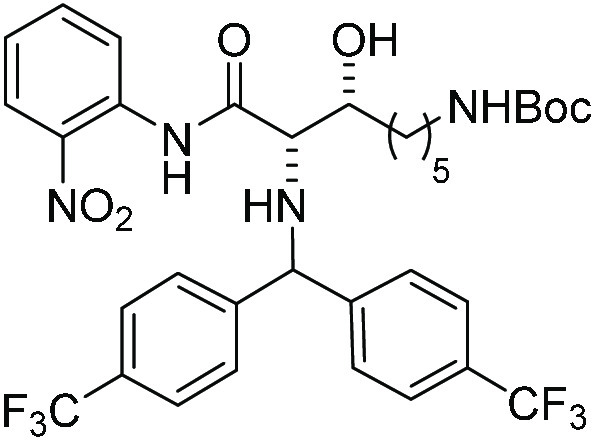



The title compound was prepared according to the general procedure starting from 2-((bis(4-(trifluoromethyl)phenyl) methylene)amino)-*N*-(2-nitrophenyl) acetamide **3** (99 mg, 0.2 mmol) and *tert*-butyl (6-oxohexyl)carbamate **4i** (130 mg, 0.6 mmol) and was purified by flash column chromatography on silica gel (hexane/EtOAc, 80:20) to afford **7i** as a yellow oil. Yield: 63% (88 mg, 0.13 mmol). [α]_D_^24^ −1.8 (*c* 1.5, 88% ee, CH_2_Cl_2_). ^1^H NMR (300 MHz, CDCl_3_): δ 11.77 (s, 1H), 8.73 (d, 1H), 8.22 (d, *J* = 9.8 Hz, 1H), 7.65 (s, 4H), 7.61 (t, *J* = 7.3 Hz, 1H), 7.58–7.47 (m, 4H), 7.20 (t, *J* = 8.4 Hz, 1H), 5.09 (s, 1H), 4.66–4.44 (m, 1H), 4.08 (s, 1H), 3.07 (s, 2H), 1.68–1.55 (m, 3H), 1.41 (s, 9H), 1.36–1.12 (m, 5H). ^13^C{^1^H} NMR (75 MHz, CDCl_3_): δ 172.9, 156.4, 146.2, 145.8, 136.9, 135.9, 135.8, 134.1, 130.3 (q, *J* = 42.7 Hz), 128.1, 127.7, 126.2, 126.1, 125.9, 123.7, 122.0, 72.1, 66.1, 55.7, 40.0, 33.9, 30.2. UPLC-DAD-QTOF, HRMS (ESI) *m*/*z*: [M + H]^+^ calcd for C_34_H_39_F_6_N_4_O_6_, 713.2774; found, 713.2774. The enantiomeric purity was determined by HPLC analysis (Daicel Chiralcel IA hexane/isopropanol 90:10, flow rate = 0.5 mL/min, retention times: 26 min (minor) and 29 min (major).

#### *tert*-Butyl (4*R*,5*S*)-5-((Bis(4-(trifluoromethyl)phenyl)methyl)amino)-4-hydroxy-6-((2-nitrophenyl)amino)-6-oxohexanoate **7j**

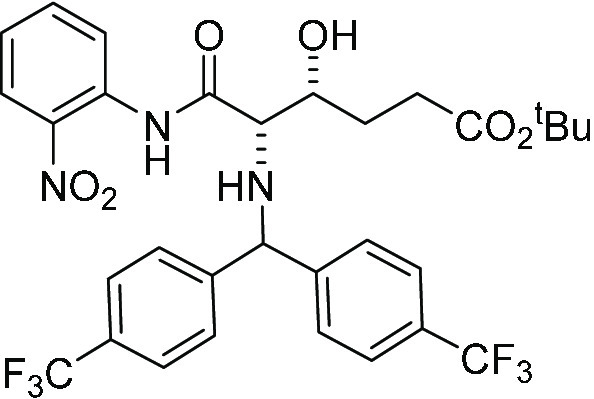



The compound was obtained following the general procedure starting from 2-((bis(4-(trifluoromethyl) phenyl)methylene)amino)-*N*-(2-nitrophenyl)acetamide **3** (99 mg, 0.2 mmol) and *tert*-butyl 4-oxobutanoate **4j** (96 mg, 0.6 mmol, 3 equiv) and was isolated by flash column chromatography on silica gel (hexane/EtOAc, 80:20) as a yellow foam. Yield (diastereomeric ratio 97:3): 63% (75 mg, 0.13 mmol). ^1^H NMR (300 MHz, CDCl_3_), major diastereomer: δ 11.80 (s, 1H), 8.72 (d, *J* = 8.4 Hz, 1H), 8.21 (d, *J* = 8.4 Hz, 1H), 7.70–7.58 (m, 5H), 7.58–7.46 (m, 4H), 7.20 (t, *J* = 7.8 Hz, 1H), 5.12 (s, 1H), 4.12–3.99 (m, 1H), 3.27 (d, *J* = 4.4 Hz, 1H), 2.51–2.41 (m, 2H), 1.94–1.79 (m, 2H), 1.42 (s, 9H). ^13^C{^1^H} NMR (75 MHz, CDCl_3_): δ 173.9, 172.5, 146.0, 145.6, 136.9, 135.7, 133.8, 130.1 (q), 128.0, 127.6, 126.0, 125.9, 125.8, 125.7, 123.7, 121.9, 81.3, 72.3, 65.9, 65.5, 32.3, 28.6, 28.0. UPLC-DAD-QTOF, HRMS (ESI) *m*/*z*: [M + H]^+^ calcd for C_31_H_32_F_6_N_3_O_6_, 656.2195; found, 656.2199. The enantiomeric purity of the major isomer was determined by chiral HPLC analysis of the crude reaction mixture (Phenomenex Amillose-1, hexane/isopropanol, 98:2; flux = 1 mL/min; retention times for major diastereomer: 44.4 min (minor), 62.4 min (major)).

#### (2*S*,3*R*)-2-(Benzhydrylamino)-*N*-(2,4-dinitrophenyl)-3-hydroxy-5-phenylpentanamide **17**

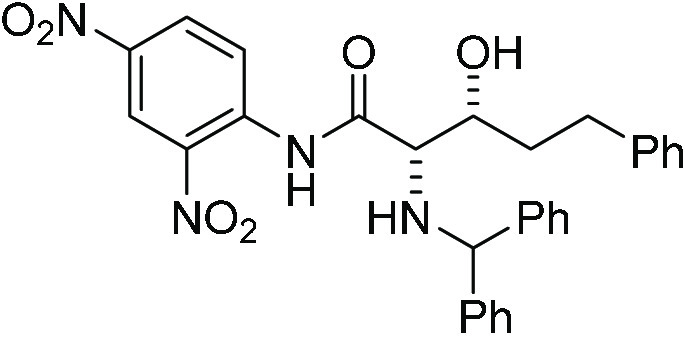



The compound was prepared according to the general procedure starting from *N*-(2,4-dinitrophenyl)-2-((diphenylmethylene) amino) acetamide **16** (81 mg, 0.2 mmol) and hydrocinnamaldehyde **4a** (80 μL, 0.6 mmol) and was isolated by flash column chromatography on silica gel (hexane/EtOAc, 95:5). Yellow oil. Yield (diastereomeric ratio 93:7): 51% (55 mg, 0.1 mmol). ^1^H NMR (300 MHz, CDCl_3_): δ 12.29 (s, 1H), 9.16–9.06 (m, 1H), 9.02 (d, *J* = 9.4 Hz, 1H), 8.46–8.35 (m, 1H), 7.5–7.44 (m, 3H), 7.43–7.07 (m, 12H), 4.93 (s, 1H), 3.39 (d, *J* = 3.6 Hz, 1H), 2.83 (d, *J* = 14.4 Hz, 1H), 2.68 (d, *J* = 11.3 Hz, 1H), 1.99–1.82 (m, 2H). ^13^C{^1^H}NMR (75 MHz, CDCl_3_): δ 174.2, 142.7, 142.0, 141.0, 139.1, 129.9, 129.1, 128.8, 128.8, 128.6, 127.9, 127.5, 127.2, 126.4, 122.2, 72.3, 67.3, 65.4, 35.7, 32.4. UPLC-DAD-QTOF, HRMS (ESI) *m*/*z*: [M + H]^+^ calcd for C_30_H_29_N_4_O_6_, 541.2087; found, 541.2076. The enantiomeric purity was determined by HPLC analysis (Daicel Chiralcel IF hexane/ethanol 90/10, flow rate = 1.0 mL/min, retention times: 16 min (minor) and 17 min (major)).

### General Procedure for the Racemic Reactions of Schiff Bases of Glycine Nitroanilide

The corresponding nitroanilide (0.2 mmol, 1 equiv) and 1,3-bis(3,5-bis(trifluoromethyl)phenyl)thiourea (0.02 mmol, 20 mol %) were dissolved in dry dichloromethane (0.5 mL) at room temperature. To the mixture was added Et_3_N (0.02 mmol, 20 mol %), followed by the corresponding aldehyde (0.6 mmol, 3 equiv). The reaction mixture was stirred at room temperature until consumption of the starting material (monitored by ^1^H NMR). To the reaction mixture was added MeOH (0.4 mL), followed by NaBH_3_CN (32 mg, 0.5 mmol, 2.5 equiv) and AcOH (24 μL, 0.4 mmol, 2 equiv). The reaction mixture was stirred for 2 h (the reduction of the imine can be followed by ^1^H NMR). The solvents were evaporated under reduced pressure, and the resulting residue was redissolved in dichloromethane and washed with a saturated NaHCO_3_ solution (1 × 4 mL). The organic phase was dried over MgSO_4_ and evaporated *in vacuo*. The crude was purified by flash column chromatography on silica gel.

### General Procedure for the Aldol Reaction of α-Pyridyl and Phenyl Acetanilides

To a solution of the corresponding acetanilide (0.2 mmol, 1 equiv) and Et_3_N (0.02 mmol, 20 mol %) in dry dichloromethane (0.5 mL) was added freshly distilled hydrocinnamaldehyde (80 μL, 0.6 mmol, 3 equiv) (previously washed with a saturated NaHCO_3_ solution) at room temperature. The reaction mixture was stirred at the same temperature until consumption of the starting material (followed by ^1^H NMR). To the reaction mixture was added pyridine/acetic anhydride (2:1, 0.4:0.2 mmol), and the resulting mixture was stirred at room temperature overnight. The mixture was then washed with 1 M HCl (1 × 4 mL) and saturated NaHCO_3_ solution (1 × 4 mL). The organic phase was dried over MgSO_4_ and evaporated *in vacuo*.

#### 1-((2-Nitrophenyl)amino)-1-oxo-5-phenyl-2-(pyridin-2-yl)pentan-3-yl acetate **22**

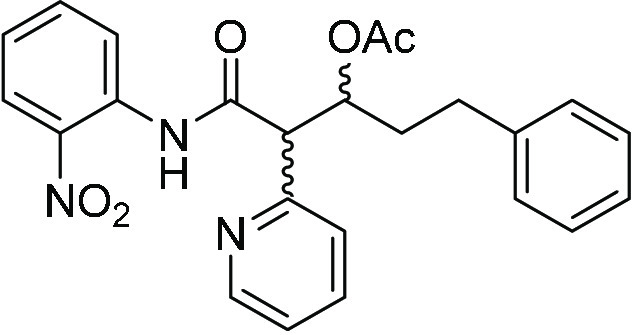



The compound was prepared according to the general procedure starting from *N*-(2-nitrophenyl)-2-(pyridin-2-yl)acetamide **18** (51 mg, 0.2 mmol) and was purified by flash column chromatography on silica gel (hexane/EtOAc, 80:20) to afford the title product as a yellow oil and as a 50:50 mixture of diastereoisomers. Data for the mixture of the diastereoisomers follows: Yield: 64% (55 mg, 0.13 mmol.). ^1^H NMR (300 MHz, chloroform-*d*): δ 11.72 (d, *J* = 13.0 Hz, 1H), 8.85–8.71 (m, 1H), 8.71–8.54 (m, 1H), 8.32–8.07 (m, 1H), 7.80–7.55 (m, 3H), 7.25 (dt, *J* = 6.6, 1.5 Hz, 2H), 7.22–7.14 (m, 3H), 7.13–7.08 (m, 1H), 5.95–5.66 (m, 1H), 4.24–3.95 (m, 1H), 3.02–2.47 (m, 4H), 2.08 (s, 3H). ^13^C{^1^H} NMR (75 MHz, CDCl_3_): δ 171.3, 170.3, 150.4, 149.9, 145.3, 144.6, 138.7, 136.6, 129.3, 129.3, 127.1, 127.0, 126.6, 125.3, 124.4, 124.2, 123.8, 123.6, 122.6, 75.9, 61.8, 36.5, 35.3, 32.4, 22.3. UPLC-DAD-QTOF, HRMS (ESI) *m*/*z*: [M + H]^+^ calcd for C_24_H_23_N_3_O_5_, 433.1638; found, 433.1636.

#### 1-((4-Nitrophenyl)amino)-1-oxo-5-phenyl-2-(pyridin-2-yl)pentan-3-yl acetate **23**


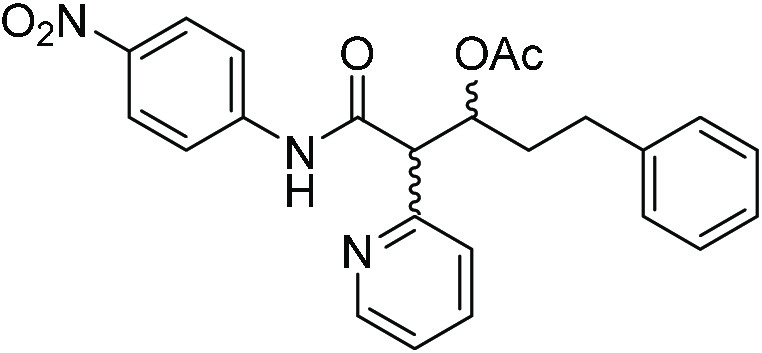


The general procedure was followed starting from *N*-(4-nitrophenyl)-2-(pyridin-2-yl)acetamide **19** (51 mg, 0.2 mmol). After 48 h at rt, the formation of the aldols as a 50:50 mixture of diastereoisomers was observed (50% conversion). The aldols were not isolated. See the ^1^H NMR spectrum of the crude in the Supporting Information.

### 3-Hydroxy-*N*,5-diphenyl-2-(pyridin-2-yl)pentanamide **24**

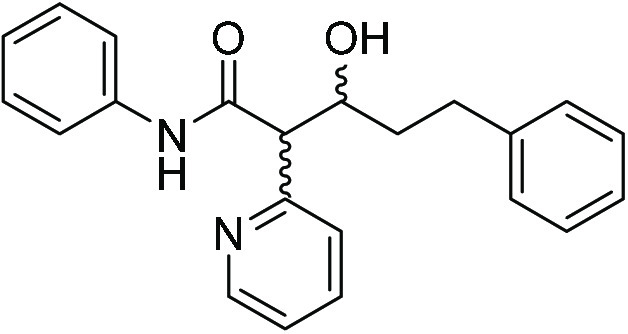



The general procedure was followed starting from *N*-phenyl-2-(pyridin-2-yl)acetamide **20** (42 mg, 0.2 mmol). After 48 h at rt, the formation of a 50:50 diastereomeric mixture of the aldols was observed (33% conversion). The aldols were not isolated. See the ^1^H NMR spectrum of an aliquot in the Supporting Information.

### General Procedure for the Control Experiments

The corresponding compounds **13**–**15** (0.2 mmol, 1 equiv) and the corresponding catalyst (0.02 mmol, 20 mol %) were dissolved in dry dichloromethane (0.5 mL) at room temperature, and hydrocinnamaldehyde (0.6 mmol, 3 equiv) was added. When reaction was observed, the work-up described in the general procedure for the asymmetric aldol reactions was followed.

### Stability of the Reaction Adducts

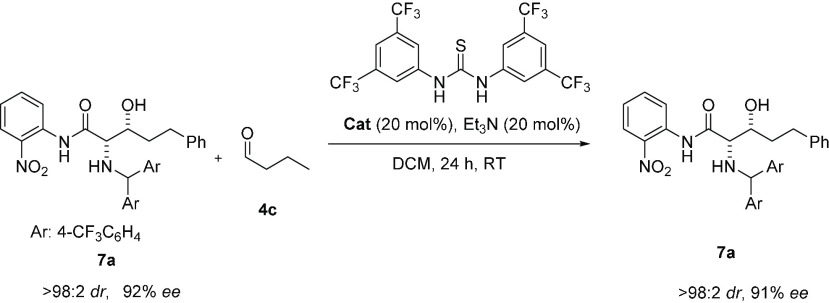



The isolated product **7a** (0.1 mmol, 1 equiv) was dissolved in dry dichloromethane (0.3 mL), and butyraldehyde **4c** (0.3 mmol, 3 equiv) was added, followed by the achiral catalyst (10 mg, 0.02 mmol, 20 mol %) and triethylamine (3 μL, 0.02 mmol, 20 mol %). The reaction mixture was stirred at room temperature for 24 h. The crude was purified by flash column chromatography on silica gel.

### Elaboration of Adducts

#### Imine Hydrolysis and Amine Protection: Synthesis of **10**

##### (2*S*,3*R*)-2-Amino-3-hydroxy-*N*-(2-nitrophenyl)-5-phenylpentanamide **9**

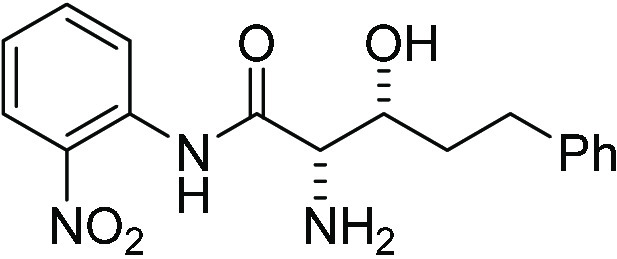



The aldol adduct, previous to reduction, (0.2 mmol, 1 equiv) was dissolved in THF (5 mL), and 1 M HCl (0.68 mL, 0.68 mmol, 3.4 equiv) was added at 0 °C. The mixture was stirred at the same temperature for 2 h. Then, after reaction completion (monitored by ^1^H NMR), the solvent was evaporated under reduced pressure, and NaHCO_3_ (sat) was added until pH 8–9. The mixture was extracted with Cl_2_CH_2_ (3 × 10 mL), and brine (10 mL) was added to the aqueous phase, which was extracted again with Cl_2_CH_2_ (3 × 5 mL). The combined organic layers were dried over MgSO_4_ and evaporated under reduced pressure. The crude was used in the next step without further purification. Yield: 80% (52 mg, 0.16 mmol). [α]_D_^23^ −8.1 (*c* 2, 94% ee, CH_2_Cl_2_). ^1^H NMR (300 MHz, CDCl_3_): δ 12.06 (s, 1H), 8.79 (dd, *J* = 8.5 Hz, 1H), 8.18 (dd, 1H), 7.63 (td, *J* = 7.6 Hz, 1H), 7.46–7.01 (m, 5H), 4.41–4.22 (m, 1H), 3.46 (d, *J* = 2.5 Hz, 1H), 2.95–2.83 (m, 1H), 2.83–2.61 (m, 1H), 2.01–1.72 (m, 2H). ^13^C{^1^H} NMR (75 MHz, CDCl_3_): δ 173.8, 141.6, 135.7, 134.2, 128.7, 128.6, 126.3, 125.9, 123.6, 122.2, 71.3, 60.0, 35.4, 32.4. UPLC-DAD-QTOF, HRMS (ESI) *m*/*z*: [M + H]^+^ calcd for C_17_H_20_N_3_O_4_, 330.1454; found, 330.1462.

##### 4-Bromo-*N*-((2*S*,3*R*)-3-hydroxy-1-((2-nitrophenyl)amino)-1-oxo-5-phenylpentan-2-yl)benzamide **10**

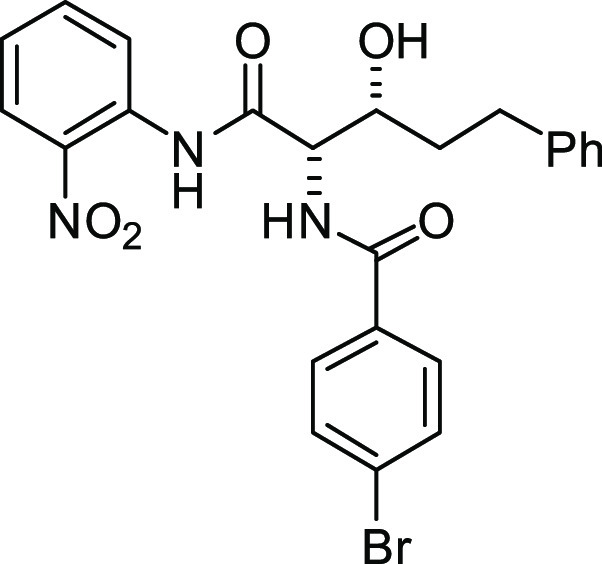



Aminoalcohol **9** (0.2 mmol, 1 equiv) was dissolved in dry THF (1 mL), and 4-bromobenzoyl chloride (0.2 mmol, 1 equiv) was added in one portion, followed by slow addition of triethylamine (0.65 mL, 4.6 mmol, 23 equiv). The reaction mixture was stirred at room temperature for 2 h until complete conversion of the starting material. Then the solvent was evaporated, and the residue was redissolved in dichloromethane, washed with water, and extracted with dichloromethane (2 × 5 mL). The combined organic phases were dried over MgSO_4_ and evaporated *in vacuo*. The crude was purified by flash column chromatography on silica gel (hexane/EtOAc, 90:10) and afforded **10** as a white solid. Yield: 75% (76 mg, 0.15 mmol). Mp: 145–147 °C. [α]_D_^23^ −7.4 (*c* 2, 92% ee, CH_2_Cl_2_). ^1^H NMR (300 MHz, CDCl_3_): δ 11.07 (s, 1H), 8.66 (dd, 1H), 8.16 (dd, *J* = 8.4, 1.4 Hz, 1H), 7.77 (d, *J* = 8.5 Hz, 2H), 7.59 (d, *J* = 8.5 Hz, 2H), 7.38–7.12 (m, 6H), 4.92 (d, *J* = 9.7 Hz, 1H), 4.54–4.39 (m, 1H), 2.86 (s, 1H), 2.78–2.60 (m, 1H), 1.86 (q, *J* = 8.4 Hz, 2H). ^13^C{^1^H} NMR (75 MHz, CDCl_3_): δ 170.1, 167.5, 141.1, 137.2, 135.8, 133.7, 132.2, 129.1, 128.7, 128.5, 127.3, 126.3, 125.9, 124.1, 122.6, 70.3, 58.7, 34.8, 32.1. UPLC-DAD-QTOF, HRMS (ESI) *m*/*z*: [M + H]^+^ calcd for C_24_H_23_BrN_3_O_5_, 512.0826; found, 512.0821.

#### Anilide Cleavage.^49^

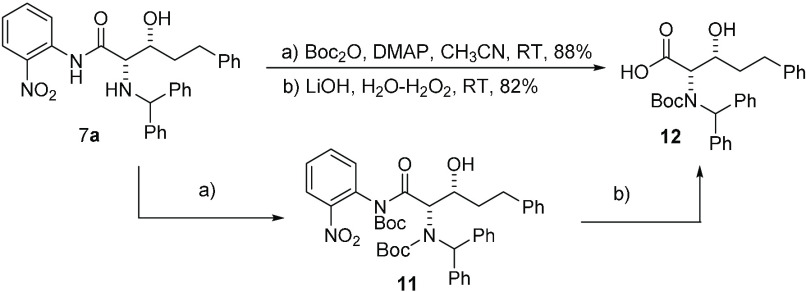



Compound **7a** (0.2 mmol) was dissolved in dry acetonitrile (0.3 mL), and DMAP (8 mg, 0.06 mmol, 30 mol %) was added, followed by di-*tert*-butyl dicarbonate (280 mg, 1.2 mmol, 6 equiv). The solution was stirred at room temperature for 16 h. Then, the solvent was evaporated under reduced pressure, and the resulting residue was purified by flash column chromatography on silica gel (hexane/EtOAc, 95:5) to afford the crude product **11** as a yellow oil. Yield: 88% (122 mg, 0.18 mmol). [α]_D_^23^ −7.9 (*c* 2, 92% ee, CH_2_Cl_2_). ^1^H NMR (300 MHz, CDCl_3_): δ 8.14 (d, *J* = 8.0 Hz, 1H), 7.60 (d, *J* = 7.3 Hz, 2H), 7.56–7.48 (m, 1H), 7.47–7.39 (m, 2H), 7.37–7.07 (m, 13H), 5.25 (t, 1H), 4.73 (s, 1H), 4.68 (s, 1H), 2.79–2.66 (m, 1H), 2.62–2.44 (m, 2H), 2.24–1.98 (m, 1H), 1.41 (s, 9H), 1.19 (s, 9H). ^13^C{^1^H} NMR (75 MHz, CDCl_3_): δ 176.0, 153.8, 150.6, 145.9, 144.5, 143.1, 141.6, 134.0, 131.7, 129.1, 128.6, 128.5, 127.3, 127.0, 126.0, 125.2, 84.9, 82.3, 76.9, 65.4, 62.2, 33.5, 31.8, 27.9, 27.5. UPLC-DAD-QTOF, HRMS (ESI) *m*/*z*: [M + H]^+^ calcd for C_40_H_46_N_3_O_8_, 696.3285; found, 696.3285.

The previous crude product **11** (139 mg, 0.2 mmol) was dissolved in THF/H_2_O (3:1, 2 mL). Then, LiOH·H_2_O (9 mg, 0.4 mmol, 2 equiv) and 30% H_2_O_2_ (22 μL, 1 mmol, 5 equiv) were added at 0 °C. The reaction mixture was stirred at room temperature for 48 h, and Na_2_SO_3_ (252 mg, 2 mmol, 10 equiv) was added. The mixture was then diluted with EtOAc, acidified with 0.5 M HCl, and extracted with EtOAc (3 × 10 mL). The organic layer was dried over anhydrous Na_2_SO_4_ and concentrated *in vacuo*. The resulting residue was purified by flash column chromatography (hexane/EtOAc, 80:20) to afford **12** as a yellow oil. Yield: 82% (77 mg, 0.16 mmol). [α]_D_^23^ −9.1 (*c* 1, 91% ee, CH_2_Cl_2_). ^1^H NMR (300 MHz, CDCl_3_): δ 7.48–7.34 (m, 3H), 7.33–6.85 (m, 12H), 5.12–5.02 (m, 1H), 4.88 (s, 1H), 3.33 (d, *J* = 3.1 Hz, 1H), 2.76–2.58 (m, 1H), 2.58–2.45 (m, 1H), 2.32–2.15 (m, 1H), 2.14–1.90 (m, 1H), 1.46 (s, 9H). ^13^C{^1^H} NMR (75 MHz, CDCl_3_): δ 176.2, 153.1, 143.4, 142.3, 141.0, 128.9, 128.8, 128.6, 128.5, 127.8, 127.6, 127.3, 126.6, 126.3, 82.8, 65.69, 61.0, 33.3, 31.8, 29.9, 27.8. UPLC-DAD-QTOF, HRMS (ESI) *m*/*z*: [M + H]^+^ calcd for C_29_H_34_NO_5_, 476.2437; found, 476.2437.

### X-ray Crystallography

Crystals suitable for X-ray crystallography were obtained by crystallization of **1** from Cl_2_CH_2_, of **3** from Et_2_O/CHCl_3_, and of **10** from CH_3_CN. Each sample was dissolved in the minimum amount of the indicated solvent at rt and was allowed to crystallize slowly at the same temperature. Intensity data were collected on an Agilent Technologies Super-Nova diffractometer, which was equipped with monochromated Cu Kα radiation (λ = 1.54184 Å) and Atlas CCD detector. Measurements were carried out at 150.01(10) K with the help of an Oxford Cryostream 700 PLUS temperature device. Data frames were processed (united cell determination, analytical absorption correction with face indexing, intensity data integration, and correction for Lorentz and polarization effects) using the Crysalis software package.^50^ The structure was solved using SHELXT^51^ and refined by full-matrix least-squares with SHELXL-97.^51,52^ Final geometrical calculations were carried out with Mercury^53^ and PLATON^54^ as integrated in WinGX.^55^ Complete structural data have been deposited with the Cambridge Crystallographic Data Centre.^29,31^

## Supporting Information Available

Catalyst screening data; X-ray diffraction data for **1**, **3**, and **10**; computational data for the *E* and *Z* enolates of ketimine **3**; spectroscopic data for all new compounds including ^1^H and ^13^C NMR spectra; HPLC chromatograms of all the aldol adducts (PDF)

## Accession Codes

CCDC 1977378, 1977381, and 2064298 contain the supplementary crystallographic data for this paper. These data can be obtained free of charge via www.ccdc.cam.ac.uk/data_request/cif, or by emailing data_request@ccdc.cam.ac.uk, or by contacting The Cambridge Crystallographic Data Centre, 12 Union Road, Cambridge CB2 1EZ, UK; fax: +44 1223 336033.

## References

[ref1] aHughes A. B., Ed. Amino Acids, Peptides and Proteins in Organic Chemistry: Building Blocks, Catalysis and Coupling Chemistry; Wiley-VCH, 2011.

[ref2] aNájera C.; Sansano J. M. Catalytic Asymmetric Synthesis of α-Amino Acids. Chem. Rev. 2007, 107, 4584–4671. 10.1021/cr050580o. 17915933

[ref3] aO’Donnell M. J.; Eckrich T. M. The Synthesis of Amino Acid Derivatives by Catalytic Phase-Transfer Alkylations. Tetrahedron Lett. 1978, 19, 4625–4628. 10.1016/S0040-4039(01)85688-4.

[ref4] Gasparski C. M.; Miller M. J. Synthesis of β-Hydroxy α-Amino Acids by Aldol Condensation Using a Chiral Phase Transfer Catalyst. Tetrahedron 1991, 47, 5367–5378. 10.1016/S0040-4020(01)80971-6.

[ref5] aOoi T.; Taniguchi M.; Kameda M.; Maruoka K. Direct Asymmetric Aldol Reactions of Glycine Schiff Base with Aldehydes Catalyzed by Chiral Quaternary Ammonium Salts. Angew. Chem., Int. Ed. 2002, 41, 4542–4544. 10.1002/1521-3773(20021202)41:23<4542::AID-ANIE4542>3.0.CO;2-3. 12458532

[ref6] Yoshikawa N.; Shibasaki M. Catalytic Asymmetric Synthesis of β-Hydroxy α-Amino Acid Esters by Direct Aldol Reaction of Glycinate Schiff Bases. Tetrahedron 2002, 58, 8289–8298. 10.1016/S0040-4020(02)00979-1.

[ref7] MacMillan J. B.; Molinski T. F. Lobocyclamide B from Lyngbya confervoides. Configuration and Asymmetric Synthesis of β-Hydroxy α-Amino Acids by (−)-Sparteine-Mediated Aldol Addition. Org. Lett. 2002, 4, 1883–1886. 10.1021/ol025876k. 12027638

[ref8] aTrost M. B.; Miege F. Development of ProPhenol Ligands for the Diastereo- and Enantioselective Synthesis of β-Hydroxy α-Amino Esters. J. Am. Chem. Soc. 2014, 136, 3016–3019. 10.1021/ja4129394. 24502188 PMC3985890

[ref9] Timofeeva D. S.; Ofial A. R.; Mayr H. Nucleophilic Reactivities of Schiff Base Derivatives of Amino Acids. Tetrahedron 2019, 75, 459–463. 10.1016/j.tet.2018.11.075.

[ref10] aWang Y.; Song X.; Wang J.; Moriwaki H.; Soloshonok V. A.; Liu H. Recent Approaches for Asymmetric Synthesis of α-Amino Acids via Homologation of Ni(II) Complexes. Amino Acids 2017, 49, 1487–1520. 10.1007/s00726-017-2458-6. 28674862

[ref11] aMaruoka K.; Ooi T. Enantioselective Amino Acid Synthesis by Chiral Phase-Transfer Catalysis. Chem. Rev. 2003, 103, 3013–3028. 10.1021/cr020020e. 12914490

[ref12] aMaruoka K., Ed. Asymmetric Organocatalysis 1: Brønsted Base and Acid Catalysts and Additional Topics; Thieme: Stuttgart, 2012.

[ref13] Xue M.-X.; Zhang X.-M.; Gong L.-Z. The First Organocatalytic Enantio- and Diastereoselective 1,3-Dipolar Cyloaddition of Azomethine Ylides with Nitroalkenes. Synlett 2008, 2008, 691–694. 10.1055/s-2008-1042802.

[ref14] aTian S.-K.; Chen Y.; Hang J.; Tang L.; McDaid P.; Deng L. Asymmetric Organic Catalysis with Modified Cinchona Alkaloids. Acc. Chem. Res. 2004, 37, 621–631. 10.1021/ar030048s. 15311961

[ref15] aMatsumoto M.; Harada M.; Yamashita Y.; Kobayashi S. Catalytic Imine-Imine Cross-Coupling Reactions. Chem. Commun. 2014, 50, 13041–13044. 10.1039/C4CC06156J. 25227870

[ref16] aGuerrero-Corella A.; Esteban F.; Iniesta M.; Martín-Somer A.; Parra M.; Díaz-Tendero S.; Fraile A.; Alemán J. 2-Hydroxybenzophenone as a Chemical Auxiliary for the Activation of ketiminoesters for Highly Enantioselective Addition to Nitroalkenes under Bifunctional Catalysis. Angew. Chem., Int. Ed. 2018, 57, 5350–5354. 10.1002/anie.201800435. PMC5947601 29493860

[ref17] Wen W.; Chen L.; Luo M.-J.; Zhang Y.; Chen Y.-C.; Ouyang Q.; Guo Q.-X. Chiral Aldehyde Catalysis for the Catalytic Asymmetric Activation of Glycine Esters. J. Am. Chem. Soc. 2018, 140, 9774–9780. 10.1021/jacs.8b06676. 29995401

[ref18] aSkulski L. The Ultraviolet and Infrarred Spectra of Some o-Nitroamides. J. Org. Chem. 1963, 28, 3565–3567. 10.1021/jo01047a513.

[ref19] Darvesh S.; McDonald R. S.; Darvesh K. V.; Mataija D.; Mothana S.; Cook H.; Carneiro K. M.; Richard N.; Walsh R.; Martin E. On the Active Site for Hydrolisis of Aryl Amides and Choline Esters by Human Cholinesterases. Bioorg. Med. Chem. 2006, 14, 4586–4599. 10.1016/j.bmc.2006.02.021. 16504521

[ref20] Steiner T. The Hydrogen Bond in the Solid State. Angew. Chem., Int. Ed. 2002, 41, 48–76. 10.1002/1521-3773(20020104)41:1<48::AID-ANIE48>3.0.CO;2-U. 12491444

[ref21] aTan B.; Hernández-Torres G.; Barbas III C. F. Rationally Designed Amide Donors for Organocatalytic Asymmetric Michael Reactions. Angew. Chem., Int. Ed. 2012, 51, 5381–5385. 10.1002/anie.201200996. 22505348

[ref22] For preliminary results on the synthesis of pronucleophile **1** and catalyst screening of the reaction of **1** with **4a** see: Sandra del Pozo, “Asymmetric α-Functionalization of Barbituric Acids via Brønsted Base Catalysis”, Ph.D. Dissertation, University of the Basque Country, UPV/EHU, 2019. Also see the Supporting Information for further details.

[ref23] aMalerich J. P.; Hagihara K.; Rawal V. R. Chiral Squaramide Derivatives are Excellent Hydrogen Bond Donor Catalysts. J. Am. Chem. Soc. 2008, 130, 14416–14417. 10.1021/ja805693p. 18847268 PMC2701638

[ref24] aFang X.; Wang C.-J. Recent Advances in Asymmetric Organocatalysis Mediated by Bifunctional Amine-Thioureas Bearing Multiple Hydrogen-Bonding Donors. Chem. Commun. 2015, 51, 1185–1197. 10.1039/C4CC07909D. 25364797

[ref25] aDiosdado S.; Etxabe J.; Izquierdo J.; Landa A.; Mielgo A.; Olaizola I.; López R.; Palomo C. Catalytic Enantioselective Synthesis of Tertiary Thiols From 5*H*-Thiazol-4-ones and Nitroolefins: Bifunctional Ureidopeptide-Based Brønsted Base Catalysis. Angew. Chem., Int. Ed. 2013, 52, 11846–11851. 10.1002/anie.201305644. 24105886

[ref26] Two different experiments carried out by treatment of two diastereomeric mixtures of adduct **7a** obtained from the reaction of **3** with the aliphatic aldehyde **4a** in a 98:2 and 80:20 ratio with catalyst **C6** for 24 h at rt revealed no change in the diastereomeric ratio and the absence of retroaldol reaction in both cases.

[ref27] The reaction of **3** with benzaldehyde was also checked, but in this case, isolation of the corresponding diastereomeric aldols was not possible. Retroaldol reaction and/or elimination, as it has been described for analogous reactions involving aromatic aldehydes (see ref (8b)), could probably be the main reason.

[ref28] No self-aldol addition was produced upon exposure of aldehyde **4a** to the Brønsted base **C6** (20 mol%) in dichloromethane at room temperature overnight.

[ref29] CCDC 1977381 and CCDC 1977378 contain the supplementary crystallographic data for compounds **1** and **10**, respectively. These data can be obtained free of charge from The Cambridge Crystallographic Data Centre.

[ref30] aFlynn D. L.; Zelle R. E.; Grieco P. A. A Mild Two-Step Method for the Hydrolysis/Methanolysis of Secondary Amides and Lactams. J. Org. Chem. 1983, 48, 2424–2426. 10.1021/jo00162a028.

[ref31] The X-ray analysis of **3** showed the same H-bonding network than **1**. For more details, see the Supporting Information. CCDC 2064298 contains the supplementary crystallographic data for **3**. These data can be obtained free of charge from The Cambridge Crystallographic Data Centre.

[ref32] Lipkowitz K. B.; Cavanaugh M. W.; Baker B.; O’Donnell M. J. Theoretical Studies in Molecular recognition: Asymmetric Induction of Benzophenone Imine Ester Enolates by the Benzylcinchoninium Ion. J. Org. Chem. 1991, 56, 5181–5192. 10.1021/jo00017a035.

[ref33] aParr R. G.; Yang W. Density-Functional Theory of Atoms and Molecules; Oxford, New York, 1989.

[ref34] Armanego W. L. F.; Perrin D. D. Purification of Laboratory Chemicals, 3rd ed.; Butterworth-Heinemann: Oxford, 1988.

[ref35] aSchläger T.; Schepmann D.; Wünsch B. Novel σ1 Receptor Ligands by Oxa-Pictet-Spengler Reaction of Pyrazolylethanol. Synthesis 2011, 24, 3965–3974.

[ref36] Yang W.; Du D.-M. Chiral Squaramide-Catalyzed Highly Enantioselective Michael Addition of 2-Hydroxy-1,4-Napphthoquinones to Nitroalkenes. Adv. Synth. Catal. 2011, 353, 1241–1246. 10.1002/adsc.201000981.

[ref37] Hu K.; Lu A.; Wang Y.; Zhou Z.; Tang C. Chiral Bifunctional Squaramide Catalyzed Asymmetric Tandem Michael-Cyclization Reaction: Efficient Synthesis of Optically Active 2-Amino-4*H*-Chromene-3-Carbonitrile Derivatives. Tetrahedron: Asymmetry 2013, 24, 953–957. 10.1016/j.tetasy.2013.07.010.

[ref38] Kurimoto Y.; Nasu T.; Fujii Y.; Asano K.; Matsubara S. Asymmetric Cycloetherification of in situ Generated Cyanohydrins through the Concomitant Construction of Three Chiral Carbon Centers. Org. Lett. 2019, 21, 2156–2160. 10.1021/acs.orglett.9b00462. 30869909

[ref39] Jiao L.; Zhao X.; Liu H.; Ye X.; Li Y.; Jiang Z. Organocatalytic Asymmetric Conjugate Addition of Diaryloxazolidin-2,4-diones to Nitroolefins: An Efficient Approach to Chiral α-Aryl-α-Hydroxy Carboxylic Acids. Org. Chem. Front. 2016, 3, 470–474. 10.1039/C5QO00428D.

[ref40] Lan P.; Porco J. A.; South M. S.; Parlow J. J. The Development of a Chromatography-Free Mitsunobu Reaction: Synthesis and Applications of an Anthracene-Tagged Phosphine Reagent. J. Comb. Chem. 2003, 5, 660–669. 10.1021/cc030028h.

[ref41] González-Sabin J.; Gotor V.; Rebolledo F. Chemoenzimatic Preparation of Optically Active *trans*-Cyclohexane-1,2-Diamine Derivatives: An Efficient Synthesis of the Analgesic U-(−)-50,488. Chem. - Eur. J. 2004, 10, 5788–5794. 10.1002/chem.200400607. 15472932

[ref42] aPintér Á.; Haberhauer G.; Hyla-Kryspin I.; Grimme S. Configurationally Stable Propeller-Like Triarylphosphine and Triarylphospohine Oxide. Chem. Commun. 2007, 3711–3713. 10.1039/b709655k. 17851603

[ref43] Rijkers D. T. S.; Adams H. P. H. M.; Hemker H. C.; Tesser G. I. A Convenient Synthesis of Amino Acid *p*-Nitroanilides; Synthons in the Synthesis of Protease Substrates. Tetrahedron 1995, 51, 11235–11250. 10.1016/0040-4020(95)00671-T.

[ref44] Soares P.; Lucas X.; Ciulli A. Thiomide Substitution to Probe the Hydroxyproline Recognition of VHL Ligands. Bioorg. Med. Chem. 2018, 26, 2992–2995. 10.1016/j.bmc.2018.03.034. 29650462 PMC6008493

[ref45] Himbert G.; Fink D.; Diehl K.; Rademacher P.; Bittner A. J. Einflu von Substituenten in *p*-, *m*- und *o*-Position am Aromaten auf die Intramolekulare Diels-Alder-Reaktion von Allencarbonsäure-aniliden und -Phenylestern. Chem. Ber. 1989, 122, 1161–1173. 10.1002/cber.19891220623.

[ref46] Respondek T.; Cueny E.; Kodanko J. J. Cumyl Ester as the C-Terminal Protecting Group in the Enantioselective Alkylation of Glycine Benzophenone Imine. Org. Lett. 2012, 14, 150–153. 10.1021/ol202939g. 22149572

[ref47] Nun P.; Prez V.; Calmès M.; Martinez J.; Lamaty F. Preparation of Chiral Amino Esters by Asymmetric Phase-Transfer Catalyzed Alkylations of Schiff Bases in a Ball Mill. Chem. - Eur. J. 2012, 18, 3773–3779. 10.1002/chem.201102885. 22322525

[ref48] Chen Z.; Liang P.; Xu F.; Deng Z.; Long L.; Luo G.; Ye M. Metal-Free Aminothiation of Alkynes: Three-Component Tandem Annulation toward Indolizine Thiones from 2-Alkylpyridines, Ynals, and Elemental Sulfur. J. Org. Chem. 2019, 84, 12639–12647. 10.1021/acs.joc.9b01802. 31545050

[ref49] aZhang S.-Y.; Li Q.; He G.; Nack W. A.; Chen G. Stereoselective Synthesis of β-Alkylated α-Amino Acids via Palladium-Catalyzed Alkylation of Unactivated Methylene C(sp3)-H Bonds with Primary Alkyl Halides. J. Am. Chem. Soc. 2013, 135, 12135–12141. 10.1021/ja406484v. 23919290

[ref50] CrysAlisPro, Version 1.171.37.31; Agilent Technologies, 2014.

[ref51] Sheldrick G. M. SHELXT-Integrated Space-Group and Crystal Structure Determination. Acta Crystallogr., Sect. A: Found. Adv. 2015, A71, 3–8. 10.1107/S2053273314026370. PMC4283466 25537383

[ref52] Sheldrick G. M. A Short History of SHELX. Acta Crystallogr., Sect. A: Found. Crystallogr. 2008, A64, 112–122. 10.1107/S0108767307043930. 18156677

[ref53] Macrae C. F.; Bruno I. J.; Chisholm J. A.; Edgington P. R.; McCabe P.; Pidcock E.; Rodriguez-Monge L.; Taylor R.; van de Streek J.; Wood P. A. Mercury CSD 2.0-New Features For The Visualization and Investigation of Crystal Structures. J. Appl. Crystallogr. 2008, 41, 466–470. 10.1107/S0021889807067908.

[ref54] aSpek A. L. PLATON, A Multipurpose Crystallographic Tool; Utrecht University Utrecht: The Netherlands, 2010.

[ref55] Farrugia L. J. WinGX Suite For Small-Molecule Single-Crystal Crystallography. J. Appl. Crystallogr. 1999, 32, 837–838. 10.1107/S0021889899006020.

