# Supplementary material for: Synthesis of β-Hydroxy α-Amino Acids Through Brønsted Base-Catalyzed syn-Selective Direct Aldol Reaction of Schiff Bases of Glycine o-Nitroanilide
Source: J Org Chem. 2021 May 16;86(11):7757–72. doi: 10.1021/acs.joc.1c00406 (PMC9490875; doi:10.1021/acs.joc.1c00406)
Supplement: Supplementary file 1 — jo1c00406_si_001.pdf [file jo1c00406_si_001.pdf]

SUPPORTING INFORMATION

Synthesis of β-Hydroxy α-Amino Acids Through Brønsted Base Catalyzed syn-Selective Direct Aldol Reaction of Schiff Bases of Glycine o-Nitroanilide

Silvia Vera,[a] Ana Vázquez,[a] Ricardo Rodríguez,[a] Sandra del Pozo,[a] Iñaki Urruzuno,[a] Abel de Cózar,[a,b] Antonia Mielgo* [a] and Claudio Palomo*[a]

[a]Departamento de Química Orgánica I, Facultad de Química, Universidad del País Vasco UPV/EHU, Apdo. 1072, 20080 San Sebastián, Spain.

[b] IKERBASQUE, Basque Foundation for Science 48009 Bilbao (Spain)

*antonia.mielgo@ehu.es

*claudio.palomo@ehu.es

Table of Contents

1. Catalyst screening S2

2. Formation of 8a through cyclization S4

3. Enantioselectivity before and after reductive work-up S5

4. X-Ray analysis S6

4. Computational analysis S7

4.1. Computational methods S7

4.2. Complete reference 33b S7

4.3. Energies, thermal corrections and cartesian coordinates of all computed stationary points S8

5. NMR spectra and HPLC chromatograms S9

1. Catalyst screening

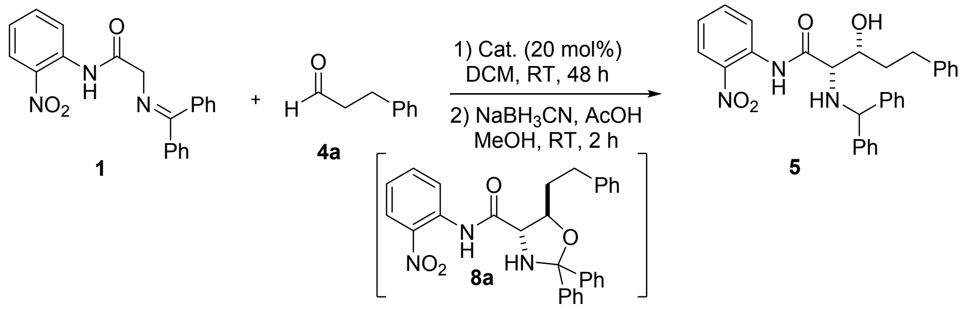

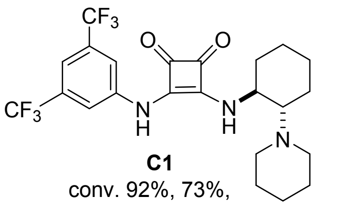

C1
conv. 92%, 73%,
75:25 *dr*, 34(83)% *ee*

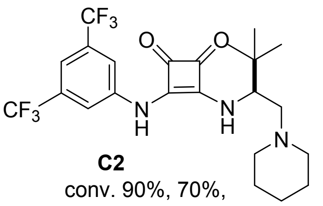

C2
conv. 90%, 70%,
75:25 *dr*, 38(93)% *ee*

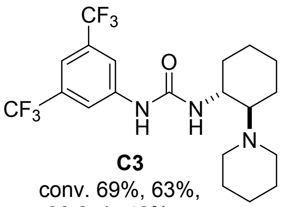

C3
conv. 69%, 63%,
>98:2 *dr*, 40% *ee*

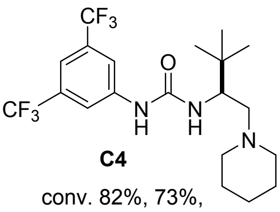

C4
conv. 82%, 73%,
95:5 *dr*, 36% *ee*

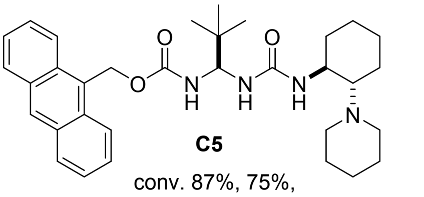

C5
conv. 87%, 75%,
>98:2 *dr*, 81% *ee*

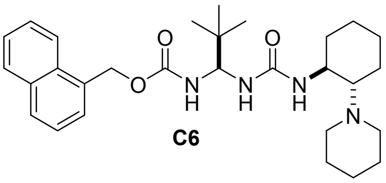

C6
conv. 95%, 70%, >98:2 *dr*, 88% *ee*
conv. 99% ,77%, >98:2 *dr*, 94% *ee*[b]

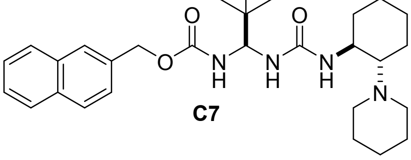

C7
conv. 78%, 58%,
>98:2 *dr*, 78% *ee*

[b] Reaction carried out at 0º C

Other catalysts screened for the reaction of **1** with **4a:**

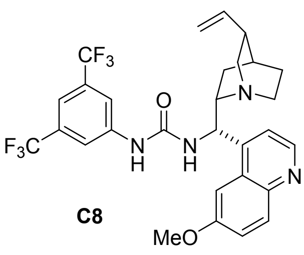

C8

conv. 58%, 43%,
88:12 *dr*, 2% *ee*

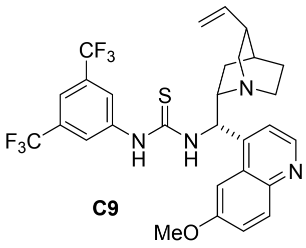

C9

conv. 43%, 30%,
83:17 *dr*, 14% *ee*

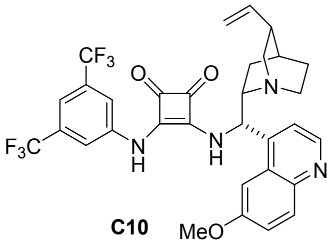

C10

conv. 80%,73%,
70:30 *dr*, 70% *ee*

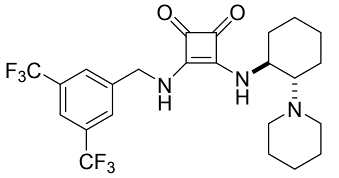

C11

conv. 40%,29%,
90:10 *dr*, 15% *ee*

2. Formation of 8a through cyclization

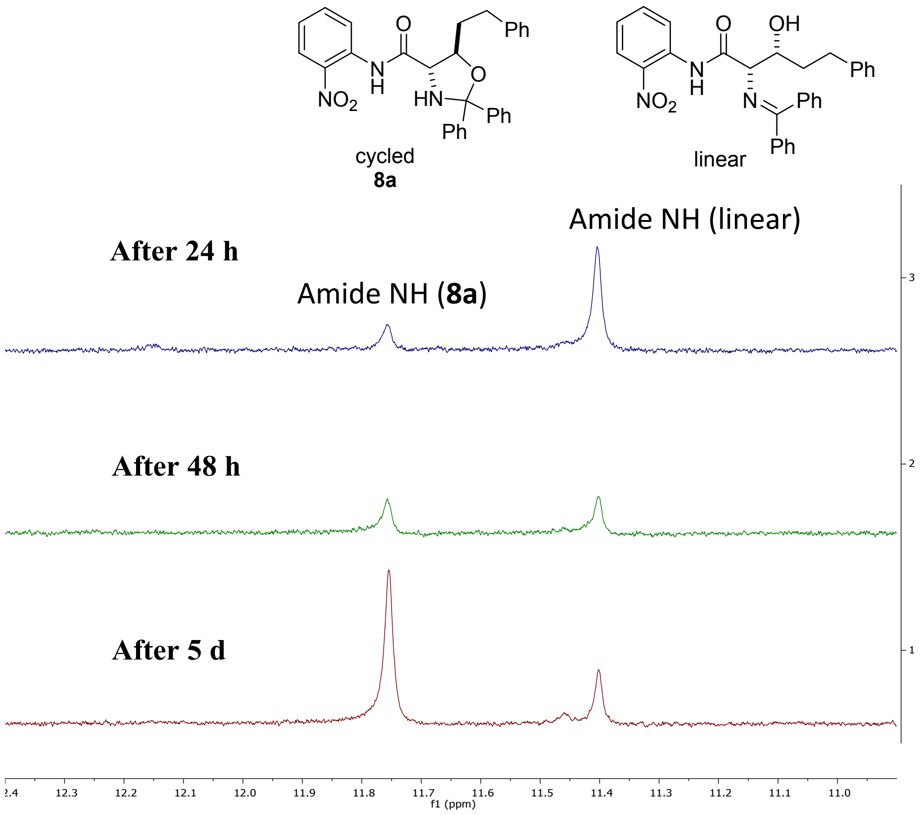

t	Conv. (%)	Linear	Cyclisized (8a)
24 h	>99%	82%	18%
48 h		53%	47%
5 days		30%	70%

Formation of the cycled product with substrates **2**, **3** was not observed under their corresponding standard reaction conditions.

3. Enantioselectivity before and after reductive work-up

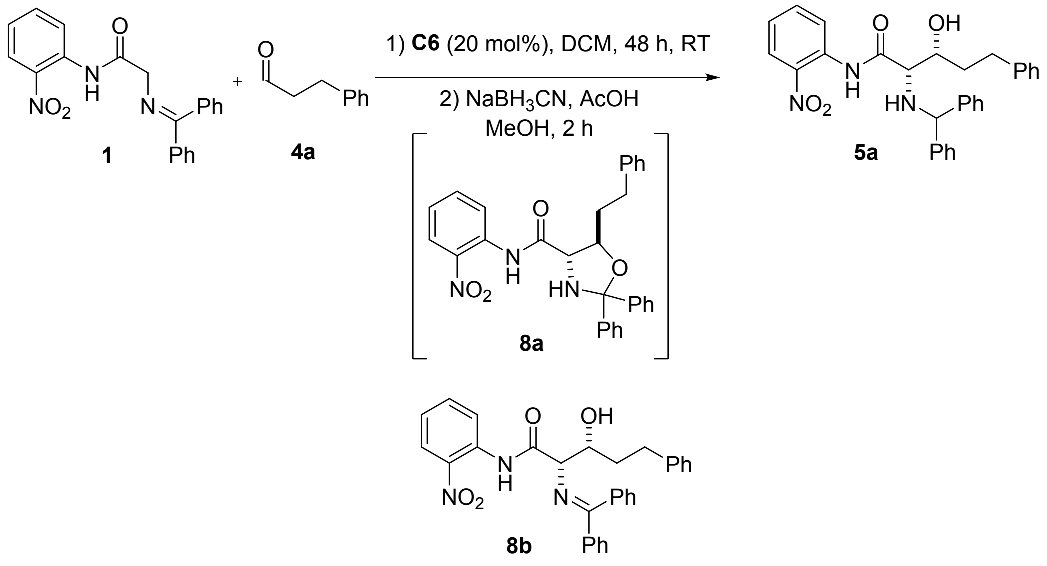

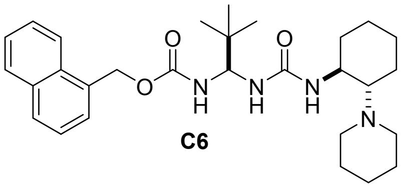

C6

Entry	Tot. Conv. (%)[b]	Yield (%)[c]	8a *dr*[d]	8b *dr*[d]	5a *dr*[d]	8b *ee* (%)[e]	5a *ee* (%)[e]
1	90	70	>98:2	>98:2	-	88	-
2	87	75	>98:2	>98:2	>98:2	-	87

Reactions conducted on a 0.2 mmol scale in 0.4 mL of CH_2Cl_2 (mol ratio *N*-(diarylmethylene)glycine o-nitroanilide / hydrocinnamaldehyde / catalyst 1:3:0.2). [b] Determined by the disappearance of the starting **1**. [c] Isolated yield of **5a** and the corresponding minor isomer. [d] Determined by ^{1}H NMR (300 MHz) analysis on an aliquote (**8a** and **8b**) or the crude product (**5a**), before isolation by column chromatography. [e] Determined by chiral HPLC.

Nitroanilide **1** (0.1 mmol, 1 equiv.) was dissolved in dry dichloromethane (0.5 mL) and hydrocinnamaldehyde (0.6 mmol, 3 equiv.) was added, followed by **C6** (11mg, 0.02 mmol, 20 mol%), and triethylamine (3 μL, 0.02 mmol, 20 mol%). The reaction mixture was stirred at room temperature for 48 h. The solvent was evaporated and the crude was purified by flash column chromatography. When reductive work up was carried out, MeOH (0.4 mL) was added, followed by $NaBH_3CN$ (32 mg, 0.5 mmol, 2.5 equiv.) and AcOH (24 μL, 0.4 mmol, 2 equiv.) and the mixture was stirred for 2 h (the reduction of the imine can be followed by ^{1}H NMR). The solvents were evaporated under reduced pressure, the residue redissolved in dichloromethane and washed with a saturated $NaHCO_3$ solution (1 x 4 mL). The organic phase was dried over $MgSO_4$ and evaporated *in vacuo*. The crude was purified by flash column chromatography on silica gel.

3. X-Ray analysis

View of the molecular structures of **1**, **3** and **10** with 50% probability displacement ellipsoids.

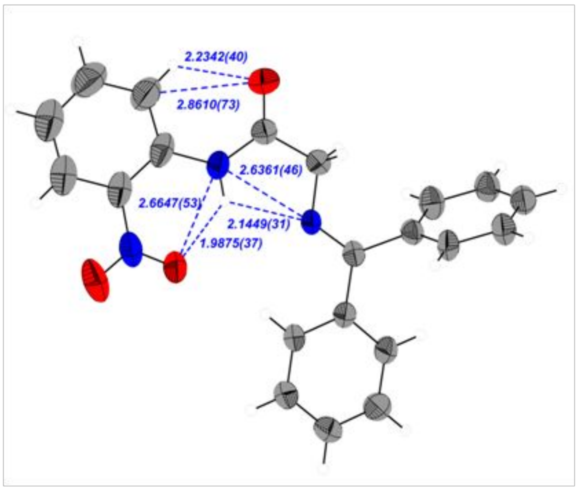

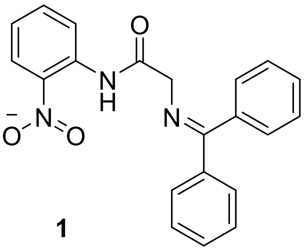

1

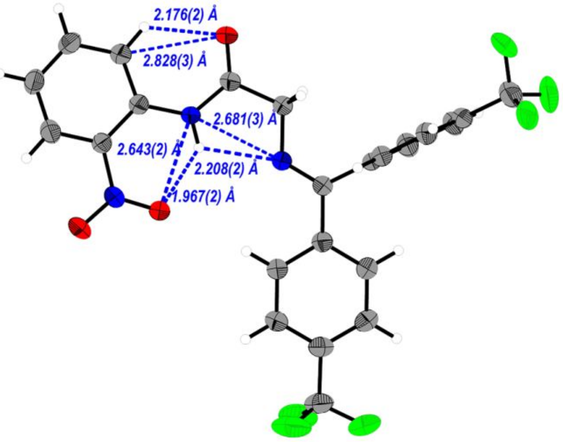

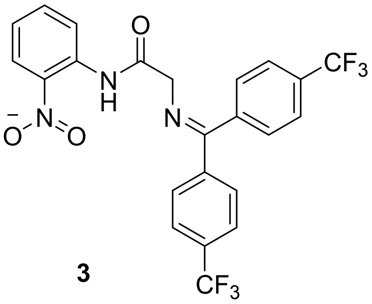

3

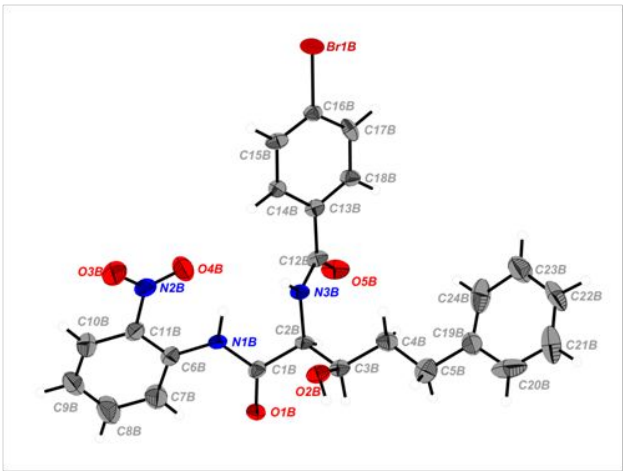

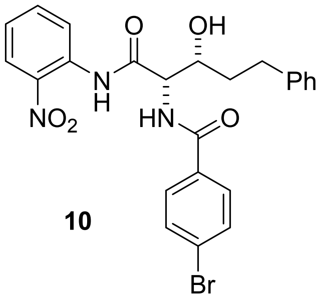

10

4. Computational analysis

4.1 Computational methods

All the computational studies were carried out by means of Gaussian 16 suite of programs. The calculations were performed within the Density Functional Theory[1] (DFT) framework using the B3LYP[2] functional in combination with 6-31G(d) or 6-311+G(d,p) basis sets. Dispersion corrections are included by means of Grimme's D3 model.[3] Solvent effects were estimated using the polarizable continuum model[4] (PCM) within the self-consistent reaction field (SCRF) approach. All SCRF-PCM calculations were performed using CH_2Cl_2 (ε =8.93) as model solvent. Thermal corrections were computed at the same level of theory as the optimization and were not scaled. All stationary points were characterized by harmonic analysis. All enolates have positive definite Hessian matrices. Relative (Gibbs) energies were calculated at room temperature.

4.2 Complete reference 33b

Gaussian 16, Revision B.01, Frisch, M. J.; Trucks, G. W.; Schlegel, H. B.; Scuseria, G. E.; Robb, M. A.; Cheeseman, J. R.; Scalmani, G.; Barone, V.; Petersson, G. A.; Nakatsuji, H.; Li, X.; Caricato, M.; Marenich, A. V.; Bloino, J.; Janesko, B. G.; Gomperts, R.; Mennucci, B.; Hratchian, H. P.; Ortiz, J. V.; Izmaylov, A. F.; Sonnenberg, J. L.; Williams-Young, D.; Ding, F.; Lipparini, F.; Egidi, F.; Goings, J.; Peng, B.; Petrone, A.; Henderson, T.; Ranasinghe, D.; Zakrzewski, V. G.; Gao, J.; Rega, N.; Zheng, G.; Liang, W.; Hada, M.; Ehara, M.; Toyota, K.; Fukuda, R.; Hasegawa, J.; Ishida, M.; Nakajima, T.; Honda, Y.; Kitao, O.; Nakai, H.; Vreven, T.; Throssell, K.; Montgomery, J. A., Jr.; Peralta, J. E.; Ogliaro, F.; Bearpark, M. J.; Heyd, J. J.; Brothers, E. N.; Kudin, K. N.; Staroverov, V. N.; Keith, T. A.; Kobayashi, R.; Normand, J.; Raghavachari, K.; Rendell, A. P.; Burant, J. C.; Iyengar, S. S.; Tomasi, J.; Cossi, M.; Millam, J. M.; Klene, M.; Adamo, C.; Cammi, R.; Ochterski, J. W.; Martin, R. L.; Morokuma, K.; Farkas, O.; Foresman, J. B.; Fox, D. J. Gaussian, Inc., Wallingford CT, **2016**.

4.3. Energies, thermal corrections and cartesian coordinates of all computed stationary points

Table S1. Total electronic energies[a] (E, in a.u.), zero point correction of the energy[b] (ZPCE), thermal corrections to Gibbs free energies[b] (TCGFE, in a.u.), and number of imaginary frequencies (NIMAG) of all stationary points discussed in the main text and in the Supporting Information.

Structure	E	ZPCE	TCGFE	NIMAG(ν)
***E*-Ar**	-1874.521539	0.338468	0.272182	0
***Z*-Ar**	-1874.514876	0.338095	0.270980	0

[a]Computed at B3LYP-D3(PCM)/6-311+G(d,p)//B3LYP-D3(PCM)/6-31G(d) level. [b]Computed at 298.15 K at B3LYP-D3(PCM)/6-31G(d) level.

Cartesian coordinates (optimized at the B3LYP-D3(PCM)/6-31G(d) level) of all the stationary points collected in the main text and in the computational part of the SI.

***E*-Ar**

Center Number	Atomic Number	Atomic Type	Coordinates (Angstroms) X	Y	Z
1	6	0	5.021082	-1.336665	-0.069825
2	6	0	4.000474	-2.344394	0.010015
3	6	0	4.460605	-3.686117	0.140180
4	6	0	5.805835	-3.991996	0.186336
5	6	0	6.790790	-2.988826	0.108067
6	6	0	6.389141	-1.676887	-0.017378
7	1	0	3.707973	-4.457857	0.201583
8	1	0	6.102500	-5.033081	0.284329
9	1	0	7.846134	-3.238925	0.141659
10	1	0	7.111965	-0.874508	-0.084365
11	7	0	4.727944	0.064979	-0.213544
12	8	0	5.658877	0.885054	-0.176427
13	8	0	3.545699	0.435495	-0.381721
14	7	0	2.668577	-2.044444	-0.021241
15	6	0	1.541843	-2.899152	0.096899
16	8	0	1.639391	-4.137638	0.195913
17	1	0	2.425404	-1.061772	-0.126642
18	6	0	0.289999	-2.198712	0.084490
19	1	0	-0.593440	-2.835450	0.154065
20	7	0	0.251546	-0.869466	0.027279
21	6	0	-0.821759	-0.091165	0.026417
22	6	0	-0.603608	1.357559	0.046959
23	6	0	-1.629544	2.282608	-0.265715
24	6	0	0.670307	1.896003	0.360487
25	6	0	-1.400568	3.653497	-0.262319
26	1	0	-2.617732	1.920060	-0.528507
27	6	0	0.901514	3.262435	0.354607
28	1	0	1.476230	1.217226	0.608635
29	6	0	-0.134088	4.158671	0.050710
30	1	0	-2.208620	4.336468	-0.506851
31	1	0	1.888122	3.642808	0.602965
32	6	0	0.132581	5.625301	-0.007337
33	9	0	-0.977281	6.365166	0.246266
34	9	0	1.082077	6.013777	0.882054
35	9	0	0.580270	6.029915	-1.230351
36	6	0	-2.209900	-0.629854	0.016564
37	6	0	-3.157892	-0.222131	0.975318
38	6	0	-2.614533	-1.579765	-0.941107
39	6	0	-4.451608	-0.734176	0.978481
40	1	0	-2.868009	0.505582	1.727521
41	6	0	-3.905583	-2.098673	-0.944492

42	1	0	-1.904916	-1.907651	-1.694345
43	6	0	-4.829047	-1.678139	0.018067
44	1	0	-5.164196	-0.414229	1.732212
45	1	0	-4.198538	-2.828302	-1.692859
46	6	0	-6.237784	-2.185447	-0.021904
47	9	0	-6.317342	-3.441972	-0.522801
48	9	0	-7.038294	-1.412070	-0.804151
49	9	0	-6.811722	-2.205372	1.205636

Z-**Ar**

Center Number	Atomic Number	Atomic Type	Coordinates (Angstroms) X	Y	Z
1	6	0	5.865089	-1.230554	0.065534
2	6	0	4.620352	-1.947196	0.012787
3	6	0	4.714788	-3.367262	-0.018353
4	6	0	5.935634	-4.010880	0.002461
5	6	0	7.145784	-3.291856	0.055699
6	6	0	7.098605	-1.915369	0.086432
7	1	0	3.787880	-3.919628	-0.059232
8	1	0	5.954687	-5.097289	-0.022633
9	1	0	8.100081	-3.808013	0.072145
10	1	0	8.004579	-1.325285	0.126893
11	7	0	5.940678	0.205868	0.098359
12	8	0	7.053354	0.752749	0.140974
13	8	0	4.889302	0.888089	0.082930
14	7	0	3.416152	-1.302345	-0.005281
15	6	0	2.090118	-1.829953	-0.065575
16	8	0	1.876008	-3.052622	-0.117107
17	1	0	3.499693	-0.291384	0.024351
18	6	0	1.114961	-0.776374	-0.062291
19	1	0	1.468886	0.257630	-0.006715
20	7	0	-0.178288	-1.087790	-0.098273
21	6	0	-1.200062	-0.247338	-0.077924
22	6	0	-2.544420	-0.828501	-0.060998
23	6	0	-3.713648	-0.052628	-0.251353
24	6	0	-2.723733	-2.224347	0.120716
25	6	0	-4.978870	-0.628313	-0.242804
26	1	0	-3.628185	1.015184	-0.422454
27	6	0	-3.984095	-2.798890	0.124838
28	1	0	-1.840649	-2.839405	0.253793
29	6	0	-5.128654	-2.006172	-0.054665
30	1	0	-5.855780	-0.007010	-0.398429
31	1	0	-4.089823	-3.870794	0.265378
32	6	0	-6.486922	-2.617225	0.024653
33	9	0	-7.410958	-1.923785	-0.688041
34	9	0	-6.506556	-3.895172	-0.433805
35	9	0	-6.964511	-2.673092	1.301480
36	6	0	-1.031355	1.234770	-0.055562
37	6	0	-0.272799	1.890627	-1.043077
38	6	0	-1.594230	2.019263	0.968934
39	6	0	-0.083857	3.269348	-1.012724
40	1	0	0.170504	1.306986	-1.844166
41	6	0	-1.413094	3.398664	1.006281
42	1	0	-2.177876	1.533709	1.745622
43	6	0	-0.653268	4.027583	0.014903
44	1	0	0.504193	3.757414	-1.783430
45	1	0	-1.846899	3.985825	1.809637
46	6	0	-0.506739	5.518719	0.019085
47	9	0	-0.514234	6.030841	1.273839
48	9	0	-1.519699	6.133454	-0.648937
49	9	0	0.641747	5.924367	-0.573508

5. NMR spectra and HPLC chromatograms

^{1}HNMR (300 MHz)

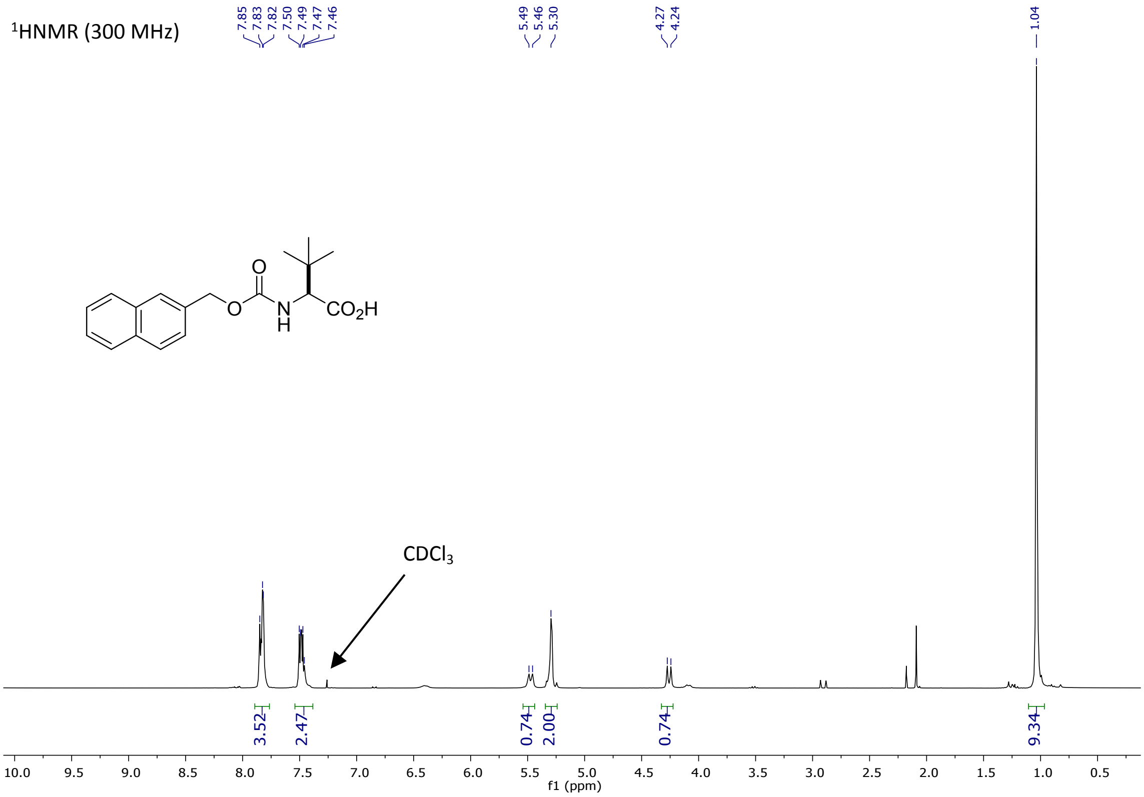

^{1}HNMR (300 MHz)

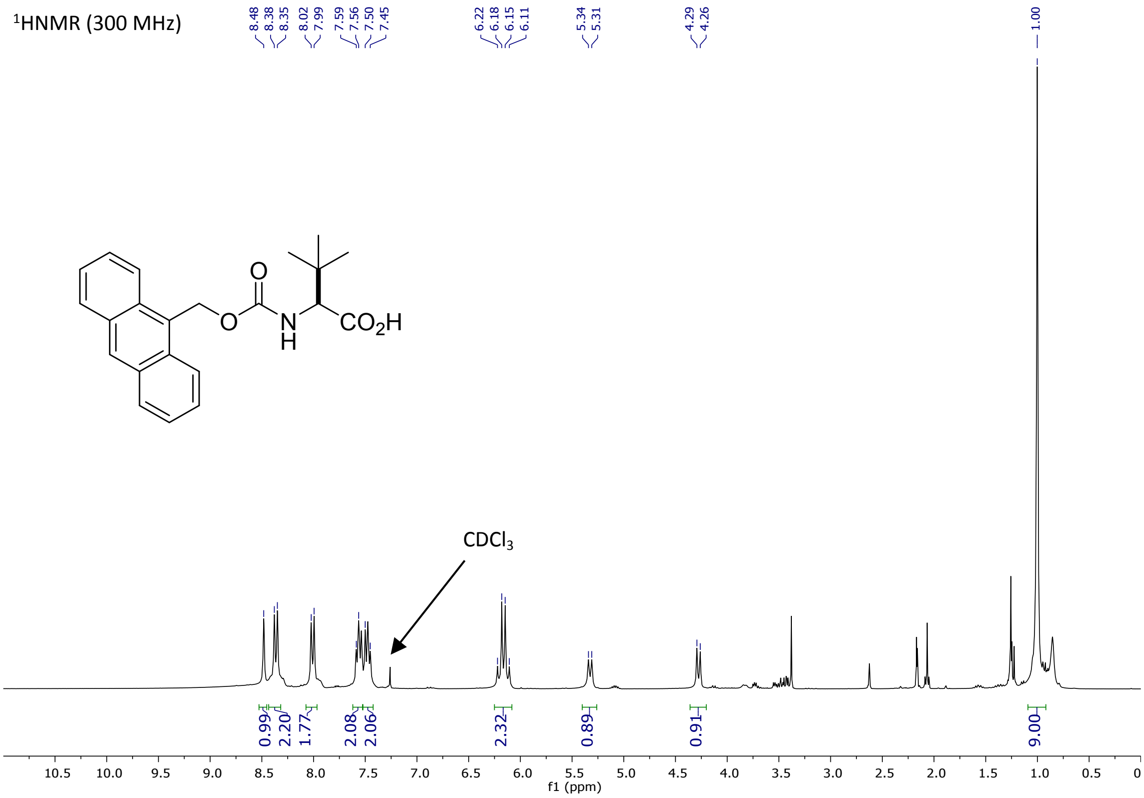

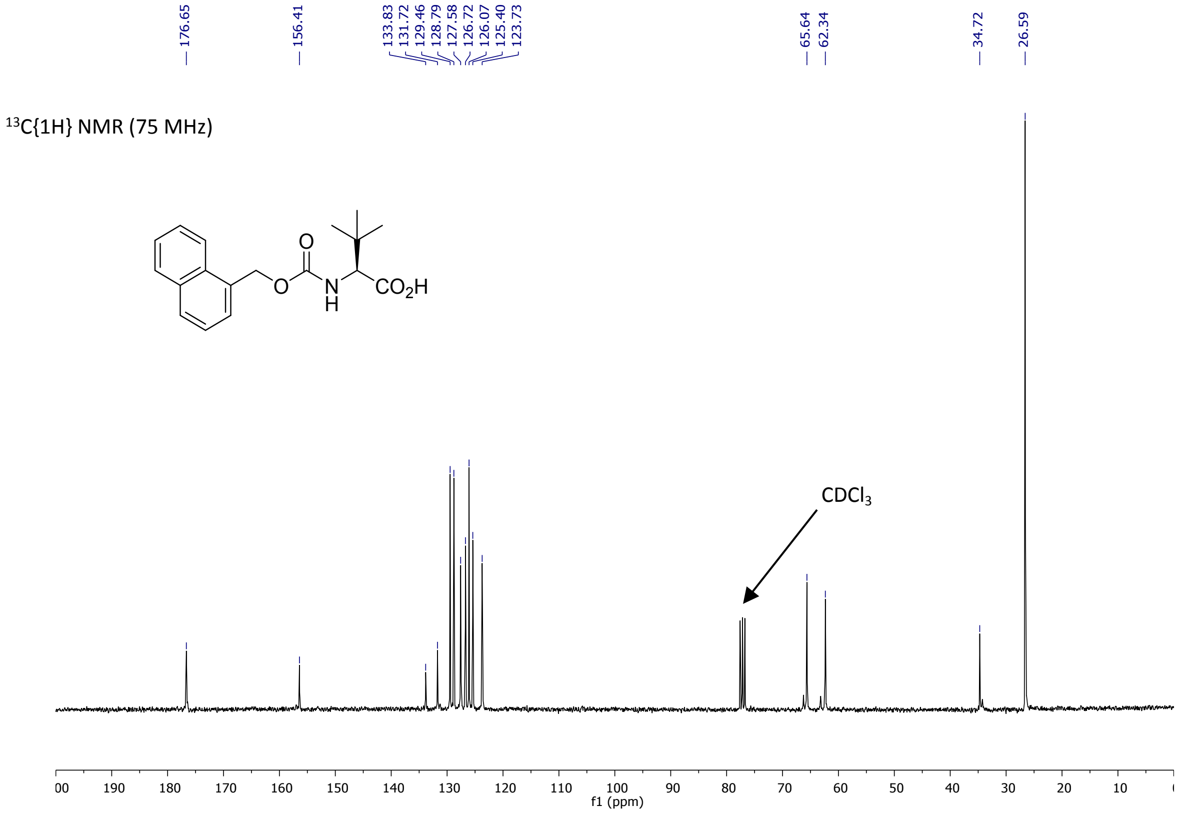

176.65
156.41
133.83
131.72
129.46
128.79
127.58
126.72
126.07
125.40
123.73
65.64
62.34
34.72
26.59
$^{13}C\{1H\}$ NMR (75 MHz)
O
O
N
H
CO_2H
$CDCl_3$
f1 (ppm)

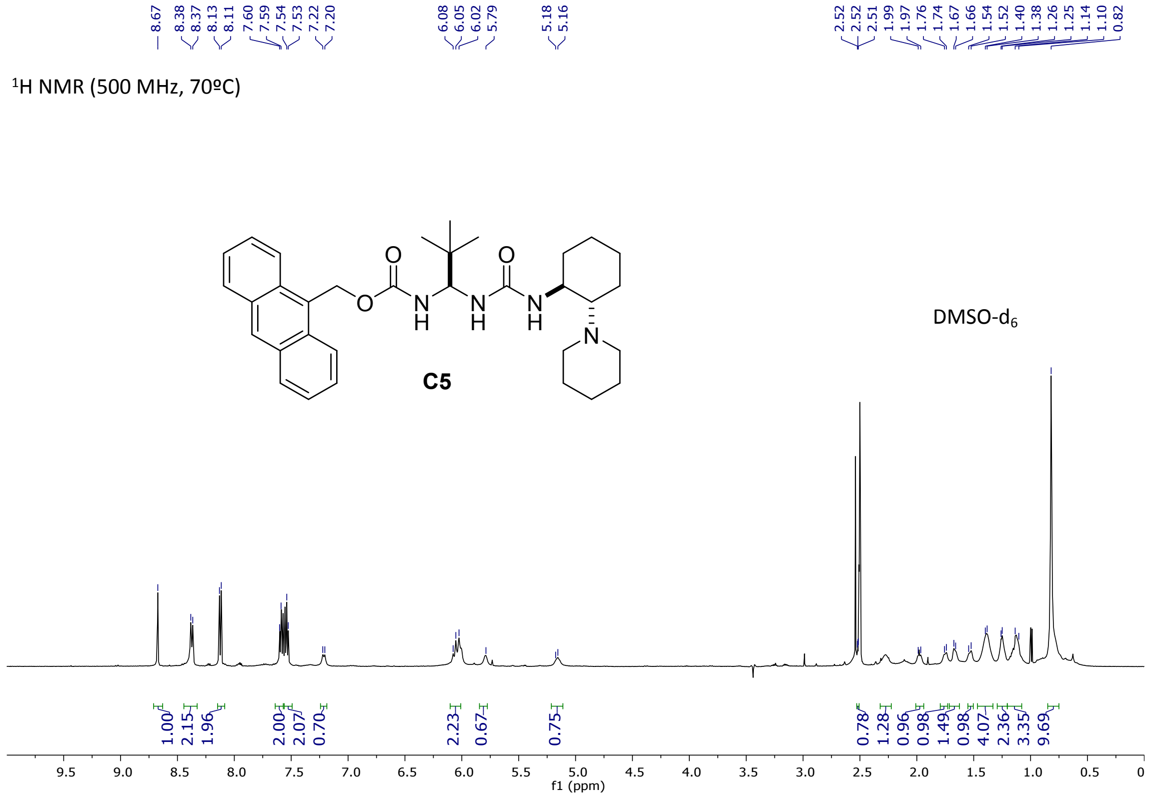
8.67 8.38 8.37 8.13 8.11 7.60 7.59 7.54 7.53 7.22 7.20 6.08 6.05 6.02 5.79 5.18 5.16 2.52 2.52 2.51 1.99 1.97 1.76 1.74 1.67 1.66 1.54 1.52 1.40 1.38 1.26 1.25 1.14 1.10 0.82
^{1}H NMR (500 MHz, 70ºC)
C5
DMSO-d$_6$
1.00 2.15 1.96 2.00 2.07 0.70 2.23 0.67 0.75 0.78 1.28 0.96 0.98 1.49 0.98 4.07 2.36 3.35 9.69
f1 (ppm)

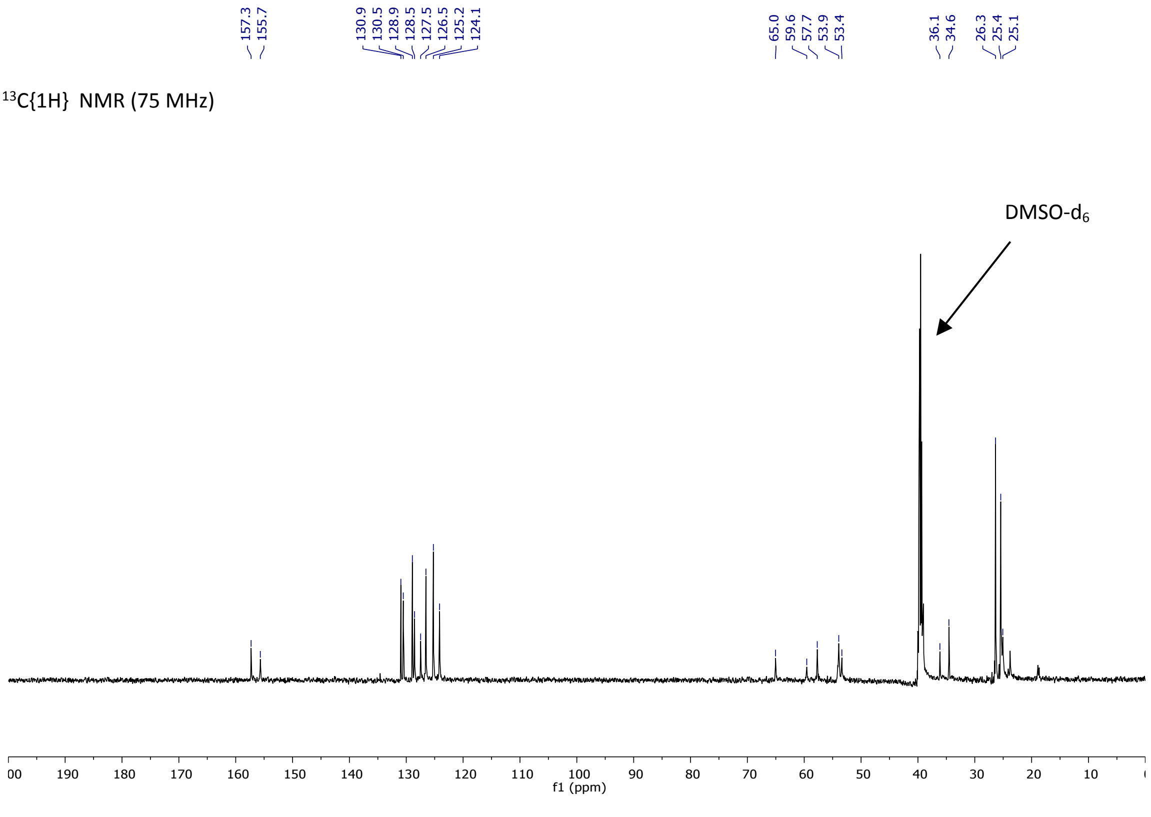
157.3 155.7 130.9 130.5 128.9 128.5 127.5 126.5 125.2 124.1 65.0 59.6 57.7 53.9 53.4 36.1 34.6 26.3 25.4 25.1
$^{13}C\{1H\}$ NMR (75 MHz)
DMSO-d$_6$
f1 (ppm)

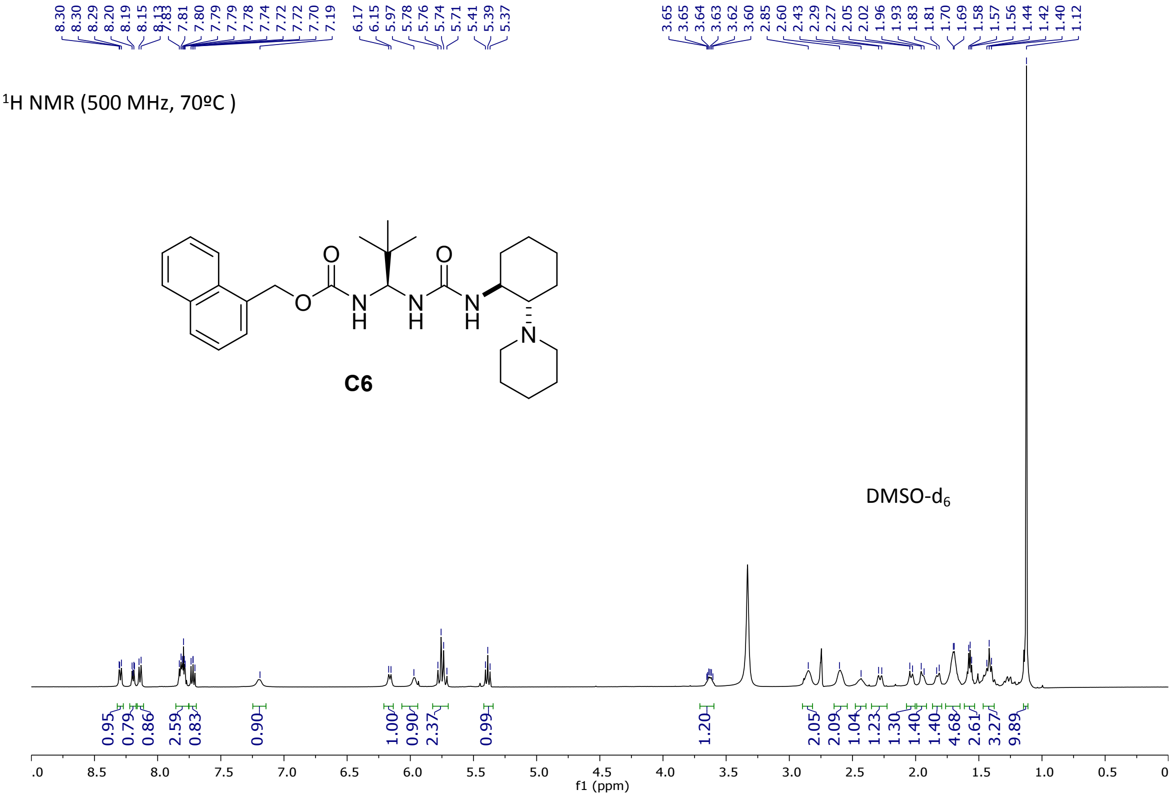
8.30 8.30 8.29 8.20 8.19 8.15 8.13 7.83 7.81 7.80 7.79 7.79 7.78 7.74 7.72 7.72 7.70 7.19 6.17 6.15 5.97 5.78 5.76 5.74 5.71 5.41 5.39 5.37
3.65 3.65 3.64 3.63 3.62 3.60 2.85 2.60 2.43 2.29 2.27 2.05 2.02 1.96 1.93 1.83 1.81 1.70 1.69 1.58 1.57 1.56 1.44 1.42 1.40 1.12
^{1}H NMR (500 MHz, 70ºC)
DMSO-d$_6$
0.95 0.79 0.86 2.59 0.83 0.90 1.00 0.90 2.37 0.99 1.20 2.05 2.09 1.04 1.23 1.30 1.40 1.40 4.68 2.61 3.27 9.89
f1 (ppm)

C6

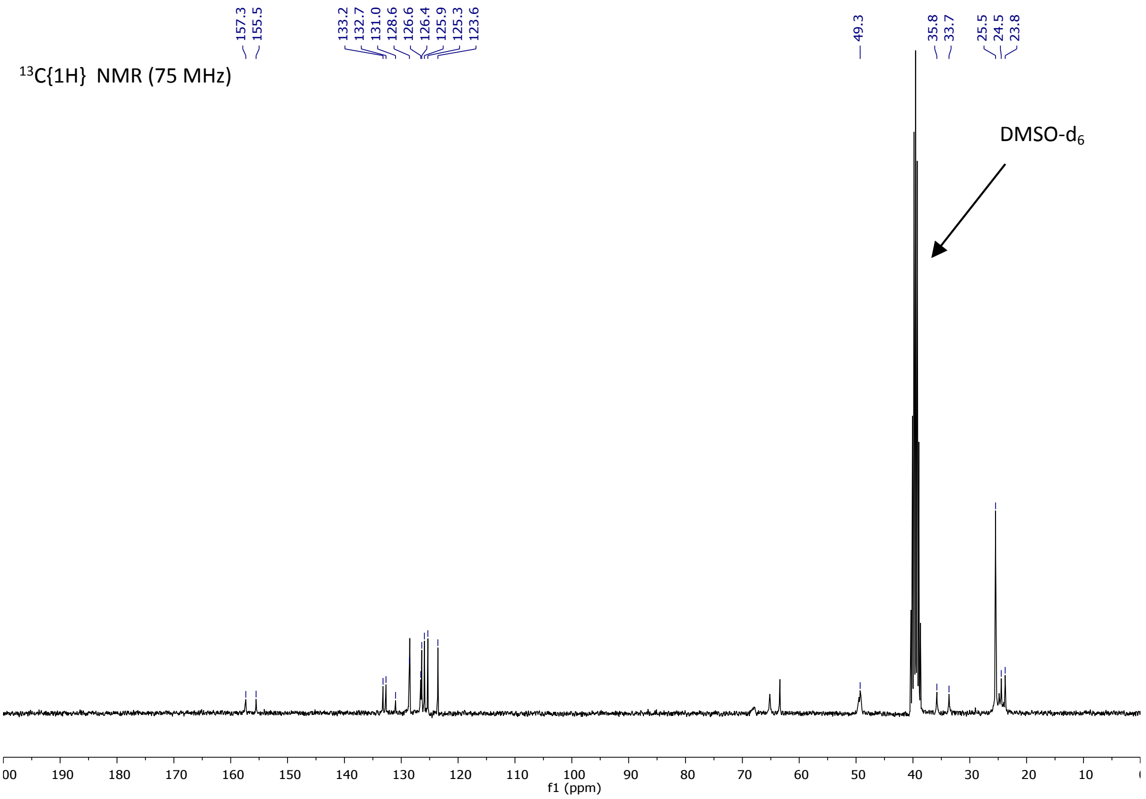
157.3 155.5 133.2 132.7 131.0 128.6 126.6 126.4 125.9 125.3 123.6 49.3 35.8 33.7 25.5 24.5 23.8
^{13}C{1H} NMR (75 MHz)
DMSO-d$_6$
f1 (ppm)

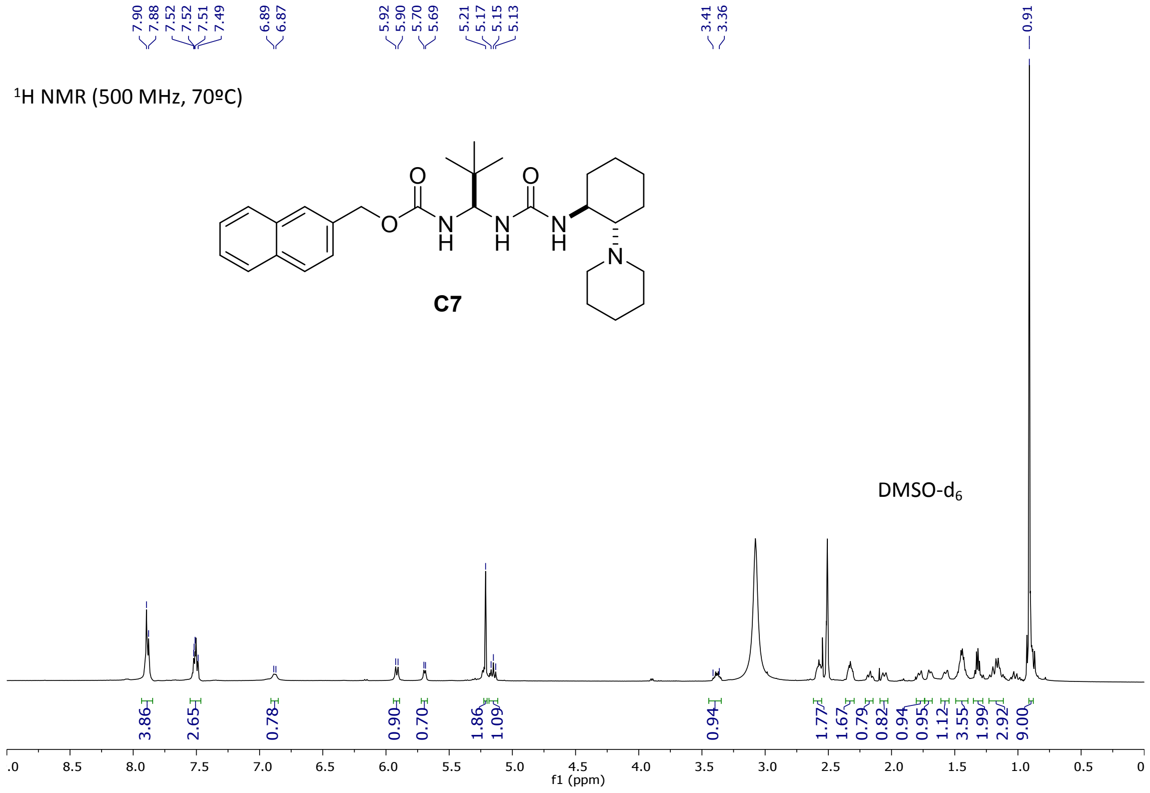
1H NMR (500 MHz, 70ºC)
C7
DMSO-d6
f1 (ppm)

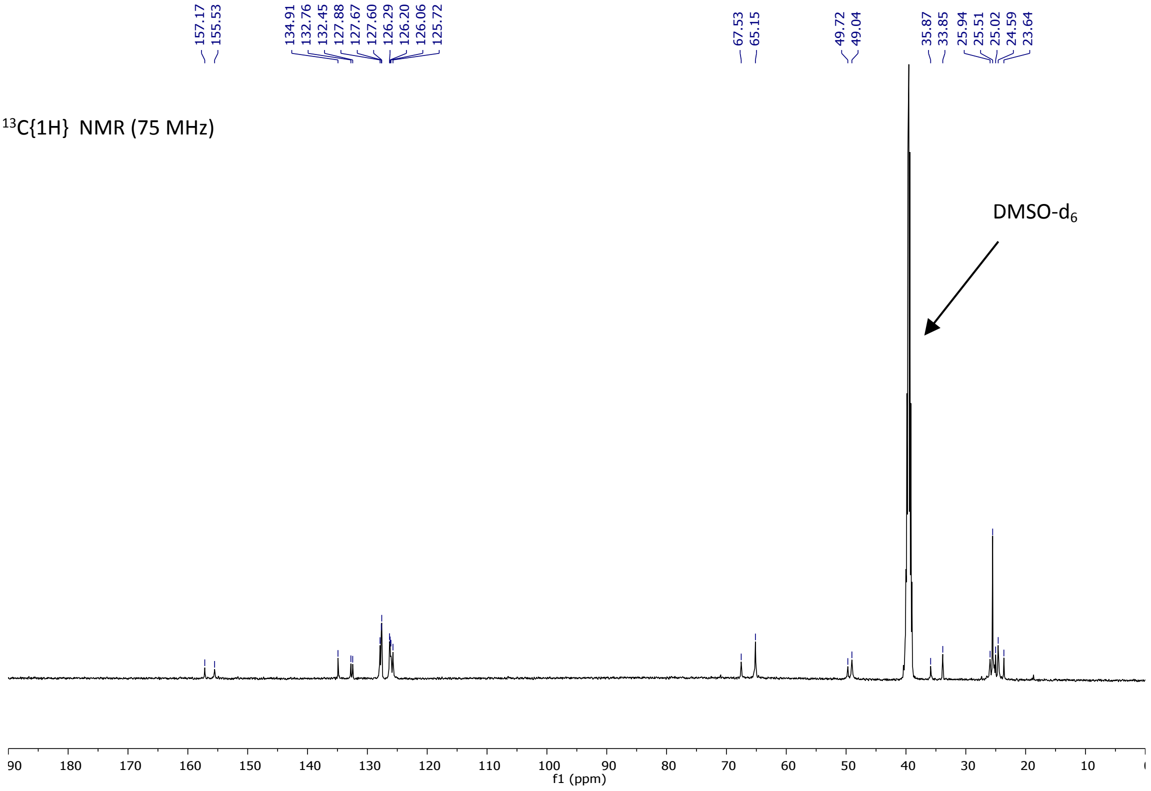
13C{1H} NMR (75 MHz)
DMSO-d6
f1 (ppm)

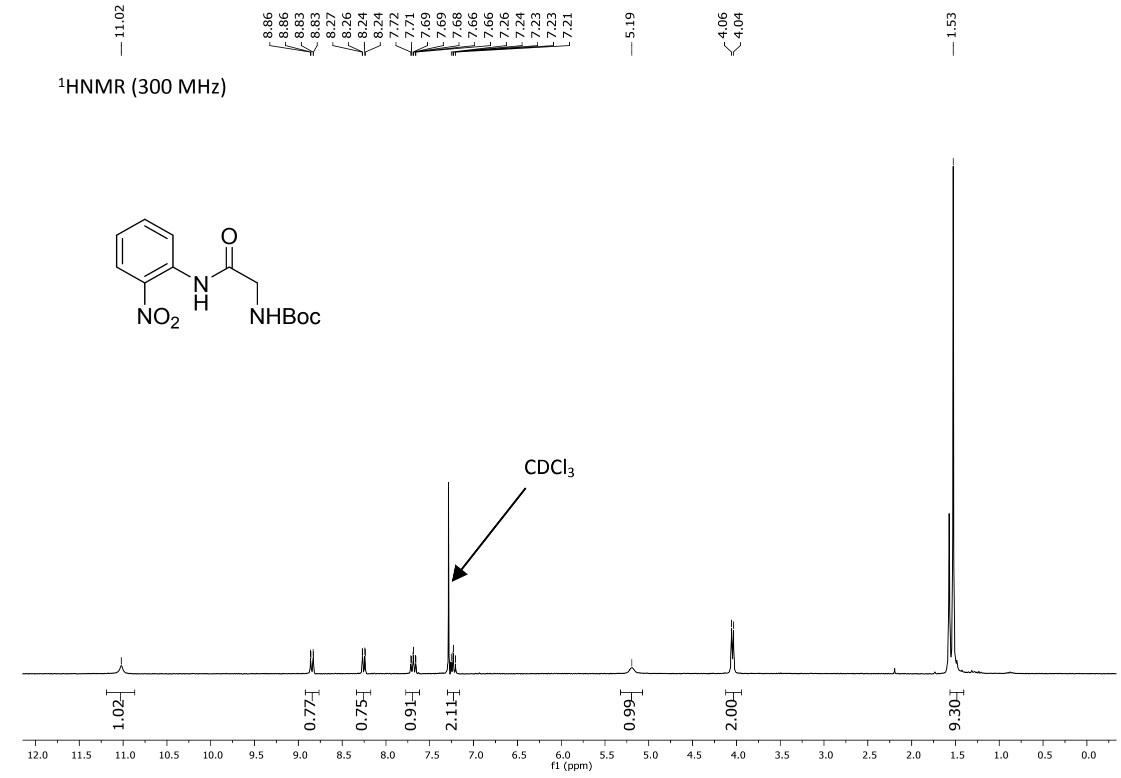
11.02
8.86
8.86
8.83
8.83
8.27
8.26
8.24
8.24
7.72
7.71
7.69
7.69
7.68
7.66
7.66
7.26
7.24
7.23
7.21
5.19
4.06
4.04
1.53
1HNMR (300 MHz)
O
N
H
NO2
NHBoc
CDCl3
1.02
0.77
0.75
0.91
2.11
0.99
2.00
9.30
f1 (ppm)

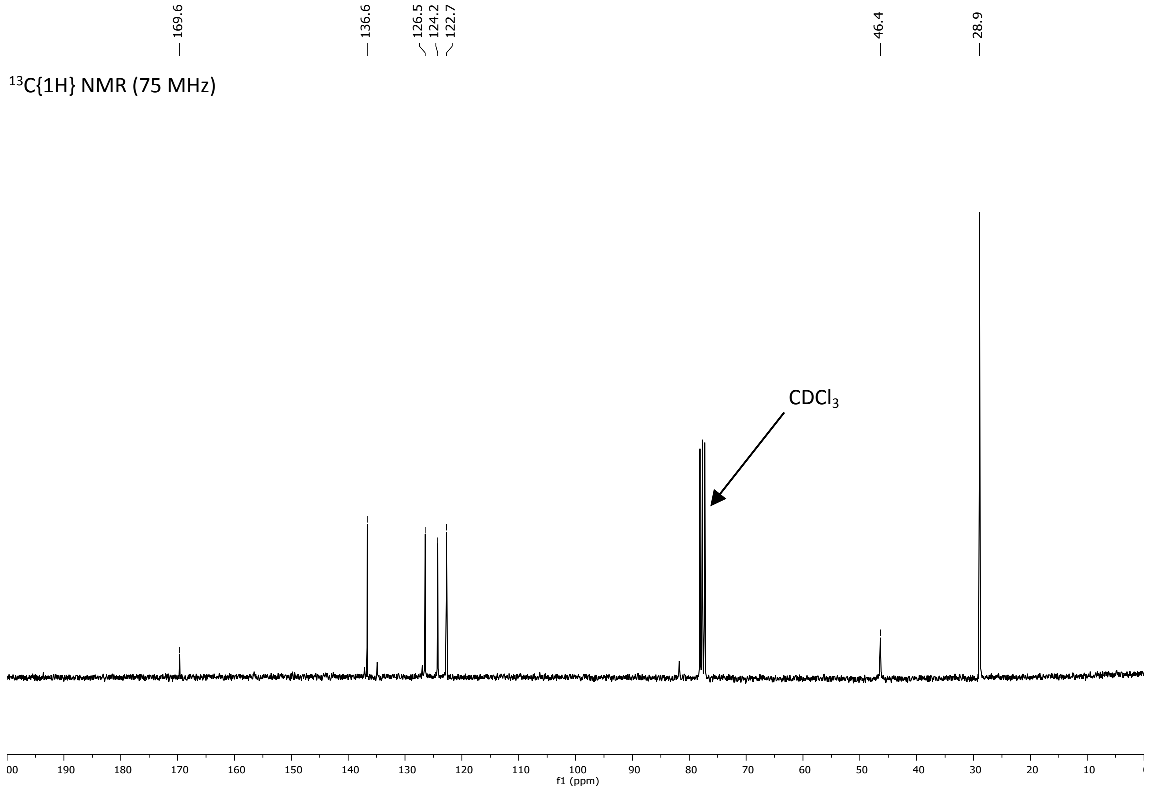
169.6
136.6
126.5
124.2
122.7
46.4
28.9
13C{1H} NMR (75 MHz)
CDCl3
f1 (ppm)

^{1}HNMR (300 MHz)

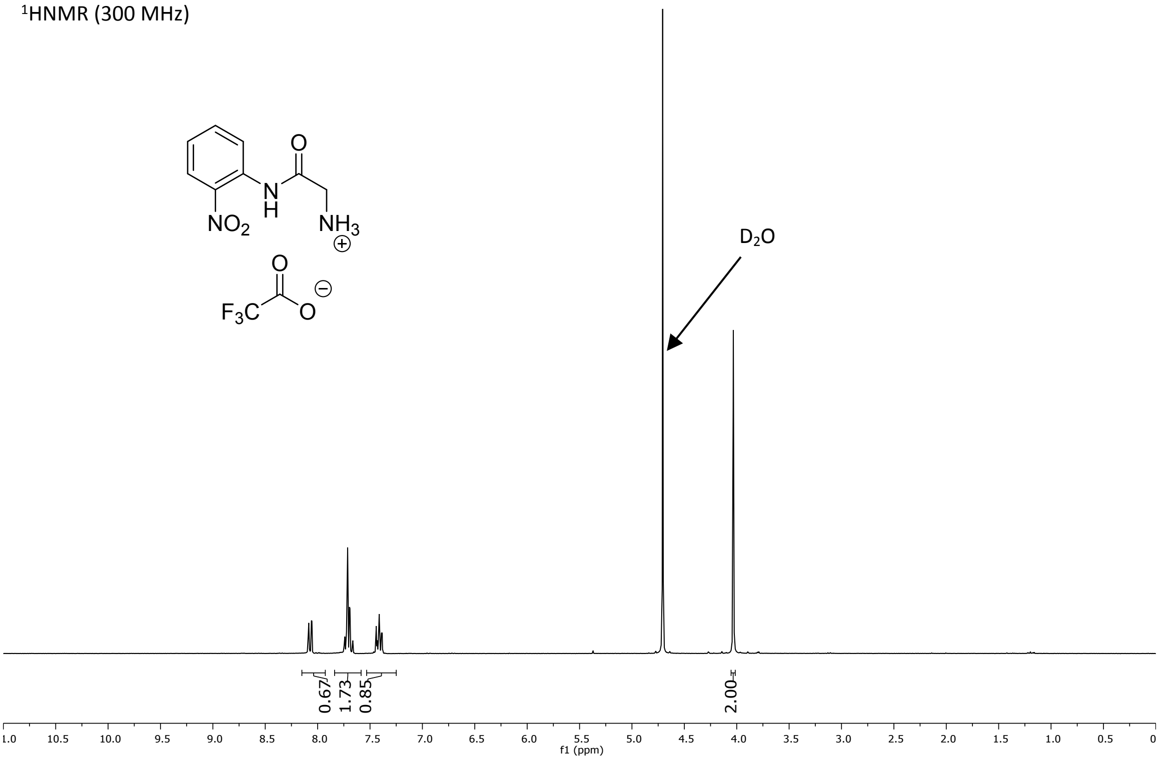

^{13}C{1H} -NMR (75 MHz)

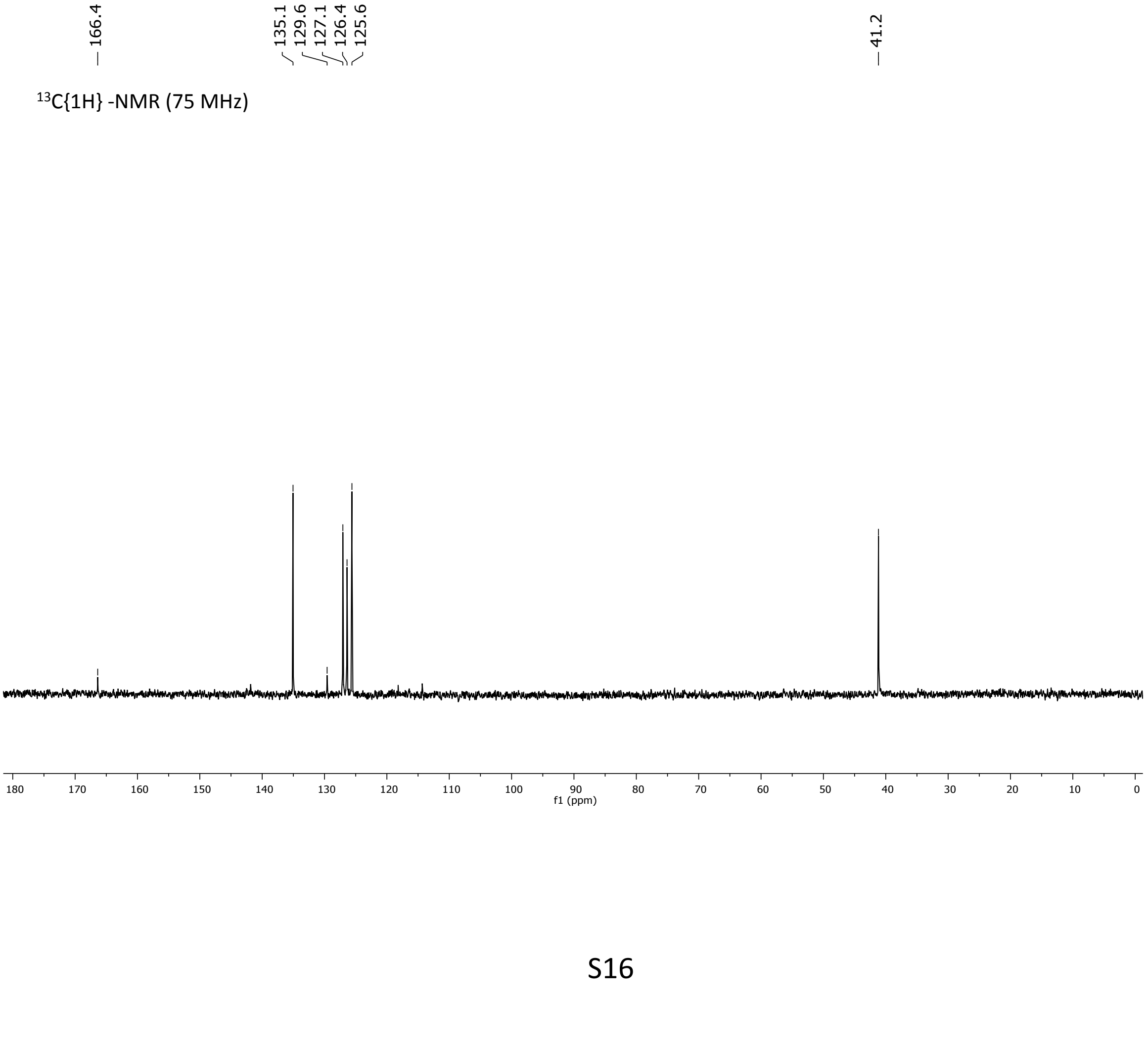

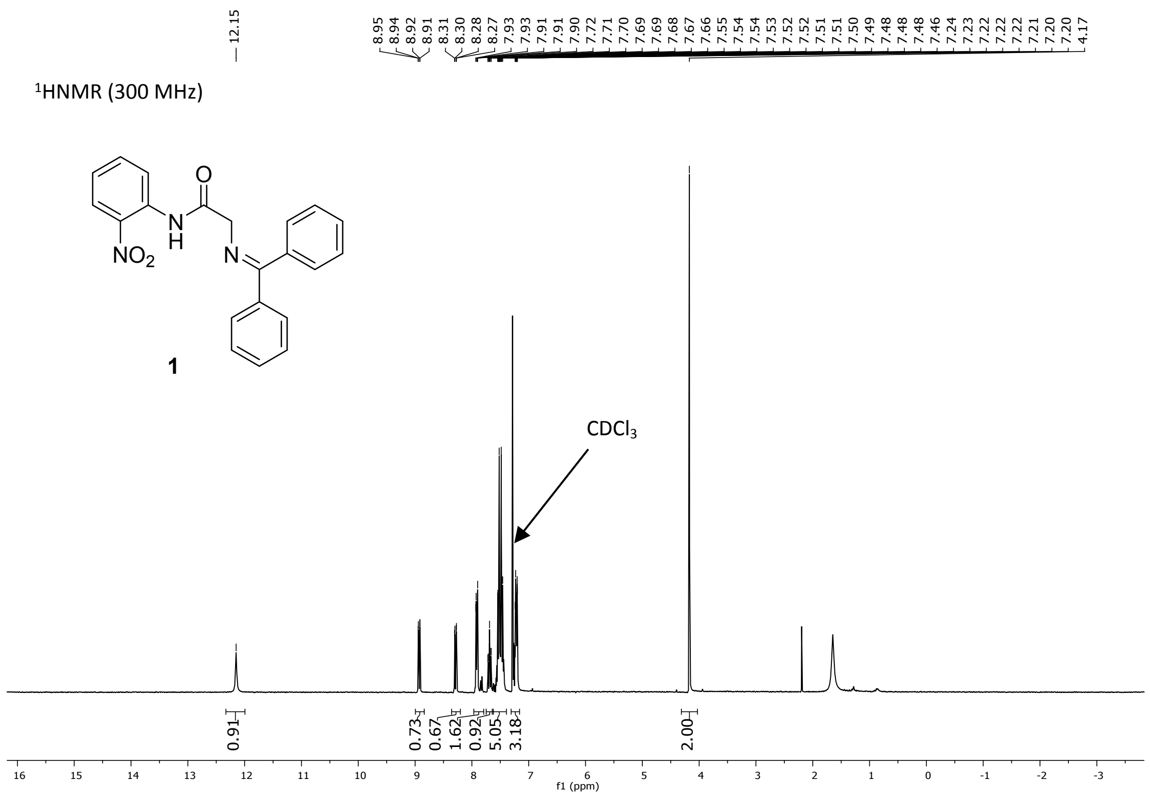
12.15
8.95 8.94 8.92 8.91 8.31 8.30 8.28 8.27 7.93 7.93 7.91 7.91 7.90 7.72 7.71 7.70 7.69 7.69 7.68 7.67 7.66 7.55 7.54 7.54 7.53 7.52 7.52 7.51 7.51 7.50 7.49 7.48 7.48 7.48 7.46 7.24 7.23 7.22 7.22 7.22 7.21 7.20 7.20 4.17
1HNMR (300 MHz)
NO2
H
N
O
N
1
CDCl3
0.91
0.73
0.67
1.62
0.92
5.05
3.18
2.00
f1 (ppm)

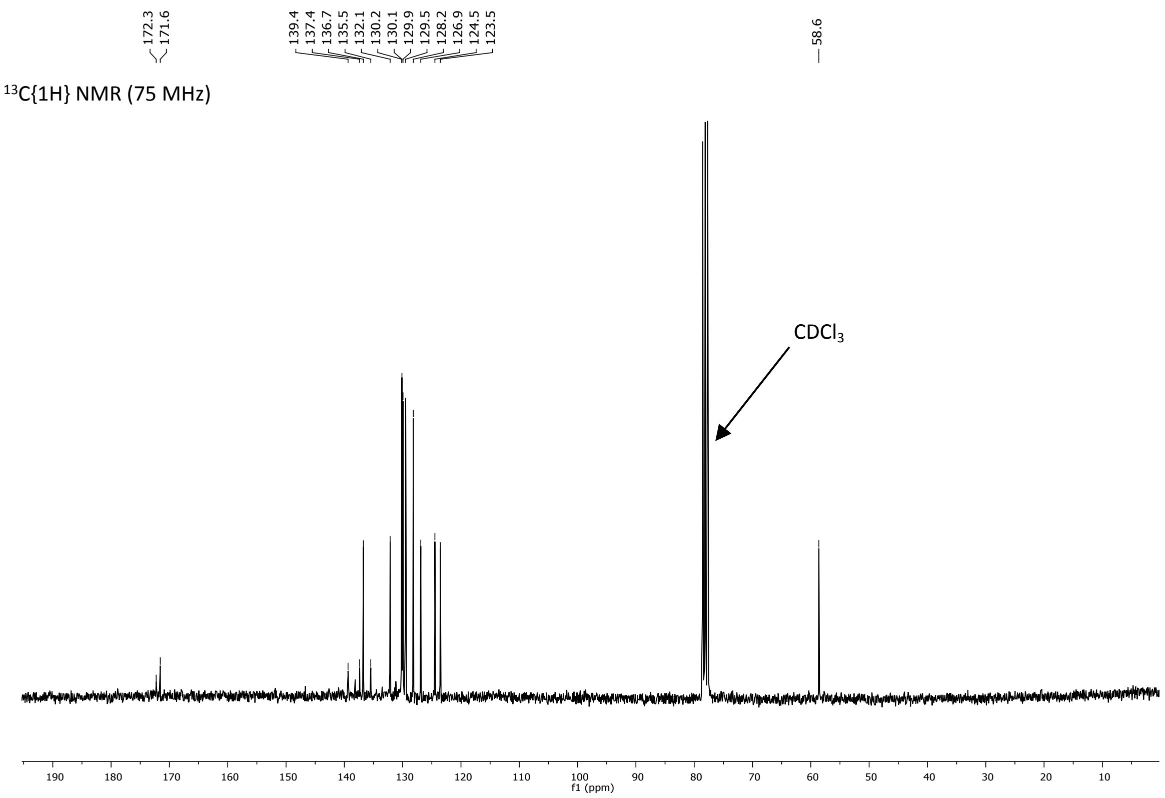
172.3
171.6
139.4 137.4 136.7 135.5 132.1 130.2 130.1 129.9 129.5 128.2 126.9 124.5 123.5
58.6
13C{1H} NMR (75 MHz)
CDCl3
f1 (ppm)

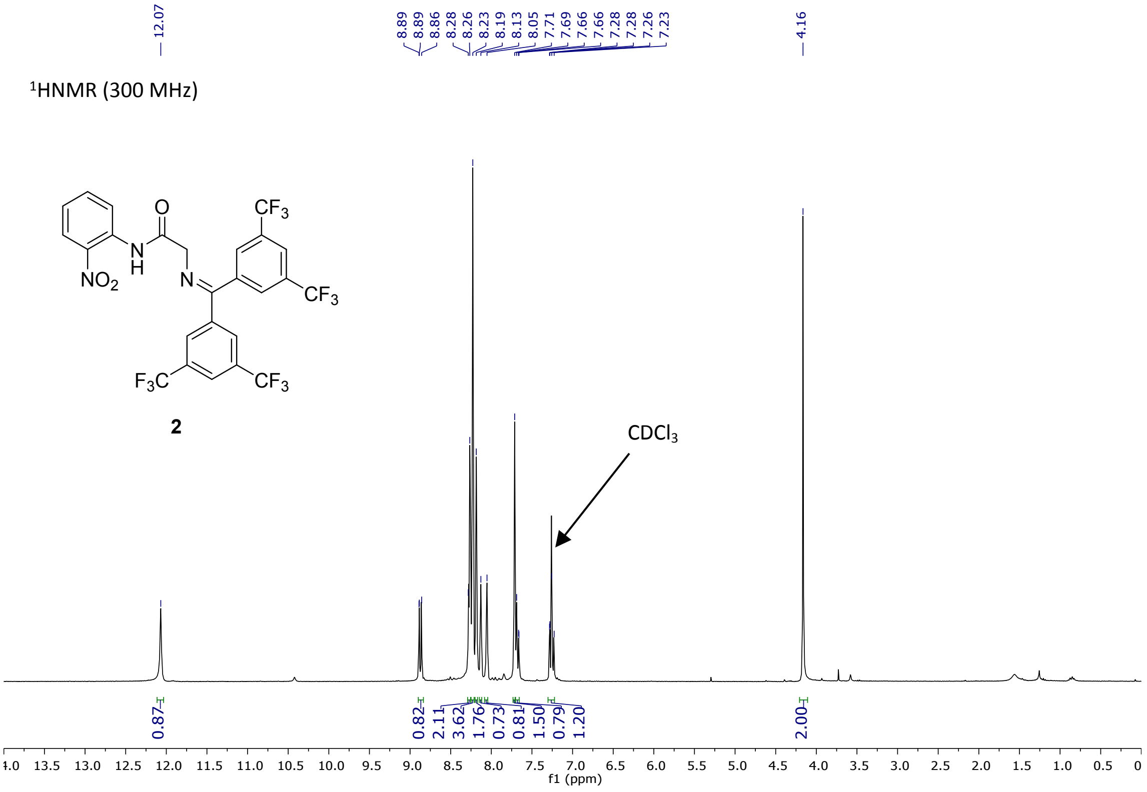

¹HNMR (300 MHz)
2
CDCl3
f1 (ppm)

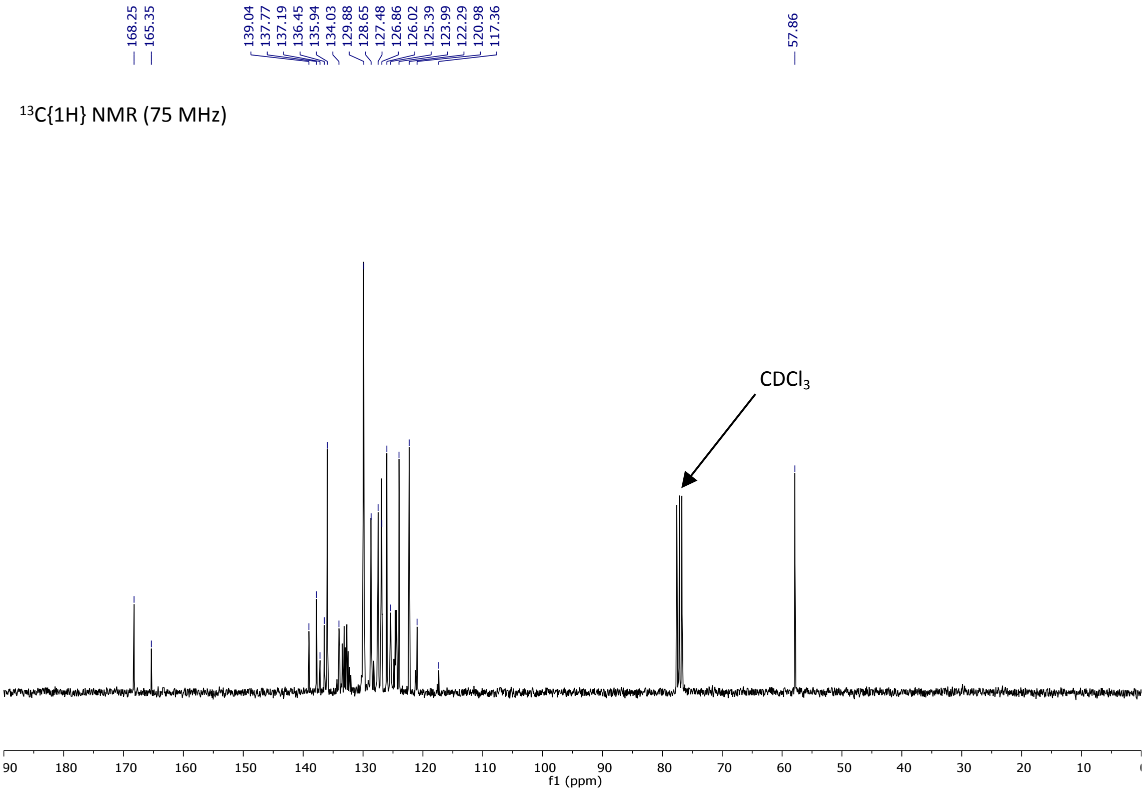

¹³C{1H} NMR (75 MHz)
CDCl3
f1 (ppm)

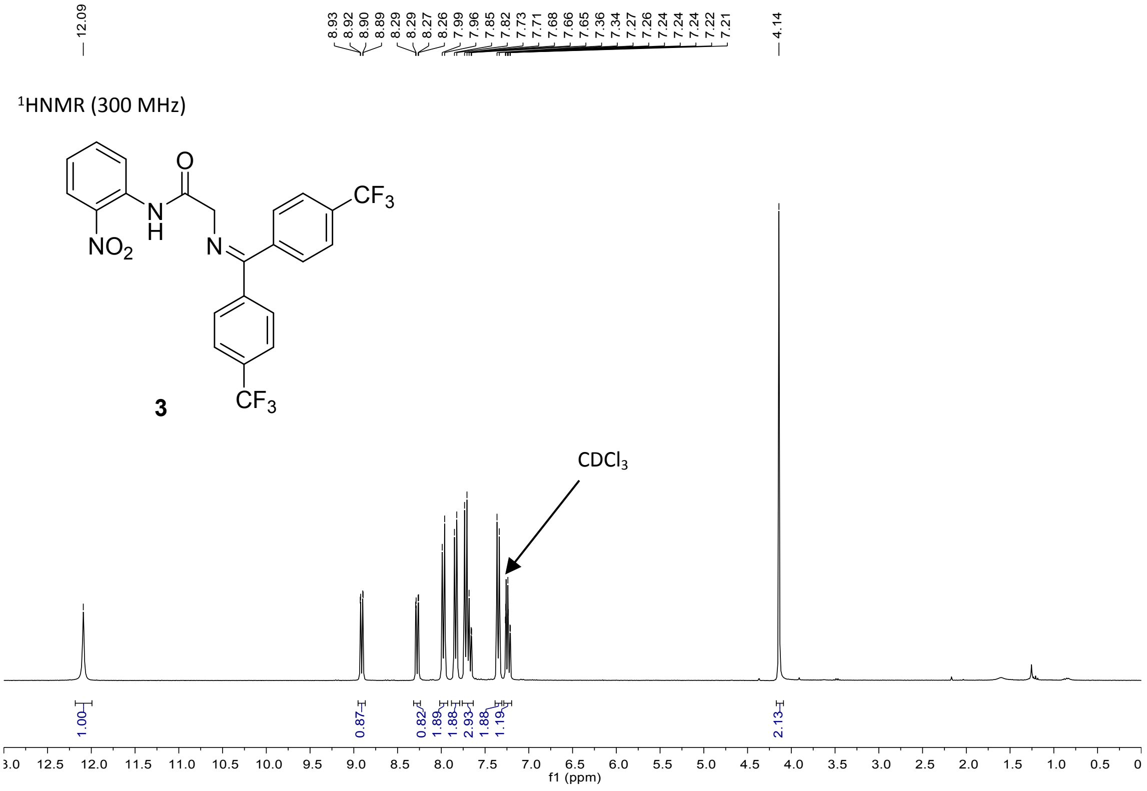
12.09
8.93 8.92 8.90 8.89 8.29 8.29 8.27 8.26 7.99 7.96 7.85 7.82 7.73 7.71 7.68 7.66 7.65 7.36 7.34 7.27 7.26 7.24 7.24 7.24 7.22 7.21
4.14
1HNMR (300 MHz)
NO2
CF3
3
CDCl3
1.00 0.87 0.82 1.89 1.88 2.93 1.88 1.19 2.13
f1 (ppm)

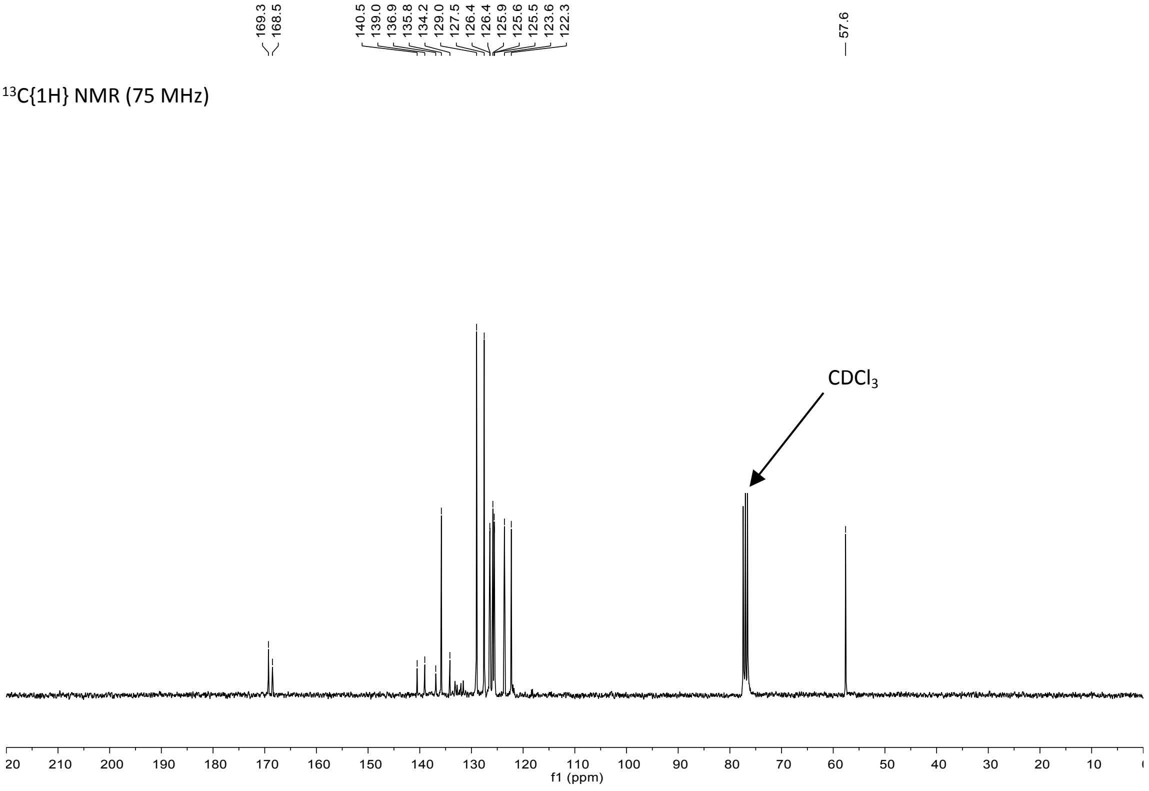
169.3 168.5
140.5 139.0 136.9 135.8 134.2 129.0 127.5 126.4 126.4 125.9 125.6 125.5 123.6 122.3
57.6
13C{1H} NMR (75 MHz)
CDCl3
f1 (ppm)

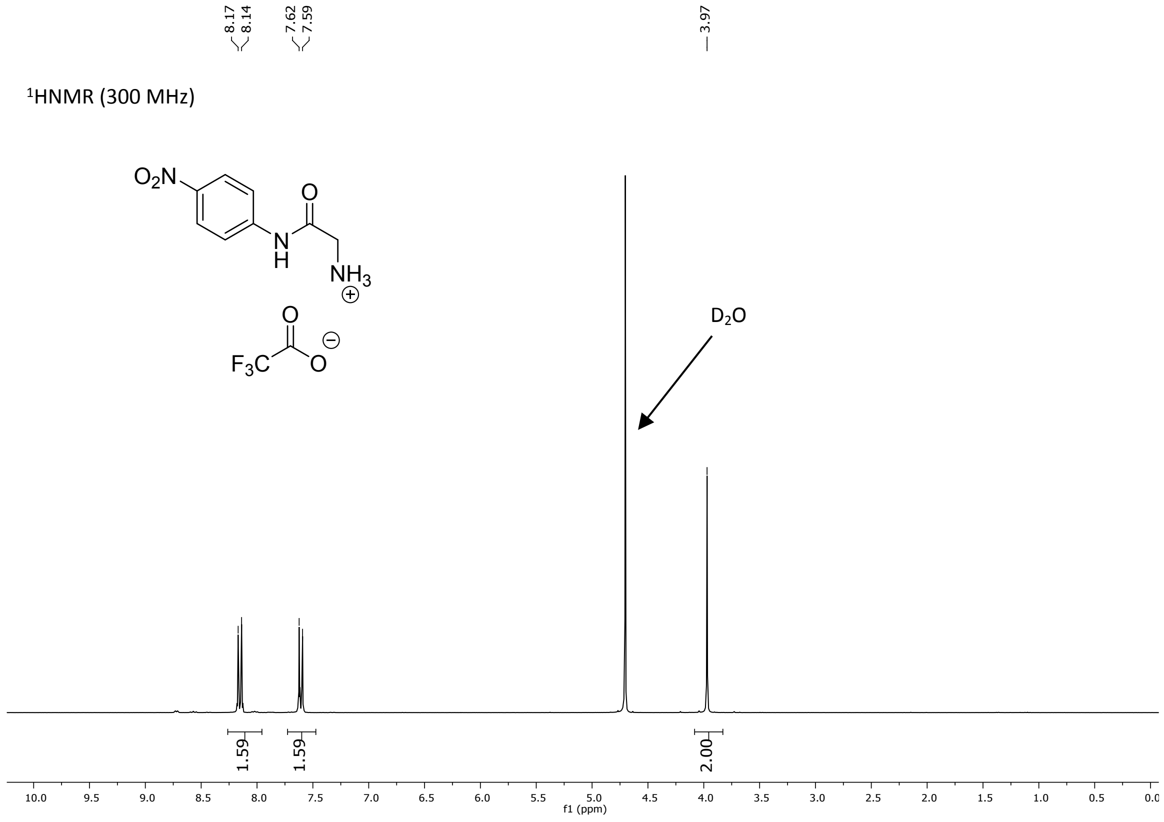
8.17
8.14
7.62
7.59
3.97
1HNMR (300 MHz)
O_2N
O
N
H
NH_3
F_3C
O
O
D_2O
1.59
1.59
2.00
10.0 9.5 9.0 8.5 8.0 7.5 7.0 6.5 6.0 5.5 5.0 4.5 4.0 3.5 3.0 2.5 2.0 1.5 1.0 0.5 0.0
f1 (ppm)

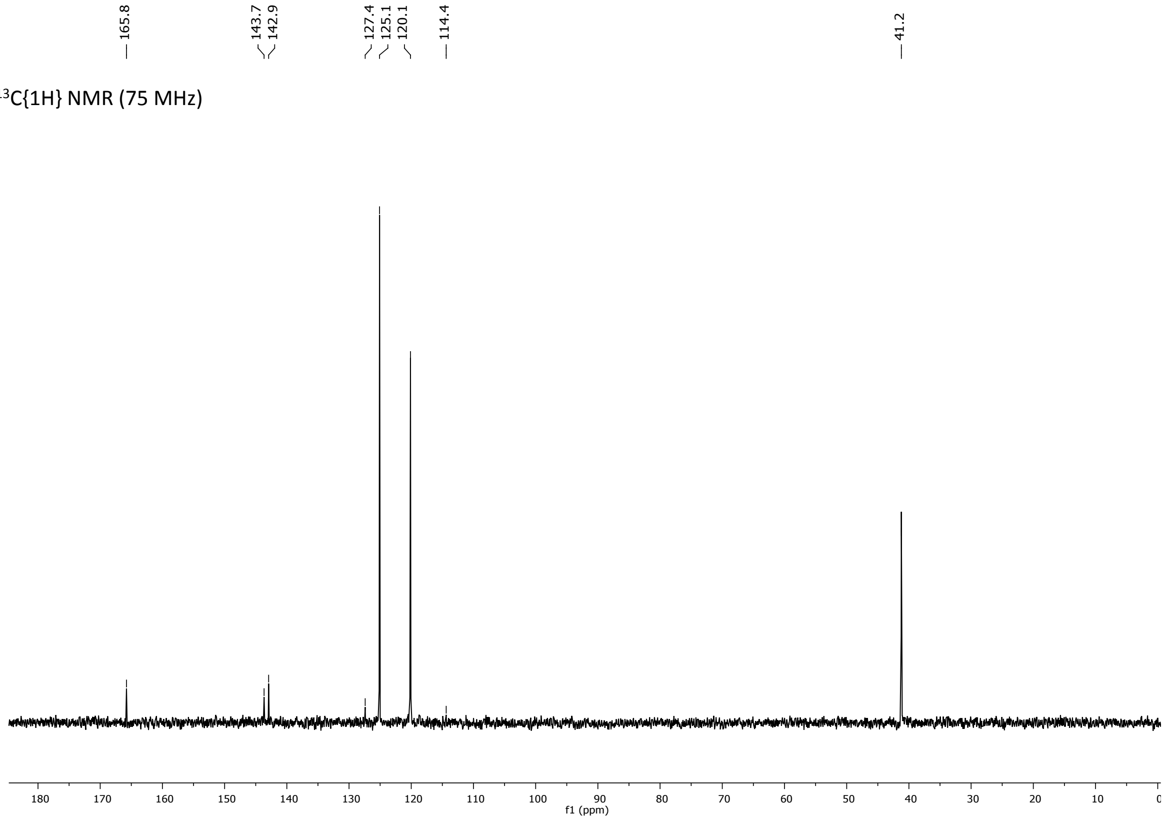
165.8
143.7
142.9
127.4
125.1
120.1
114.4
41.2
13C{1H} NMR (75 MHz)
180 170 160 150 140 130 120 110 100 90 80 70 60 50 40 30 20 10 0
f1 (ppm)

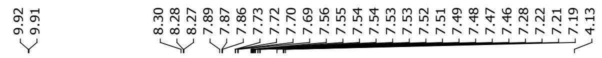

^{1}HNMR (300 MHz)

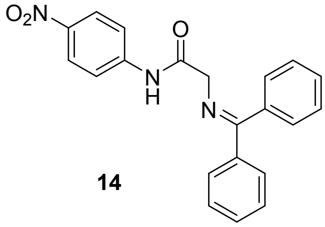

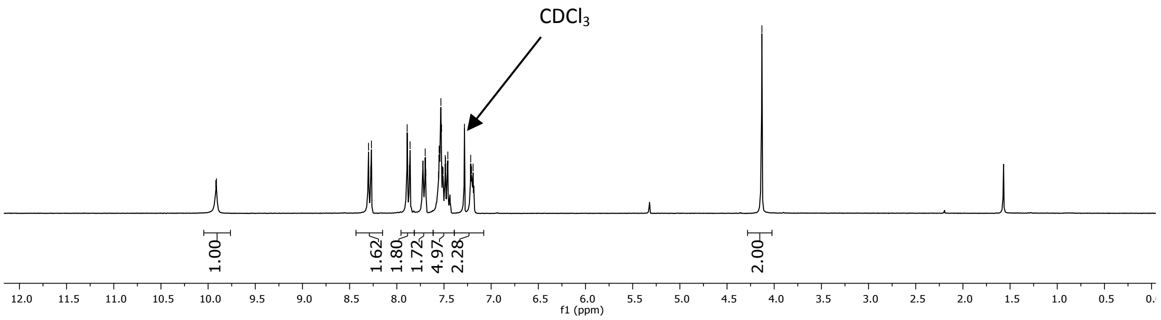

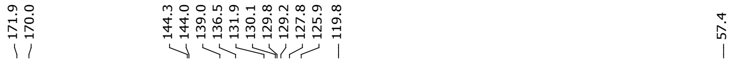

^{13}C{1H} NMR (75 MHz)

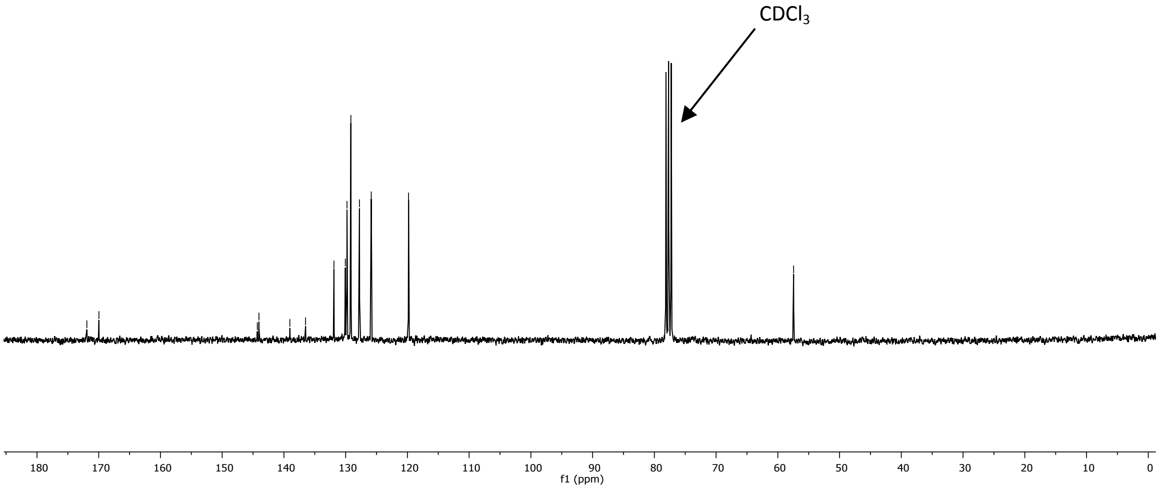

^{1}HNMR (300 Hz)

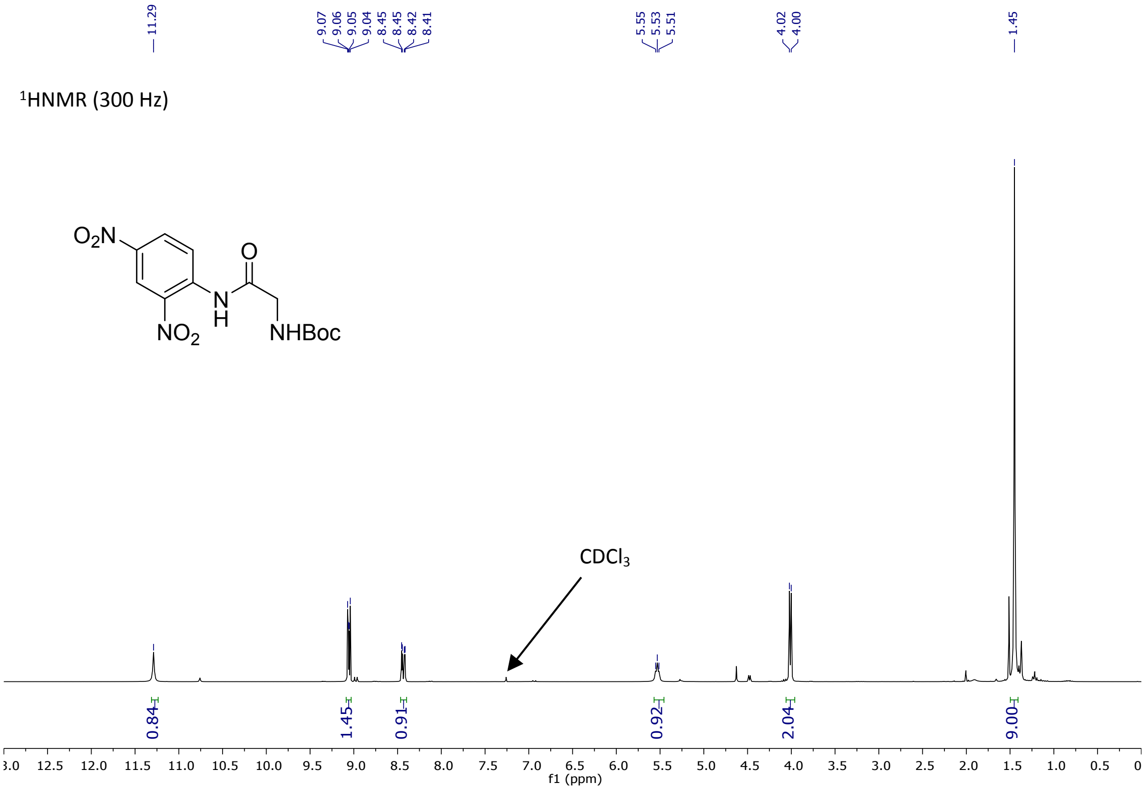

^{13}C{1H} NMR (75 MHz)

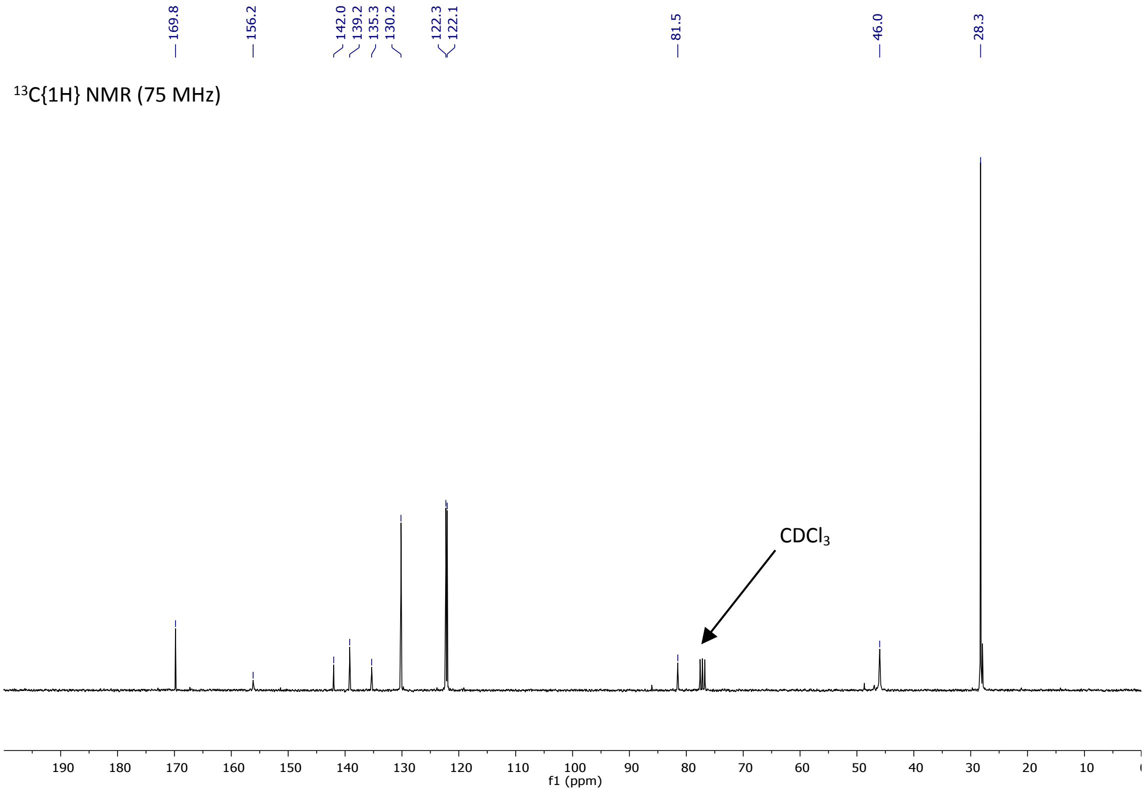

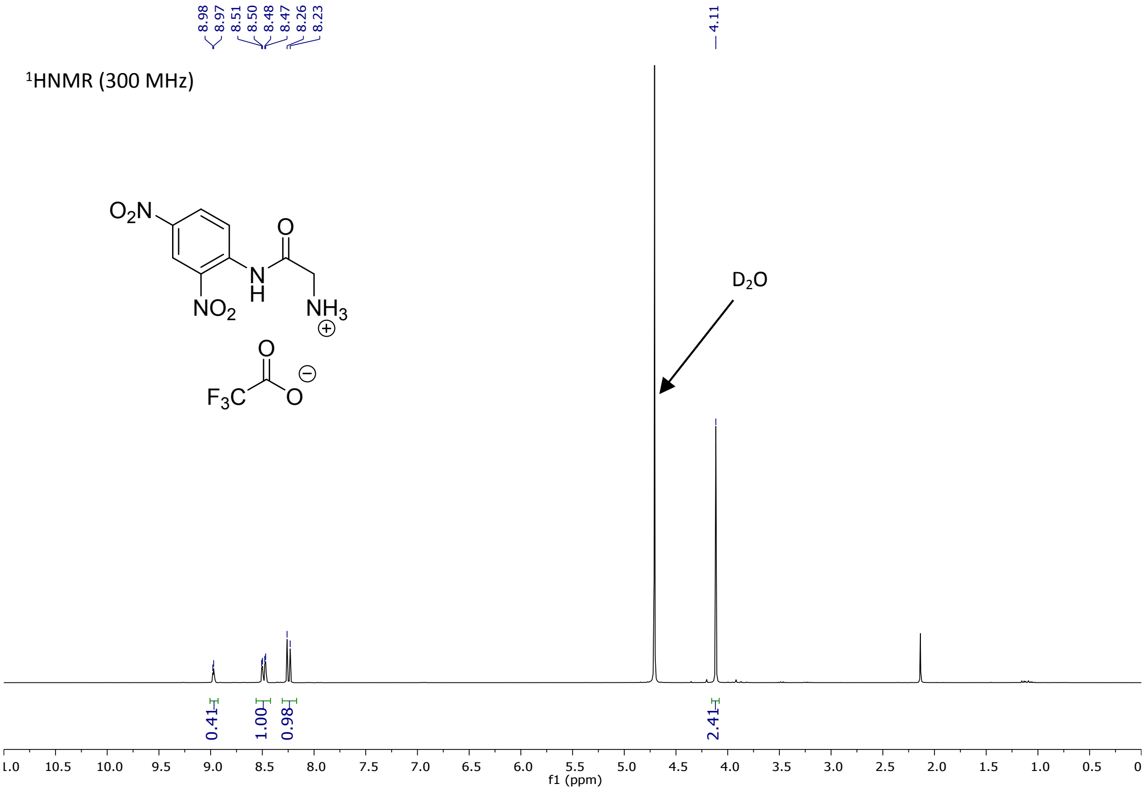

^{1}HNMR (300 MHz)
8.98
8.97
8.51
8.50
8.48
8.47
8.26
8.23
4.11
D_2O
0.41
1.00
0.98
2.41
f1 (ppm)

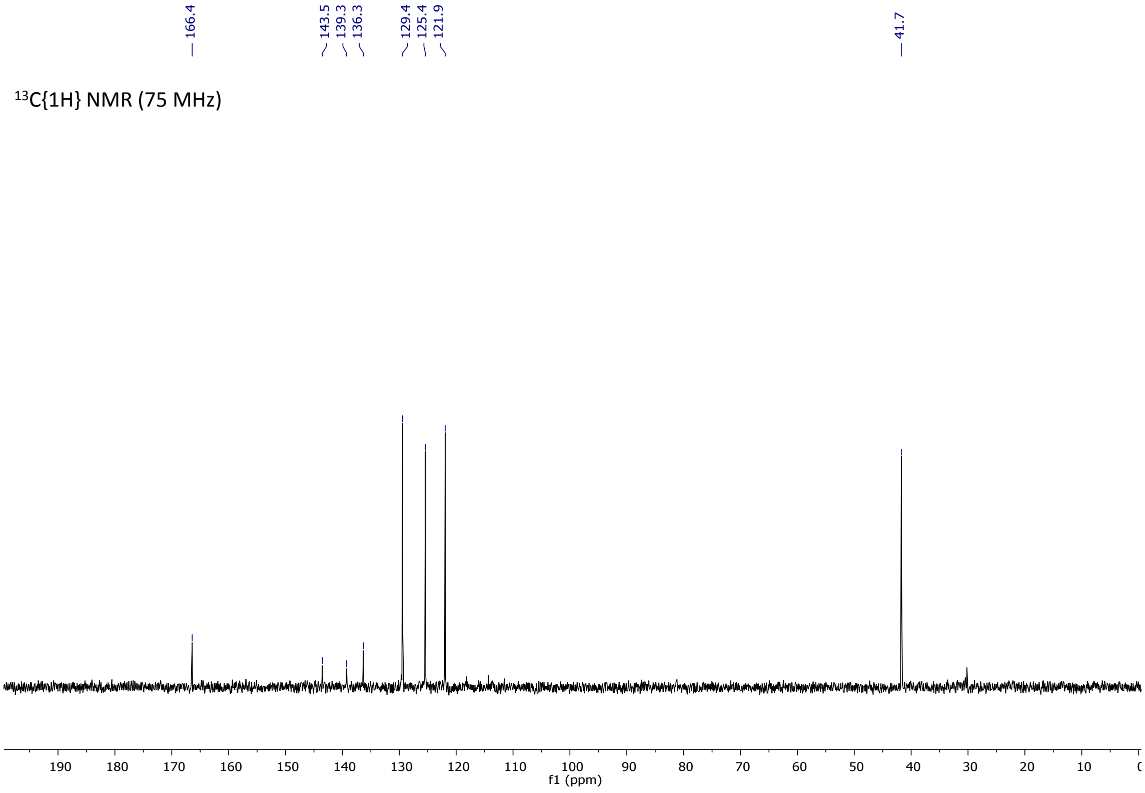

^{13}C{1H} NMR (75 MHz)
166.4
143.5
139.3
136.3
129.4
125.4
121.9
41.7
f1 (ppm)

^{1}HNMR (300 MHz)

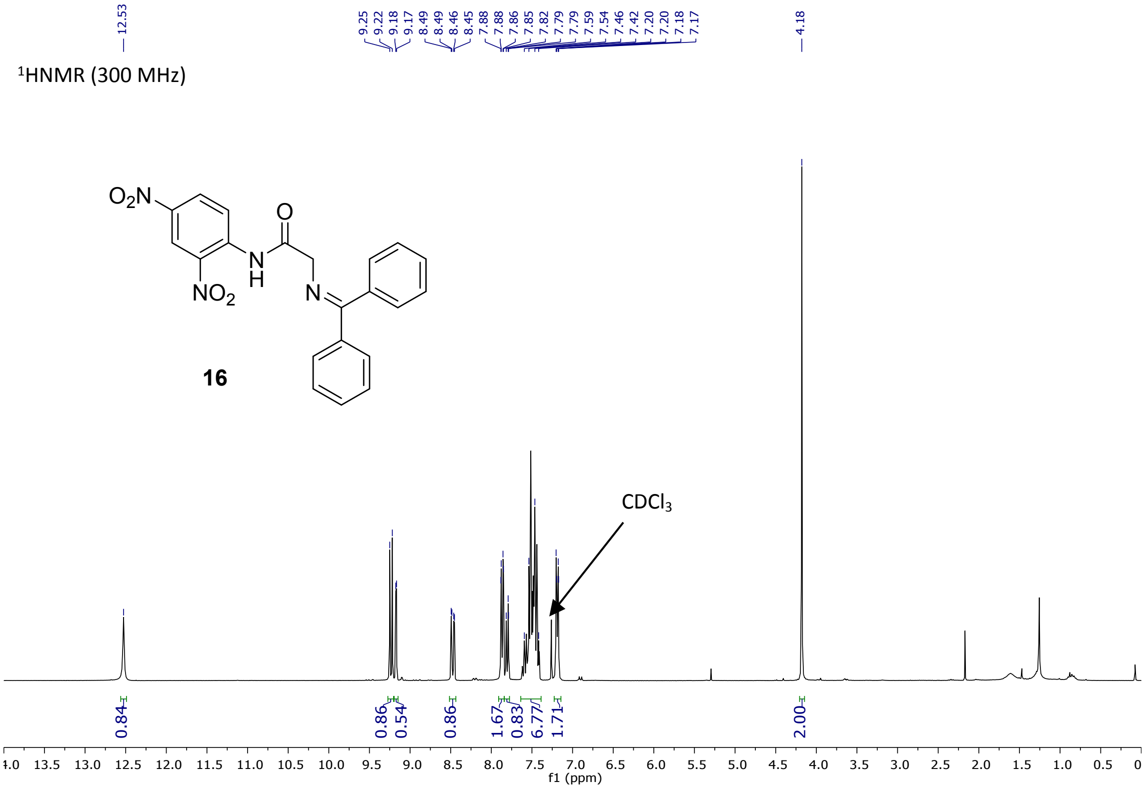

^{13}C{1H} NMR (75 MHz)

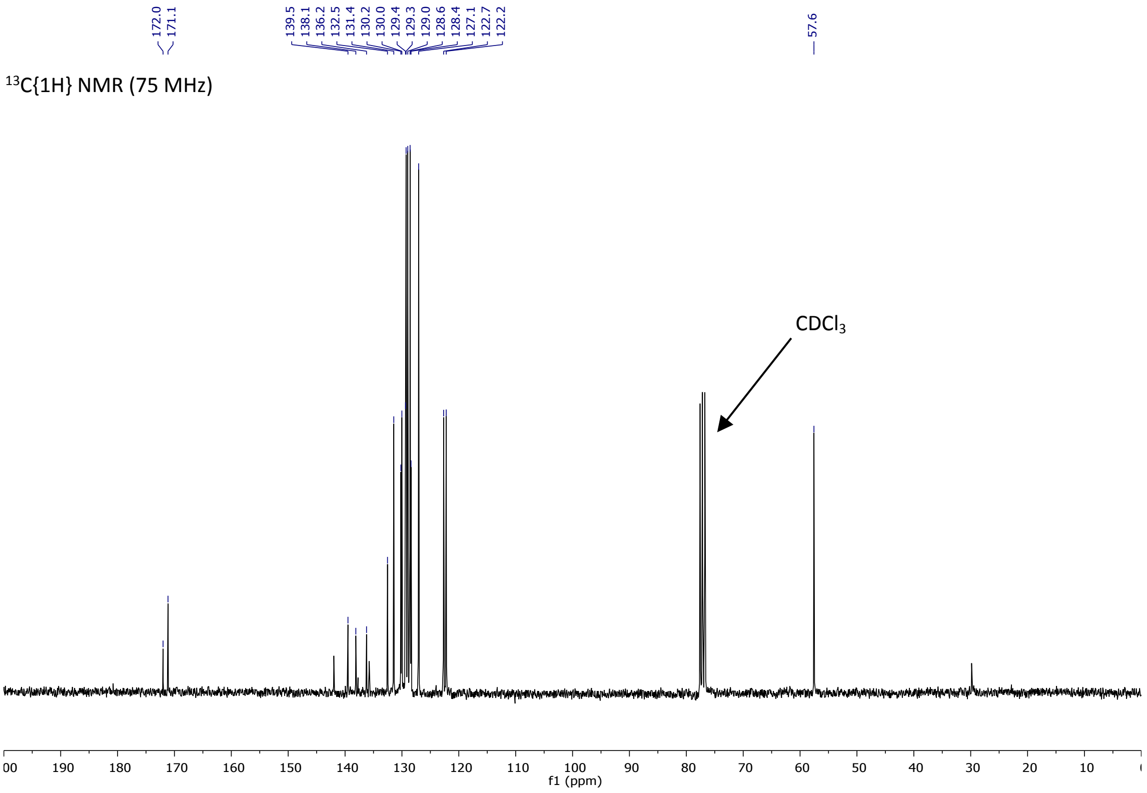

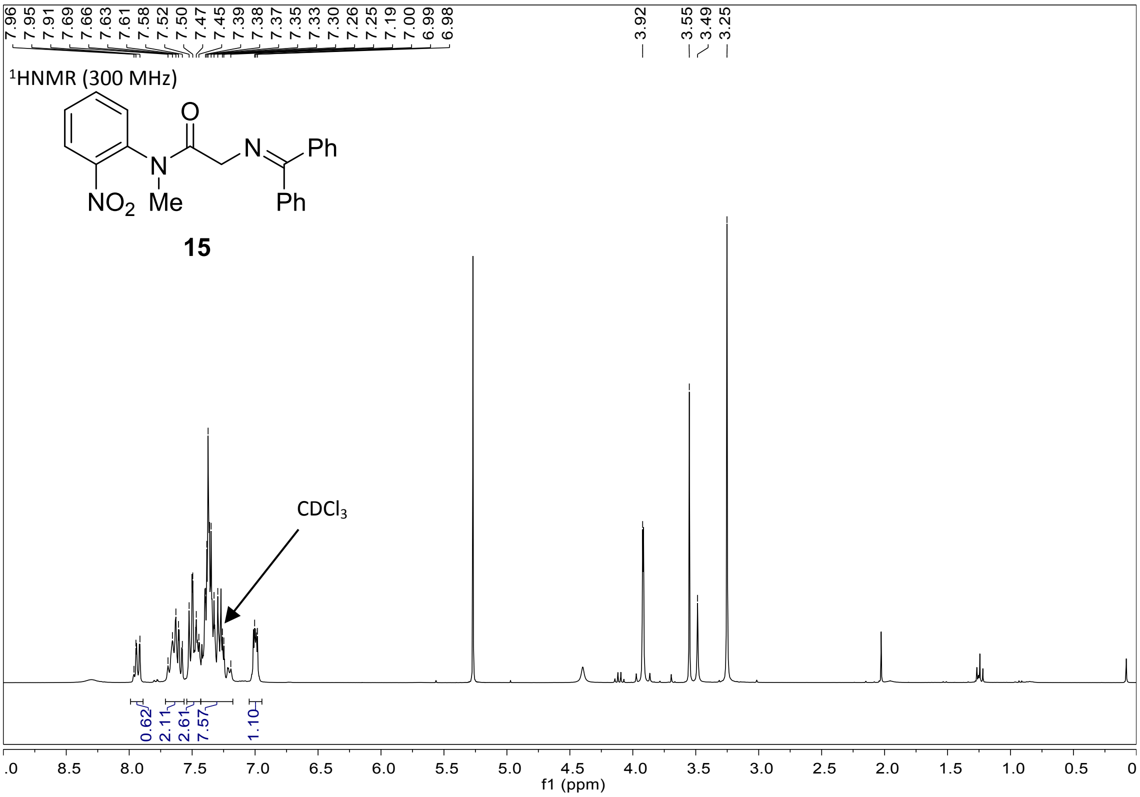
7.96 7.95 7.91 7.69 7.66 7.63 7.61 7.58 7.52 7.50 7.47 7.45 7.39 7.38 7.37 7.35 7.33 7.30 7.26 7.25 7.19 7.00 6.99 6.98
3.92
3.55 3.49 3.25
1HNMR (300 MHz)
O
N
Ph
N
NO2 Me
Ph
15
CDCl3
0.62 2.11 2.61 7.57
1.10
f1 (ppm)

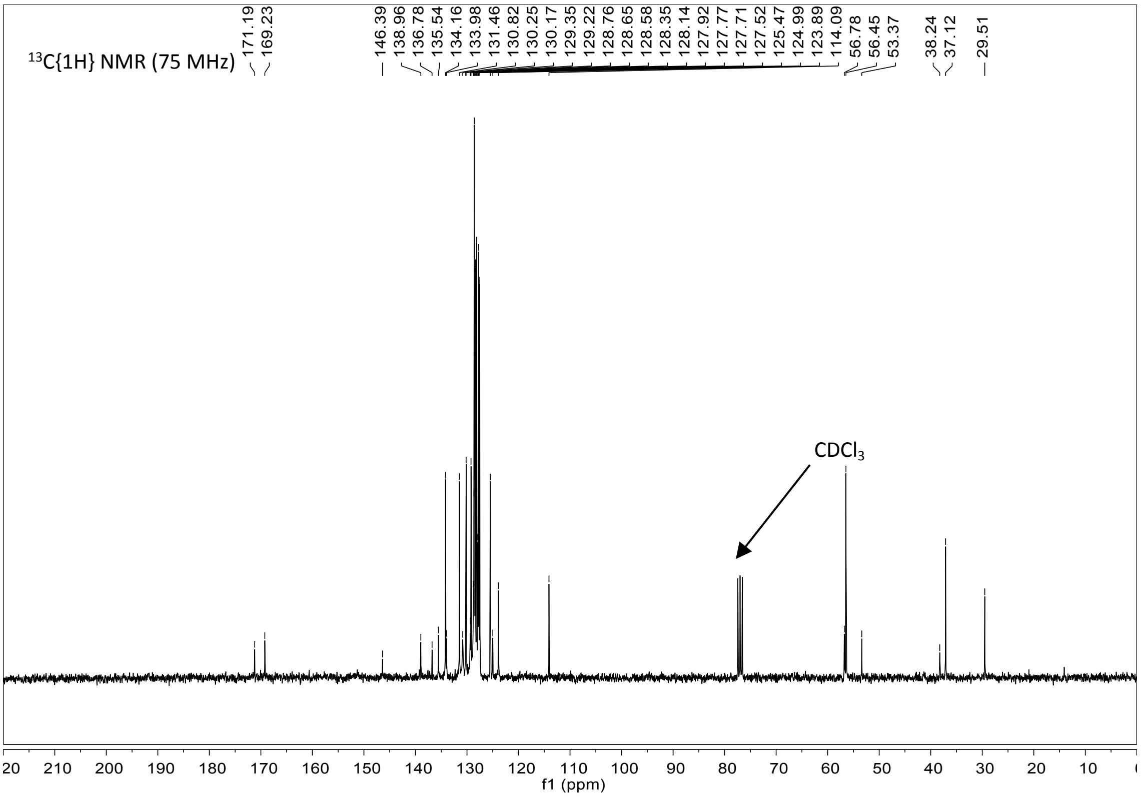
171.19 169.23
146.39 138.96 136.78 135.54 134.16 133.98 131.46 130.82 130.25 130.17 129.35 129.22 128.76 128.65 128.58 128.35 128.14 127.92 127.77 127.71 127.52 125.47 124.99 123.89 114.09
56.78 56.45 53.37
38.24 37.12
29.51
13C{1H} NMR (75 MHz)
CDCl3
f1 (ppm)

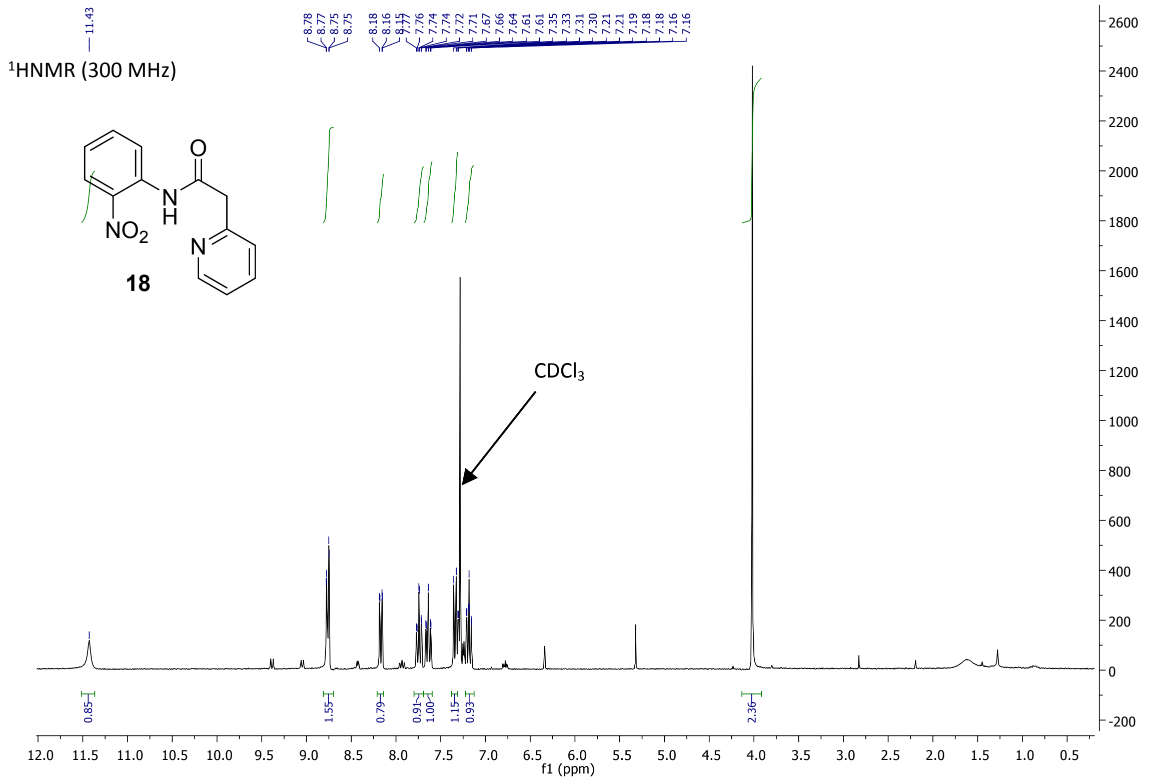

1HNMR (300 MHz)
18
CDCl3
f1 (ppm)

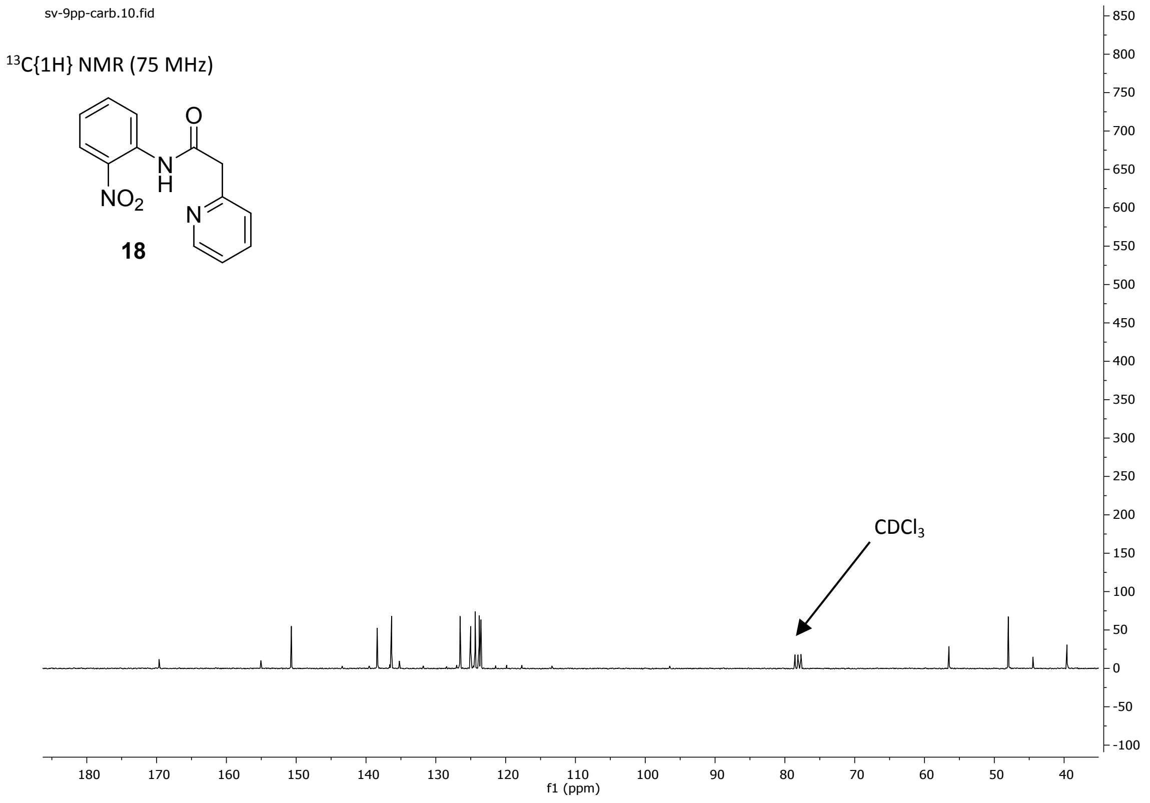

sv-9pp-carb.10.fid
13C{1H} NMR (75 MHz)
18
CDCl3
f1 (ppm)

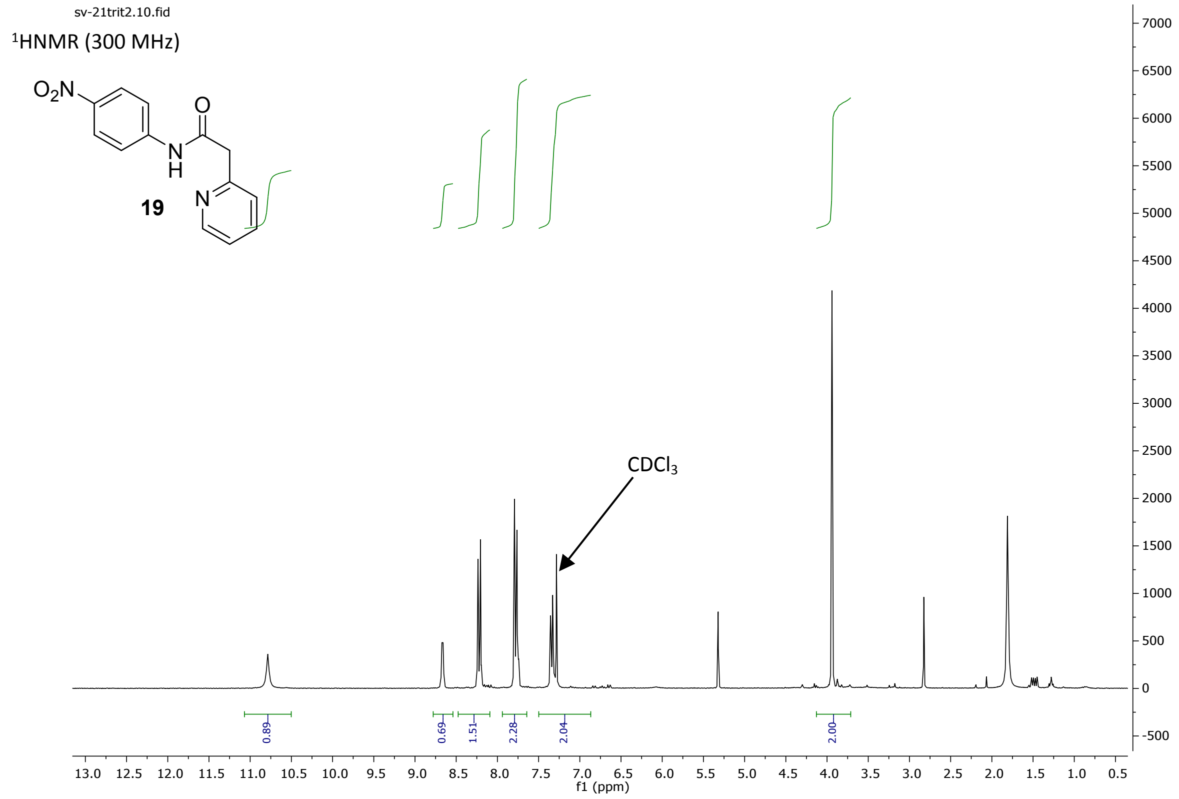
sv-21trit2.10.fid
1HNMR (300 MHz)
O2N
O
N
H
N
19
CDCl3
0.89
0.69
1.51
2.28
2.04
2.00
f1 (ppm)

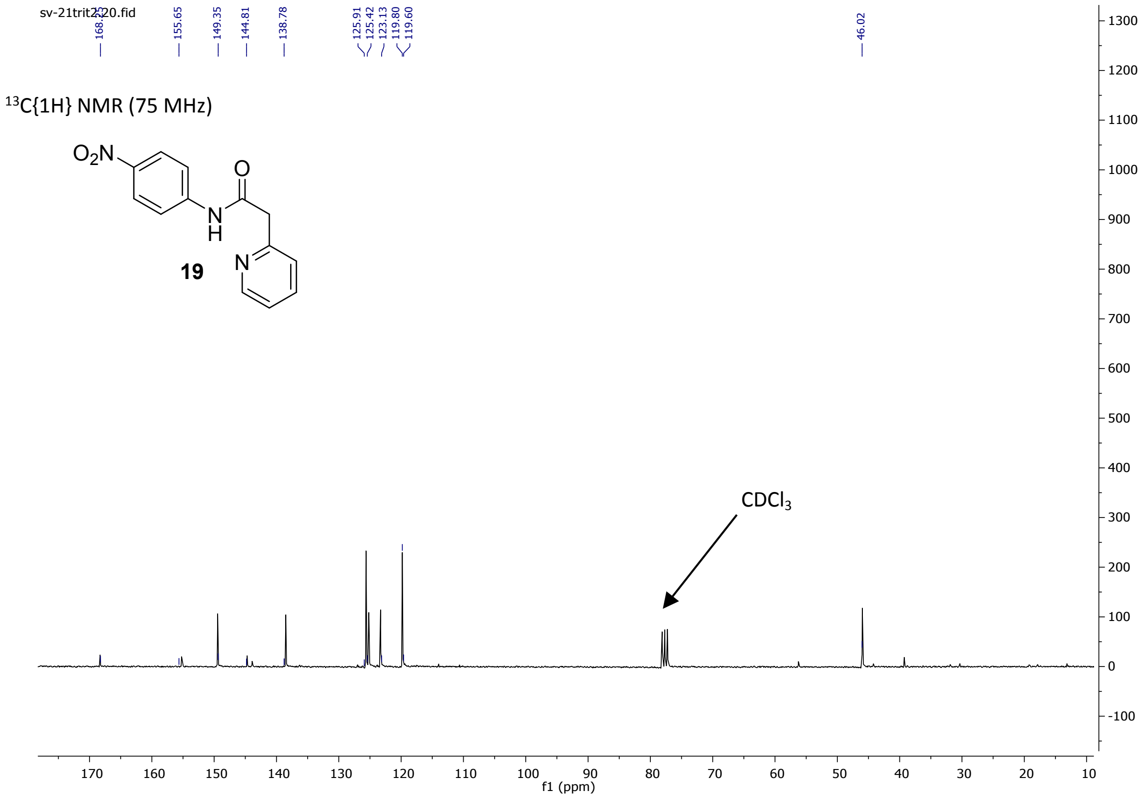
sv-21trit2.20.fid
168.22
155.65
149.35
144.81
138.78
125.91
125.42
123.13
119.80
119.60
46.02
13C{1H} NMR (75 MHz)
O2N
O
N
H
N
19
CDCl3
f1 (ppm)

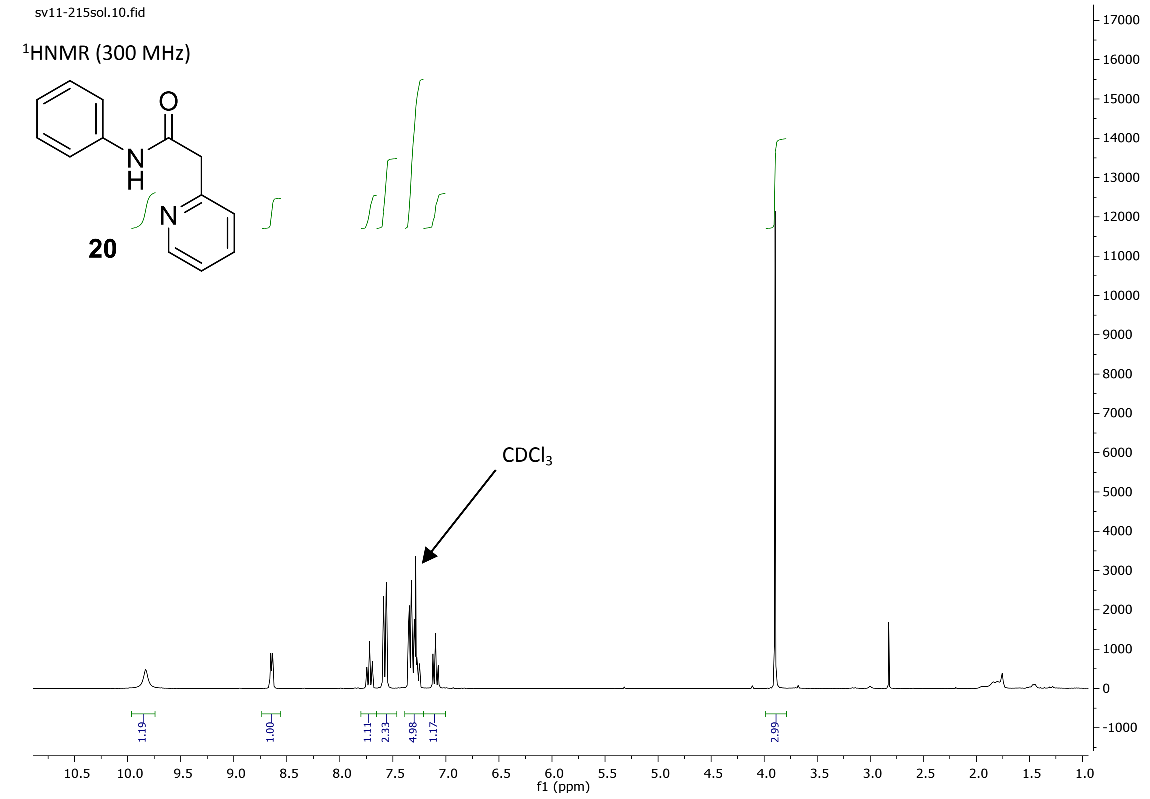
sv11-215sol.10.fid
^{1}HNMR (300 MHz)
20
CDCl$_3$
1.19
1.00
1.11
2.33
4.98
1.17
2.99
f1 (ppm)

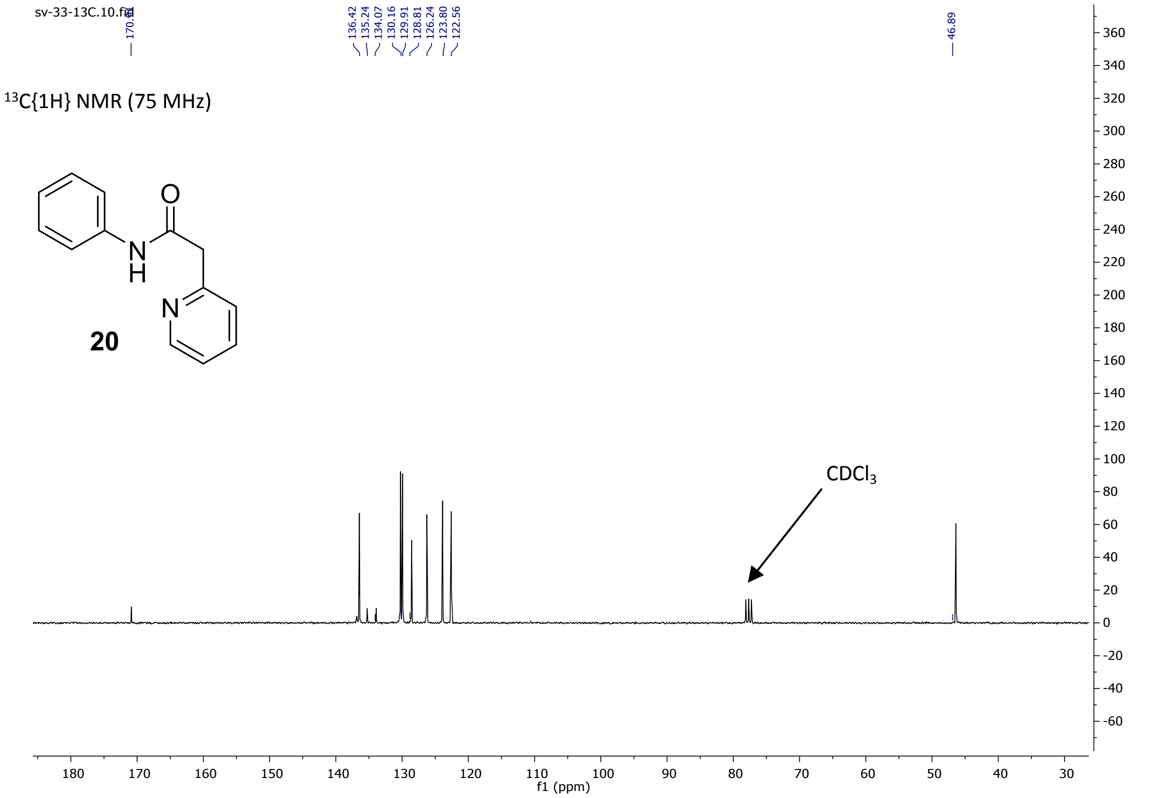
sv-33-13C.10.fid
170.91
136.42
135.24
134.07
130.16
129.91
128.81
126.24
123.80
122.56
46.89
^{13}C{1H} NMR (75 MHz)
20
CDCl$_3$
f1 (ppm)

^{1}HNMR (300 MHz)

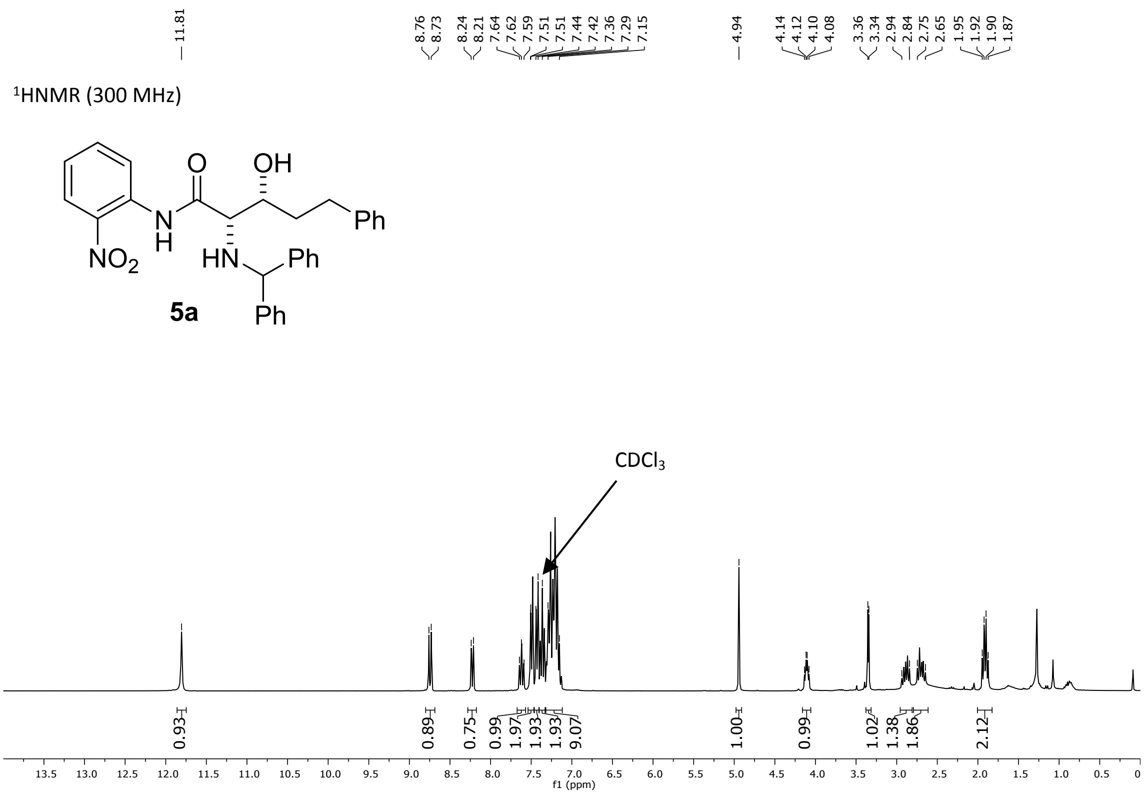

^{13}C{1H} NMR (75 MHz)

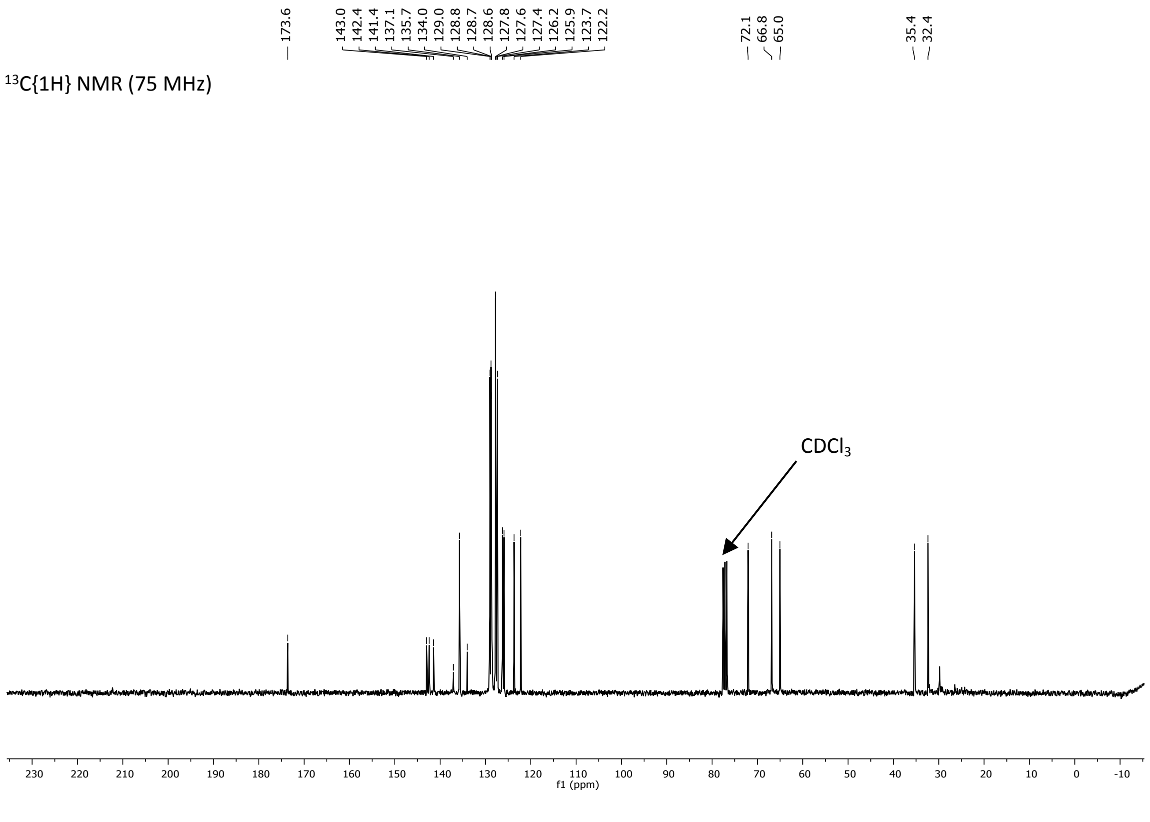

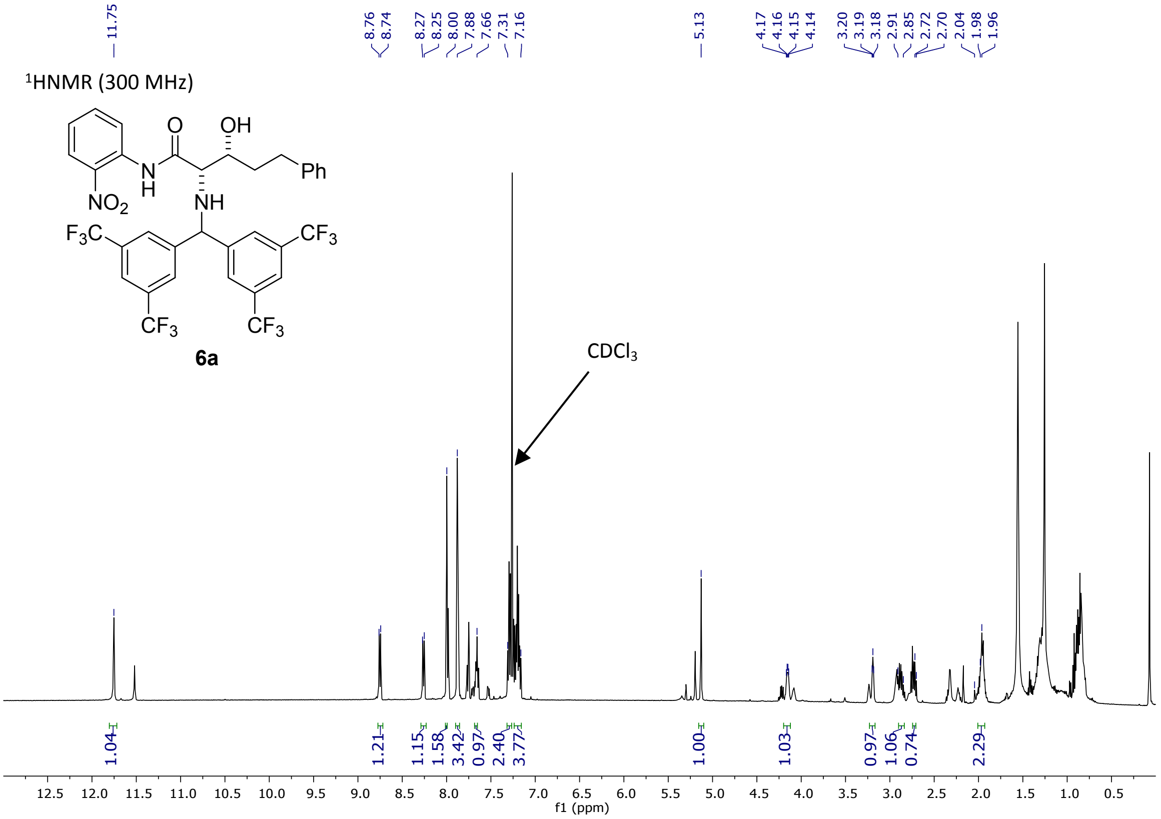
1HNMR (300 MHz)
O
OH
N
H
Ph
NO2
NH
F3C
CF3
CF3
CF3
6a
CDCl3
11.75
8.76
8.74
8.27
8.25
8.00
7.88
7.66
7.31
7.16
5.13
4.17
4.16
4.15
4.14
3.20
3.19
3.18
2.91
2.85
2.72
2.70
2.04
1.98
1.96
1.04
1.21
1.15
1.58
3.42
0.97
2.40
3.77
1.00
1.03
0.97
1.06
0.74
2.29
f1 (ppm)

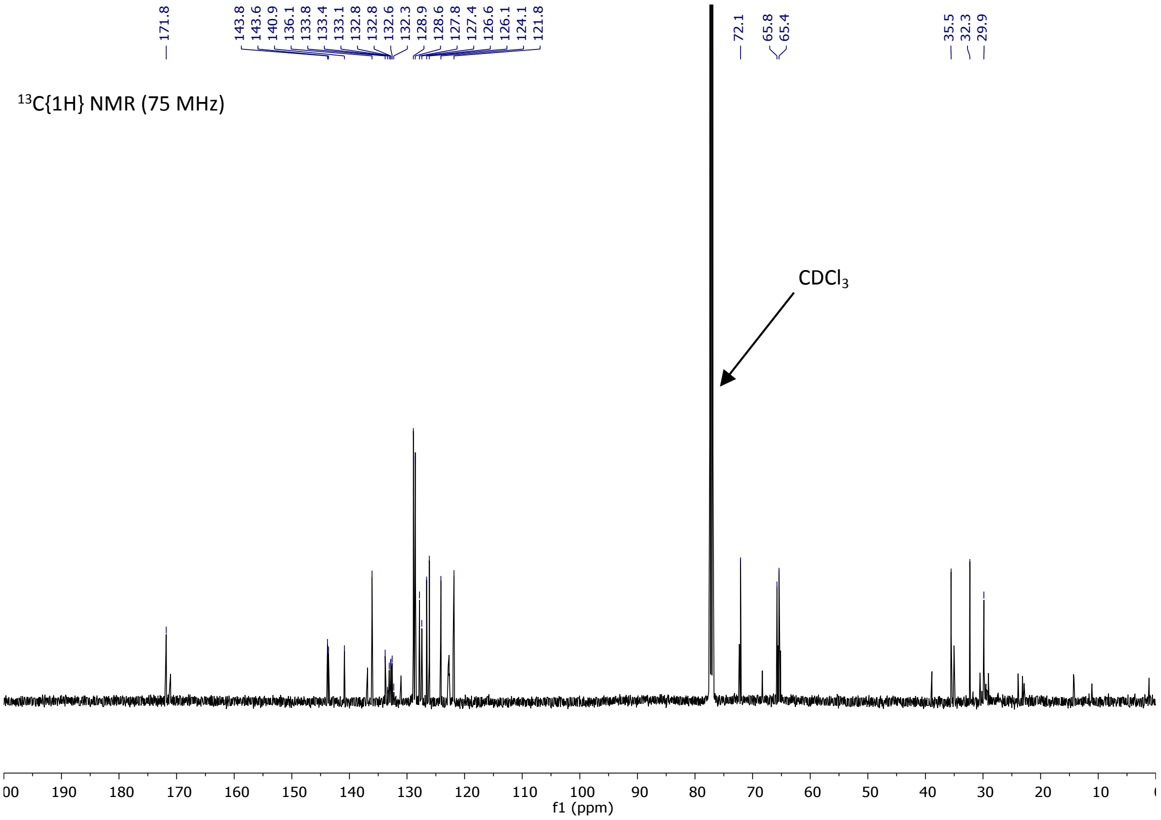
13C{1H} NMR (75 MHz)
CDCl3
171.8
143.8
143.6
140.9
136.1
133.8
133.4
133.1
132.8
132.6
132.3
128.9
128.6
127.8
127.4
126.6
126.1
124.1
121.8
72.1
65.8
65.4
35.5
32.3
29.9
f1 (ppm)

^{1}HNMR (300 MHz)

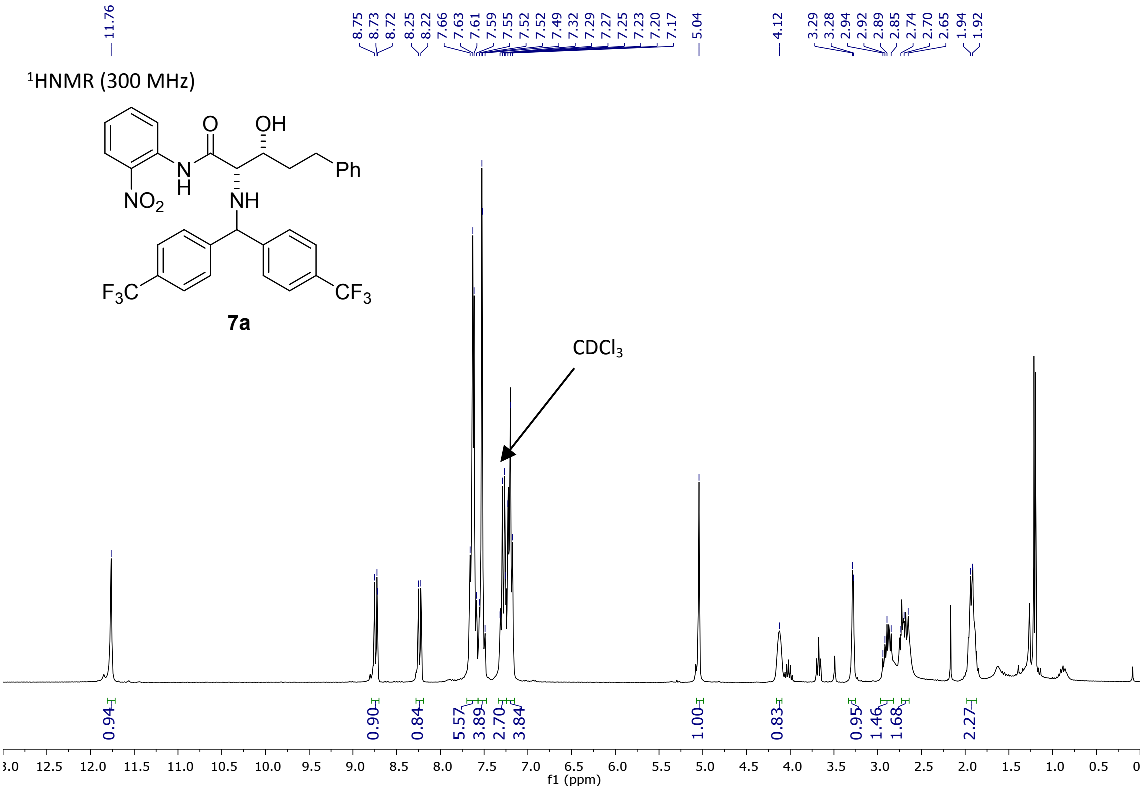

^{13}C{1H} NMR (75 MHz)

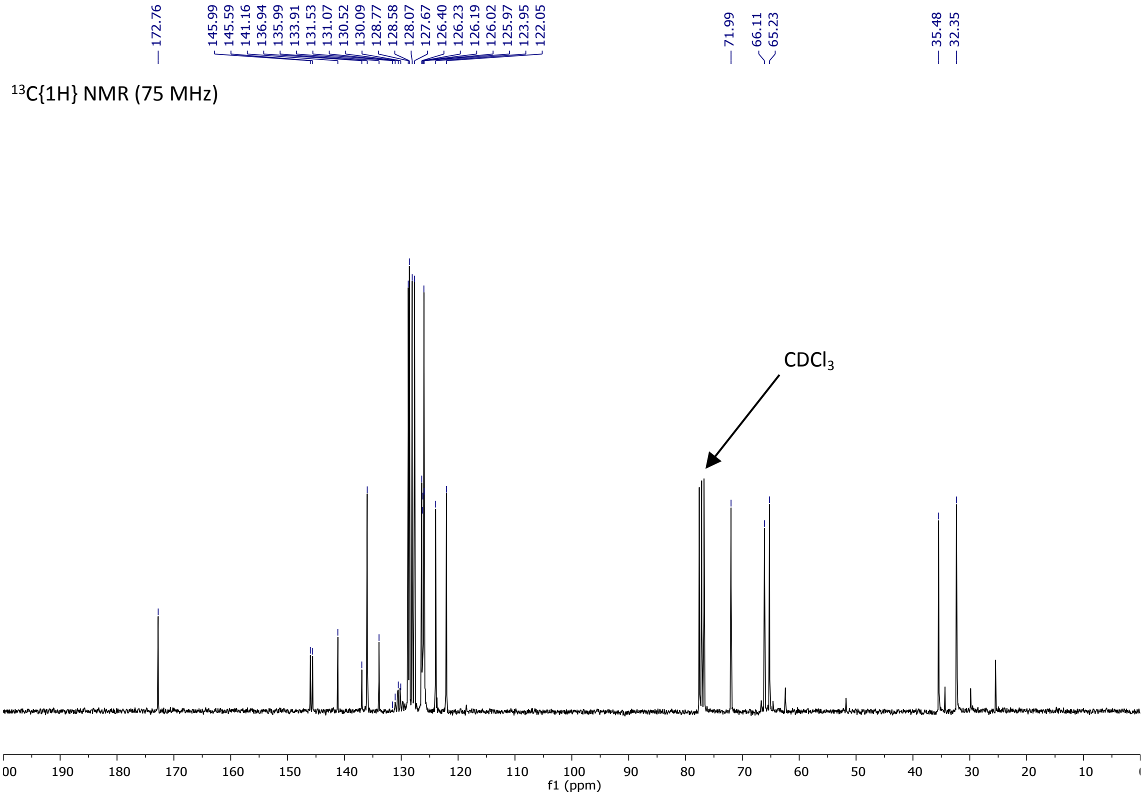

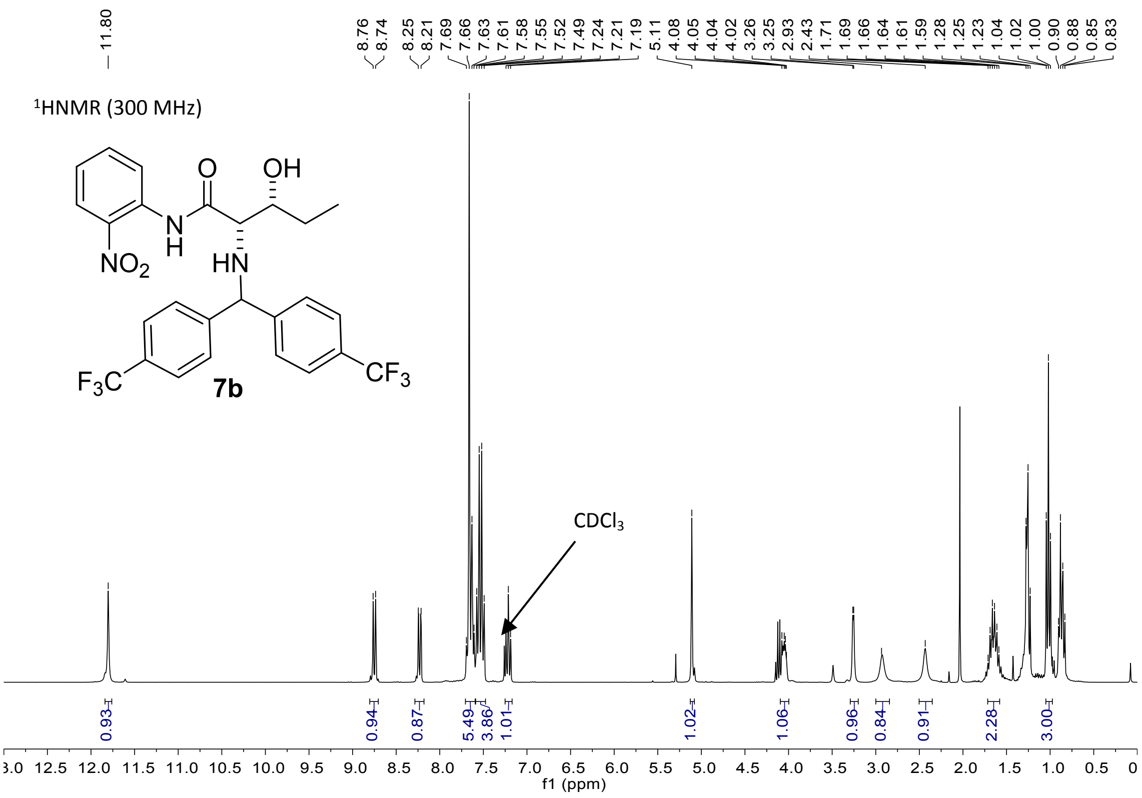
11.80
8.76 8.74 8.25 8.21 7.69 7.66 7.63 7.61 7.58 7.55 7.52 7.49 7.24 7.21 7.19 5.11 4.08 4.05 4.04 4.02 3.26 3.25 2.93 2.43 1.71 1.69 1.66 1.64 1.61 1.59 1.28 1.25 1.23 1.04 1.02 1.00 0.90 0.88 0.85 0.83
1HNMR (300 MHz)
O
OH
N
H
NO2
HN
F3C
CF3
7b
CDCl3
0.93 0.94 0.87 5.49 3.86 1.01 1.02 1.06 0.96 0.84 0.91 2.28 3.00
f1 (ppm)

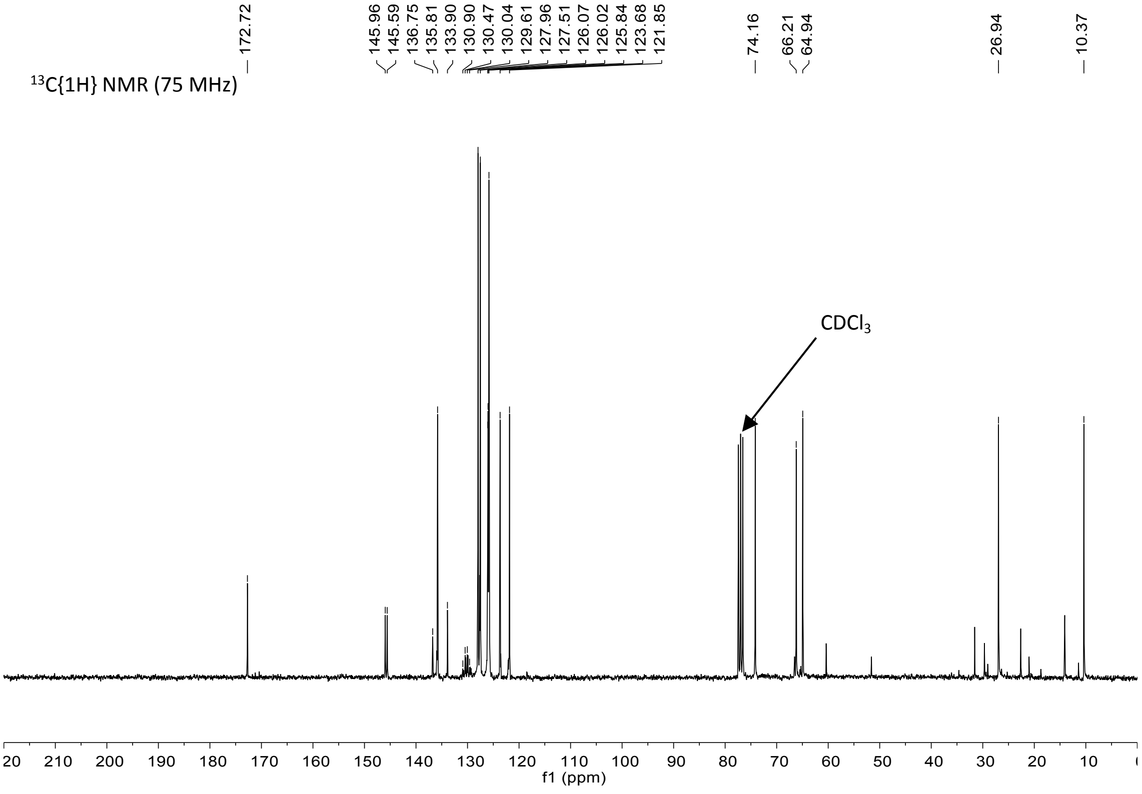
172.72
145.96 145.59 136.75 135.81 133.90 130.90 130.47 130.04 129.61 127.96 127.51 126.07 126.02 125.84 123.68 121.85
74.16 66.21 64.94
26.94
10.37
13C{1H} NMR (75 MHz)
CDCl3
f1 (ppm)

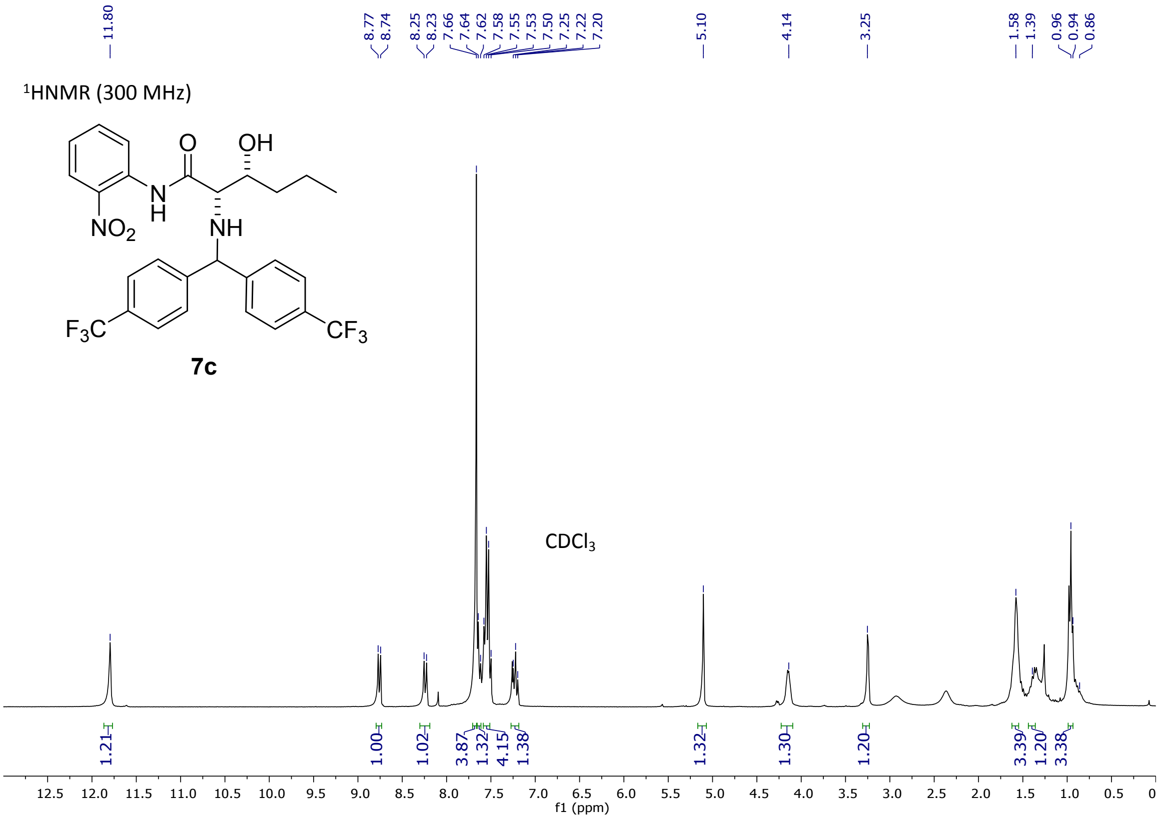
11.80
8.77
8.74
8.25
8.23
7.66
7.64
7.62
7.58
7.55
7.53
7.50
7.25
7.22
7.20
5.10
4.14
3.25
1.58
1.39
0.96
0.94
0.86
¹HNMR (300 MHz)
OH
O
N
H
NO2
NH
F3C
CF3
7c
CDCl3
1.21
1.00
1.02
3.87
1.32
4.15
1.38
1.32
1.30
1.20
3.39
1.20
3.38
f1 (ppm)

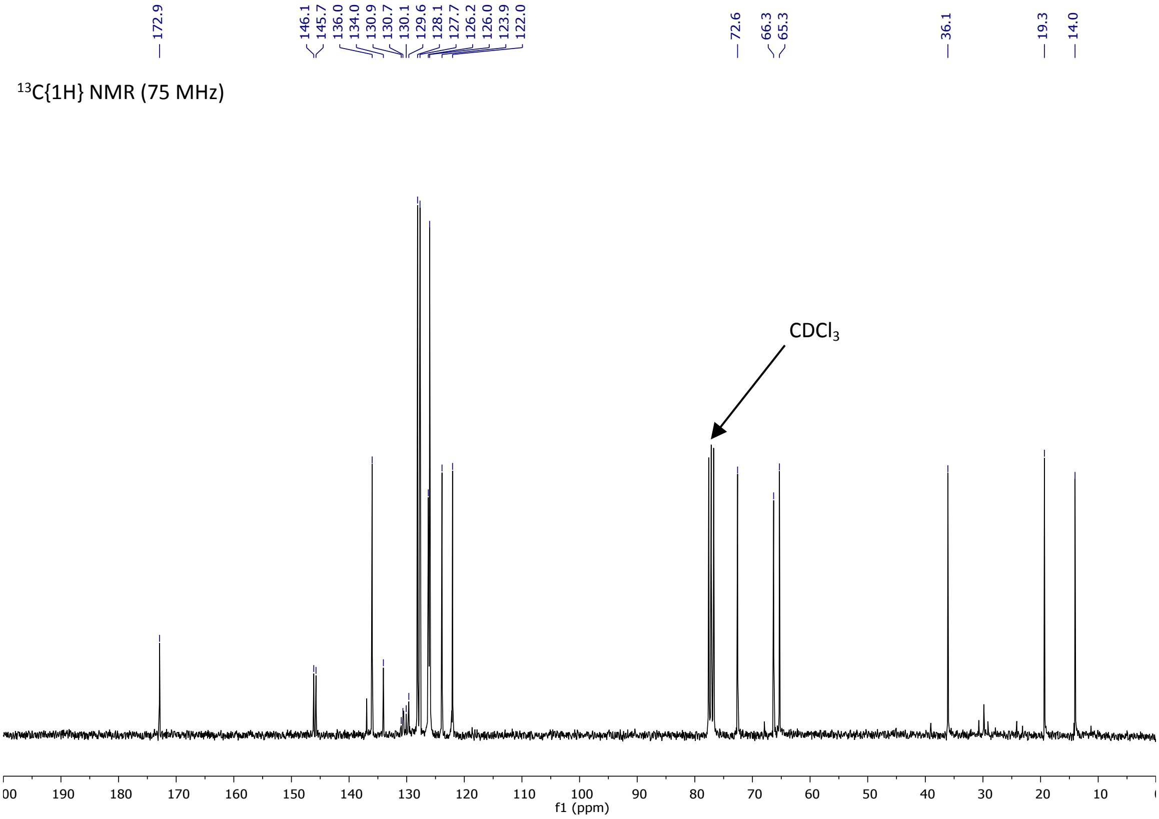
172.9
146.1
145.7
136.0
134.0
130.9
130.7
130.1
129.6
128.1
127.7
126.2
126.0
123.9
122.0
72.6
66.3
65.3
36.1
19.3
14.0
¹³C{1H} NMR (75 MHz)
CDCl3
f1 (ppm)

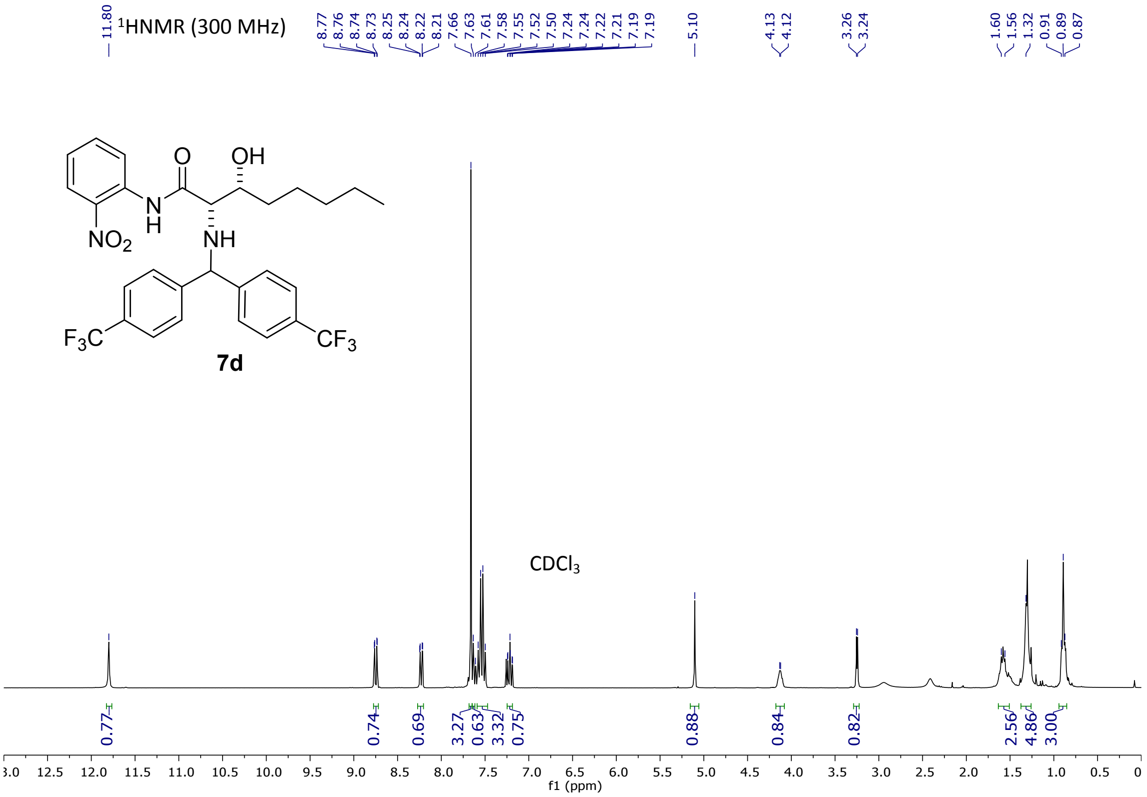

¹HNMR (300 MHz)
7d
CDCl₃
f1 (ppm)

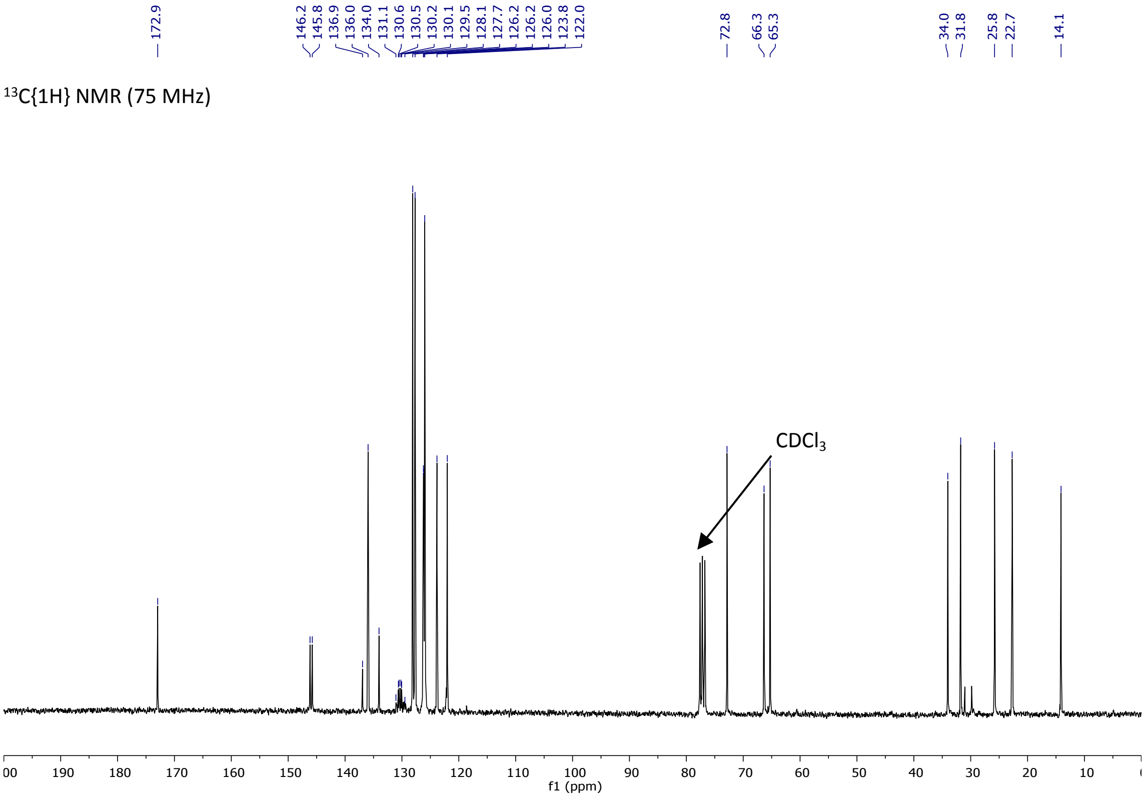

¹³C{1H} NMR (75 MHz)
CDCl₃
f1 (ppm)

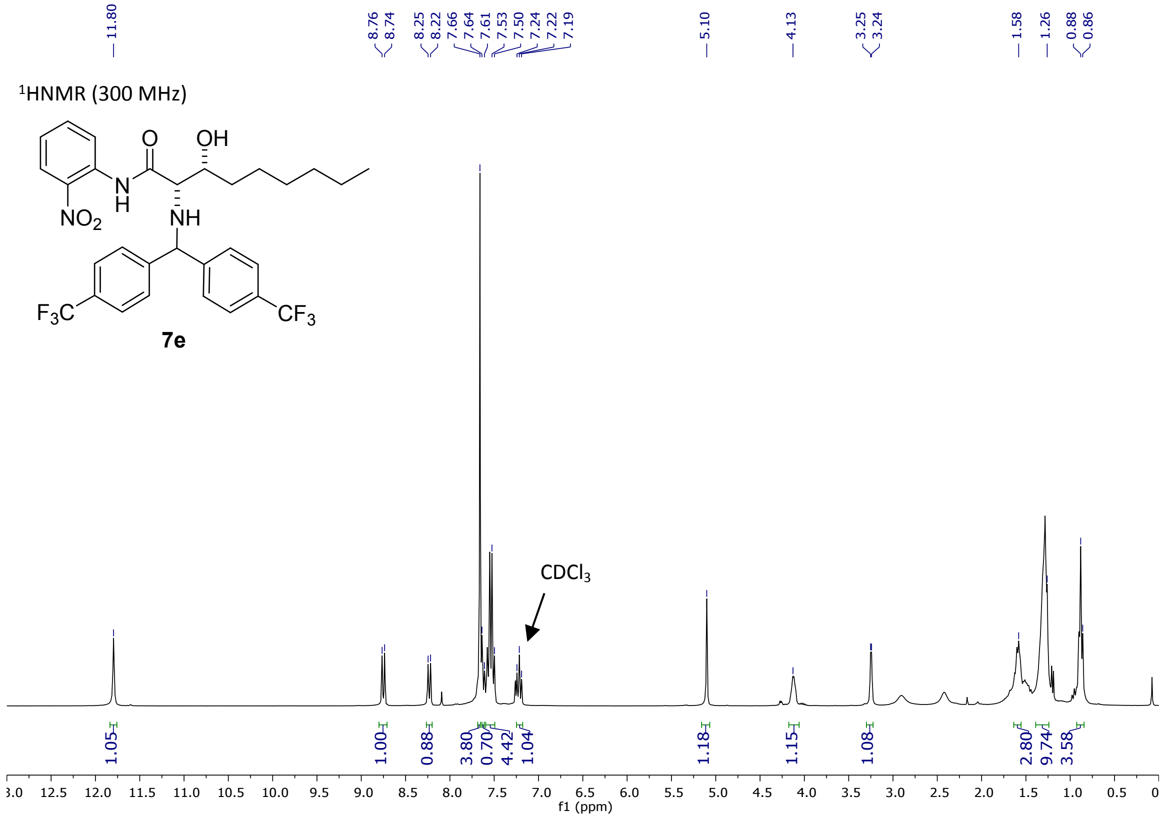

1HNMR (300 MHz)
7e
CDCl3
f1 (ppm)

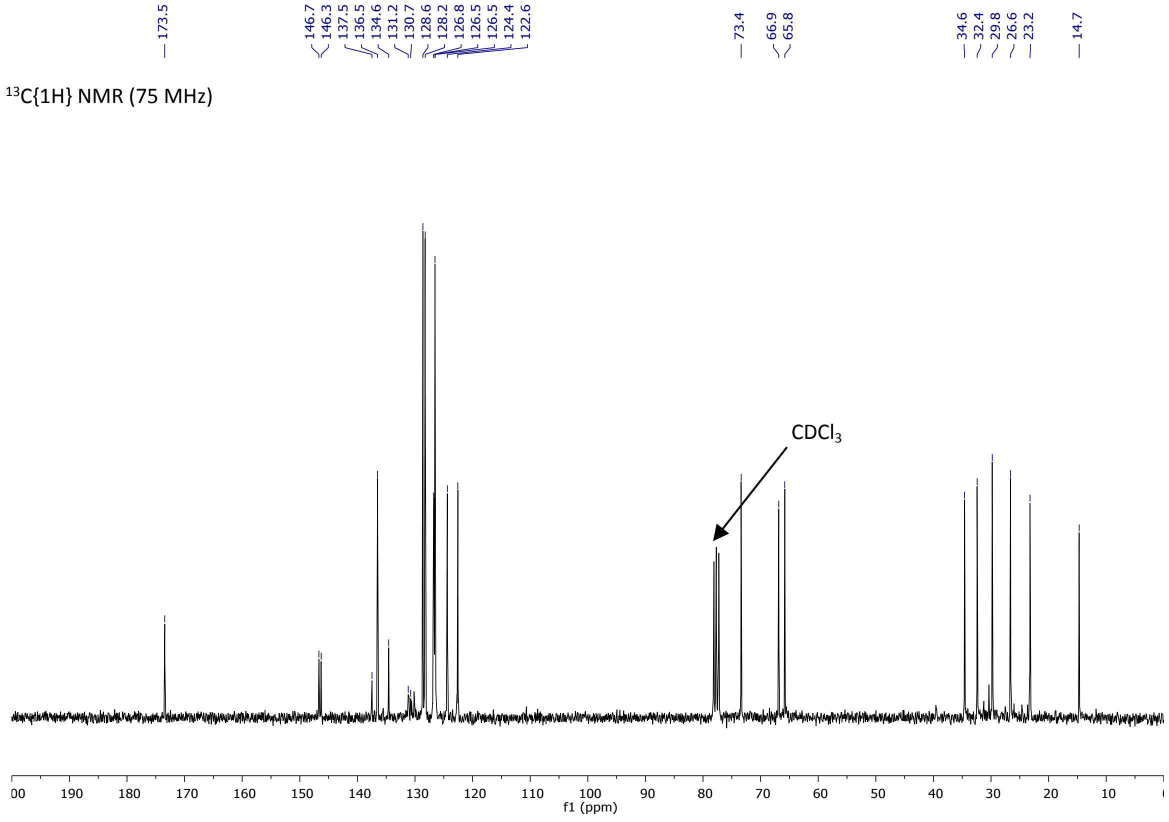

13C{1H} NMR (75 MHz)
CDCl3
f1 (ppm)

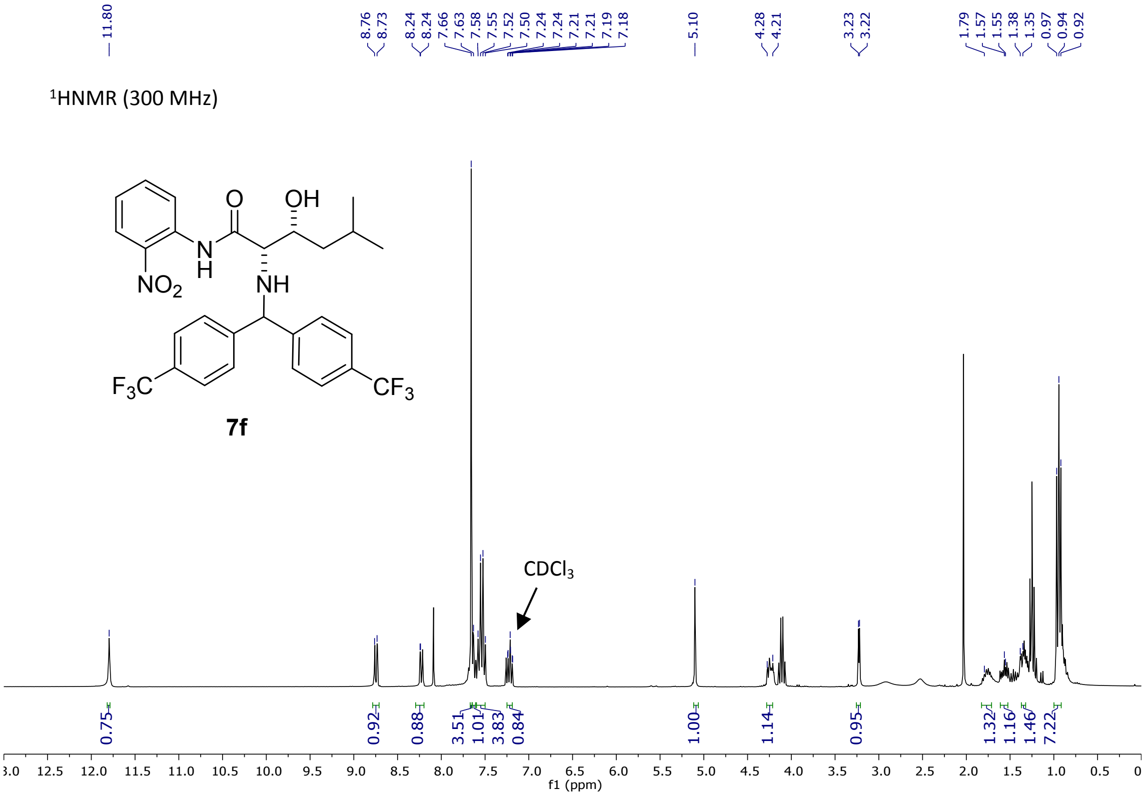

^{1}HNMR (300 MHz)
7f
CDCl$_3$
f1 (ppm)

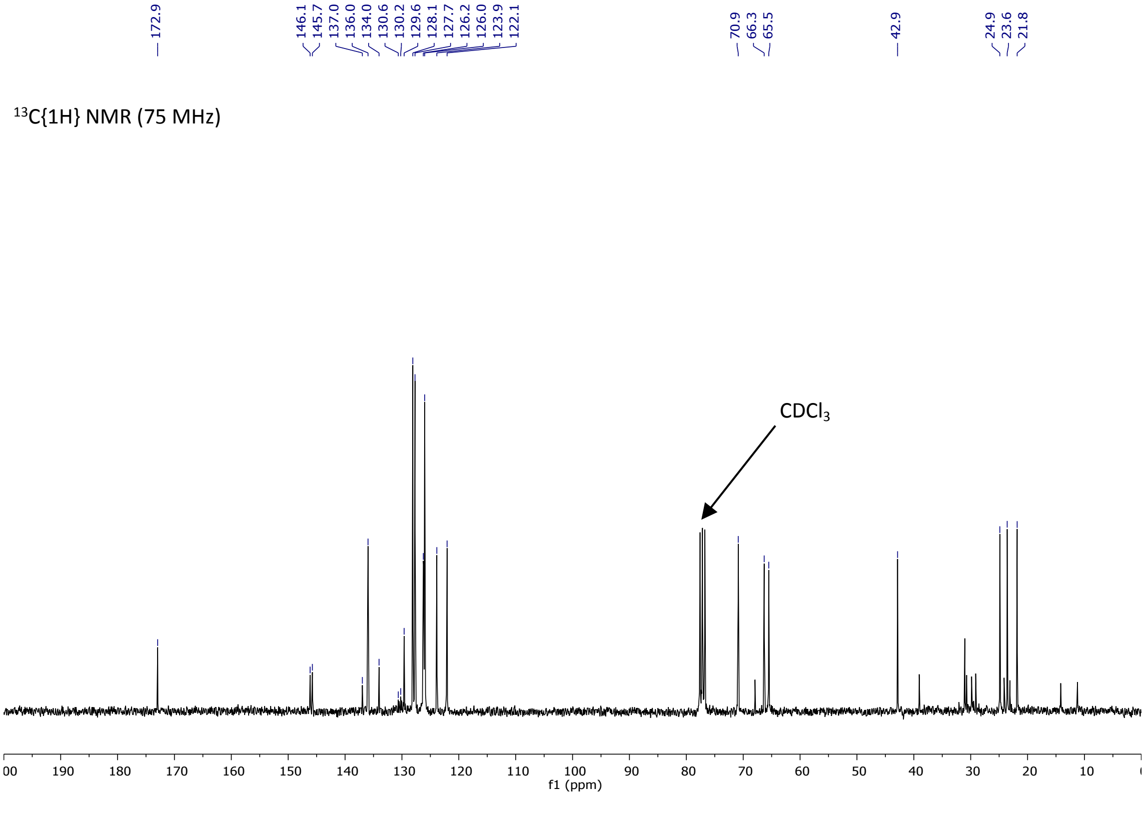

^{13}C{1H} NMR (75 MHz)
CDCl$_3$
f1 (ppm)

¹HNMR (300 MHz)

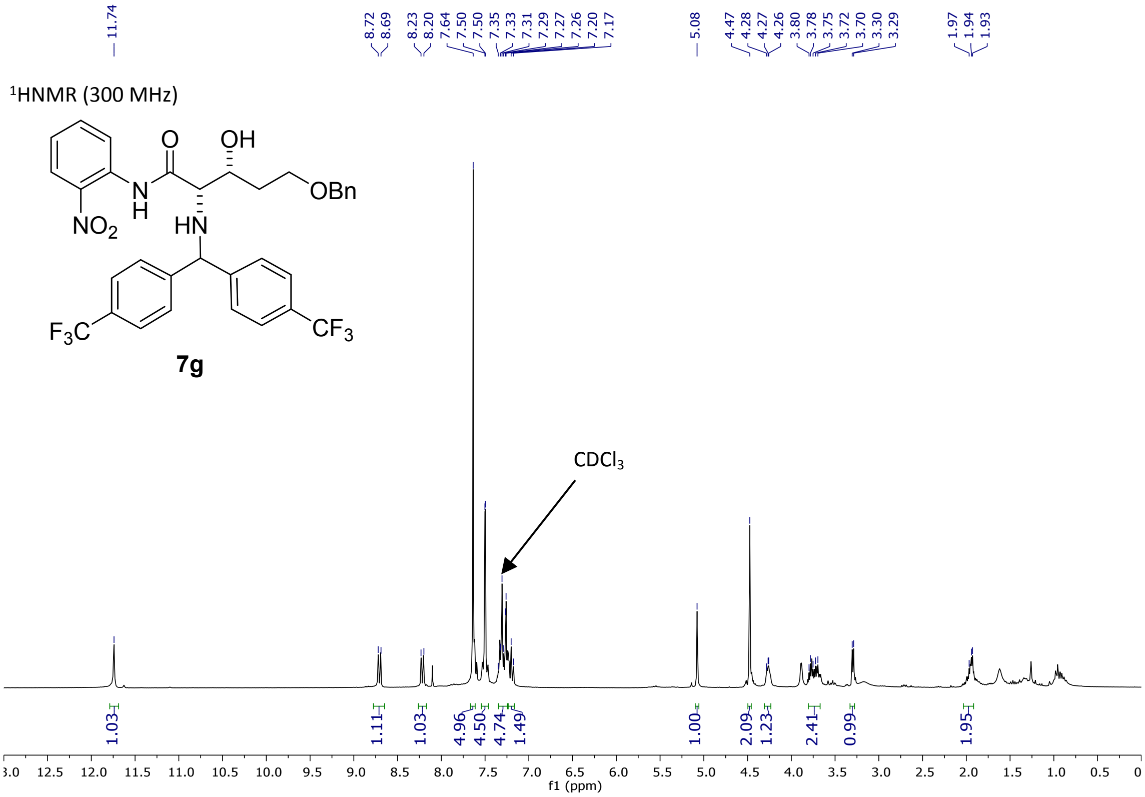

^{13}C{1H} NMR (75 MHz)

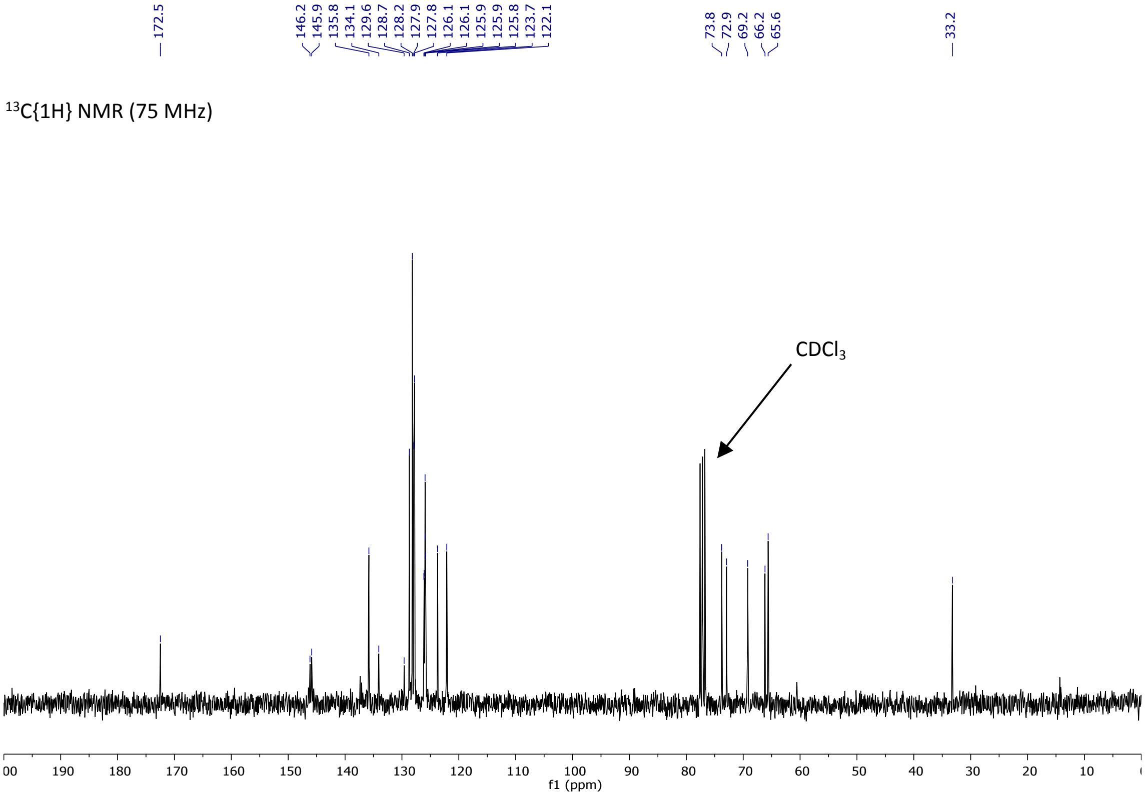

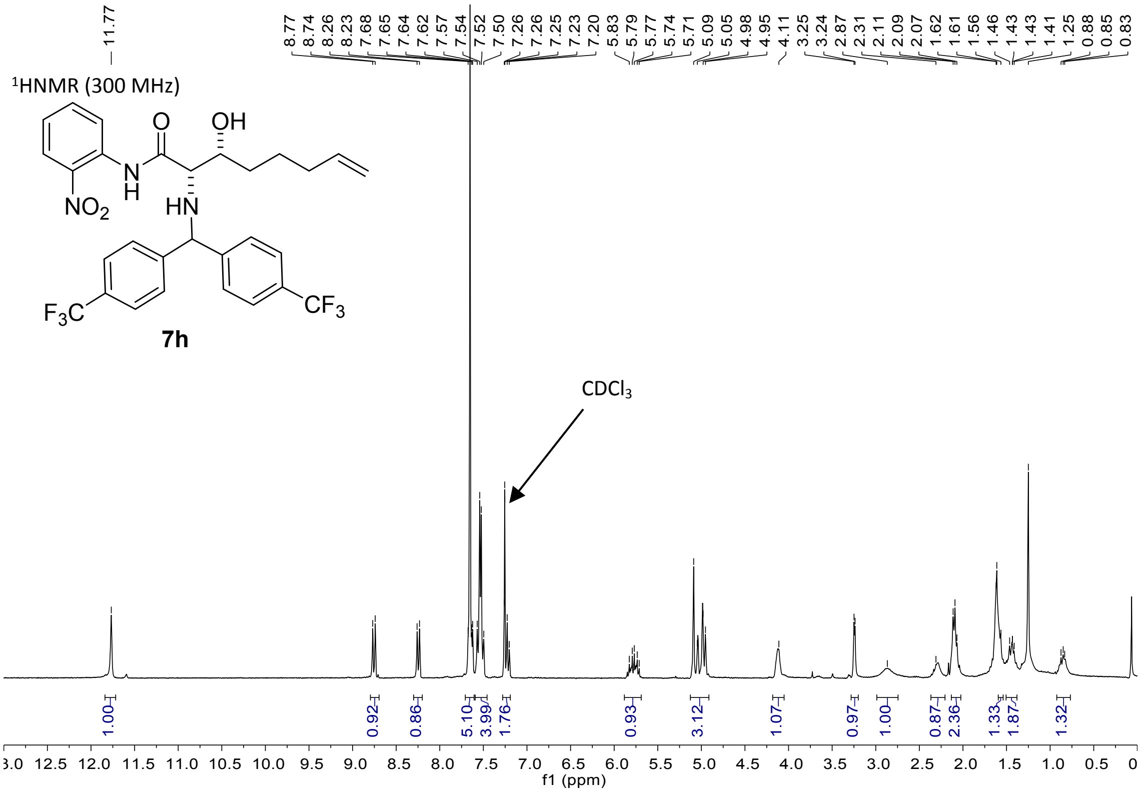

¹HNMR (300 MHz)
7h
CDCl₃
11.77
8.77 8.74 8.26 8.23 7.68 7.65 7.64 7.62 7.57 7.54 7.52 7.50 7.26 7.26 7.25 7.23 7.20 5.83 5.79 5.77 5.74 5.71 5.09 5.05 4.98 4.95 4.11 3.25 3.24 2.87 2.31 2.11 2.09 2.07 1.62 1.61 1.56 1.46 1.43 1.43 1.41 1.25 0.88 0.85 0.83
1.00 0.92 0.86 5.10 3.99 1.76 0.93 3.12 1.07 0.97 1.00 0.87 2.36 1.33 1.87 1.32
f1 (ppm)

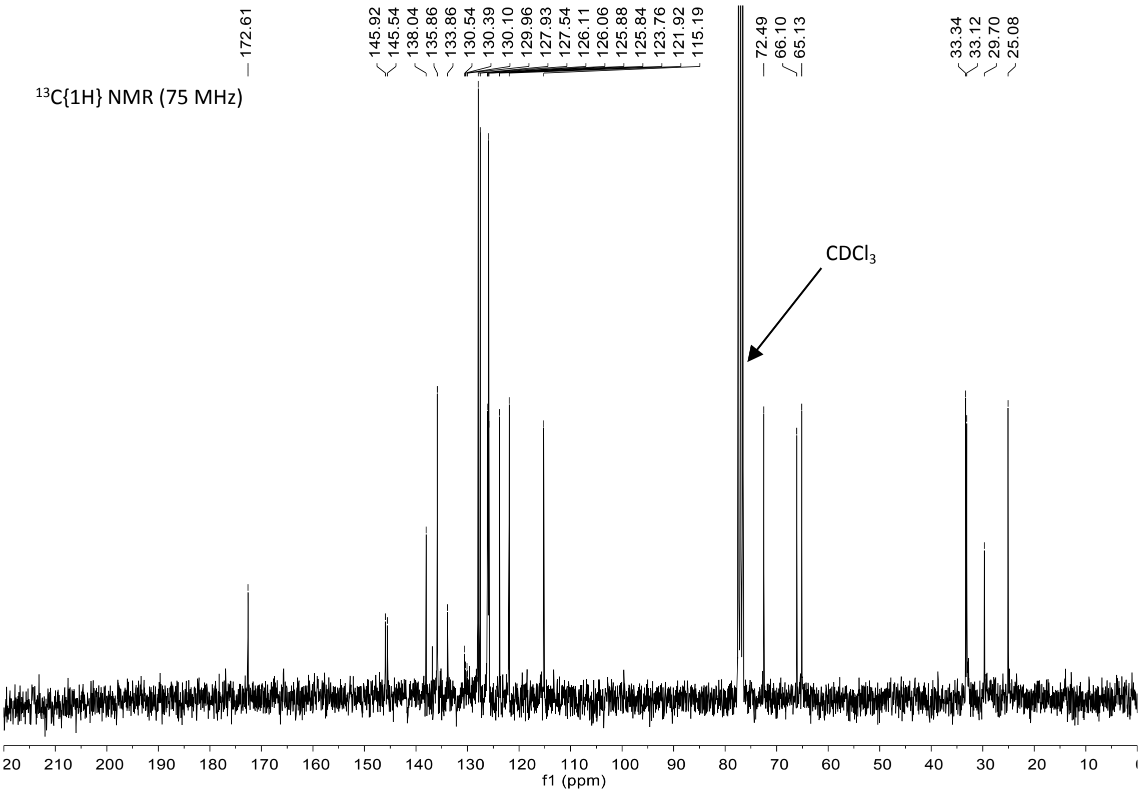

¹³C{1H} NMR (75 MHz)
CDCl₃
172.61
145.92 145.54 138.04 135.86 133.86 130.54 130.39 130.10 129.96 127.93 127.54 126.11 126.06 125.88 125.84 123.76 121.92 115.19
72.49 66.10 65.13
33.34 33.12 29.70 25.08
f1 (ppm)

^{1}HNMR (300 MHz)

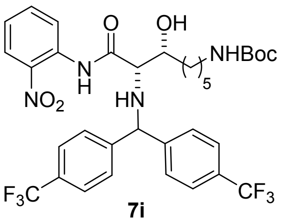

7i

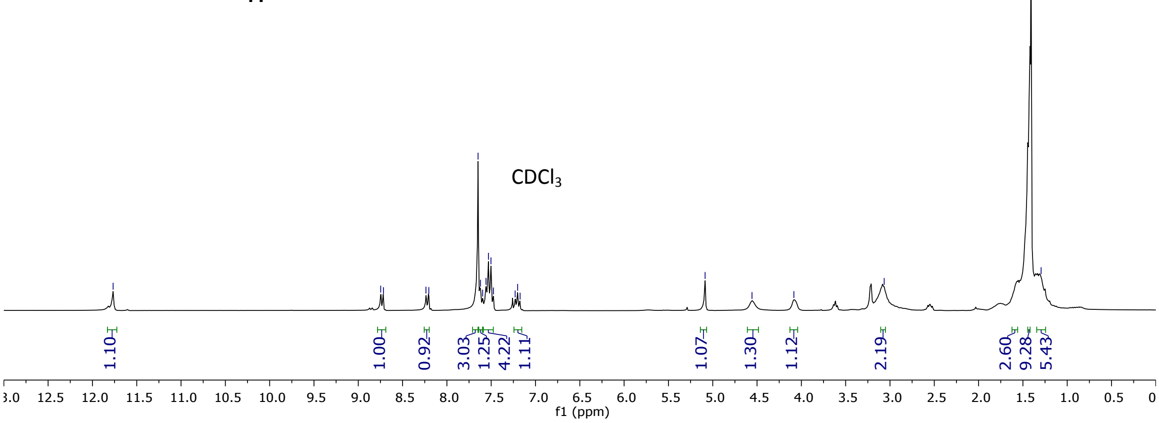

^{13}C{1H} NMR (75 MHz)

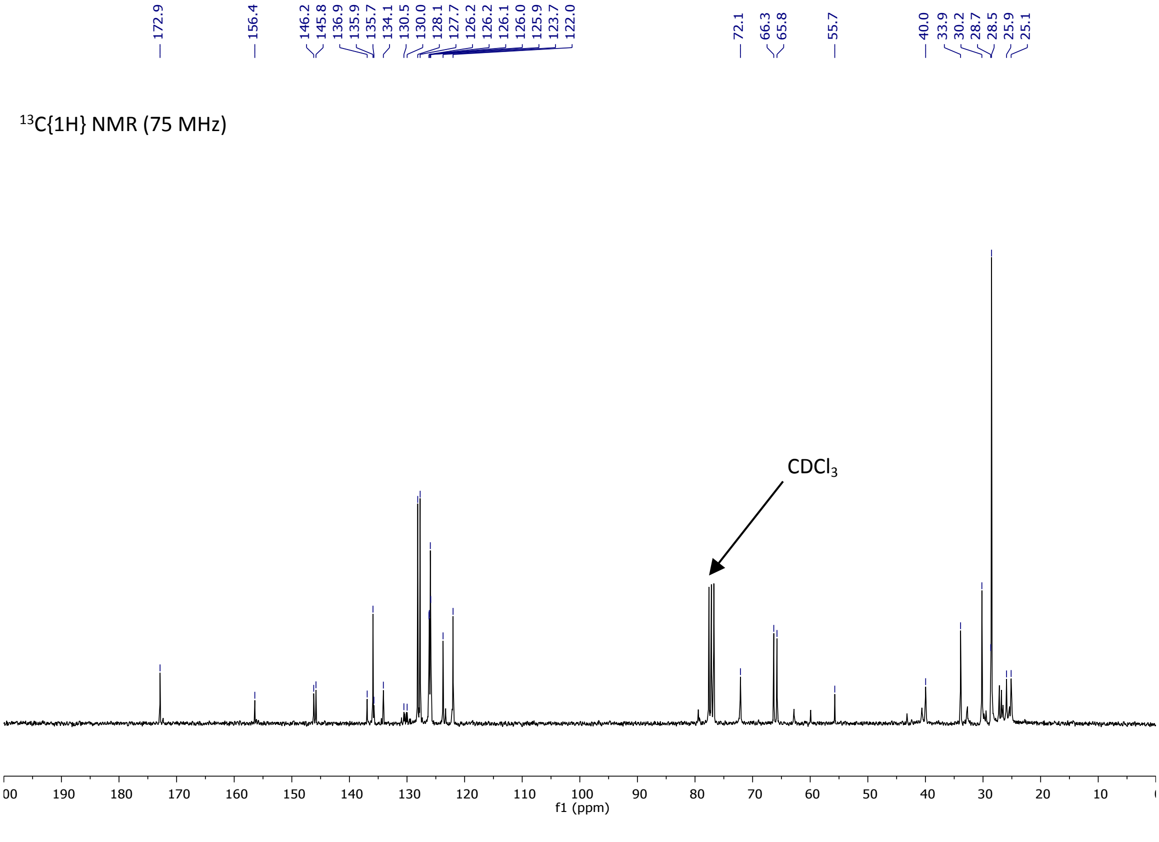

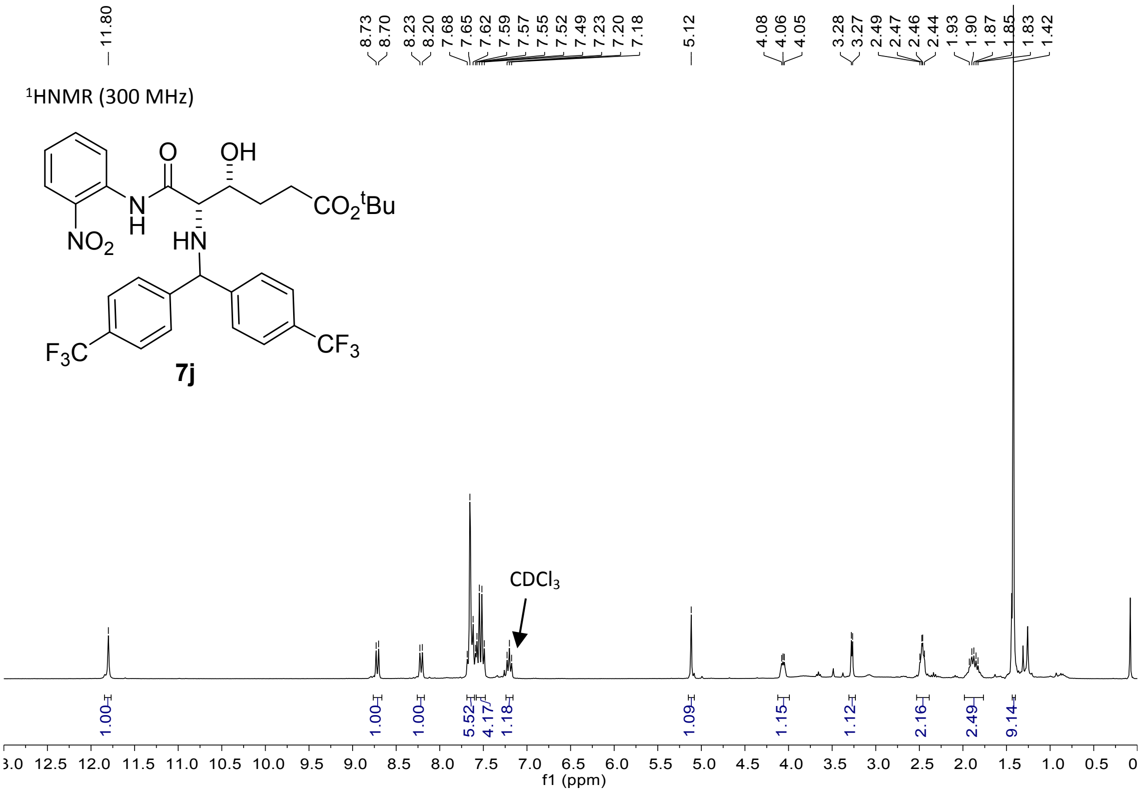
11.80
8.73
8.70
8.23
8.20
7.68
7.65
7.62
7.59
7.57
7.55
7.52
7.49
7.23
7.20
7.18
5.12
4.08
4.06
4.05
3.28
3.27
2.49
2.47
2.46
2.44
1.93
1.90
1.87
1.85
1.83
1.42
¹HNMR (300 MHz)
O
OH
N
H
NO2
HN
CO2tBu
F3C
CF3
7j
CDCl3
1.00
1.00
1.00
5.52
4.17
1.18
1.09
1.15
1.12
2.16
2.49
9.14
f1 (ppm)

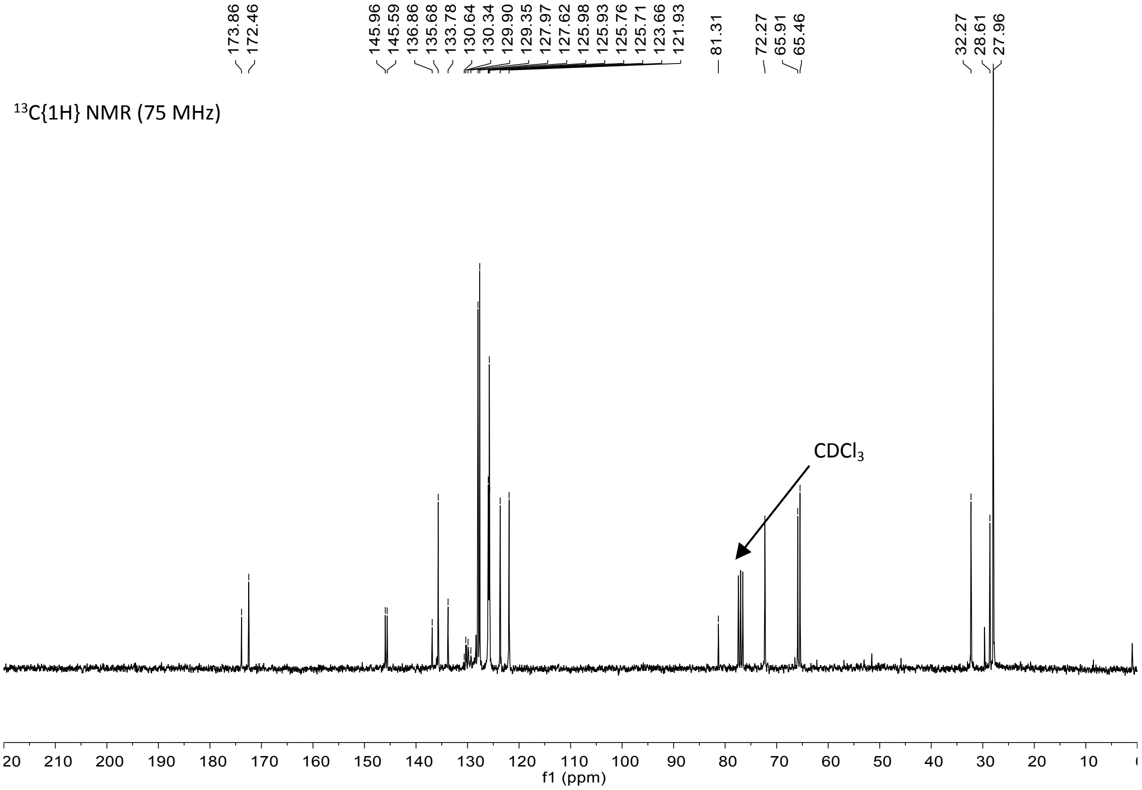
173.86
172.46
145.96
145.59
136.86
135.68
133.78
130.64
130.34
129.90
129.35
127.97
127.62
125.98
125.93
125.76
125.71
123.66
121.93
81.31
72.27
65.91
65.46
32.27
28.61
27.96
¹³C{1H} NMR (75 MHz)
CDCl3
f1 (ppm)

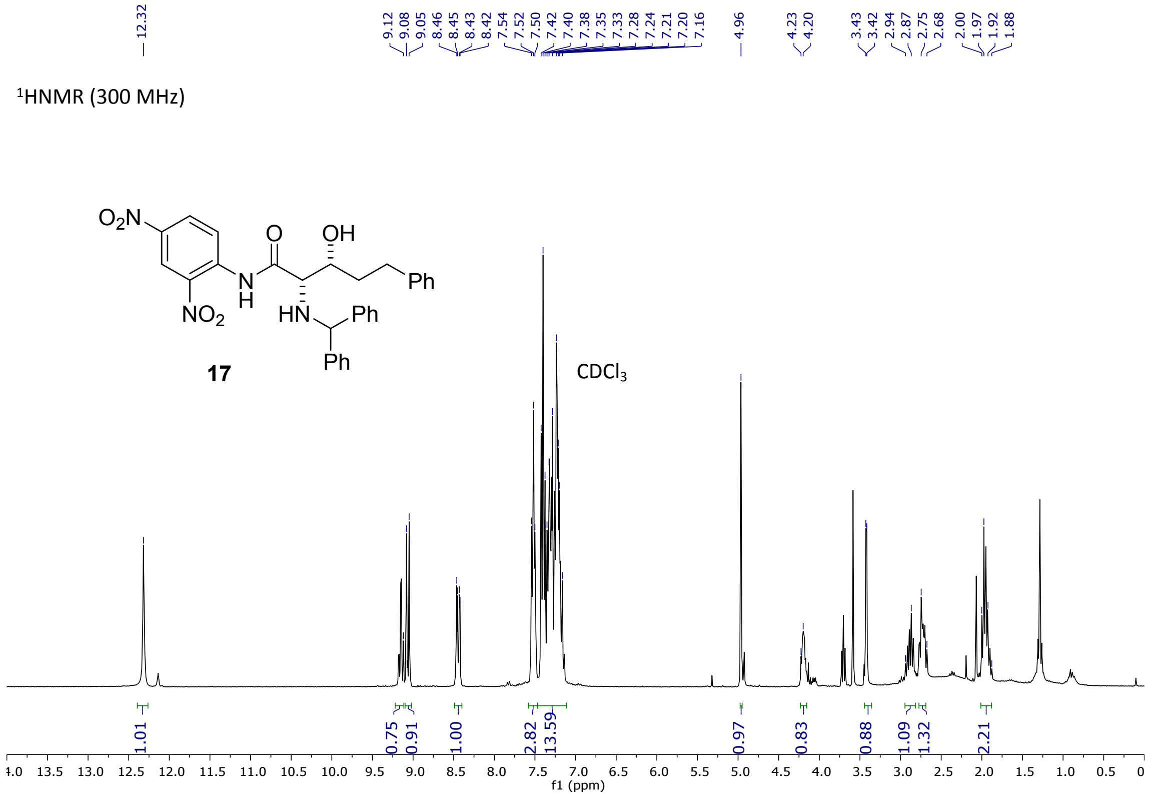
12.32
9.12 9.08 9.05 8.46 8.45 8.43 8.42 7.54 7.52 7.50 7.42 7.40 7.38 7.35 7.33 7.28 7.24 7.21 7.20 7.16
4.96
4.23 4.20
3.43 3.42 2.94 2.87 2.75 2.68 2.00 1.97 1.92 1.88
1HNMR (300 MHz)
O2N
NO2
N
H
O
OH
Ph
HN
Ph
Ph
17
CDCl3
1.01 0.75 0.91 1.00 2.82 13.59 0.97 0.83 0.88 1.09 1.32 2.21
f1 (ppm)

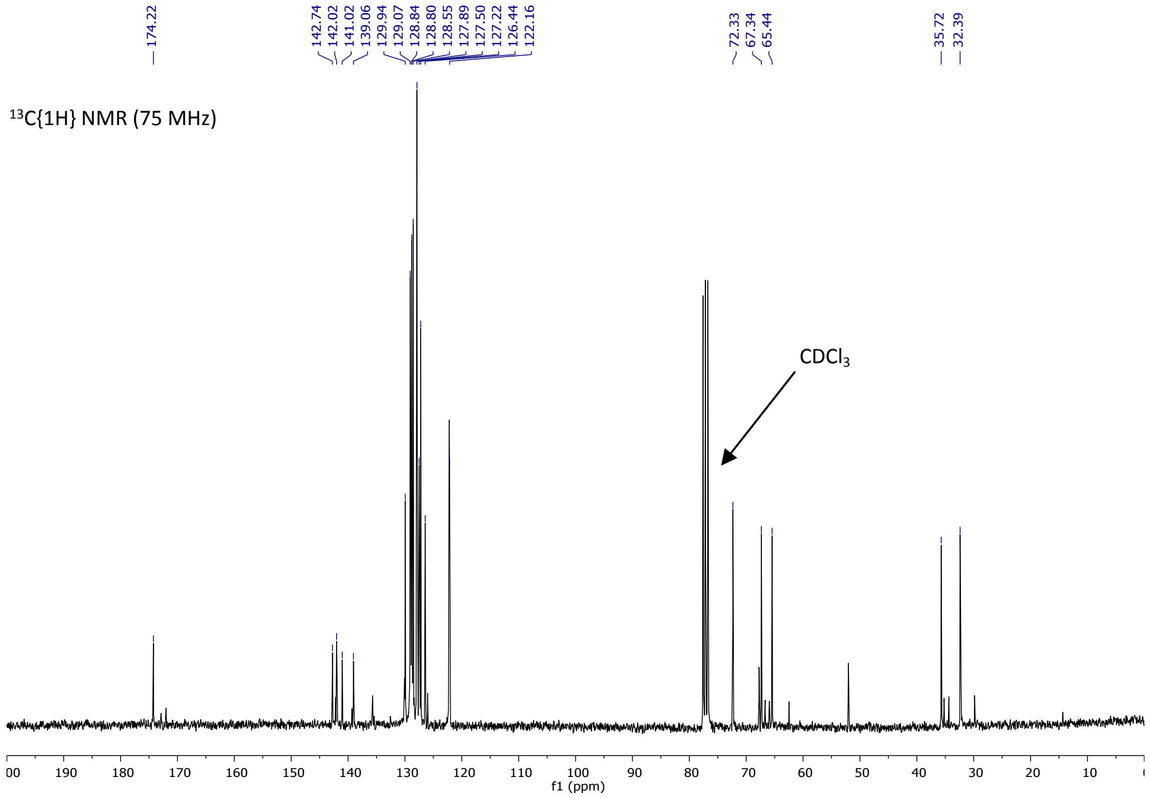
174.22
142.74 142.02 141.02 139.06 129.94 129.07 128.84 128.80 128.55 127.89 127.50 127.22 126.44 122.16
72.33 67.34 65.44
35.72 32.39
13C{1H} NMR (75 MHz)
CDCl3
f1 (ppm)

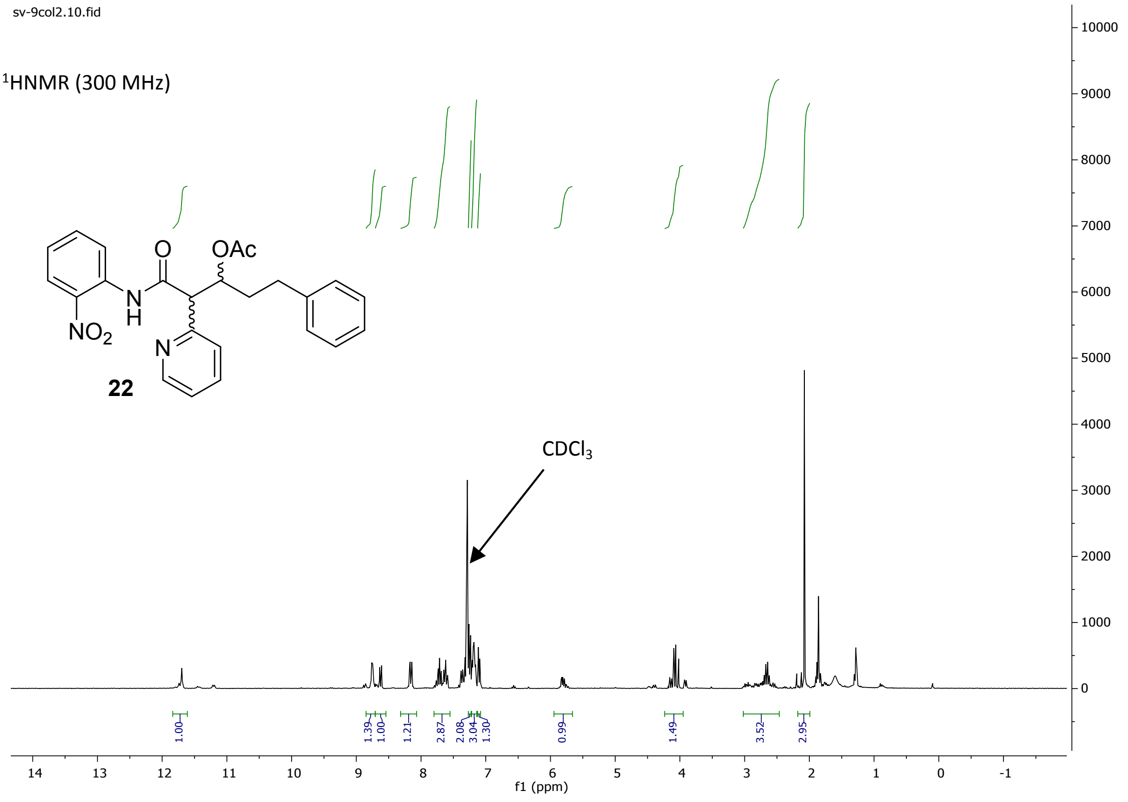
sv-9col2.10.fid
¹HNMR (300 MHz)
OAc
O
N
H
NO2
N
22
CDCl3
1.00
1.39
1.00
1.21
2.87
2.08
3.04
1.30
0.99
1.49
3.52
2.95
f1 (ppm)

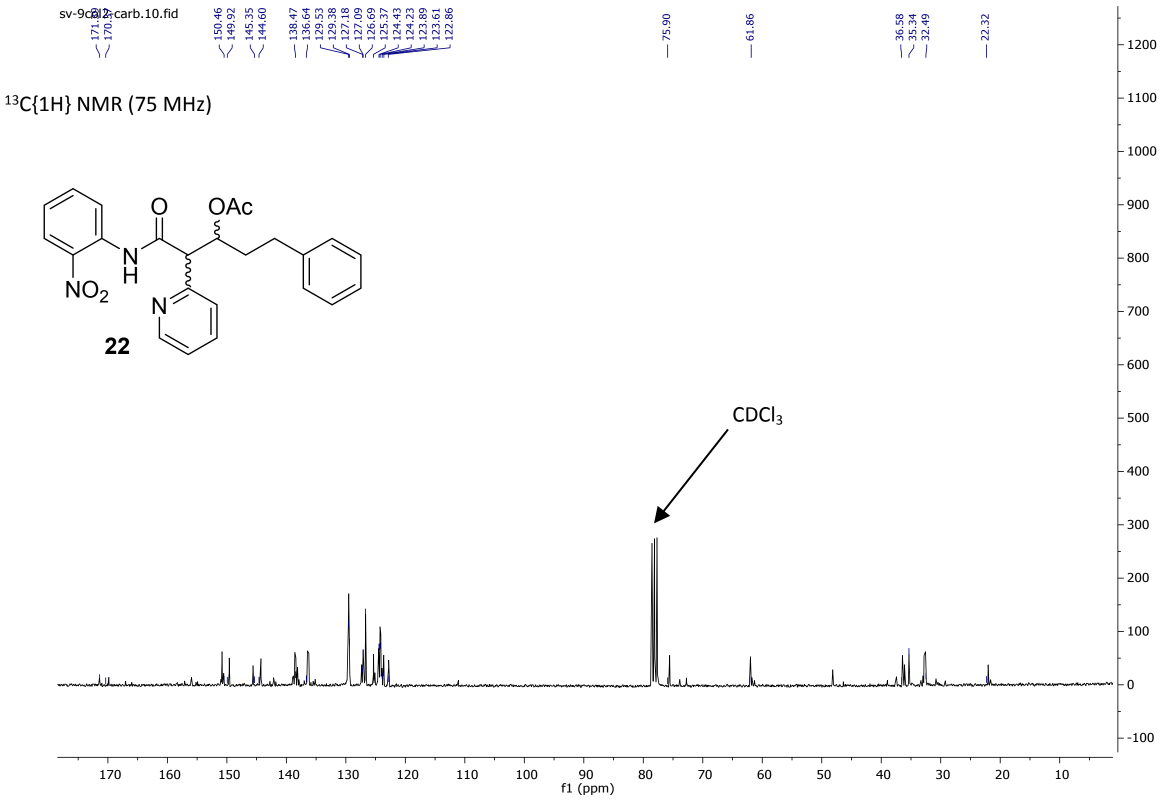
sv-9col2-carb.10.fid
150.46
149.92
145.35
144.60
138.47
136.64
129.53
129.38
127.18
127.09
126.69
125.37
124.43
124.23
123.89
123.61
122.86
75.90
61.86
36.58
35.34
32.49
22.32
¹³C{1H} NMR (75 MHz)
OAc
O
N
H
NO2
N
22
CDCl3
f1 (ppm)

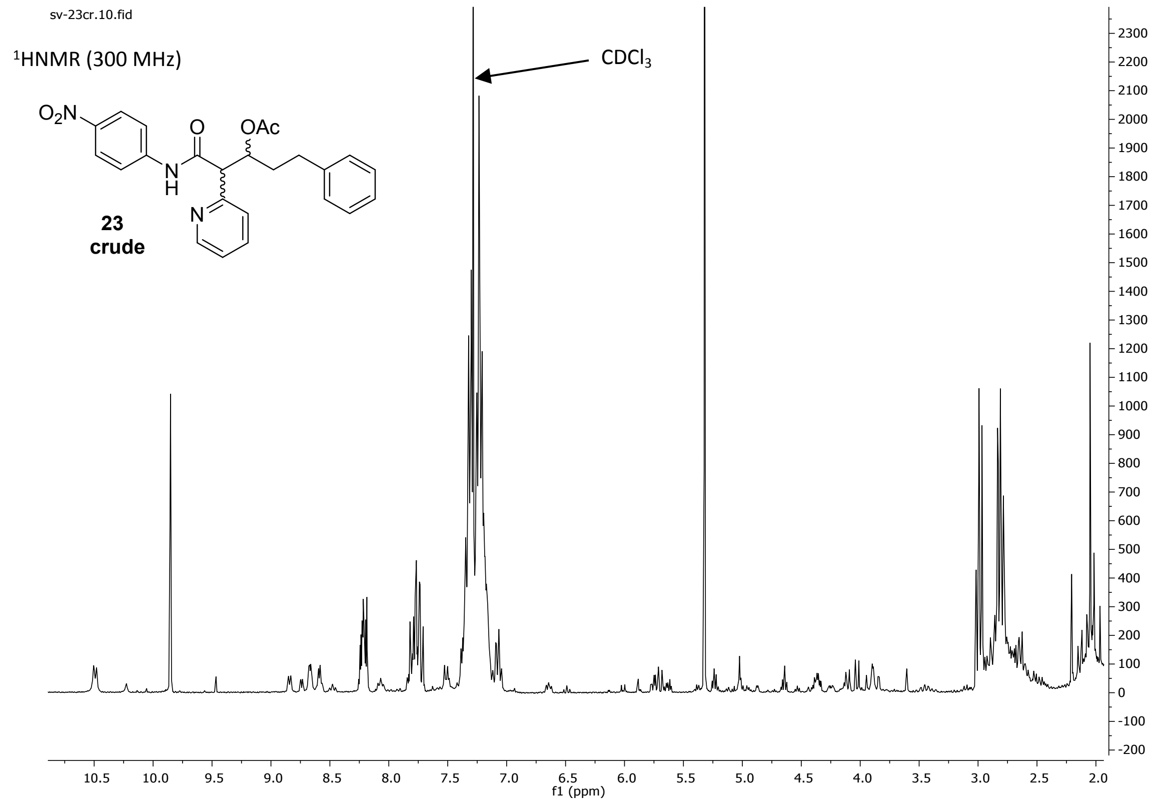
sv-23cr.10.fid
1HNMR (300 MHz)
CDCl3
O2N
O
OAc
N
H
N
23
crude
f1 (ppm)

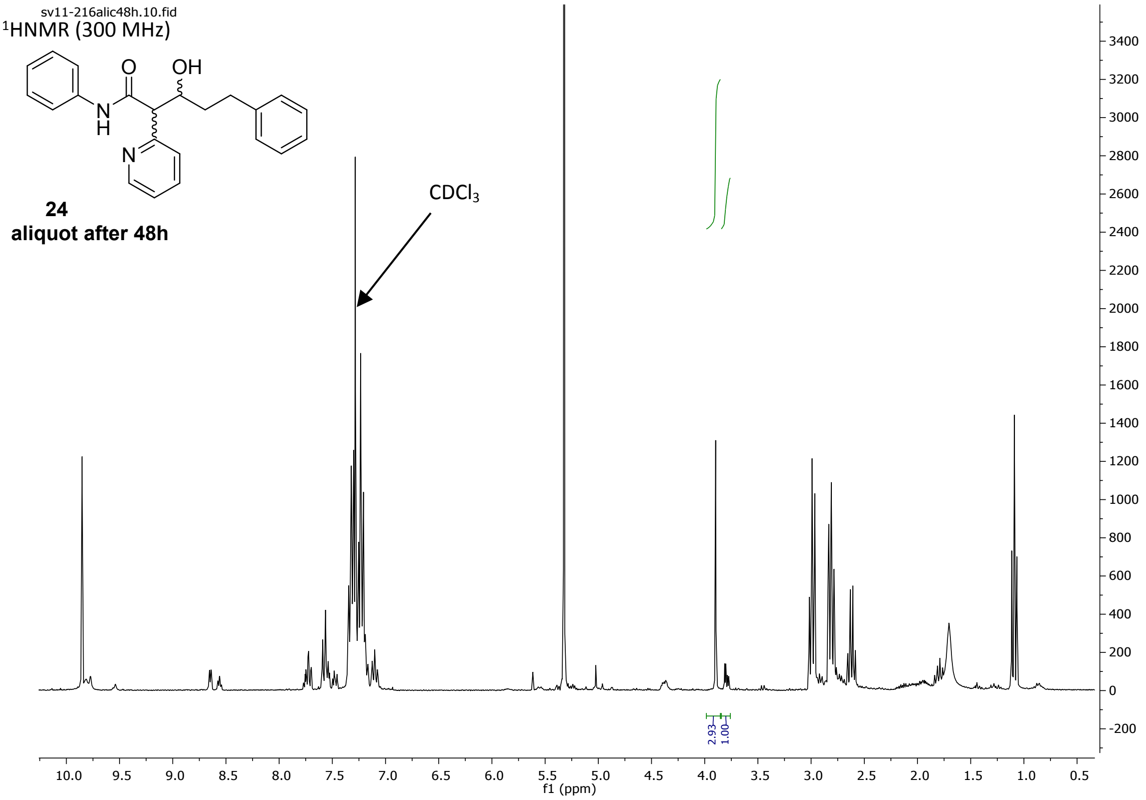
sv11-216alic48h.10.fid
^{1}HNMR (300 MHz)
OH
O
N
H
N
24
aliquot after 48h
CDCl$_3$
2.93
1.00
f1 (ppm)

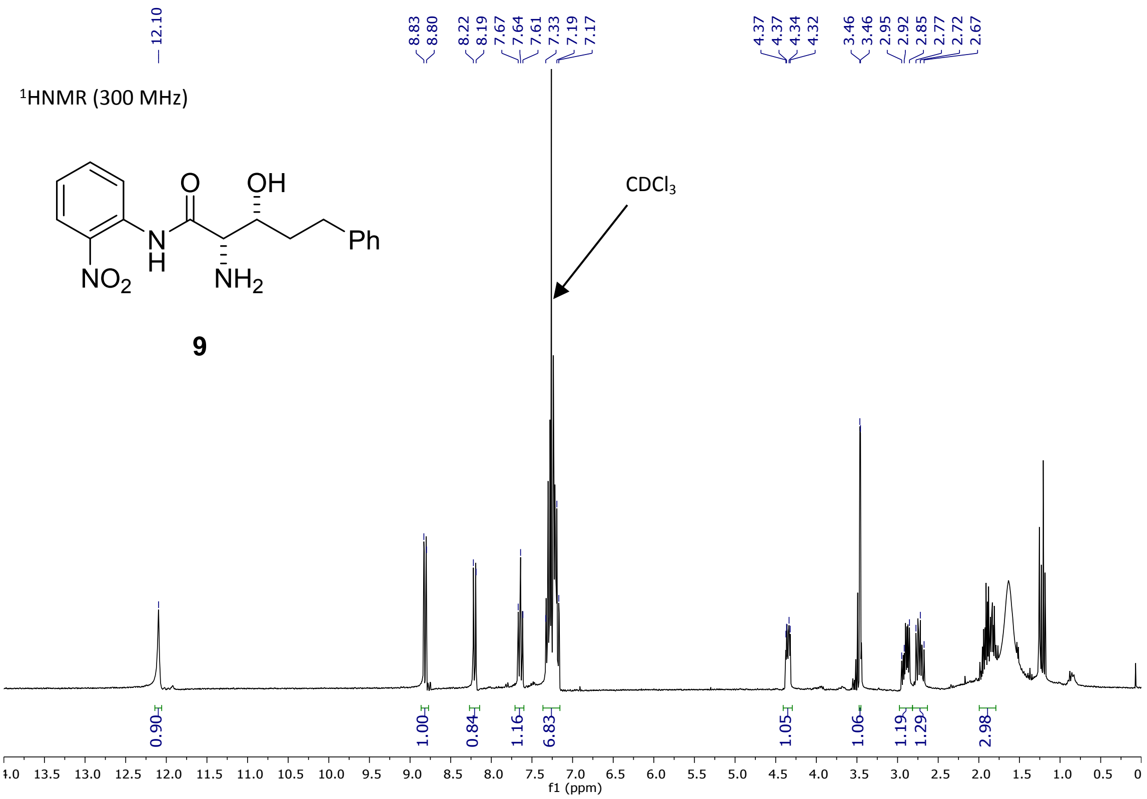
1HNMR (300 MHz)
12.10
8.83
8.80
8.22
8.19
7.67
7.64
7.61
7.33
7.19
7.17
4.37
4.37
4.34
4.32
3.46
3.46
2.95
2.92
2.85
2.77
2.72
2.67
CDCl3
O
OH
N
H
NO2
NH2
Ph
9
0.90
1.00
0.84
1.16
6.83
1.05
1.06
1.19
1.29
2.98
f1 (ppm)

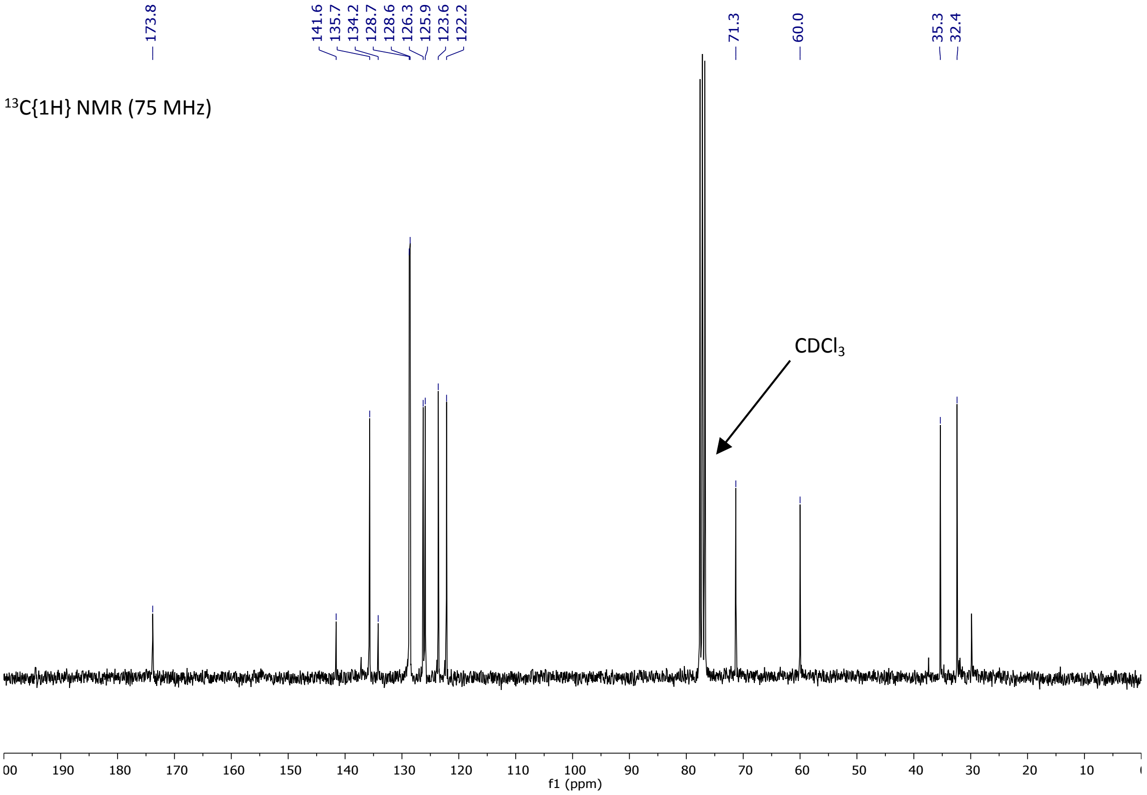
13C{1H} NMR (75 MHz)
173.8
141.6
135.7
134.2
128.7
128.6
126.3
125.9
123.6
122.2
71.3
60.0
35.3
32.4
CDCl3
f1 (ppm)

^{1}HNMR (300 MHz)

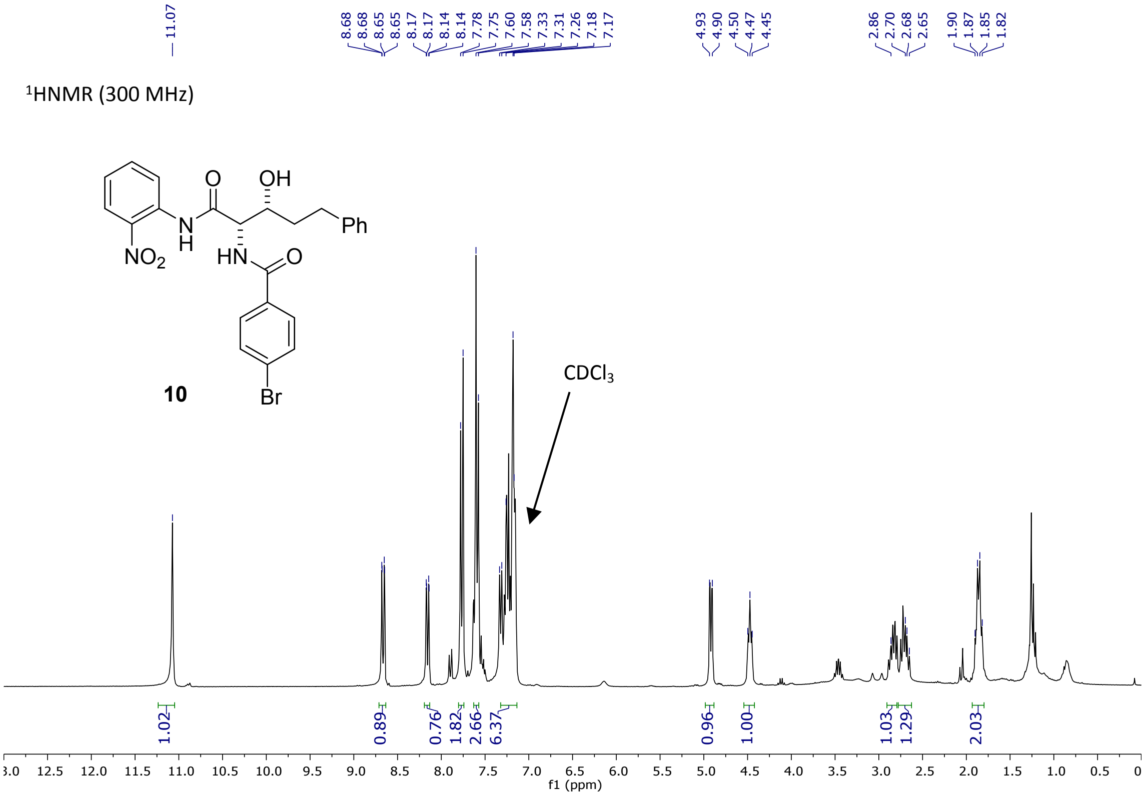

^{13}C{1H} NMR (75 MHz)

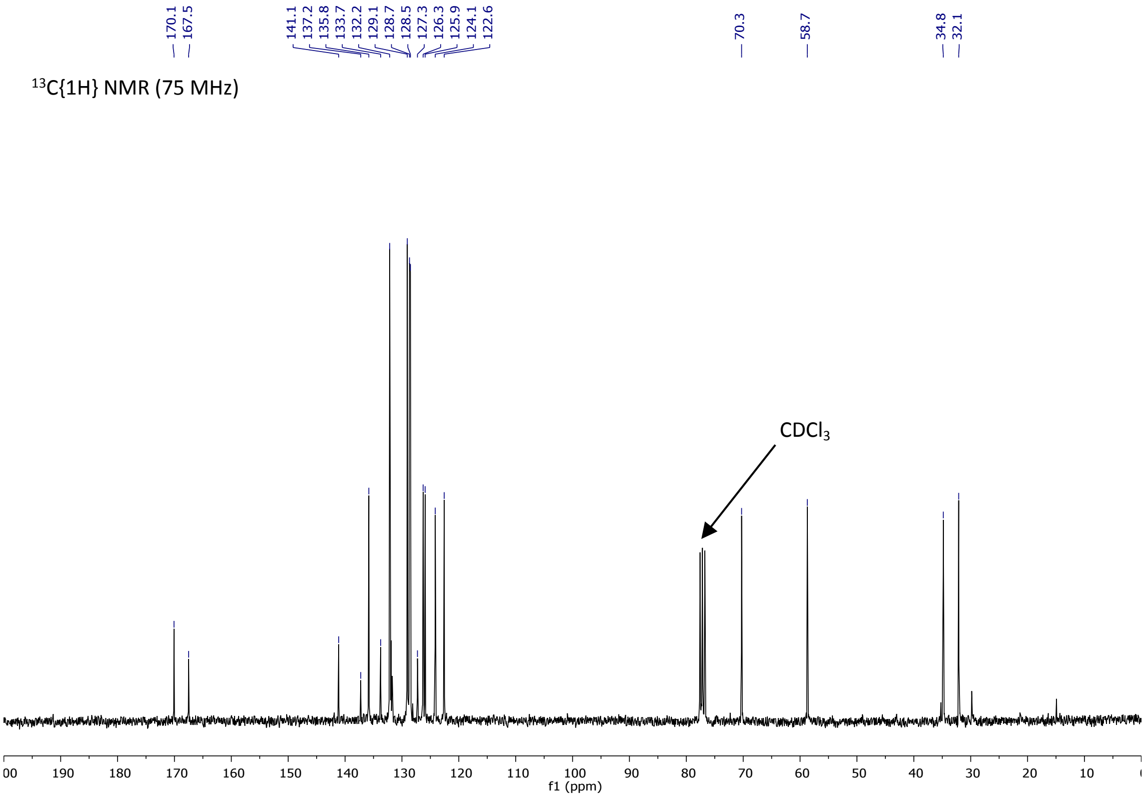

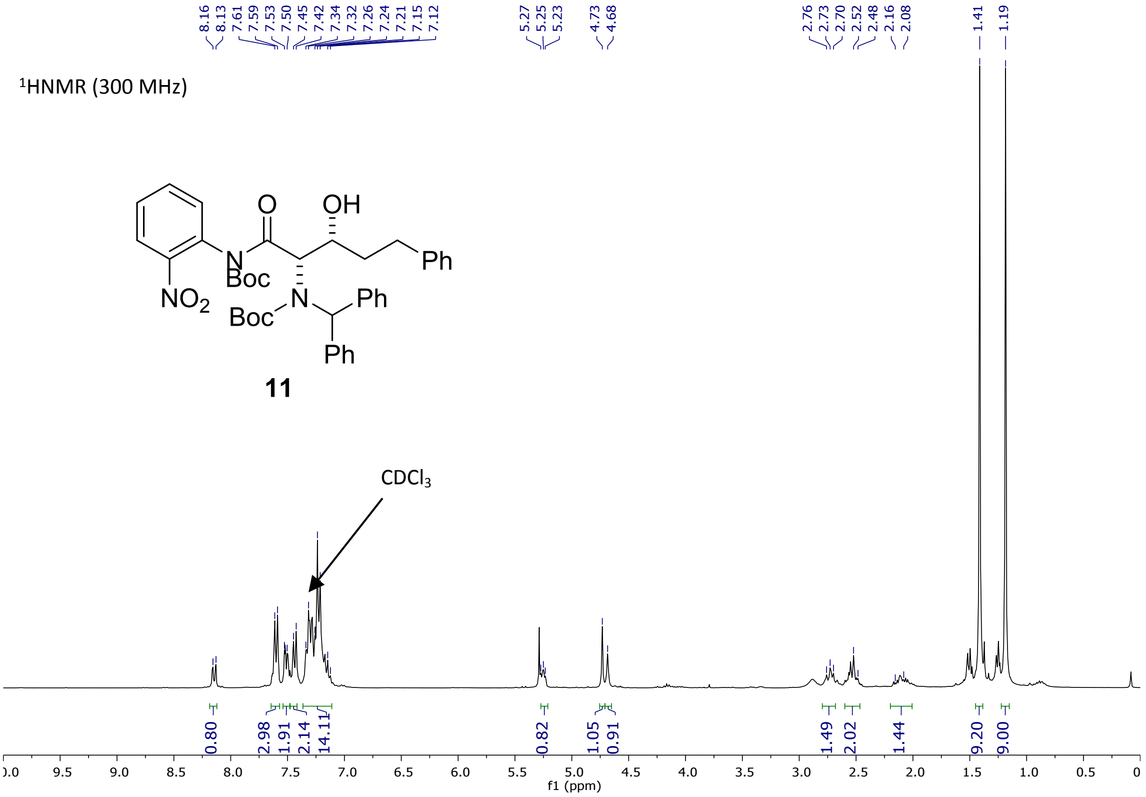

8.16 8.13 7.61 7.59 7.53 7.50 7.45 7.42 7.34 7.32 7.26 7.24 7.21 7.15 7.12
5.27 5.25 5.23
4.73 4.68
2.76 2.73 2.70 2.52 2.48 2.16 2.08
1.41
1.19
¹HNMR (300 MHz)
O
OH
N
Boc
Ph
NO_2
Boc
N
Ph
Ph
11
$CDCl_3$
0.80 2.98 1.91 2.14 14.11 0.82 1.05 0.91 1.49 2.02 1.44 9.20 9.00
f1 (ppm)

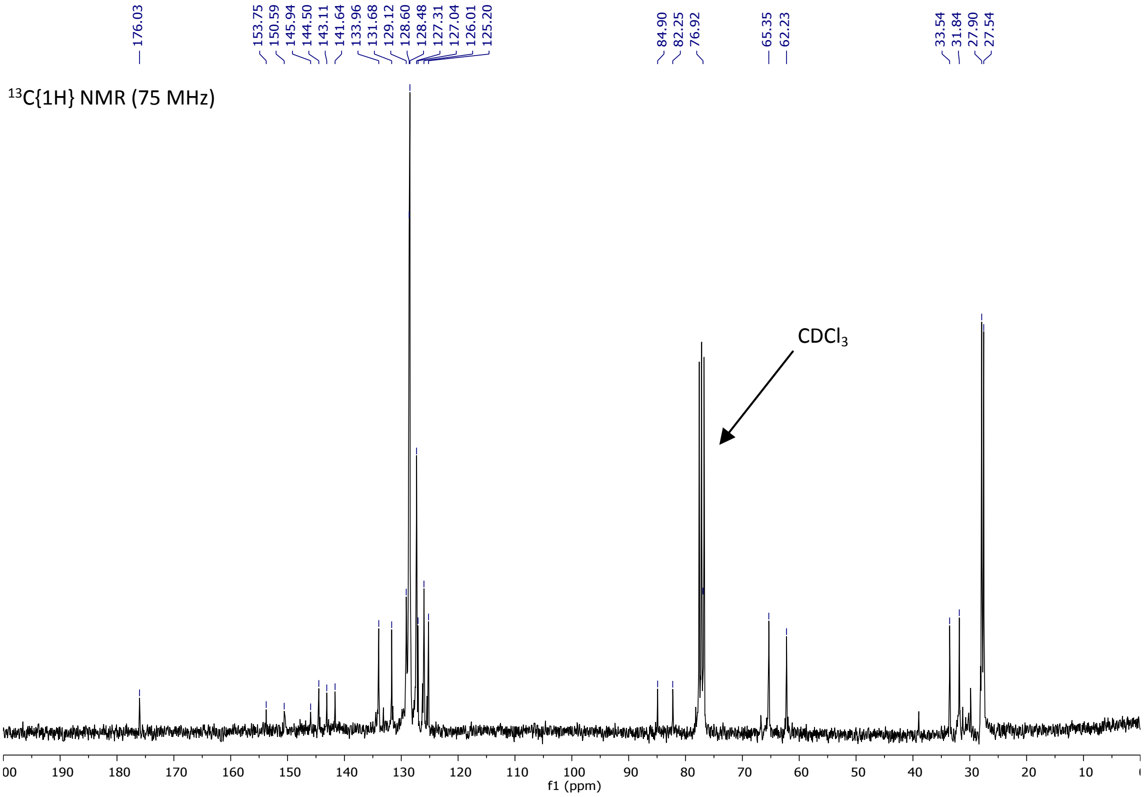

176.03
153.75 150.59 145.94 144.50 143.11 141.64 133.96 131.68 129.12 128.60 128.48 127.31 127.04 126.01 125.20
84.90 82.25 76.92
65.35 62.23
33.54 31.84 27.90 27.54
¹³C{1H} NMR (75 MHz)
$CDCl_3$
f1 (ppm)

^{1}HNMR (300 MHz)

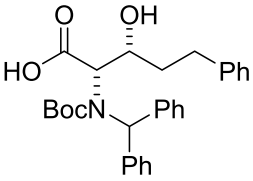

12

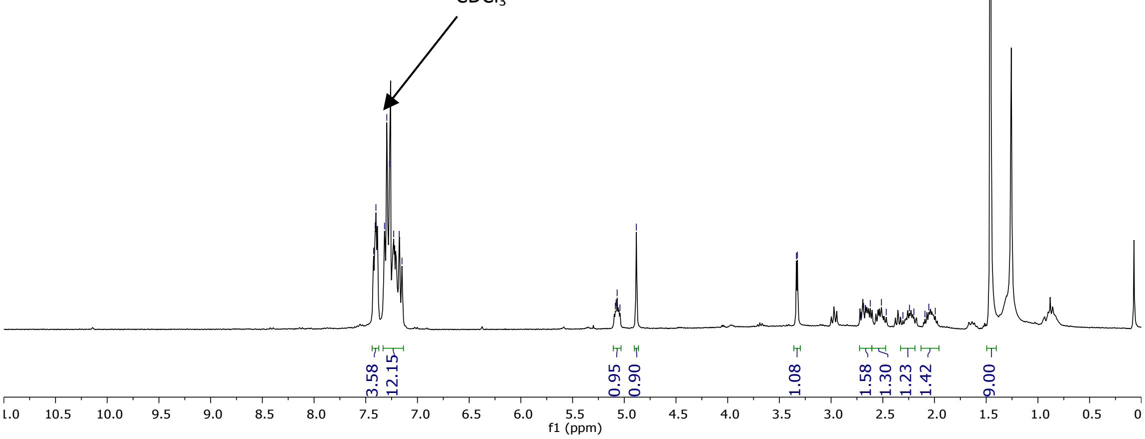

^{13}C{1H} NMR (75 MHz)

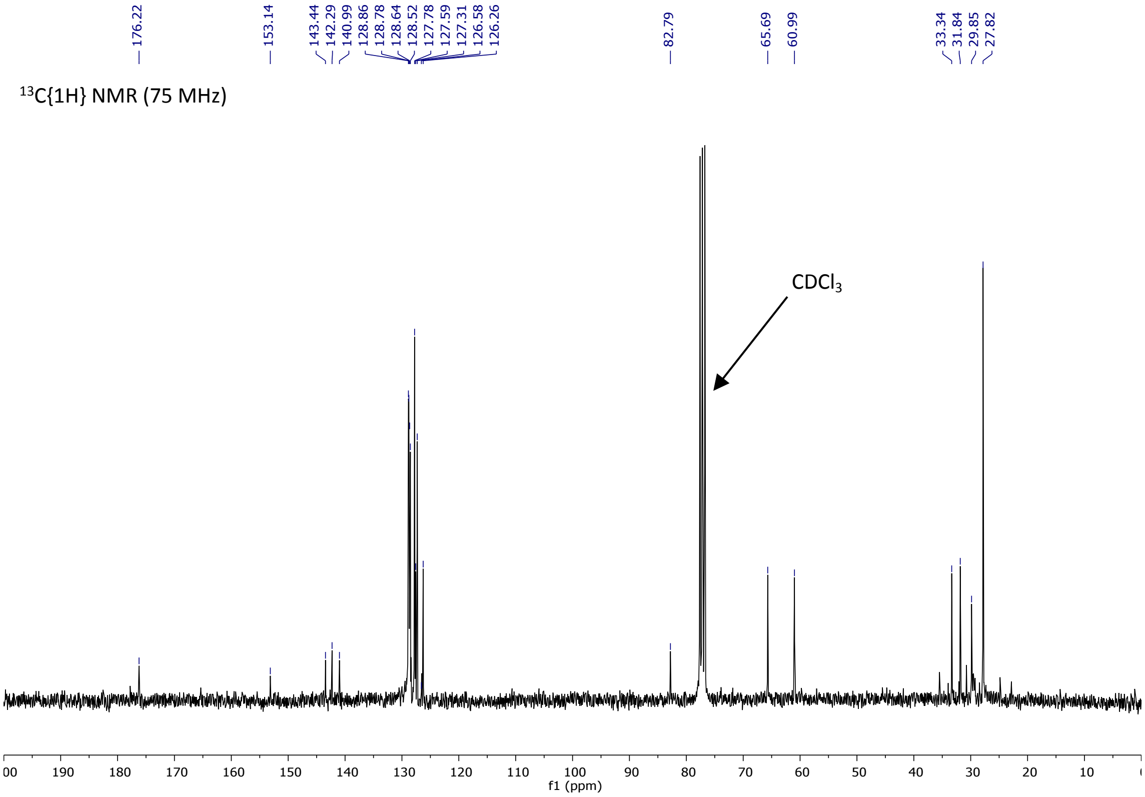

HPLC Chromatograms

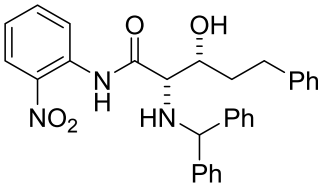

5a

Eluent: Hx/EtOH, 90/10

Flow: 1 mL/min

Column: IA

λ: 254 nm

rac-**5a**

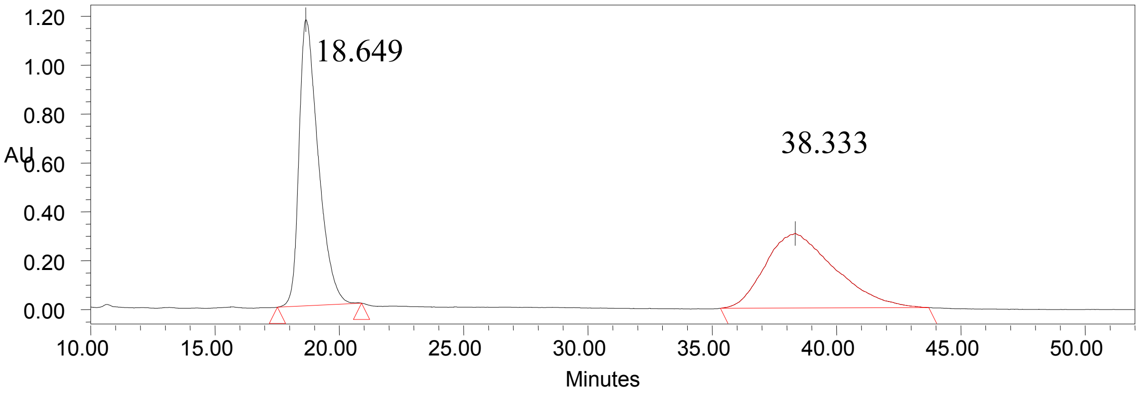

	Retention Time	% Area
1	18.649	50.47
2	38.333	49.53

5a

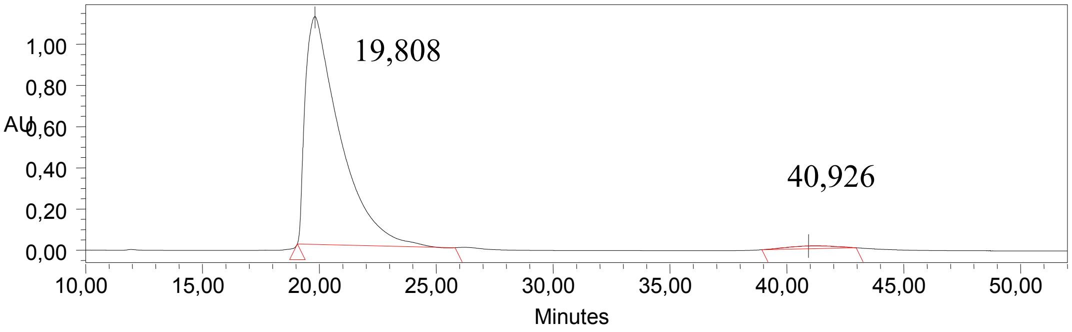

	Retention Time	% Area
1	19,808	97,12
2	41,228	2,88

94% *ee*

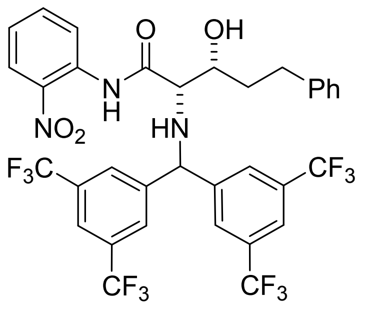

6a

Eluent: Hx/*i*PrOH, 98/2

Flow: 0.5 mL/min

Column: IAIA

λ: 254 nm

rac-**6a**

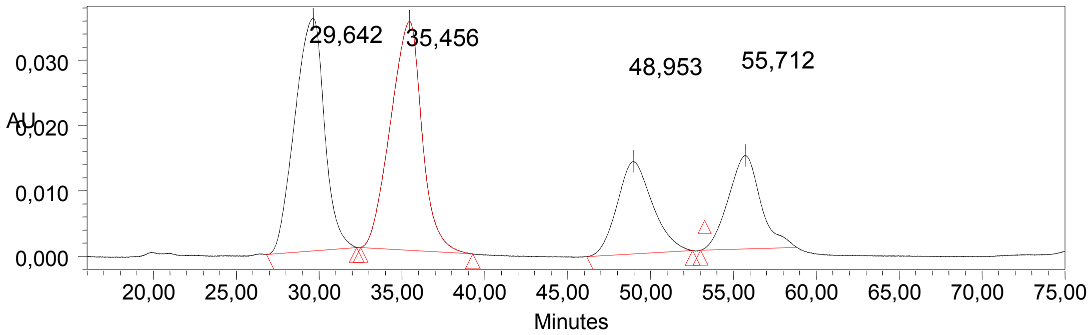

	Retention Time	% Area
1	29,642	33,89
2	35,456	35,33
3	48,953	15,97
4	55,712	14,81

6a

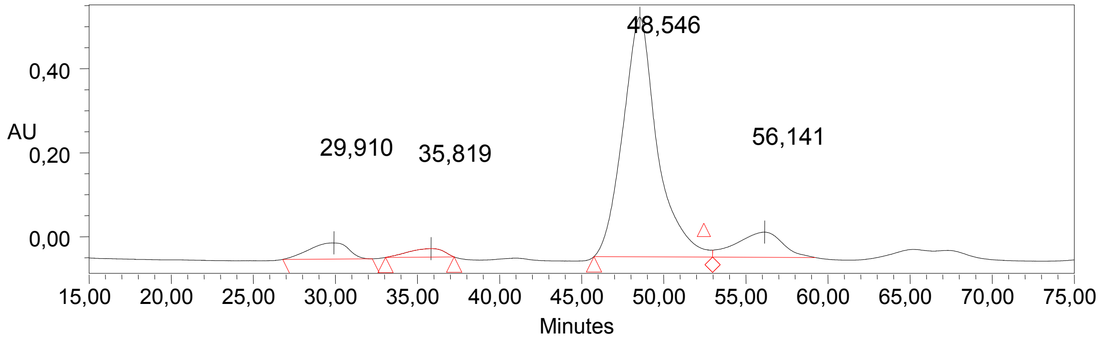

	Retention Time	% Area
1	48,546	87,65
2	56,141	12,35

76% *ee*

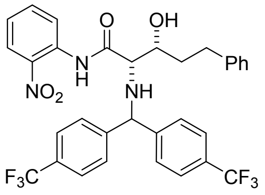

7a

Eluent: Hx/EtOH, 98/2

Flow: 0.5 mL/min

Column: IF

λ: 254 nm

Rac-**7a**

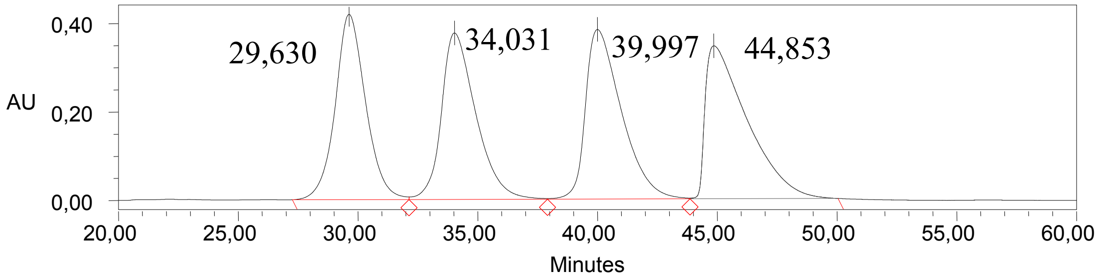

	Retention Time	% Area
1	29,630	23,67
2	34,031	24,00
3	39,997	25,50
4	44,853	26,82

7a

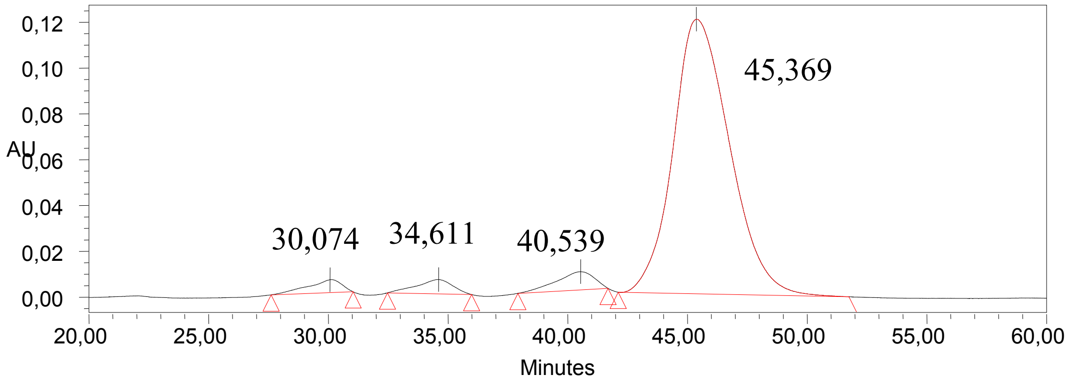

	Retention Time	% Area
1	40,539	4,29
2	45,369	95,71

92% *ee*

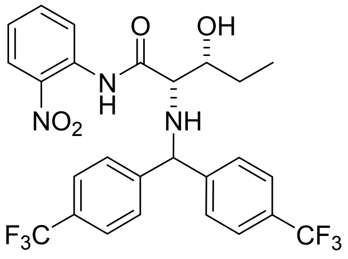

7b

Eluent: Hx/*i*PrOH, 98/2

Flow: 1 mL/min

Column: Phenomenex-Lux 3μm Amylose-1

λ: 227 nm

Rac-**7b**

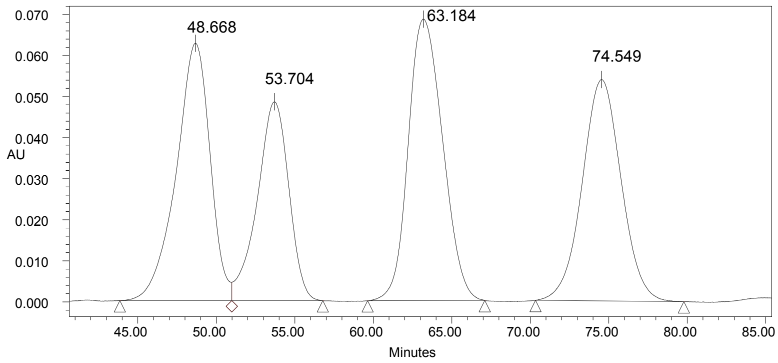

	Retention Time	% Area
1	48.668	22.20
2	53.704	20.05
3	63.184	29.03
4	74.549	28.71

7b

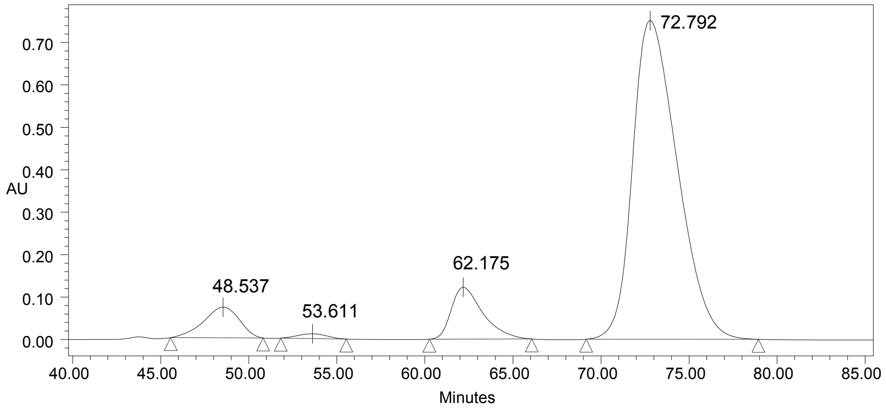

	Retention Time	% Area
1	48.538	4.60
2	53.611	0.64
3	62.176	7.26
4	72.792	87.50

	Retention Time	% Area
1	62.176	7.66
2	72.792	92.23

85% *ee*

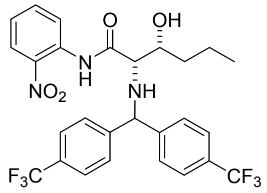

7c

Eluent: Hx/*i*PrOH, 95/5

Flow: 0.5 mL/min

Column: Phenomenex-Lux 3µm Amylose-1

λ: 254 nm

Rac-**7c**

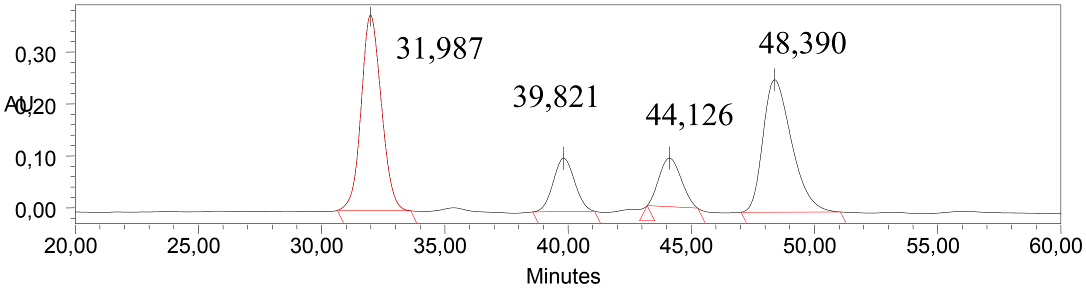

	Retention Time	% Area
1	31,987	39,71
2	39,821	11,48
3	44,126	10,76
4	48,390	38,06

7c

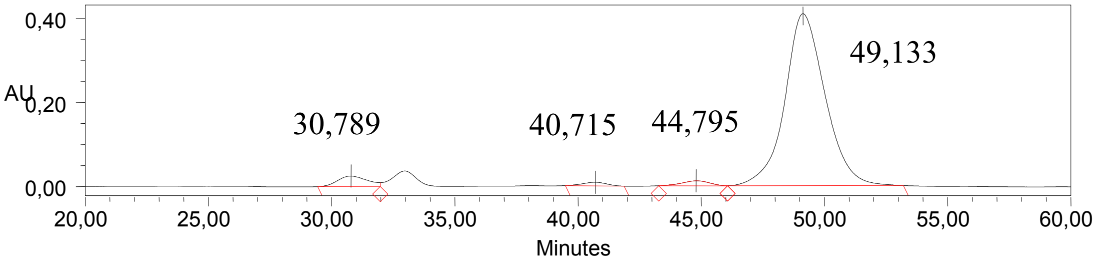

	Retention Time	% Area
1	30,789	4,18
2	40,715	1,16
3	44,795	1,89
4	49,133	92,78

	Retention Time	% Area
1	30,789	4,31
2	49,133	95,69

92% *ee*

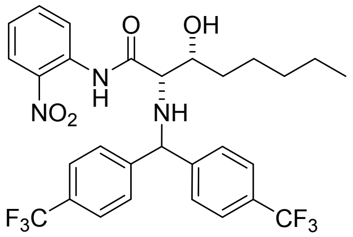

7d

Eluent: Hx/*i*PrOH, 95/5

Flow: 0.5 mL/min

Column: Phenomenex-Lux 3μm Amylose-1

λ: 254 nm

Rac-**7d**

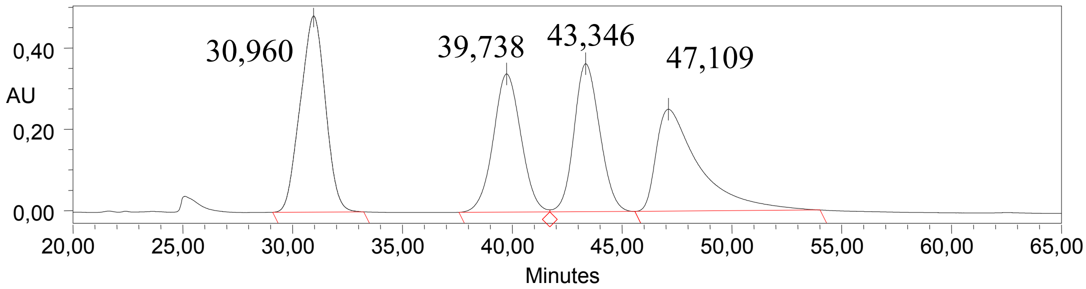

	Retention Time	% Area
1	30,961	28,36
2	39,738	22,59
3	43,346	22,38
4	47,109	26,67

7d

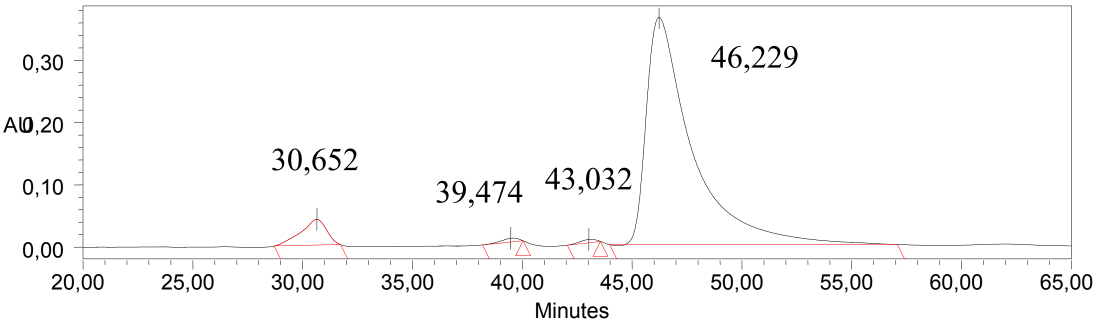

	Retention Time	% Area
1	30,652	5,13
2	39,474	0,56
3	43,032	0,47
4	46,229	93,84

	Retention Time	% Area
1	30,652	5,18
2	46,229	94,82

90% *ee*

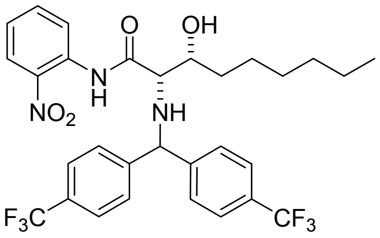

7e

Eluent: Hx/*i*PrOH, 95/5

Flow: 0.5 mL/min

Column: Phenomenex-Lux 3 µm Cellulose-1

λ: 254 nm

Rac-**7e**

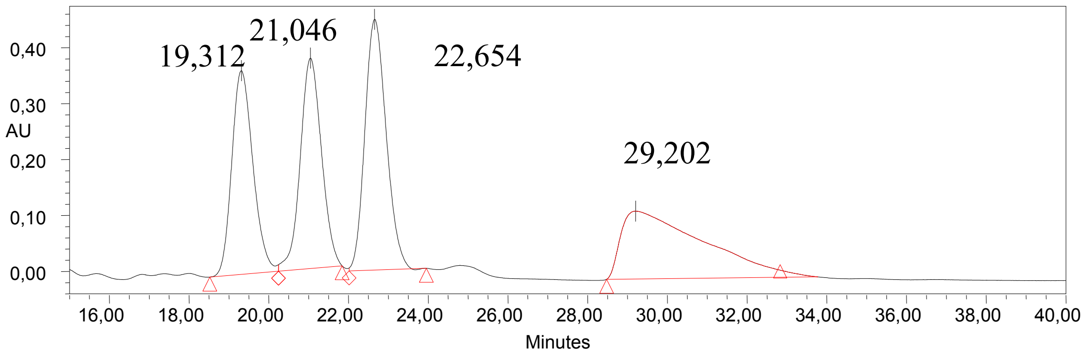

	Retention Time	% Area
1	19,312	21,87
2	21,046	22,64
3	22,654	27,16
4	29,202	28,33

7e

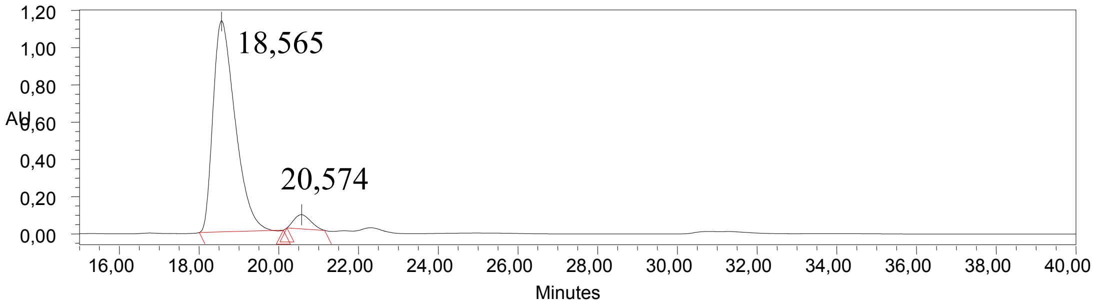

	Retention Time	% Area
1	18,565	95,14
2	20,574	4,86

90% *ee*

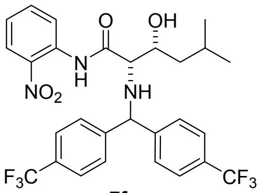

7f

Eluent: Hx/*i*PrOH, 98/2

Flow: 0.5 mL/min

Column: Phenomenex-Lux 3µm Amylose-1

λ: 254 nm

Rac-**7f**

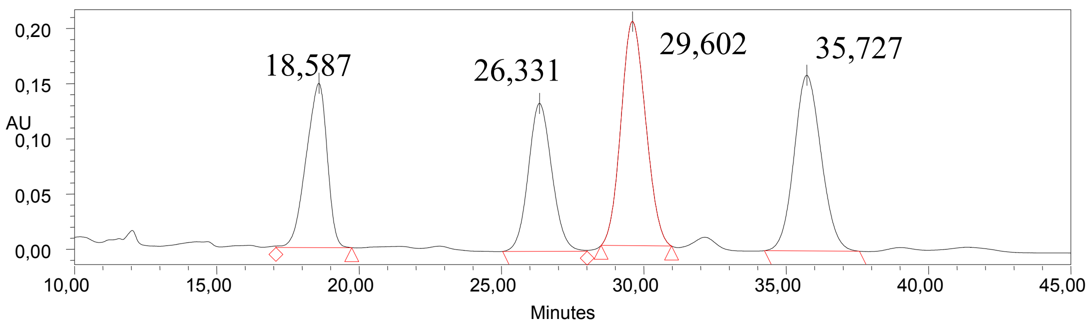

	Retention Time	% Area
1	18,587	20,01
2	26,331	20,19
3	29,602	32,13
4	35,727	27,68

7f

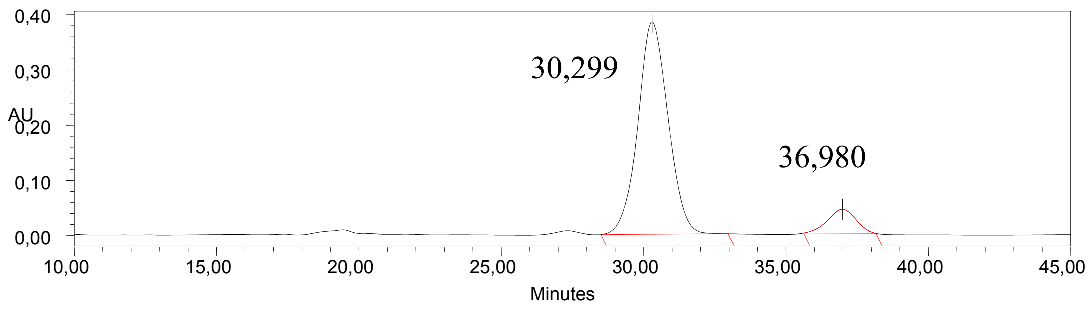

	Retention Time	% Area
1	30,299	92,49
2	36,980	7,51

84% *ee*

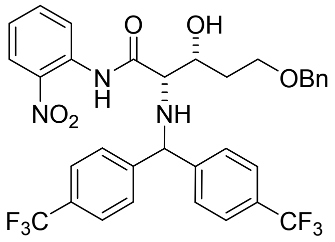

7g

Eluent: Hx/*i*PrOH, 95/5

Flow: 0.5 mL/min

Column: Phenomenex-Lux 3 μm Cellulose-1

λ: 254 nm

Rac-**7g**

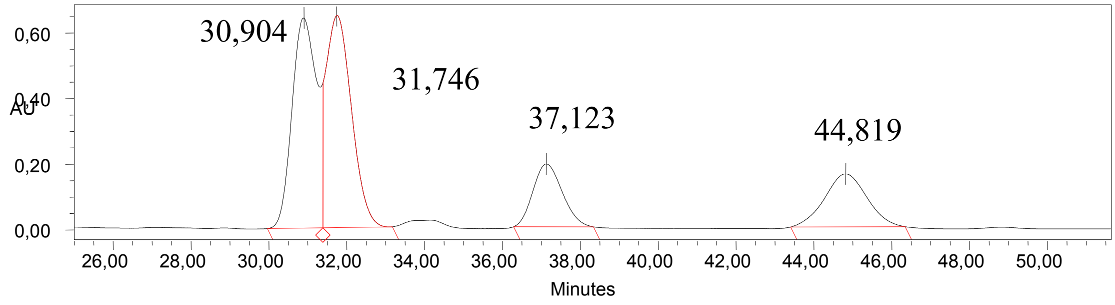

	Retention Time	% Area
1	30,904	35,84
2	31,746	36,45
3	37,123	12,52
4	44,819	15,20

7g

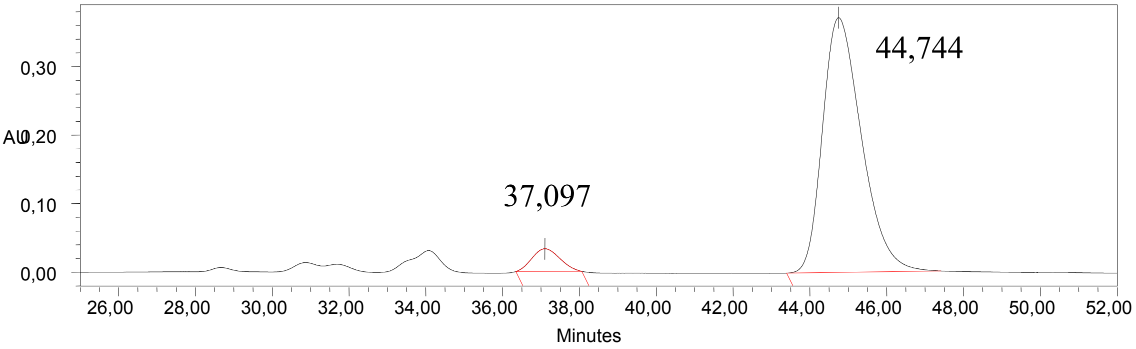

	Retention Time	% Area
1	37,106	5,31
2	44,744	94,69

90% *ee*

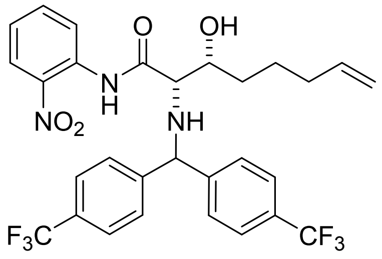

7h

Eluent: Hx/*i*PrOH, 98/2

Flow: 1 mL/min

Column: Phenomenex-Lux 3μm i-Cellulose-5

λ: 227 nm

Rac-**7h**

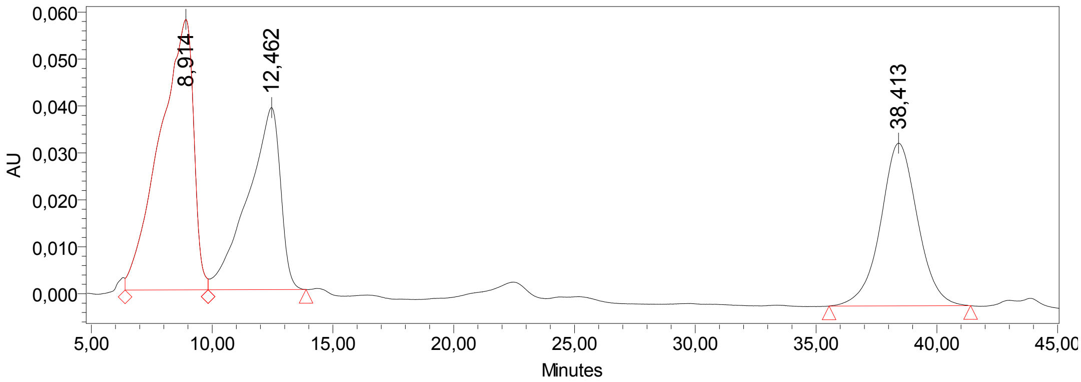

	Retention Time	% Area
1	8,914	43,38
2	12,462	28,21
3	38,413	28,41

7h

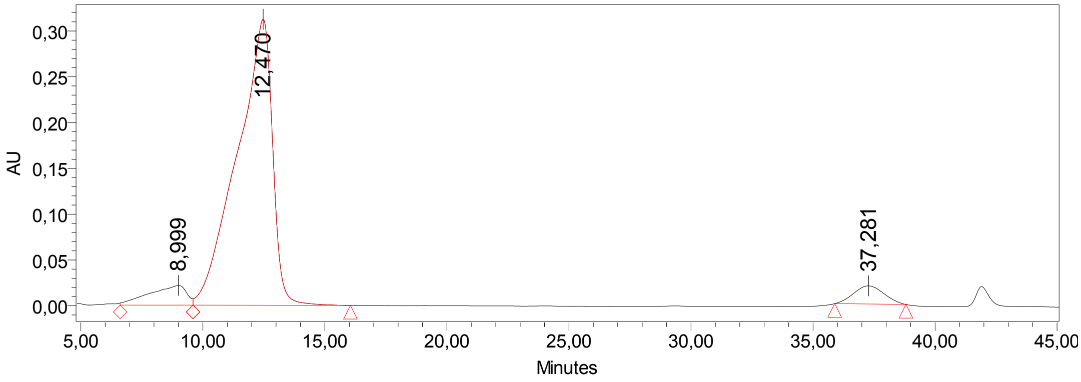

	Retention Time	% Area
1	8,999	6,40
2	12,470	88,76
3	37,281	4,84

	Retention Time	% Area
1	12,470	94,82
2	37,281	5,17

90% *ee*

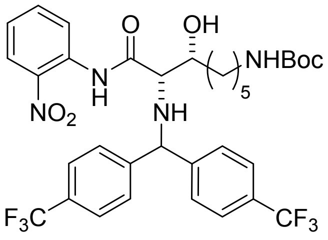

7i

Eluent: Hx/*i*PrOH, 90/10

Flow: 0.5 mL/min

Column: IA

λ: 254 nm

Rac-**7i**

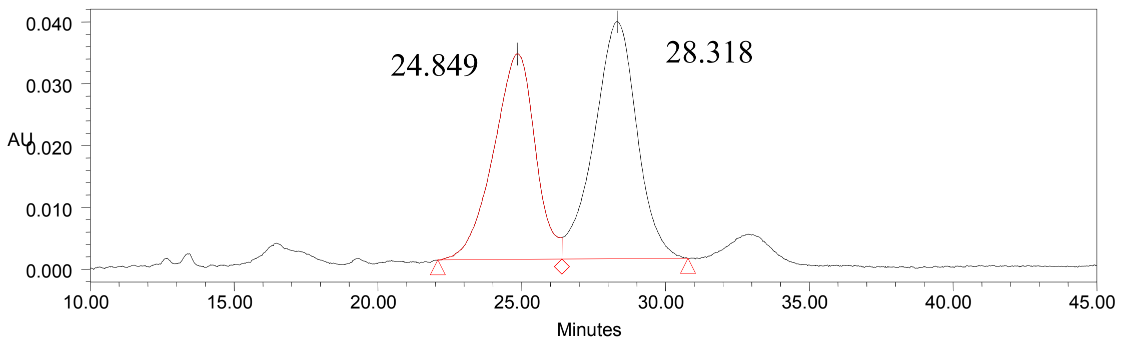

	Retention Time	% Area
1	24.861	45.25
2	28.333	54.75

7i

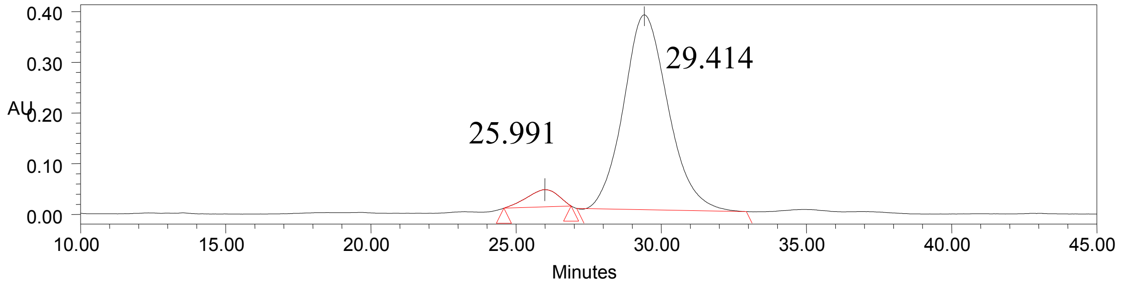

	Retention Time	% Area
1	25.991	5.85
2	29.414	94.15

88%*ee*

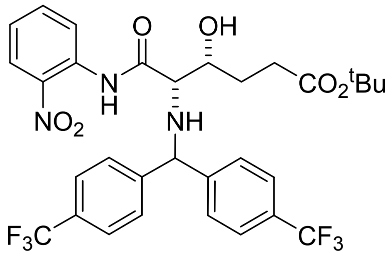

7j

Eluent: Hx/*i*PrOH, 98/2

Flow: 1 mL/min

Column: Phenomenex-Lux 3μm Amylose-1

λ: 227 nm

Rac-**7j**

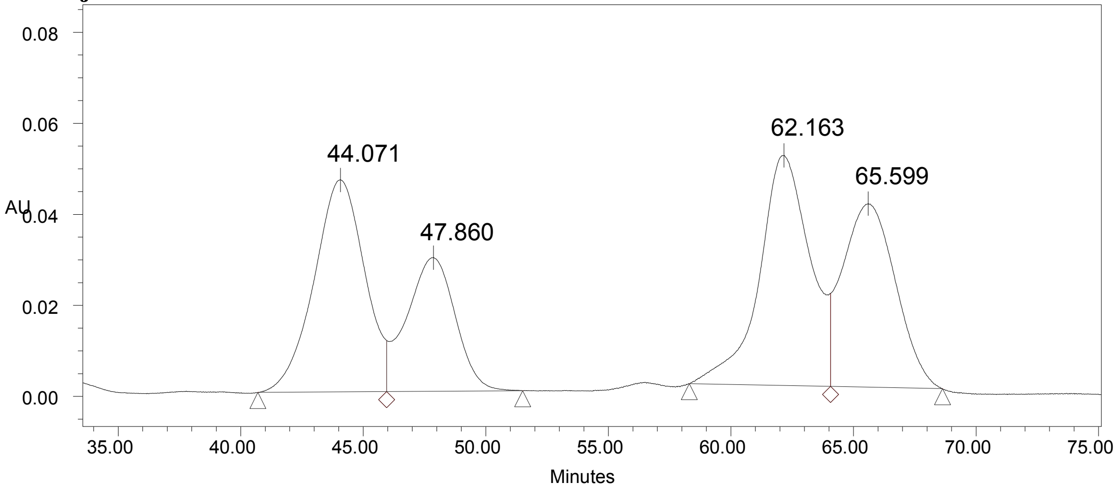

	Retention Time	% Area
1	44.071	27.70
2	47.860	19.89
3	62.163	30.38
4	65.599	23.03

7j

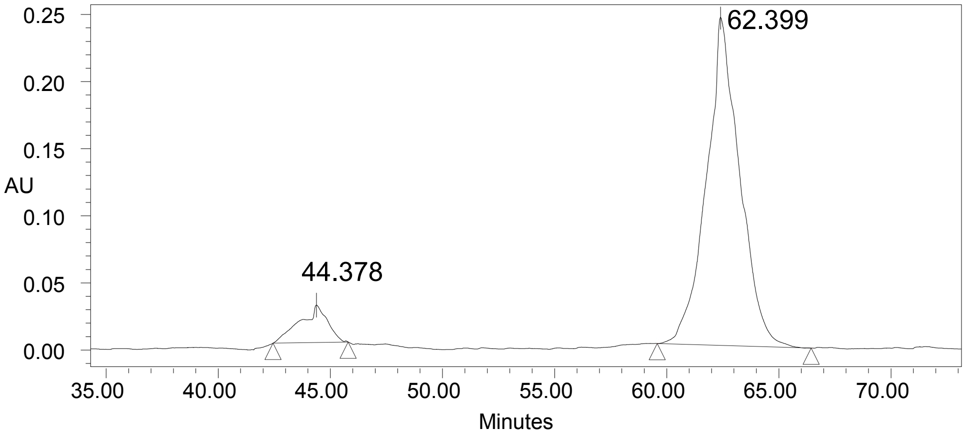

	Retention Time	% Area
1	44.378	7.62
2	62.399	92.38

85% *ee*

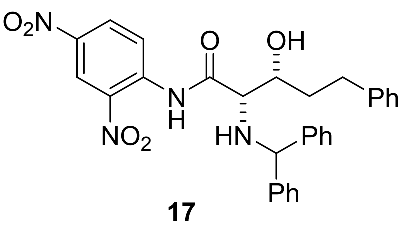

17

Eluent: Hx/EtOH, 90/10

Flow: 1 mL/min

Column: IF

λ: 254 nm

rac-**17**

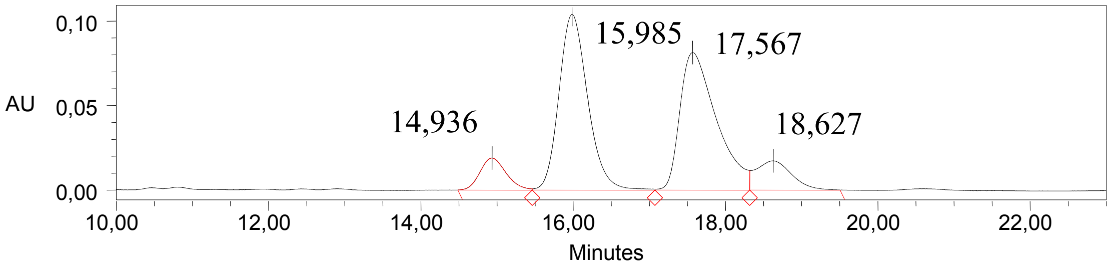

	Retention Time	% Area
1	14,936	6,98
2	15,985	42,26
3	17,567	42,09
4	18,627	8,68

17

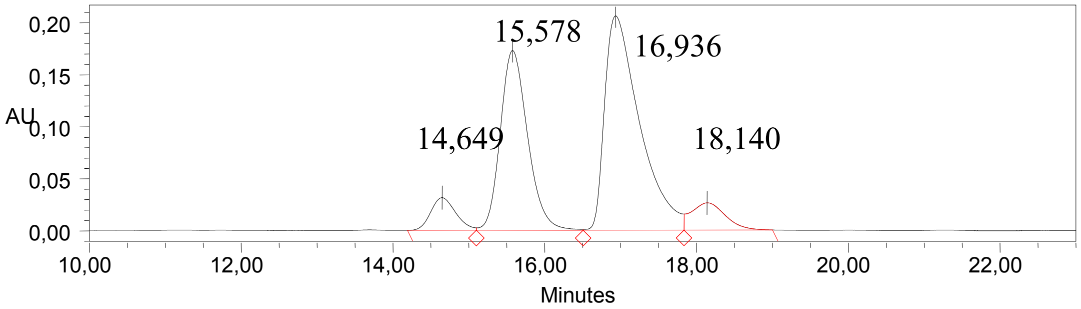

	Retention Time	% Area
1	14,649	5,76
2	15,578	34,74
3	16,936	52,97
4	18,140	6,53

20% *ee*
